# Reinventing metabolic pathways: Independent evolution of benzoxazinoids in flowering plants

**DOI:** 10.1073/pnas.2307981120

**Published:** 2023-10-09

**Authors:** Matilde Florean, Katrin Luck, Benke Hong, Yoko Nakamura, Sarah E. O’Connor, Tobias G. Köllner

**Affiliations:** ^a^Department of Natural Product Biosynthesis, Max Planck Institute for Chemical Ecology, Jena 07745, Germany; ^b^Research Group Biosynthesis/NMR, Max Planck Institute for Chemical Ecology, Jena 07745, Germany

**Keywords:** benzoxazinoids, evolution, pathway, biosynthesis, defense compounds

## Abstract

Benzoxazinoids (BXDs) form a class of indole-derived specialized plant metabolites with broad antimicrobial and antifeedant properties. Unlike most specialized metabolites, which are typically lineage-specific, BXDs occur sporadically in a number of distantly related plant orders. This observation suggests that BXD biosynthesis arose independently numerous times in the plant kingdom. However, although decades of research in the grasses have led to the elucidation of the BXD pathway in the monocots, the biosynthesis of BXDs in eudicots is unknown. Here, we used a metabolomic and transcriptomic-guided approach, in combination with pathway reconstitution in *Nicotiana benthamiana,* to identify and characterize the BXD biosynthetic pathways from both *Aphelandra squarrosa* and *Lamium galeobdolon*, two phylogenetically distant eudicot species. We show that BXD biosynthesis in *A. squarrosa* and *L. galeobdolon* utilize a dual-function flavin-containing monooxygenase in place of two distinct cytochrome P450s, as is the case in the grasses. In addition, we identified evolutionarily unrelated cytochrome P450s, a 2-oxoglutarate-dependent dioxygenase, a UDP-glucosyltransferase, and a methyltransferase that were also recruited into these BXD biosynthetic pathways. Our findings constitute the discovery of BXD pathways in eudicots. Moreover, the biosynthetic enzymes of these pathways clearly demonstrate that BXDs independently arose in the plant kingdom at least three times. The heterogeneous pool of identified BXD enzymes represents a remarkable example of metabolic plasticity, in which BXDs are synthesized according to a similar chemical logic, but with an entirely different set of metabolic enzymes.

## Significance

Enzymes that catalyze the same chemical reaction can evolve independently many times. However, examples of independent evolution of an entire pathway that consists of many enzymatic steps are rare. In this manuscript, we report the discovery of two biosynthetic pathways that both synthesize a class of plant defensive molecule called benzoxazinoid. Our discoveries demonstrate that the benzoxazinoid pathway has evolved independently in flowering plants at least three times. These findings provide a deeper understanding of the mechanisms underlying the evolution of metabolic pathways. In addition, the discovery of these benzoxazinoid biosynthetic pathways that have independently evolved in distantly related plants will allow us to probe the biological functions of these molecules.

Natural products, also referred to as specialized metabolites, allow plants to interact with their environment using the language of chemistry. Among specialized plant metabolites, the indole containing benzoxazinoids (BXDs) are well known for their varied and versatile biological functions. These compounds have long been known to be toxic against a wide range of insects and herbivores, but more recent work has shown the BXDs also act as allelochemicals and can promote iron uptake from soil as phytosiderophores (1–3). BXDs are often referred to as the typical specialized metabolites of the grasses (Poaceae), a monocot family that includes agronomically important species such as maize (*Zea mays*), wheat (*Triticum aestivum*), and rye (*Secale cereale*). However, although BXDs are best studied in the grasses, the appearance of this group of specialized metabolites is not restricted to this family. BXDs also occur in a number of eudicot species belonging to the Ranunculaceae, Lamiaceae, Acanthaceae, Plantaginaceae, Calceolariaceae, and Apocynaceae families (4–8).

**Fig. 1.**
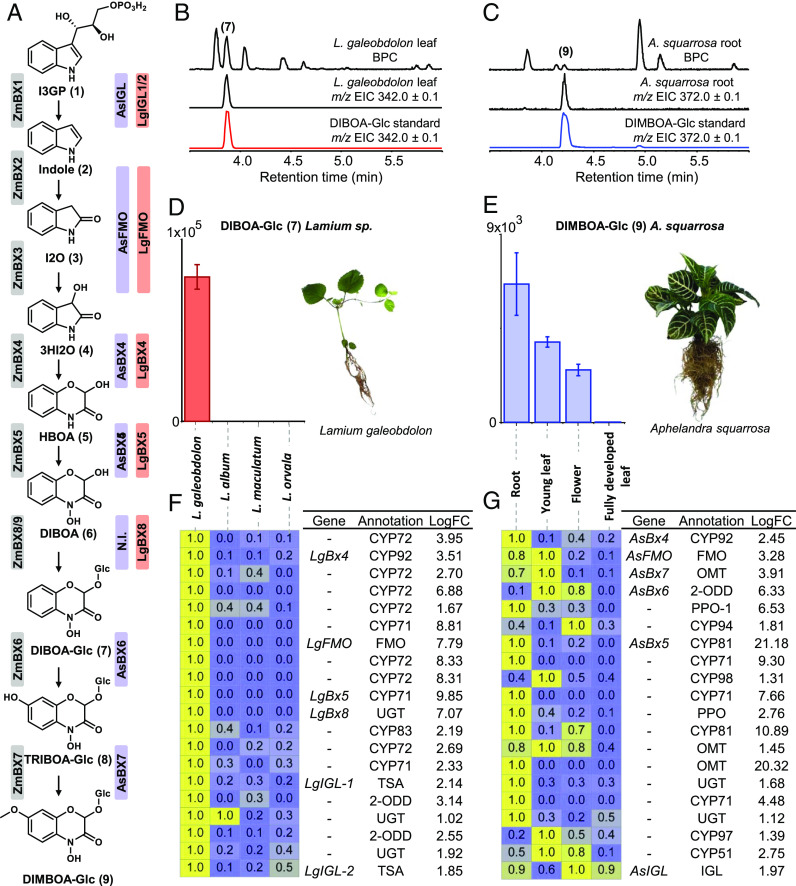
Identification of *Bx* genes in *L. galeobdolon* and *A. squarrosa.* (*A*) BXD pathway in *Z. mays*, *L. galeobdolon,* and *A. squarrosa*. N.I.: Not Identified. (*B* and *C*) LC-qTOF of methanol extracts made from *L. galeobdolon* leaves (*B*) and *A. squarrosa* roots (*C*). Accumulation of DIBOA-Glc (7) and DIMBOA-Glc (9) was confirmed with authentic standards. BPC, base peak chromatogram; EIC, extracted ion chromatogram. (*D* and *E*) Accumulation of DIBOA-Glc in leaves of different *Lamium* species (*D*) and DIMBOA-Glc abundance in different *A. squarrosa* tissues (*E*). Methanol extracts were analyzed using LC-qTOF and average peak area ± SE (n = 3) is shown. (*F* and *G*) Relative expression of candidate genes from *Lamium sp.* and *A. squarrosa*. Values from 0 (lowest) to 1 (highest) indicate the relative expression of each gene among the tested conditions. Genes with RPKM values > 10 and LogFC > 1 in BXD-accumulating species or tissues were selected and then sorted from the highest to lowest expression. The first top 20 candidates from *L. galeobdolon* and *A. squarrosa* are displayed. Previously identified *LgIGL-1* and *LgIGL-2* and *AsIGL* that did not fit the selection parameters were added to the table manually. Enzyme abbreviations: cytochrome P450 (CYP), flavin-containing monooxygenase (FMO), UDP-glucosyl transferase (UGT), tryptophan synthase α-subunit (TSA), 2-oxoglutarate-dependent dioxygenase (2-ODD), polyphenol oxidase (PPO), *O*-methyltransferase (OMT).

The relevance of BXDs as major defensive compounds in maize prompted the elucidation of their biosynthesis in this species (Fig. 1*A*) (1, 9–12). The pathway starts with the formation of indole from indole 3-glycerol phosphate, an intermediate in tryptophan biosynthesis, by action of indole 3-glycerol phosphate lyase (IGL), also known as benzoxazinoneless 1 (ZmBX1). Indole then undergoes a series of sequential oxidations catalyzed by four cytochrome P450 monooxygenases (CYP) named ZmBX2, ZmBX3, ZmBX4, and ZmBX5, respectively (Fig. 1*A*). ZmBX2 converts indole to indolin-2-one (I2O), which is further metabolized by ZmBX3 to 3-hydroxy indolin-2-one (3HI2O). ZmBX4 catalyzes a hydroxylation of 3HI2O and a subsequent ring expansion to produce 2-hydroxy-3,4-dihydro-1,4-benzoxazin-3-one (HBOA), while ZmBX5 mediates the N-hydroxylation of HBOA to 2,4-dihydroxy-1,4-benzoxazin-3-one (DIBOA). DIBOA is then glucosylated by the UDP-glucosyltransferases (UGT) ZmBX8 and ZmBX9, which quench BXD aglucone reactivity and promote BXD storage as stable glucosides in the vacuole. DIBOA-Glc exhibits substantial defensive properties, and this molecule constitutes the end product of the pathway in several grasses (1, 3, 13, 14). In other species, including maize, DIBOA-Glc can undergo a further hydroxylation that is catalyzed by the 2-oxoglutarate-dependent dioxygenase (2-ODD) ZmBX6, and the resulting product, 2-(2,4,7-trihydroxy-1,4-benzoxazin- 3-one)-β-D-glucopyranose (TRIBOA-Glc), is finally methylated by the *O*-methyltransferase (OMT) ZmBX7 to form 2-(2,4-dihydroxy-7-methoxy-1,4-benzoxazin-3-one)-β-D-glucopyranose (DIMBOA-Glc) (11) (Fig. 1*A*). While DIMBOA-Glc represents the major BXD in young and undamaged maize, insect herbivory or pathogen infestation induces its conversion to 2-(2-hydroxy-4, 7-dimethoxy-1, 4-benzoxazin-3-one)-β-D-glucopyranose (HDMBOA- Glc) and other hydroxylated and methoxylated BXDs (15–18). The involved enzymes, four OMTs (ZmBX10, ZmBX11, ZmBX12, and ZmBX14) and the 2-ODD ZmBX13, have recently been identified in maize (17, 18).

Identification and characterization of BXD genes from additional grass species revealed that the core BXD pathway leading to DIMBOA-Glc is of monophyletic origin. Both the evolution of BX1 from the tryptophan synthase α-subunit (TSA) and the evolution of BX3, BX4, and BX5 from a BX2-like ancestor most likely preceded the radiation of the Poaceae (19, 20), indicating that the pathway evolved in an early grass species or even before the grasses arose. In contrast, the OMT BX10, which converts DIMBOA-Glc to HDMBOA-Glc, has evolved at least twice during grass evolution (21). Despite the extensive knowledge of BXD formation in the grasses, little is known about their origin and evolution in eudicots. In principle, BXDs could have arisen early in the history of the angiosperms and then been lost in multiple taxa, or alternatively, BXDs could have arisen independently several times during angiosperm evolution. Examples for both evolutionary trajectories have already been reported for specialized metabolites in plants (22–27). Previous studies using radiolabeled BXD precursors have shown that the BXD pathway in dicotyledonous plants most likely starts from indole, as in maize (28). However, the recent identification and phylogenetic analysis of IGLs, the predicted first enzyme of the BXD pathway, from *Lamium galeobdolon* (Lamiaceae), *Aphelandra squarrosa* (Acanthaceae), and *Consolida orientalis* (Ranunculaceae), three BXD-producing eudicot species, strongly suggests independent evolution of IGL activity between eudicots and monocots (28).

The aim of our study was to elucidate the formation of BXDs in two nonrelated eudicot species to address the origin of this pathway in the angiosperms. Integrated metabolomics and transcriptomics analyses of *A. squarrosa* and *L. galeobdolon* enabled us to identify candidate genes potentially involved in BXD biosynthesis. By expressing the maize BXD pathway in *Nicotiana benthamiana* and replacing individual maize genes with candidate genes from *A. squarrosa* and *L. galeobdolon*, we established a screening platform that allowed rapid testing of candidates for each step of the pathway. Our results indicate that the BXD pathway evolved independently in *Z. mays*, *A. squarrosa*, and *L. galeobdolon*, making this specialized metabolite pathway an excellent model for studying the mechanisms underlying pathway evolution in plants.

## Results

### Identification of Candidate BXD Genes in *A. squarrosa* and *L. galeobdolon*.

*A. squarrosa* and *L. galeobdolon* have been described to produce significant amounts of BXDs, with *A. squarrosa* accumulating DIBOA-Glc and DIMBOA-Glc in roots and *L. galeobdolon* accumulating exclusively DIBOA-Glc, but throughout the entire plant (28). In our lab, DIBOA-Glc and DIMBOA-Glc could be detected in roots of *A. squarrosa*, though also in young leaves and flowers. Fully developed leaves, however, showed no BXD accumulation. In *L. galeobdolon*, we could only detect DIBOA-Glc, which accumulated in all tested tissues (Fig. 1 *B*–*E* and *SI Appendix*, Fig. S1).

To identify candidate BXD genes in *A. squarrosa*, we sequenced the transcriptomes of fully developed leaves, young leaves, flowers, and roots, constructed a de novo assembly, and selected genes that were expressed in roots with RPKM values higher than 10. Fold change (FC) between BXD-accumulating tissues (roots, young leaves, or flowers) and the BXD non-accumulating fully developed leaves were calculated. Genes with LogFC >1 were selected and sorted by highest expression. Among the resulting 90 genes were 40 CYPs, 1 flavin-dependent monooxygenase (FMO), 15 2-ODDs, 5 polyphenol oxidases (PPOs), 2 hydroxylases, 6 UGTs, and 21 OMTs (Fig. 1*G* and *SI Appendix*, Fig. S2*A*), which were all considered for further characterization.

Since *L. galeobdolon* accumulated DIBOA-Glc in all organs (*SI Appendix*, Fig. S1*C*), the comparative analysis approach used for *A. squarrosa* would have been more challenging in this species. Therefore, we screened other *Lamium* species for BXD production. We identified three nonproducers, *L. album*, *L. maculatum*, and *L. orvala,* that are closely related to *L. galeobdolon* (Fig. 1*D* and *SI Appendix*, Fig. S1*A*). By sequencing the leaf transcriptomes of all four *Lamium* species and sorting transcripts with the same parameters used for the transcriptomic analysis of *A. squarrosa*, we identified 57 genes including 40 CYPs, 2 FMOs, 4 2-ODDs, 1 IGL, and 10 UGTs, that showed high expression in *L. galeobdolon* leaves, but no or nearly no expression in leaves of *L. album*, *L. maculatum*, and *L. orvala* (Fig. 1*F* and *SI Appendix*, Fig. S2*B*).

### Establishing *N. benthamiana* as an Expression Platform for Fast BXD Gene Screening.

A major bottleneck in elucidating metabolic pathways is that pathway intermediates are often commercially unavailable or difficult to synthesize and are therefore not available as substrates for in vitro enzyme assays. To facilitate the BXD gene discovery process, we established *N. benthamiana* as a platform for fast gene screening by expressing maize *ZmBx1*-*8* in various combinations with candidate BXD genes from *L. galeobdolon* and *A. squarrosa*. We demonstrated that *Agrobacterium tumefaciens*-mediated transient expression of the entire maize pathway consisting of *ZmBx1*-*8* led to the accumulation of DIMBOA-Glc in infiltrated *N. benthamiana* leaves, as revealed by liquid chromatography-quadrupole-time-of-flight mass spectrometry (LC-qTOF) of methanolic *N. benthamiana* leaf extracts (*SI Appendix*, Fig. S3). In addition, we detected I2O and glucosylated forms of all other pathway intermediates (3HI2O-Glc, HBOA-Glc, DIBOA-Glc, and TRIBOA-Glc) (*SI Appendix*, Fig. S3) when different combinations of the maize BX enzymes were expressed. These results show that *N. benthamiana* is an efficient expression system for BXD gene screening. Note that due to the unstable nature of the BXD aglucones (2), we added the UDP-glucosyltransferase BX8 (UDP, uridine diphosphate) to each combination of expressed genes, also in the case of the screening of *Bx* candidate genes described in the next paragraphs.

### Flavin-Dependent Monooxygenases Catalyze the Formation of 3HI2O from Indole in *A. squarrosa* and *L. galeobdolon*.

**Fig. 2.**
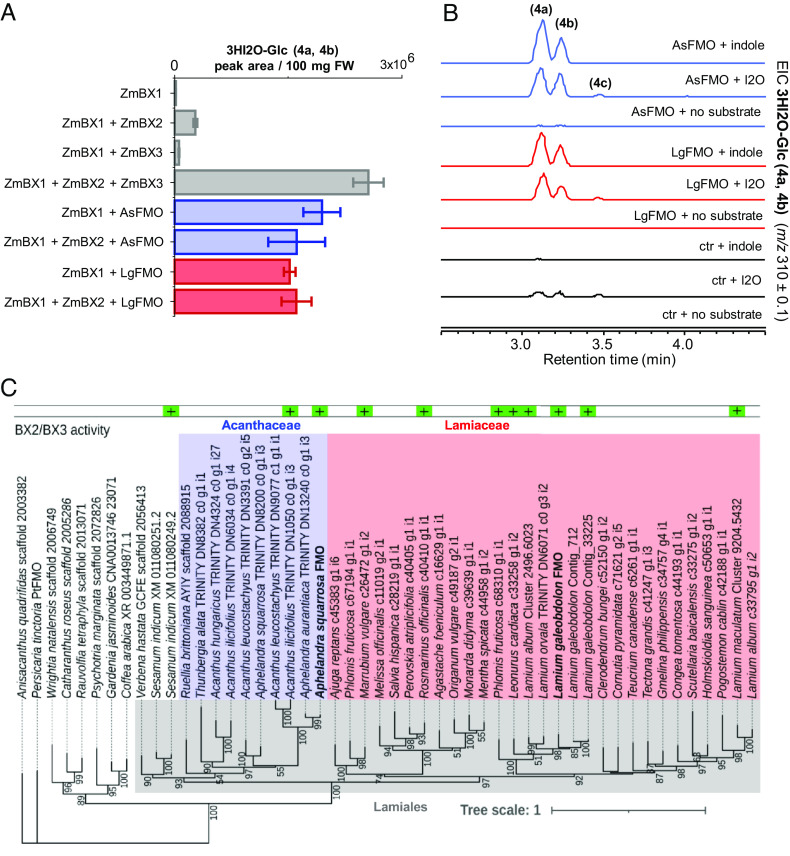
AsFMO and LgFMO act as bifunctional enzymes both on indole and I2O. (*A*) AsFMO and LgFMO can use both indole and I2O as substrate for 3HI2O-Glc biosynthesis. The bar graph displays 3HI2O-Glc (4a and 4b) peak area (average ± SE, n = 3) of methanolic extracts of *N. benthamiana* leaves coinfiltrated with different combinations of BXD genes. Methanol extracts were analyzed using LC-qTOF. 3HI2O was detected as two glycosylated isomers (4a and 4b, *SI Appendix*, Fig. S3). Trace levels of putative 5HI2O-Glc (4c) were detected when I2O was used as a substrate. (*B*) Expression of *AsFMO* and *LgFMO* in *N. benthamiana* and coinfiltration with indole or I2O led to the production of 3HI2O-Glc (4a and 4b). *AsFMO* and *LgFMO*, respectively, were transiently expressed together with *ZmBx8* in *N. benthamiana*. Controls (ctr) consisted of transient expression of *ZmBx8* only. Three days post transformation, indole, I2O, or infiltration buffer were infiltrated in the transformed leaves. Methanol extracts were analyzed using LC-qTOF. EIC, extracted ion chromatogram. (*C*) Maximum likelihood phylogenetic analysis of FMO proteins from several eudicot species. Sequences with a colored box on the top were tested for BX2/BX3 activity in *N. benthamiana* [green (+) = active, red (−) = non active]. The gray box indicates species belonging to the order Lamiales.

In maize and other grasses, after the generation of indole from indole 3-glycerol phosphate, two distinct CYPs (BX2 and BX3) sequentially oxidize indole to I2O and 3HI2O (Fig. 1*A*) (9). None of the tested CYP candidate genes from *A. squarrosa* and *L. galeobdolon* were able to functionally substitute for *Bx2* or *Bx3* when expressed together with the maize IGL *ZmBx1* alone or with *ZmBx1* and the downstream maize oxidase gene *ZmBx2* in *N. benthamiana.* However, we identified a gene annotated as a flavoenzyme (FMO) in both *A. squarrosa* (*AsFMO*) and in *L. galeobdolon* (*LgFMO*), both of which allowed the production of 3HI2O when expressed with *ZmBx1* or *ZmBx1-2* (Fig. 2*A*). To identify the substrate of these FMOs, we infiltrated either indole or I2O into *N. benthamiana* leaves transiently transformed with *AsFMO* or *LgFMO*. Infiltration with either indole or I2O always led to the accumulation of 3HI2O-Glc, the glucosylated form of known BXD intermediate 3HI2O (Fig. 2*B* and *SI Appendix*, Fig. S4*B*). This indicated that AsFMO and LgFMO are bifunctional enzymes catalyzing a two-step oxidation of indole to 3HI2O, most likely via I2O as an intermediate. Infiltration of I2O into *N. benthamiana* plants expressing only *ZmBx8* (negative control) resulted in small amounts of 3HI2O-Glc, indicating that *N. benthamiana* is able to compensate for BX3 activity, albeit at very low levels (Fig. 2 *A* and *B*). 3HI2O was detected in *N. benthamiana* leaf extracts as glycoside forming two peaks, which we here hypothesize being isomers or epimers of 3HI2O-Glc (*SI Appendix*, Fig. S3). Interestingly, when *N. benthamiana* leaves were fed with I2O, a third compound with the same mass as 3HI2O-Glc was detected (Fig. 2*B*, peak 4c). Since the presence of a hydroxylated derivative of I2O, indolin-2-one-5-β-D-glucopyranoside (5HI2O-Glc), has already been reported in *Arabidopsis thaliana* overexpressing maize *ZmBx1* and *ZmBx2* (12), we hypothesize that this compound detected in *N. benthamiana* corresponds to 5HI2O-Glc, which is likely produced by an endogenous oxidase activity. All attempts to isolate active recombinant AsFMO and LgFMO from *N. benthamiana* leaves or from the other heterologous hosts *Escherichia coli*, *Saccharomyces cerevisiae*, and *Spodoptera frugiperda* Sf9 cells were not successful, so further mechanistic studies were not performed.

### AsFMO and LgFMO Belong to a Family of Lamiales FMOs with Conserved BX2/BX3 Activity.

Phylogenetic analysis of AsFMO and LgFMO revealed that these enzymes belong to two separate family-specific clades (Fig. 2*C*). To see whether related FMOs from other species in the Lamiales also have BX2/BX3 activity, we tested a number of candidates by expressing them with *ZmBx1* using our *N. benthamiana* expression system. Surprisingly, all AsFMO/LgFMO-like enzymes tested showed BX2/BX3 activity, whether or not the plants from which they were derived produce BXDs and regardless of the percentage of sequence identity between them (spanning between 60 and 89%) (Fig. 2*C* and *SI Appendix*, Fig. S4*A* and Table S1*A*). Specifically, the FMO from the BXD producer *Acanthus ilicifolius* (TRINITY DN6034 c0 g1 i4) converted indole to 3HI2O in the same manner as FMOs from the nonproducing species *L. album* (Cluster 2496.6023), *Leonurus cardica* (c33258 g1 i2), *Phlomis fruticosa* (c68310 g1 i1), *Rosmarinus officinalis* (c40410 g1 i1), and *Marrubium vulgare* (c26472 g1 i2). Even a related FMO (XM011080249.2) from *Sesamum indicum,* a species belonging to another Lamiales family (Pedaliaceae), showed 3HI2O biosynthetic activity when tested in *N. benthamiana* with ZmBX1 (*SI Appendix*, Fig. S4). The percentages of amino acid sequence identities of all tested enzymes are reported in *SI Appendix*, Table S1. These results suggest that BX2/BX3 activity is widespread, at least in the Lamiales, and not restricted to BXD-producing plants.

### *A. squarrosa* and *L. galeobdolon* Both Possess a CYP92 Enzyme Able to Convert 3HI2O to HBOA.

**Fig. 3.**
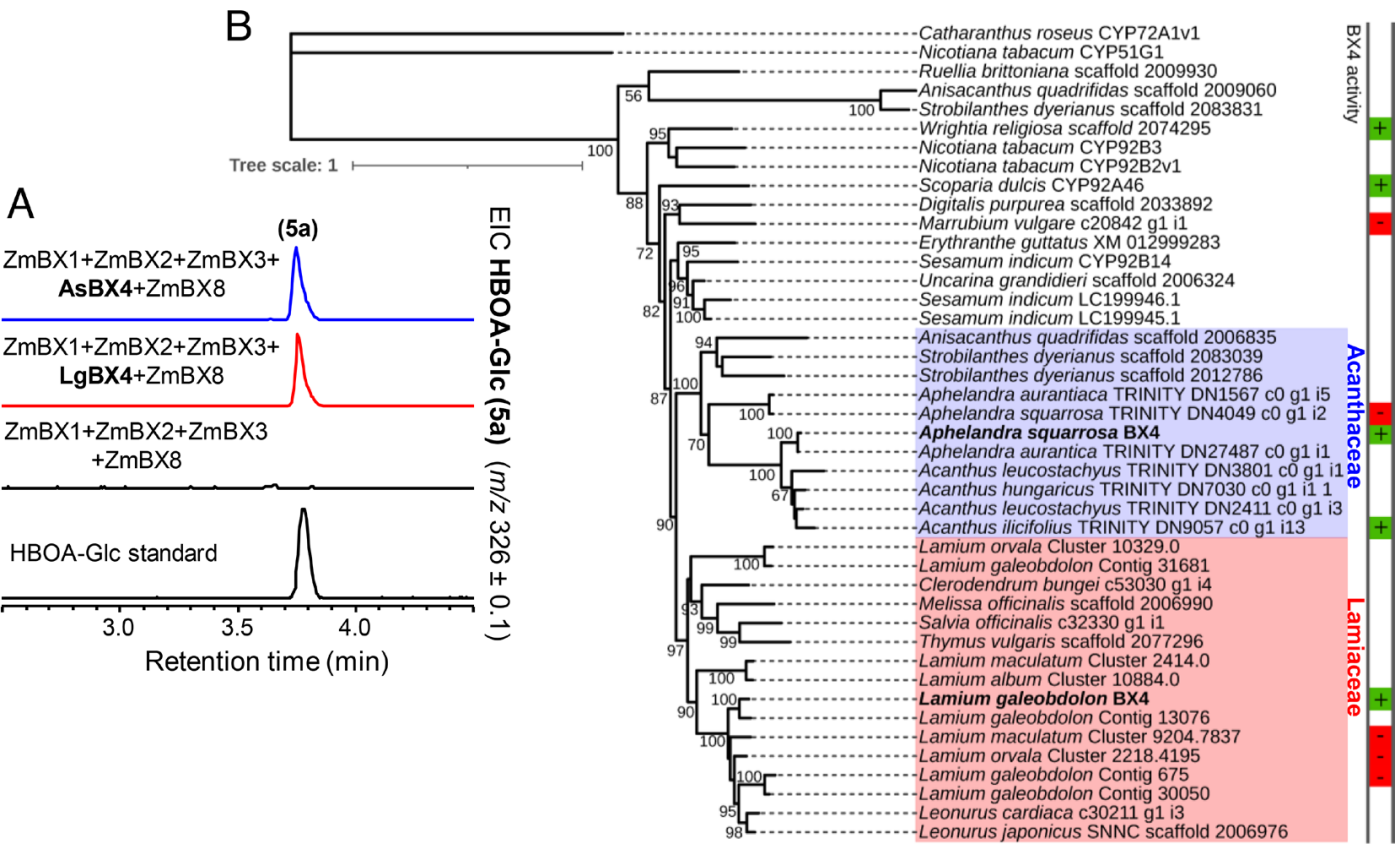
AsBX4 and LgBX4 produce HBOA-Glc (5a). (*A*) Expression of *AsBx4* and *LgBx4*, respectively, with *ZmBx1-3 + ZmBx8* in *N. benthamiana* resulted in HBOA-Glc (5a, *SI Appendix*, Fig. S3) accumulation. Methanol extracts of *N. benthamiana* leaves were analyzed using LC-qTOF. HBOA-Glc (5a) accumulation was confirmed with an authentic standard. EIC, extracted ion chromatogram. (*B*) Maximum likelihood phylogenetic analysis of AsBX4, LgBX4, and CYP92 proteins from other plants. Sequences with a colored box next to their names were tested for BX4 activity [green (+) = active, red (−) = non active] in *N. benthamiana*.

In the biosynthesis of BXDs in the Poaceae, 3HI2O undergoes an oxidative ring expansion reaction that generates the intermediate HBOA (Fig. 1*A*) (29). This reaction is catalyzed by BX4, a cytochrome P450 belonging to the CYP71C subfamily (9). In both *Aphelandra* and *Lamium,* we identified a CYP92 enzyme (AsBX4 and LgBX4, respectively) capable of converting 3HI2O into HBOA. AsBX4 and LgBX4 showed BX4 activity both in vivo when expressed in *N. benthamiana* together with *ZmBx1-3* + *ZmBx8* (Fig. 3*A*) and in vitro when expressed in *S. cerevisiae* and tested as microsomal preparations on chemically synthesized 3HI2O (*SI Appendix*, Fig. S5*A*). Phylogenetic analysis revealed that AsBX4 and LgBX4 cluster in two family-specific clades and are more related to other Acanthaceae and Lamiaceae CYP92 enzymes, respectively, than to each other (Fig. 3*B*). *Scoparia dulcis* and *Wrightia religiosa,* belonging to the Plantaginaceae and Apocynaceae families, respectively, have recently been described to produce BXDs (5, 7, 30). We could also identify two CYP92s in the publicly available transcriptomes of these species that showed BX4 activity when expressed in *N. benthamiana* (Fig. 3*B* and *SI Appendix*, Fig. S5*B*). However, closely related CYP92s from *M. vulgare, A. squarrosa,* and different *Lamium* species did not show BX4 activity (Fig. 3*B* and *SI Appendix*, Fig. S5*B* and Table S1*B*).

### A CYP81 and a CYP71D Convert HBOA into DIBOA in *A. squarrosa* and *L. galeobdolon*, Respectively.

**Fig. 4.**
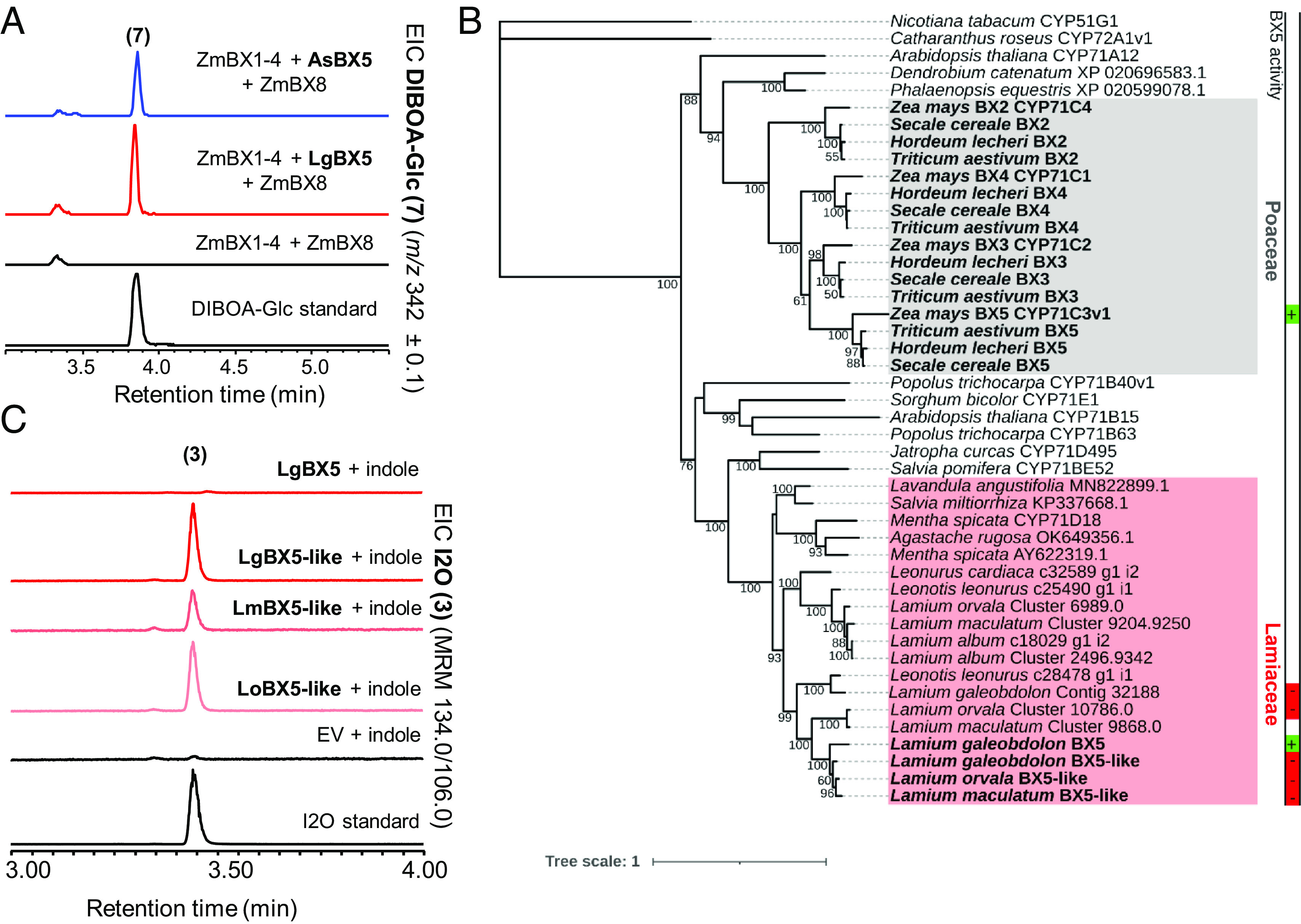
Identification and characterization of BX5 and BX5-like activities in *L. galeobdolon* and *A. squarrosa*. (*A*) AsBX5 (CYP81) and LgBX5 (CYP71) allow the production of DIBOA-Glc (7, *SI Appendix*, Fig. S6) in *N. benthamiana*. *AsBx5* and *LgBx5*, respectively, were transiently expressed with *ZmBx1-4 + ZmBx8* in *N. benthamiana* leaves and DIBOA-Glc was analyzed using LC-qTOF. EIC, extracted ion chromatogram. (*B*) Maximum likelihood phylogenetic analysis of LgBX5, ZmBX5, and related CYP71 proteins from different monocot and eudicot species. A green or red box next to the sequence names in the tree indicates presence [green (+)] or absence [red (−)] of BX5 activity in *N. benthamiana*. (*C*) BX5-like enzymes from different *Lamium* species showed low but specific BX2 activity in vitro. Genes were expressed in *S. cerevisiae* and microsomes were incubated with indole and NADPH. Produced I2O (3) was analyzed using LC-MS/MS.

The *N*-hydroxylation of HBOA is catalyzed by a CYP71, BX5, in the Poaceae (9). We found a CYP81 in *A. squarrosa* (AsBX5) and a CYP71 in *L. galeobdolon* (LgBX5) that were able to functionally replace maize ZmBX5 in our *N. benthamiana* expression system (Fig. 4*A*). Unfortunately, all attempts to express *AsBx5* and *LgBx5* in yeast were unsuccessful, so we were unable to characterize these enzymes in vitro. AsBX5 and LgBX5 share only 29.5% amino acid sequence similarity and belong to two different CYP families, strongly suggesting independent evolution of BX5 activity in the Acanthaceae and Lamiaceae. Moreover, although LgBX5 and the Poaceae BX5 enzymes belong to the same CYP family, they share very low amino acid sequence identity (32.7% between LgBX5 and ZmBX5). Phylogenetic analysis showed that LgBX5 clustered together with other eudicot CYP71s, including a number of well-characterized enzymes known to be involved in other metabolic pathways, such as CYP71B40 from *Populus trichocarpa* and CYP71E1 from *Sorghum bicolor*, which both convert aldoximes to nitriles (31, 32). In contrast, the Poaceae BX2-5 enzymes formed a well-defined clade with other monocot CYP71s and CYP71A12 from *A. thaliana* (Fig. 4*B*). This, together with the fact that three enzymes with high sequence similarity to BX5 found in *L. galeobdolon* (LgBX5-like), *L. maculatum* (LmBX5-like), and *L. orvala* (LoBX5-like), respectively, did not show BX5 activity (Fig. 4*B* and *SI Appendix*, Fig. S6*A* and Table S1*C*), suggests that this activity arose only in *L. galeobdolon* and independently of the BX5 activity in the Poaceae.

### LgBX5-Like Has Activity as Indole Oxidase In Vitro, but Not In Planta.

In *L. galeobdolon,* we also identified an additional gene with 94% nucleotide sequence identity to *LgBx5*. Very closely related sequences to this gene, designated *LgBx5-like*, could also be cloned from *L. maculatum* (*LmBx5-like*) and *L. orvala* (*LoBx5-like*). In contrast to LgBx5, which was highly expressed in *L. galeobdolon* leaves, *LgBx5-like* showed only trace expression in this organ (*SI Appendix*, Fig. S6*E*). However, *Bx5-like* genes were highly expressed in BXD-free *Lamium* species. Despite the high sequence similarity of *LgBx5-like, LmBx5-like,* and *LoBx5-like* with *LgBx5* (91% on average), the encoded proteins LgBX5-like, LmBX5-like, and LoBX5-like (which shared 86% AA sequence identity) were unable to functionally replace ZmBX5 in our *N. benthamiana* expression system (*SI Appendix*, Fig. S6*A*). Interestingly, microsome preparations of *S. cerevisiae* expressing *Bx5-like* genes incubated with indole as substrate and NADPH as cosubstrate, showed I2O biosynthetic (BX2) activity (Fig. 4*C*). This activity was much lower compared to ZmBX2, but clearly distinguishable from the noise of the empty vector control (*SI Appendix*, Fig. S6*F*). To test whether the low BX2 activity of BX5-like enzymes observed in vitro is of biological relevance, we expressed them with maize *ZmBx1 + ZmBx3-5 + ZmBx8* in *N. benthamiana.* However, neither DIBOA-Glc nor any other pathway intermediate could be detected (*SI Appendix*, Fig. S6*B*), suggesting that the kinetic properties of LgBX5-like, LmBX5-like, and LoBX5-like are not sufficient to drive indole oxidation under natural substrate concentrations.

### *L. galeobdolon* BX8 Belongs to the UGT85 Family.

In maize, the formation of DIBOA is followed by glucosylation (Fig. 1*A*), and two UDP-glucosyl transferases belonging to the UGT710 family, named ZmBX8 and ZmBX9, have been proven to be involved in this process (33, 34). In *L. galeobdolon,* we identified a UGT85, LgBX8, sharing only 31.4% amino acid sequence identity with ZmBX8 (*SI Appendix*, Table S1*F*). LgBX8 was able to catalyze BXD glucosylation when expressed with maize *ZmBx1-5* transiently in *N. benthamiana* leaves (*SI Appendix*, Fig. S7*A*). Moreover, in vitro characterization of LgBX8 revealed that this transferase accepted both HBOA and DIBOA as substrates, but not 3HI2O (*SI Appendix*, Fig. S7 *B*–*D*). Despite extensive screening, we were not able to identify a UGT in *A. squarrosa* that glucosylates BXDs.

### A 2-ODD Acts as BX6 in *A. squarrosa*.

**Fig. 5.**
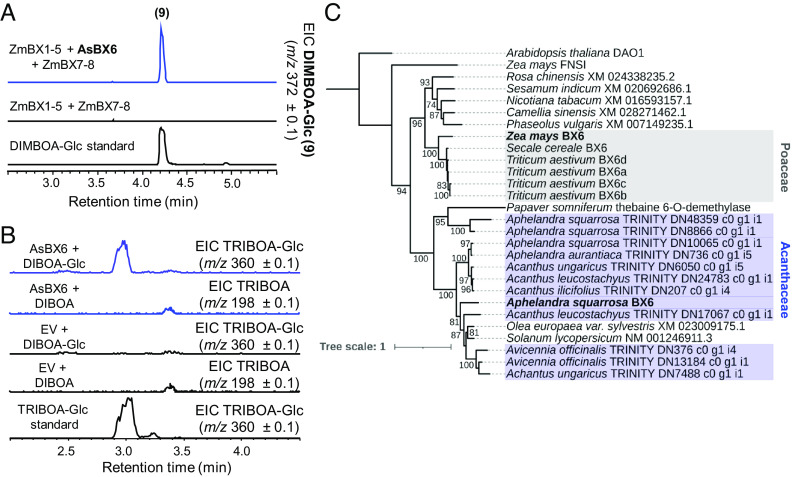
*A. squarrosa* possesses a 2-ODD with BX6 activity. (*A*) *AsBx6* was transiently expressed with *ZmBx1-5 + ZmBx7-8* in *N. benthamiana.* Leaf methanol extracts were analyzed using LC-qTOF. (*B*) In vitro characterization of AsBX6. The gene was expressed in *E. coli* and purified AsBX6 was tested with DIBOA-Glc and DIBOA as potential substrates. Enzyme products were analyzed using LC-qTOF. EIC, extracted ion chromatogram; EV, empty vector control. (*C*) Maximum likelihood phylogenetic analysis of AsBX6, ZmBX6, and related 2-ODD proteins from different monocot and eudicot species.

While the BXD pathway in *L. galeobdolon* stops at DIBOA-Glc, the BXD pathways in maize and *A. squarrosa* proceed with hydroxylation of DIBOA-Glc, resulting in TRIBOA-Glc (Fig. 1*A*). In maize, this reaction is catalyzed by a 2-ODD named BX6 (11). In *A. squarrosa,* we also identified a 2-ODD among the top 10 candidate genes highly expressed in young leaves and flowers. Expression of this gene, named *AsBx6*, with maize *ZmBx1-5* + *ZmBx7-8* in *N. benthamiana* leaves resulted in the formation of DIMBOA-Glc (Fig. 5*A*). Enzyme assays with purified AsBX6 heterologously expressed in *E. coli* showed that the enzyme accepted only DIBOA-Glc and not the corresponding aglycone DIBOA (Fig. 5*B*). Phylogenetic analysis of AsBX6, ZmBX6, and related 2-ODD-like proteins from other plants revealed that AsBX6 and ZmBX6 belong to two well-separated clades and only share 29.6% amino acid sequence identity (Fig. 5*C* and *SI Appendix*, Table S1*D*), indicating that ZmBX6 is more related to other proteins from eudicot species not known to produce BXDs rather than to AsBX6. Therefore, as with *Bx5*, it is likely that *Bx6* arose independently in the monocots and eudicots.

### Two PPOs from *A. squarrosa* Show Low BX6 Activity.

Two PPOs (AsPPO-1 and AsPPO-2) from *A. squarrosa* that appeared among the 25 top coexpressed gene candidates with high expression in roots, showed trace BX6 activity when expressed with maize *ZmBx1-5* + *ZmBx7-8* in *N. benthamiana* (*SI Appendix*, Fig. S8*A*). Since TRIBOA-Glc ionizes poorly in most mass spectrometry methods, we tested BX6 activity by expressing the enzyme together with the last pathway enzyme BX7 (see below) to observe DIMBOA-Glc, which can be detected in a more sensitive manner by mass spectrometry. The activity of AsPPO-1 and AsPPO-2 was low when compared to AsBX6, but clearly different when compared to the respective control (*ZmBx1-5* + *ZmBx7-8*) (*SI Appendix*, Fig. S8*A*). To test whether the low BX6 activity of AsPPO-1 and AsPPO-2 is a general feature of PPOs, we tested all other PPOs that we could retrieve from our transcriptomes of *A. squarrosa* and *L. galeobdolon*. However, none of the 11 additional PPOs tested, 5 from *A. squarrosa* and 6 from *L. galeobdolon*, showed BX6 activity when expressed with *ZmBx1-5* + *ZmBx7-8* in *N. benthamiana* (*SI Appendix*, Fig. S8 *A* and *B*). Signal peptide prediction suggested that AsPPO-1 and AsPPO-2 are localized to the plastid. Since plastidial localization is not consistent with the localization of the predicted cytosolic location of upstream (BX5) and downstream (BX7) enzymes, and since AsPPO-1 and AsPPO-2 had low BX6 activity compared with AsBX6, we hypothesize that these two PPOs are unlikely to be involved in BXD formation in planta.

### *A. squarrosa* AsBX7 Is Not Related to ZmBX7.

**Fig. 6.**
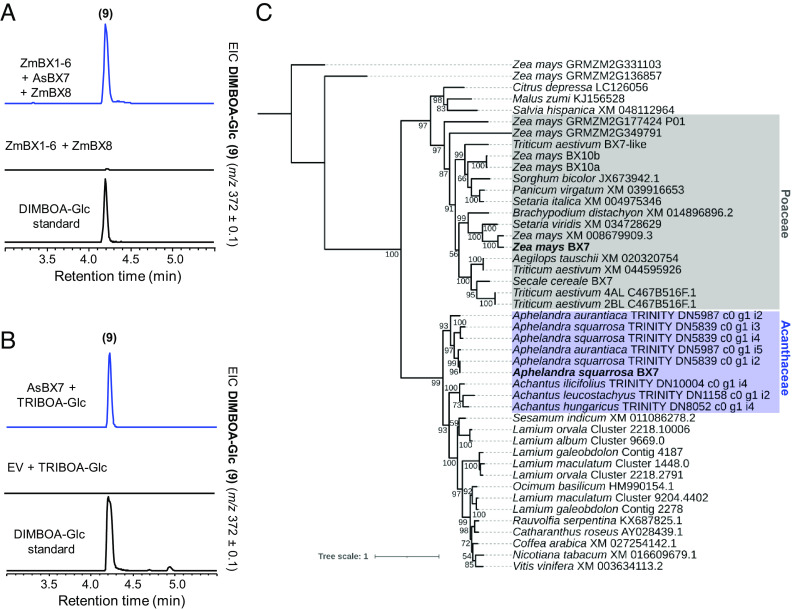
Characterization of AsBX7 in *N. benthamiana* and in vitro. (*A*) Coexpression of *AsBx7* with *ZmBx1-6 + ZmBx8* in *N. benthamiana* resulted in DIMBOA-Glc (9) accumulation. Methanol extracts of transiently transformed *N. benthamiana* leaves were analyzed using LC-qTOF. EIC, extracted ion chromatogram. (*B*) In vitro characterization of AsBX6. *AsBx6* was expressed in *E. coli* and His-purified protein was incubated with TRIBOA-Glc and the cosubstrate SAM. Enzyme products were analyzed using LC-qTOF. EV, empty vector control. (*C*) Maximum likelihood phylogenetic analysis of AsBX7, ZmBX7, and related OMTs from other monocot and eudicot plants.

The last step of the BXD pathway in *A. squarrosa* consists of *O*-methylation of TRIBOA-Glc to form DIMBOA-Glc (Fig. 1*A*). We identified an OMT, AsBX7, that showed BX7 activity both in *N. benthamiana* when transiently expressed with the upstream maize pathway genes *ZmBx1-6* + *ZmBx8* and in vitro when assayed with the putative substrate TRIBOA-Glc (Fig. 6 *A* and *B*). Phylogenetic analysis of AsBX7 and maize ZmBX7 showed that both proteins cluster in two well-distinct clades consisting of eudicot and Poaceae OMTs, respectively (Fig. 6*C*). Furthermore, AsBX7 and ZmBX7 only share 27.5% amino acid sequence identity (*SI Appendix*, Table S1*E*) indicating that the BX7 activity evolved independently in monocots and eudicots.

### Reconstitution of the BXD Pathways from *L. galeobdolon* and *A. squarrosa* in *N. benthamiana* Resulted in BXD Accumulation.

IGL, which catalyzes the formation of the starting substrate indole from indole 3-glycerol phosphate, had been previously identified and characterized from *L. galeobdolon* and *A. squarrosa* (28). To test whether the BXD enzymes identified in this study form functional pathways *in planta*, we expressed all pathway genes with *LgIGL1*, *LgIGL2*, and *AsIGL*, respectively, in *N. benthamiana*. While the reconstitution of the *L. galeobdolon* BXD pathway consisting of LgIGL1, LgFMO, LgBX4, LgBX5, and LgBX8 led to accumulation of DIBOA-Glc, only traces of this compound could be detected when LgIGL2 was used as BX1 (*SI Appendix*, Fig. S9*A*). This suggests that LgIGL1 but not LgIGL2 is involved in BXD formation in *L. galeobdolon*. Reconstitution of the *A. squarrosa* BXD pathway by expressing *AsIGL*, *AsFMO*, *AsBx4-7*, and *ZmBx8* in *N. benthamiana* also led to the accumulation of BXDs, primarily DIMBOA-Glc (*SI Appendix*, Fig. S9*B*). ZmBX8 was used to reconstitute the pathway since BX8 could not be identified in *A. squarrosa,* despite extensive screening.

### The BXD Pathway in the Acanthaceae Is Likely of Monophyletic Origin.

While *L. galeobdolon* is the only species in the Lamiaceae family known to produce BXDs, many Acanthaceae species have been described to produce these compounds (8, 28, 35, 36). To obtain insights into BXD evolution in the Acanthaceae, we searched for BXD genes in the publicly available transcriptome of *A. ilicifolius*, a member of this family. Interestingly, we could identify potential orthologs for *AsFMO*, *AsBx4*, and *AsBx5*, which had BX2/3, BX4, and BX5 activity, respectively, when expressed in *N. benthamiana* (Figs. 2*C* and 3*B* and *SI Appendix*, Figs. S5*B* and S6 *C* and *D*). The identified enzymes, AiFMO, AiBX4, and AiBX5, showed on average 80% amino acid sequence identity with their *A. squarrosa* orthologs (*SI Appendix*, Table S1). However, the *A. ilicifolius* transcriptome did not reveal apparent orthologs for *AsBx6* and *AsBx7,* the most similar genes had less than 60 % sequence identity, which is consistent with the fact that this species produces only DIBOA-Glc (35). Therefore, evolution of BXDs is likely of monophyletic origin within the Acanthaceae family.

## Discussion

We report here the elucidation of BXD biosynthesis in two eudicot species, *L. galeobdolon* and *A. squarrosa*, resolving a question that had remained unanswered for more than two decades since the initial discovery of BXDs in these species. Remarkably, our discoveries show that monocots and eudicots use different catalytic strategies to synthesize the same compounds. Our results suggest that the BXD pathway evolved multiple times in parallel not only in monocots and eudicots, but that even within eudicots at least single steps, if not the entire pathway, evolved independently.

One of the most unusual catalytic innovations made by the eudicots in BXD biosynthesis is the evolution of a bifunctional flavoenzyme. While in maize and other grasses the initial indole oxidations leading to 3HI2O are catalyzed by two P450 enzymes, eudicots instead recruited and evolved a single FMO to catalyze these two reactions (Fig. 2*A*). FMO and P450 enzymes can in principle catalyze similar oxidation reactions. For example, in cyanogenic glycoside biosynthesis in seed plants, the formation of aldoximes from amino acids is mediated by CYP79 enzymes (37–39), while in ferns, an FMO is responsible for this reaction (40). Also, oxidation of indoles can be catalyzed by FMO enzymes. *Persicaria tinctoria*, for example, uses an FMO (PtFMO) to oxidize indole to 3-hydroxy-indole (41). Though despite the similar biochemical activity, LgFMO and AsFMO share only approximately 50% sequence identity with PtFMO (Fig. 2*C*), and are most likely not of common origin with PtFMO.

In maize and other grasses, the P450s BX3, BX4, and BX5 all likely evolved by successive tandem gene duplications from a BX2 precursor (19). In contrast, both *L. galeobdolon* and *A. squarrosa* recruited a variety of P450s for the BXD pathway. In grasses, the oxidative ring expansion of 3HI2O to HBOA (BX4 activity) is catalyzed by a CYP71, while both *L. galeobdolon* and *A. squarrosa* possess a CYP92 that catalyzes this reaction (Fig. 3*A*). While it is clear that BX4 activity arose independently in the monocots and eudicots, the evolutionary origin of this enzyme activity within the eudicots cannot be answered unequivocally. The fact that CYP92 enzymes closely related to LgBX4 and AsBX4 from other *Lamium* species and from *M. vulgare* lack BX4 activity (Fig. 3 and *SI Appendix*, Fig. S5) suggests that LgBX4 and AsBX4 evolved their activity independently. However, the discovery of CYP92 enzymes with BX4 activity in *S. dulcis* and *W. religiosa*, which belong to the Plantaginaceae and Apocynaceae, respectively, could also be explained by a common ancestor with BX4 activity and the multiple loss of BX4 activity in non-BXD-producing species. Reconstruction and functional characterization of this potential BX4 ancestor could contribute to a better understanding of the evolution of BX4 activity in eudicots in future work. Notably, very few CYP92s have been assigned a biochemical function. The most closely related characterized enzyme to LgBX4 and AsBX4 is CYP92B14 from *S. indicum* (63% and 55% sequence identity with LgBX4 and AsBX4, respectively), which oxidizes the lignan substrate (+)-sesamin to (+)-sesamolin and (+)-sesaminol (42). These CYP92 enzymes therefore expand the substrate repertoire of a P450 family about which almost nothing is known. The number of P450 families involved in BXD biosynthesis in the eudicots increases further when we consider BX5. We showed that *A. squarrosa* possesses a CYP81 that catalyzes HBOA-N hydroxylation, whereas in *L. galeobdolon* the same reaction is carried out by a CYP71 (Fig. 4*A*). The recruitment of BX5 enzymes from different P450 families in *A. squarrosa* and *L. galeobdolon* as well as the phylogenetic placement of LgBX5 and ZmBX5 (Fig. 4*B*) suggest that BX5 activity evolved independently in the Poaceae, Acanthaceae, and Lamiaceae.

Glycosylation of BXDs is a prerequisite for storage in the vacuole. In principle, glycosylation can occur on any BXD intermediate containing a hydroxyl moiety (3HI2O, HBOA, DIBOA, TRIBOA, and DIMBOA, Fig. 1*A*). We identified a UGT in *L. galeobdolon* (LgBX8) that was able to glucosylate DIBOA and HBOA, but not 3HI2O in vitro (*SI Appendix*, Fig. S7 *A*–*D*). Although we performed extensive screening of UGTs from *A. squarrosa* (*SI Appendix*, Fig. S2*A*), we could not find a functional BX8 in this plant. However, characterization of the hydroxylase AsBX6 showed that the enzyme accepted only DIBOA-Glc and not free DIBOA as substrate (Fig. 5*B*), suggesting that BXD glucosylation in *A. squarrosa* also occurs on either HBOA or DIBOA, as in *L. galeobdolon* and maize. While LgBX8 belongs to the UGT85 family, maize ZmBX8 and ZmBX9 are members of the UGT710 family (34). This and the low sequence similarity between ZmBX8/9 and LgBX8 (*SI Appendix*, Fig. S7*E and* Table S1*F*) indicates independent evolution. Finally, in contrast to *L. galeobdolon*, the BXD pathway in *A. squarrosa* continues with hydroxylation and methylation of DIBOA-Glc to form the final product DIMBOA-Glc. As in maize and other grasses, the involved enzymes belong to the 2-ODD family (AsBX6) and OMT family (AsBX7), respectively, but phylogenetic analyses showed that also these last steps of the BXD pathway evolved independently in the Poaceae and Acanthaceae (Figs. 5*C* and 6*C*).

Several models have been proposed to explain the evolution of whole metabolic pathways (43). The “forward or cumulative hypothesis” and “retrograde hypothesis”, for example, describe a scenario in which the pathway either evolves in the forward direction, from the first acting biosynthetic enzyme onward, or alternatively, in the reverse direction, from the last acting enzyme of the pathway toward the first acting one, using in both cases tandem gene duplication and neofunctionalization as the major sources of innovation in the evolutionary process (44–46). In contrast, the “patchwork hypothesis” suggests that each biosynthetic activity has been recruited independently from preexisting enzymes that have inherent promiscuity toward chemically related substrates (47). Our results show that the repeated evolution of BXDs in angiosperms has followed different evolutionary trajectories. Given the inferred evolutionary history of the *Bx2-5* genes in the Poaceae (1, 19), the “forward” hypotheses is the model that best describes the evolution of the core BXD pathway in this plant family. In contrast, in eudicotyledons, the recruitment of different P450 families and different enzyme classes to BXD formation seem more consistent with the “patchwork hypothesis”. Furthermore, the identification of promiscuous enzymes with BX activities such as AsPPO-1/2 (*SI Appendix*, Fig. S8) and the various *Lamium* BX5-like enzymes (Fig. 4 *B* and *C*) suggests that the mechanisms underlying the “patchwork hypothesis” may represent a strong evolutionary force for BXD evolution in eudicots.

The recruitment of a 3HI2O-producing FMO in *L. galeobdolon* and *A. squarrosa* and the identification of related FMOs with BX2/BX3 activity in plants unable to produce BXDs (Fig. 2*C* and *SI Appendix*, Fig. S4*A*) is also consistent with the “patchwork hypothesis”. Here, the widespread presence of BX2/BX3 activity in eudicots, together with the fact that many of these plants can produce indole through the action of IGL (28, 48, 49), could explain the sporadic evolution of BXDs in distantly related families. In this scenario, changes in transcriptional regulation would be the switching event leading to BXD-committed IGLs and FMOs, and the concomitant or subsequent emergence of BX4 and BX5 activities then results in the formation of DIBOA, which could be glycosylated by a preexisting UGT with broad substrate specificity.

Another aspect that distinguishes BXD biosynthesis in the different plant families is their occurrence in each family. As in the Poaceae, BXDs are widespread in the Acanthaceae, whereas only one species is known to produce BXDs in each of the Lamiaceae, Ranunculaceae, and other families. Identification of *Bx* genes in several Poaceae species has shown that the BXD pathway in this family is monophyletic in origin (19, 20), and our results suggest the same for the Acanthaceae. *Acanthus*, a genus that belongs to the Acanthaceae, comprises several species that produce DIBOA-Glc (8, 35). By screening a transcriptome of *A. ilicifolius*, we identified genes that have an average nucleotide sequence identity of 80% with the *Bx* genes of *A. squarrosa* and encode active BX enzymes (Figs. 2*C* and 3*B* and *SI Appendix*, Figs. S4*A*, S5*B*, and S6 *C* and *D* and Table S1). This suggests that the occurrence of BXDs in the Acanthaceae, as in the Poaceae, preceded the radiation of this family and is monophyletic in origin.

In the Lamiaceae, our transcriptome data indicate that there are no *Bx* genes in species that cannot produce BXDs. Thus, the appearance of BXDs in the Lamiaceae and other families, of which only a single species is known to produce BXDs, is likely a recent evolutionary event compared to the Poaceae and Acanthaceae. However, without the genome sequences of the species in question, it is not possible to make a definitive statement. It could be that orthologous genes exist in various *Lamium* species but are not expressed here.

The independent evolution of identical enzyme activities can be specified by the terms “convergent evolution” and “parallel evolution”. According to the definition of Weng and Noel (50), “convergent evolution” is used when proteins without structural similarity, i.e., with different folds, have the same enzyme activity. In contrast, “parallel evolution” is used when proteins with the same function have evolved from precursors with different enzymatic activities but the same fold. Thus, the evolution of the BXD pathway in angiosperms is an example of parallel evolution except for the BX2/BX3 step. The recruitment of cytochrome P450s in the monocots and FMOs in eudicots, however, constitutes an example of convergent evolution. Although examples of parallel and convergent evolution in plant-specialized metabolism have been reported, they are often confined to pathways with a limited number of reactions or describe single enzymatic steps that lead to modification or decoration of core structures produced by conserved pathways (21–23, 51–53). By elucidating the BXD pathway in two eudicot species and comparing the identified enzymes with those already known from grasses, we could show that the entire pathway, consisting of up to 8 steps, evolved independently several times in the angiosperms. While the evolution of the core BXD pathway in the Poaceae is consistent with the “forward” hypotheses, BXD pathway evolution in the eudicots is more consistent with the “patchwork hypothesis”. Here, repeated recruitment of preexisting enzymes with activities that could be used in BXD biosynthesis could explain the sporadic distribution of BXDs in this lineage.

## Materials and Methods

Detailed materials and methods for all experiments are provided in *SI Appendix*, *Materials and Methods*.

### Plant Material and Growth.

*A. squarrosa* plants were grown on a 14-h-light/ 10-h-dark photoperiod with 40 to 70% humidity. *L. galeobdolon* plants were grown on a 16-h-light/ 8-h-dark photoperiod with 45 to 60% humidity. *Z. mays* cultivar “sweet nugget” plants used for BXD isolation were grown on a 14-h-light/ 10-h-dark photoperiod with 40 to 70% humidity. *N. benthamiana* plants used for transient gene expression were grown on a 16-h-light/8-h-dark photoperiod at 22 °C and 60% relative humidity. Plants were grown for 3 to 4 wk before infiltration of gene candidates. All plant were grown in a greenhouse.

### De Novo Transcriptome Assembly and Gene Candidate Identification.

Gene expression profiling in *A. squarrosa and Lamium sp*. tissues was obtained by RNA extraction. Three biological replicates of each tissue were prepared. Upon library preparation, samples were sequenced using Illumina sequencing. De novo trancriptome assemblies and read mapping were performed using OmixBox and CLC Genomic workbench. The average RPKM expression value per tissue was calculated for gene candidates belonging to enzymatic classes of interest. Transcripts with a maximum RPKM value lower than 10 were removed. Transcripts with LogFC < 1 between BXD-accumulating and nonaccumulating tissues/species were removed.

### *A. tumefaciens*-Mediated Transient Transformation of *N. benthamiana*.

Sequence-confirmed constructs of candidate genes were transformed in *A. tumefaciens* GV3101 through electroporation. Transformed cells were plated on LB media with appropriate selection and incubated 48 h at 28 °C. For *N. benthamiana* transient-transformation, overnight cultures started from single colonies were pelleted by centrifugation and resuspended in infiltration media (10 mM MES, 10 mM MgCl_2_, 100 µM acetosyringone, pH 5.7) to OD_600_ = 0.5 to 0.6 and incubated from 1.5 to 2.5 h at 28 °C, 200 rpm. Isovolumes of the prepared infiltration solutions were mixed to obtain the desired coinfiltration mixtures. The coinfiltration mixtures were infiltrated in 3 to 4-wk-old *N. benthamiana* leaves using a needless 1-mL syringe. The infiltrated leaves were harvested 5 d post infiltration. When exogenous substrate was used, 1 mL substrate dissolved in infiltration media (500 µM) was injected into transformed leaves 3 d after transformation with a needleless syringe.

### Heterologous Expression of Candidate Genes in *S. cerevisiae*, Microsome Preparation and In Vitro Assay.

*S. cerevisiae* WAT11 was transformed using the lithium acetate method. Positive *S. cerevisiae* WAT11 colonies were inoculated and cultured in SD-Leu medium (+2% Glucose) and protein expression was induced exchanging the media to SD-Leu (+2% Galactose). Pelletted cultures were disrupted using glass beads. The supernatant was then centrifuged at 100,000 × g, 4 °C, 90 min. The formed pellet was homogenized using a potter. An aliquot of microsomes (15 µL) was used for in vitro assays in KPO_4_ buffer (25 mM, pH 7.5) with 1 mM substrate and 3 mM NADPH in 100 µL total volume. The reactions were incubated at 30 °C, 300 rpm, for 2 h before quenching with an isovolume of methanol.

### Heterologous Expression of Candidate Genes in *E. coli* and In Vitro Assays.

Single colonies of *E. coli* DE3 transformed with sequence-confirmed plasmids were inoculated in liquid LB medium with selection. The seed culture was used to inoculate 2 x YT medium with selection and the culture was grown until OD_600_ = 0.6 to 0.8. Cultures were then incubated at 18 °C before addition of 500 µM IPTG. Induced cultures were incubated at 18 °C, 250 rpm, overnight. Cells harvested by centrifugation were disrupted by sonication on ice. Cell debris was removed by centrifugation and the N-terminal His-tagged proteins were purified from the supernatant using NiNTA agarose beads. In vitro assays were performed in KPO_4_ buffer (25 mM, pH 7.5) containing 1 mM substrate, 1 µg protein, and variable cofactors depending on the enzyme tested. BX6 (2-ODD): 500 µM FeSO_4_, 10 mM L-ascorbate, 10 mM 2-oxoglutarate, 3 mM DTT; BX7 (OMT): 1 mM SAM, 3 mM DTT; BX8 (UGT): 1.5 mM UDP-glucose, 100 µM MgCl_2_.

### LC-qTOF Mass Spectrometry Analysis.

Samples were analyzed as described in ref. 54 with minor variations. Briefly, compounds were separated by reverse-phase liquid chromatography using a Phenomenex Kinetex XB-C18 column (100 × 2.1 mm, 2.6 µm; 100 Å) at 35 °C. The mobile phases for metabolite separation consisted of water with 0.1% formic acid (A) and acetonitrile (B). A flow rate of 0.3 mL/min was used for the chromatography with an injection volume of 2 µL. The chromatographic separation was performed starting at 5% B for 1 min, linear gradient from 5 to 50% B in 7 min, 100% B for 2.5 min, 5% B for 2.5 min. Authentic standards were prepared as 20 µM solutions in Mass spectrometry acquisition was performed in positive or negative electrospray ionization mode depending on the compound of interest.

### Phylogenetic Analysis and Signal Peptide Prediction.

All sequences used for phylogenetic analysis were retrieved either from publicly available databases (NCBI, 1KP (55), Mint genome project (56), from in-house generated transcriptomes (*A. squarrosa* and *Lamium species*), or from publicly available transcriptomes downloaded from the European Nucleotide Archive (*Avicennia officinalis,* study accession PRJNA381534; *Aphelandra aurantiaca,* study accession PRJNA527030; *Acanthus leucostachyus* and *A. ilicifolius,* study accession PRJNA274850, *Acanthus hungaricus,* study accession PRJNA636634; *S. dulcis,* study accession PRJDB4273; *W. religiosa,* study accession PRJNA219429). Sequences are given in fasta format in *SI Appendix*, Data set 1.

Full-length amino acid sequences were aligned with webPRANK (https://www.ebi.ac.uk/goldman-srv/webprank/) (57). The resulting alignments were used to infer Maximul Likelihood phylogenies using IQ-TREE web server (http://iqtree.cibiv.univie.ac.at/) (58) with automatic substitution model and bootstrap value of 1,000. Trees were visualized and graphically modified using iTOL (https://itol.embl.de/personal_page.cgi). In silico localization analysis (signal peptide prediction) was performed using Predotar (https://urgi.versailles.inra.fr/Tools/Predotar).

## Supplementary Material

Appendix 01 (PDF)

## Data Availability

Raw reads from the transcriptome sequencing were deposited in the NCBI Sequence Read Archive (SRA) under the BioProject accession PRJNA967136 (59). Characterized genes were deposited in NCBI GenBank with the accession numbers given in *SI Appendix*, Table S3. All other data are included in the manuscript and/or *SI Appendix*.
