# Supplementary material for: Reinventing metabolic pathways: Independent evolution of benzoxazinoids in flowering plants
Source: Proc Natl Acad Sci U S A. 2023 Oct 9;120(42):e2307981120. doi: 10.1073/pnas.2307981120 (PMC10589660; doi:10.1073/pnas.2307981120)
Supplement: Supplementary file 1 — Appendix 01 (PDF) [file pnas.2307981120.sapp.pdf]

PNAS

Supporting Information for

Reinventing metabolic pathways: independent evolution of benzoxazinoids in flowering plants

Matilde Florean, Katrin Luck, Benke Hong, Yoko Nakamura, Sarah E. O'Connor, and Tobias G. Köllner

Correspondence: Tobias G. Köllner (koellner@ice.mpg.de) and Sarah E. O'Connor (oconnor@ice.mpg.de)

This PDF file includes:

Supporting text (Material and Methods)
Figures S1 to S10
Tables S1 to S3
Data set S1 (Sequences used for reconstructing phylogenetic trees)
Data set S2 (Supplementary NMR data)

SI Material and methods

Chemicals

All chemicals used in this study were purchased molecular biology grade or higher from Sigma Aldrich or Thermo Fisher unless otherwise stated.

Plant material and growth

Aphelandra squarrosa plants were grown in a greenhouse on a 14-h-light/ 10-h-dark photoperiod at 21 - 25°C during the day and 16 - 22°C during the night with 40 - 70 % humidity. *Lamium galeobdolon* plants were grown in a greenhouse on a 16-h-light/ 8-h-dark photoperiod at 20 - 24°C during the day and 16 - 20°C during the night with 45 - 60 % humidity. *Zea mays* cultivar "sweet nugget" plants used for BXD isolation were grown in a greenhouse on a 14-h-light/ 10-h-dark photoperiod at 21 - 24°C during the day and 19 - 22°C during the night with 40 - 70 % humidity. *Nicotiana benthamiana* plants used for transient gene expression were grown in a greenhouse on a 16-h-light/8-h-dark photoperiod at 22 °C and 60% relative humidity. Plants were grown for 3-4 weeks before infiltration of gene candidates.

Metabolite extraction and LC-MS metabolic analysis

Plant material was frozen in liquid nitrogen and ground to fine-powder in pre-chilled mortars or with 3 mm Tungsten Carbide Beads using a TissueLyser II (Quiagen). Tissue samples (100 mg ± 5%) were extracted with 500 µL methanol (LC-MS grade). Samples were vigorously vortexed and then incubated at 25°C with shaking for 15 min, followed by 15 min centrifugation at maximum speed using a table top centrifuge. Samples were then filtered with a 0.22 µm PTFE syringe filter before dilution for LC-MS analysis.

RNA-Seq and gene candidate identification

Total RNA from *A. squarrosa* and *Lamium sp.* tissues was extracted using RNeasy Mini Kit (Quiagen) according to manufacturer's instructions. RNase-Free DNase Set (Quiagen) was used to perform on-column genomic DNA digestion. RNA quality was assessed using a Nanophotometer N60 (Implen) as well as with a Bioanalyzer 2100 (BioRad). Three biological replicates of each tissue were prepared. Samples were submitted to Novogene for total RNA sequencing using the company's standard protocol for library preparation and Illumina RNA-sequencing. Approximately ≥ 40 million paired-end reads were acquired per sample. Reads were trimmed using the program OmicsBox (Biobam) and then assembled using the Trinity algorithm implemented in OmixBox and the CLC Genomics workbench (Qiagen)-integrated de novo assembly algorithm (bubble size, 800; minimum contig length, 500; mismatch cost, 2; insertion cost, 3; deletion cost, 3; length fraction, 0.8; similarity fraction, 0.9). The completeness of the generated *de novo* transcriptome assemblies was assessed by calculating the BUSCO value and the assembly used only if the value was ≥ 90%. The assembled transcriptomes were annotated using SwissProt 2021 database (blasting parameters: E-Value, 1.0E-3; number of Blast Hits, 20; word size, 3; low complexity filter, on; number of threads, 40; HSP length cutoff, 33). Reads were mapped on the annotated transcriptomes using CLC Genomics workbench (mapping parameters: mismatch cost, 2; insertion cost, 3; deletion cost, 3; length fraction, 0.8; similarity fraction 0.9; auto-detect paired distances, on; maximum number of hits for a read, 10). Gene candidate selection was performed by filtering the transcripts for enzymatic classes of interest and calculating the average RPKM expression value per tissue. Transcripts with a maximum RPKM value lower than 10 were removed. For the remaining transcripts, log fold change (logFC) between BXD-accumulating and non-accumulating tissues / species was calculated and the transcripts with LogFC < 1 were removed. Transcripts were then sorted from highest to lowest RPKM values.

Cloning

cDNA was prepared from total RNA with SuperScript IV VILO Master Mix (ThermoFisher Scientific) according to the manufacturer's instructions. Candidate genes were amplified from cDNA using Platinum SuperFi II PCR Master Mix (ThermoFisher Scientific). In the case of synthetic gene sequences obtained from Twist Biosciences, the synthesized sequences were used as template for PCR. PCR products were purified using DNA Clean and Concentrator-5 (Zymo). Amplified genes were inserted with In-Phusion HD

Cloning (Takara Bio) in the vectors p3Ω1 (*Bsa*I-HF digested), pOPINF (*Hind*III-HF/*Kpn*I-HF digested), or pESC-Leu (*Not*I-HF/*Pac*I-HF digested). *Escherichia coli* Top10 was transformed using the heat shock method and plated on LB agar-plates with appropriate selection. Overnight colonies were inoculated in liquid LB with appropriate selection and incubated at 37°C with 250 rpm. Plasmid DNA was isolated with Wizard Plus SV Minipreps DNA Purification System kit (Promega) and the inserted gene was fully sequenced. Primer sequences are given in SI Table 2.

A. tumefaciens-mediated transient transformation of _Nicotiana benthamiana_

Electrocompetent cells of *A. tumefaciens* GV3101 (Goldbio) were thawed on ice, mixed with 100 ng of sequence-confirmed plasmid, and incubated 30 min on ice. The cells were transferred into an electroporation cuvette (path, 1 cm) and electroporated using a Micropulser (BioRad). Transformed cells were recovered in 1 mL LB media and incubated for 3 h at 28°C, with 200 rpm shaking prior to plating on LB-agar plates with appropriate selection. Plates were incubated at 28°C for 48 h. Single colonies were inoculated in liquid LB with appropriate selection and incubated at 28°C, 200 rpm shaking over-night. For *N. benthamiana* transient-transformation, overnight cultures were pelleted by centrifugation at 4000 rpm for 10 min (14°C). The pellet was resuspended in infiltration media (10 mM MES, 10 mM $MgCl_2$, 100 µM acetosyringone, pH 5.7) to OD_{600}= 0.5-0.6 and incubated from 1.5 to 2.5 h at 28°C, 200 rpm. Isovolumes of the prepared infiltration solutions were mixed to obtain the desired co-infiltration mixtures. The co-infiltration mixtures were infiltrated in the abaxial side of 3-4 week old *N. benthamiana* leaves using a needless 1 mL syringe. Infiltrated plants were kept in the dark overnight and then transferred to a growth chamber and placed under growth lights. The infiltrated leaves were harvested 5 days post infiltration. In all transformations, a construct encoding the silencing repressor protein p19 was co-infiltrated to increase expression. When exogenous substrate was used, 1 mL of substrate dissolved in infiltration media (500 µM) was injected into transformed leaves three days after transformation with a needleless syringe.

Heterologous expression of candidate genes in _S. cerevisiae_

Saccharomyces cerevisiae WAT11 was made competent using the lithium acetate method (1). In brief, *S. cerevisiae* WAT11 was streaked to single colony on YPAD plates. A 10 mL YPAD seed culture inoculated with a single colony was grown overnight at 30°C, with shaking at 180 rpm. The seed culture was then topped with 40 mL YPAD and cultivated for 4-5 additional hours. After centrifugation (3000 x *g*, 4°C, 5 min), the pellet was washed with 25 mL ddH_2O and then again with 1 mL of ddH_2O. Cells were resuspended in 1 mL ddH_2O and 100 µL aliquots of the resuspended cells were transferred to a microcentrifuge tube and centrifuged at maximum speed for 1 min. The cell pellet was resuspended in the transformation mixture (240 µL PEG 3350 (50% w/v), 36 µL LiOAc (1 M), 100 µg salmon testis DNA, 1 µg plasmid DNA), incubated at 42°C for 50 min, centrifuged (6000 x *g*, room temperature, 1 min), resuspended again in 300 mL ddH_2O, and plated on SD-Leu agar plates containing 2% glucose. The plates were incubated at 30°C for 48-72 h. Single colonies were dissolved in 20 µL NaOH (20 mM) and boiled 10 min in a heat block. Two µL of the boiled colonies were added to a 15 µL PCR reaction with backbone-annealing primers to check for transformation.

Microsome preparation and microsome _in vitro_ assay

Positive *S. cerevisiae* WAT11 colonies were inoculated in 30 mL SD-Leu medium (+ 2% Glucose) and incubated overnight at 30°C, 180 rpm. An aliquot of cell culture corresponding to OD_{600}=1 was added to 100 mL SD-Leu medium (+ 2% Glucose) and incubated for 30-35 h at 30°C, with shaking at 180 rpm. Cells were pelleted (5000 x *g*, 16°C, 5 min), the supernatant removed, and the pellet resuspended in 100 mL SD-Leu (+ 2% Galactose). Cultures were then incubated for 15-18 h at 30°C, with shaking at180 rpm. Cells were harvested by centrifugation (4000 x *g*, 4°C, 10 min), resuspended in 30 mL TEK buffer (50 mM Tris-HCl, 1 mM EDTA, 100 mM KCl, pH 7.5), then in 2 mL TES buffer (50 mM Tris-HCl, 1 mM EDTA, 600 mM sorbitol, 10g/l bovine serum albumin, 1.5 mM β-mercaptoethanol/100 mL, pH 7.5). Glass beads (500 µm diameter) were added and the cultures were manually shaken 5 x 1 min interspaced by 1 min cooling on ice. The beads were washed 3 times with 5 mL TES buffer and the supernatant fractions were pooled together and centrifuged (7500 x *g*, 4°C, 10 min). The supernatant was then centrifuged at 100,000 x *g*,

4°C, 90 min. The formed pellet was washed first with 2.5 mL TES buffer, then with 2.5 mL TEG buffer (50 mM Tris-HCl, 1 mM EDTA, 30% glycerol, pH 7.5), and ultimately with 1 mL TEG buffer. The solution was homogenized using a glass potter. Microsomes were stored at -20°C. An aliquot of microsomes (15 µL) were used for *in vitro* assays in KPO_4 buffer (25 mM, pH 7.5) with 1 mM substrate and 3 mM NADPH in 100 µL total volume. The reactions were incubated at 30°C, 300 rpm, for 2 h before quenching with an isovolume of methanol. Mixtures were incubated for 30 min on ice, centrifuged at maximum speed for 20 min, and filtered through a 0.22 µm PTFE syringe filter before LC-MS analysis.

Heterologous expression of candidate genes in *Escherichia coli* and *in vitro* assays

E. coli DE3 (ThermoFisher Scientific) cultures were transformed with sequence-confirmed plasmids using the heat-shock method, plated on LB-agar plates with appropriate selection and grown at 37°C overnight. Single colonies were inoculated in liquid LB medium with selection and grown at 37°C, 250 rpm, overnight. The seed culture (100 µL) was used to inoculate 100 mL 2 x YT medium with selection and the culture was grown at 37°C, 250 rpm shaking, until OD_{600} = 0.6-0.8. Cultures were then incubated at 18°C, 250 rpm, for 20 min before addition of 500 µM IPTG. Induced cultures were incubated at 18°C, 250 rpm, overnight. The cells were harvested by centrifugation (4000 x *g*, 4°C, 10 min), resuspended in 10 mL A1 buffer (50 mM TRIS-HCl, 50 mM glycine, 5% v/v glycerol, 0.5 M NaCl, 20 mM imidazole, pH 8) with 0.2 g / L lysozyme and 1 tablet / 50 mL buffer of EDTA-free protease inhibitor, and disrupted by sonication for 2 min (2 s on, 3 s off) on ice (Bandelin UW 2070). Cell debris was removed by centrifugation at 35,000 × *g* at 4°C for 20 min and the N-terminal His-tagged proteins were purified from the supernatant using NiNTA agarose (Qiagen) beads according to the manufacturer's instructions. Proteins were eluted using elution Buffer (A1 buffer + 500 mM imidazole, pH 8). Ultimately, elution buffer was exchanged for protein storage buffer (20 mM HEPES, 150 mM NaCl, pH 7.5) using Amicon 10 KDa concentrator columns (Merck Millipore). Proteins were stored at –20°C. In vitro assays were performed in KPO_4 buffer (25 mM, pH 7.5) containing 1 mM substrate, 1 µg protein, and variable cofactors depending on the enzyme tested. BX6 (2-ODD): 500 µM $FeSO_4$, 10 mM L-ascorbate, 10 mM 2-oxoglutarate, 3 mM DTT; BX7 (OMT): 1 mM SAM, 3 mM DTT; BX8 (UGT): 1.5 mM UDP-glucose, 100 µM $MgCl_2$.

Liquid chromatography-quadrupole time-of-flight mass spectrometry (LC-qTOF) analysis

Samples were analyzed as described in (2, 3) with minor variations. Liquid chromatography-quadrupole time-of-flight mass spectrometry (LC-qTOF) analysis were performed using a Thermo Scientific UltiMate 3000 ultra-high performance liquid chromatography (UHPLC) system coupled to an Impact II UHR-Q-ToF (Ultra-High Resolution QuadrupoleTime-of-Flight) mass spectrometer (Bruker Daltonics). Compounds were separated by reverse-phase liquid chromatography using a Phenomenex Kinetex XB-C18 column (100 x 2.1 mm, 2.6 µm; 100 Å) at 35 °C. The mobile phases for metabolite separation consisted of water with 0.1% formic acid (A) and acetonitrile (B). A flow rate of 0.3 mL/min was used for the chromatography with an injection volume of 2 µL. The chromatographic separation was performed starting at 5% B for 1 min, linear gradient from 5% to 50% B in 7 min, 100% B for 2.5 min, 5% B for 2.5 min. Authentic standards were prepared as 20 µM solutions in methanol and 2 µL aliquots were injected under the chromatographic conditions described above. Mass spectrometry acquisition was performed in positive or negative electrospray ionization mode depending on the compound of interest. For positive ionization mode, a capillary voltage of 3500 V and an end plate offset of 500 V were used; a nebulizer pressure of 2.8 bar was used, with nitrogen at 280 °C and a flow of 8 l/min as the drying gas. Acquisition was performed at 12 Hz in the mass range from *m/z* 100 to 1000, with data dependent MS2 and an active exclusion window of 1 min. For collision energy, the stepping option model (from 20 to 50 eV) was used. For negative ionization mode, a capillary voltage of 3500 V and an end plate offset of 500 V were used; a nebulizer pressure of 2.0 bar was used, with nitrogen at 250 °C and a flow of 10 l/min as the drying gas. Acquisition was performed at 12 Hz in the mass range from *m/z* 80 to 1000, with data dependent MS2 and an active exclusion window of 1 min. In both modes, for collision energy, the stepping option model (from 20 to 50 eV) was used. At the beginning of each sample run, a sodium formate-isopropanol calibration solution was directly injected in the source at 0.18 mL/hour using a syringe pump to calibrate MS spectra recorded. To avoid injection peak

and salt contamination of the MS, the initial 1 min of the active chromatographic gradient of each run was discarded to waste.

Liquid chromatography-tandem mass spectrometry (LC-MS/MS) analysis

Targeted analysis of BXDs was performed as previously described by (4) using an Agilent 1260 Infinity II LC system (Agilent Tecnologies) equipped with a ZORBAX Eclipse XDB-C18 column (50 x 4.6 mm, 1.8 µm; Agilent Technologies) coupled with a Sciex QTRAP 6500+ tandem mass spectrometer. Chromatographic conditions consisted of water with 0.05% formic acid (A) and acetonitrile (B) at a flow rate of 1.1 mL / min with a column temperature of 20°C and injection volume 2 µL. Chromatographic conditions consisted of: 0 – 0.5 min, 5% B; 0.5 – 6 min, 5 – 32.5% B; 6.02 – 7.0 min, 100% B; 7.10 – 9.5 min, 5% B. The mass spectrometer, equipped with a Turbospray ESI source, was operated in negative ionization mode. Multiple reaction monitoring was used to monitor analyte precursor ion – product ion transitions.

Synthesis of 3HI2O and HBOA

3HI2O was synthesized according to the method described in (5). In brief, sodium borohydride (385 mg, 10.2 mmol) was added in small portions to a stirred suspension of isatin (1 g, 6.8 mmol) in 40 mL of a 1:1 dichloromethane/ethanol mixture at 0 °C. The mixture was stirred at 0 °C until the suspension became colorless (about 30 min). Then water (20 mL) was added and the reaction mixture was stirred until bubbling stopped. The mixture was extracted with dichloromethane (3 x 20 mL), the combined organic extracts were dried ($MgSO_4$), and the solvent evaporated under reduced pressure to yield 3HI2O (410 mg, 41%). HBOA was synthesized according to the method reported in (6). A solution of DIBALH in toluene (1 M, 2.46 mL, 2.46 mmol) was added within 5 min to a solution of 2,3-dioxo-1,4-benzoxazine (7) (200 mg, 1.23 mmol) in anhydrous toluene (5 mL) at –78 ^oC. The mixture was stirred for 2 h allowing it slowly to warm to room temperature. Then, the reaction was hydrolyzed by water (4 mL) at 0 ^oC. The mixture was acidified by 12 N HCl to pH 1. The aqueous layer was extracted with EtOAc (3 x 4 mL). The extracts were combined, dried with Na_2SO_4, and the solvents removed *in vacuo* to afford HBOA (126 mg, 63%). NMR spectra for synthesized 3HI2O and HBOA were measured on a 400 MHz Bruker Avance III HD spectrometer (Bruker Biospin GmbH, Rheinstetten, Germany). CD_3OD and $CDCl_3$ were used as solvents. NMR spectra were referenced to the residual solvent signals at δ_H 3.31 and δ_C 49.0 for CD_3OD and δ_H 7.26 and δ_C 77.0 for $CDCl_3$. For spectrometer control and data processing, Bruker TopSpin ver. 3.6.1 was used. The spectroscopic data for 3HI2O and HBOA are in accordance with the values reported in the literature (5, 8). NMR data for 3HI2O and HBOA are given in SI data set 2.

Isolation of HBOA-Glc, DIBOA-Glc, TRIBOA-Glc, and DIMBOA-Glc

HBOA-Glc was enzymatically synthesized by incubating LgBX8 with synthetically prepared HBOA, using the same reaction conditions described above for the *in vitro* assay. Ninety small scale reactions were combined and concentrated on a rotavapor prior to purification using preparative HPLC. DIBOA-Glc was isolated from 25 g of *L. galeobdolon* leaves. Leaves frozen in liquid nitrogen were ground in pre-cooled mortars and then extracted with three volumes of methanol, incubated at 25°C for 15 min, and then centrifuged at 4000 x *g* for 10 min. The methanol fraction was dried in a rotavapor and then re-extracted with 60% methanol in water. This fraction was purified on a SPE column (Oasis PRIME HLB 6cc- 500mg-Extraction Cartridge, Waters). The SPE-purified fraction was diluted 1:2 with methanol (60%) prior to preparative HPLC injection. TRIBOA-Glc was obtained enzymatically by using AsBX6 as described for the in vitro enzymatic assays by combining 50 small scale reactions. Reactions were combined, concentrated on a rotavapor, and injected on a preparative HPLC. DIMBOA-Glc was isolated from *Zea mays* 'Sweet Nugget' two weeks-old seedlings as described for DIBOA-Glc isolation. Preparative-scale chromatographic separation was performed using an Agilent 1260 Infinity II preparative HPLC system coupled to a multiple wavelength detector and fraction collector. A Phenomenex Kinetex XB-C18 column (250 x 10 mm, 5 µm, 100 A) was used. The mobile phases used for separation were A (H_2O + 0.1% formic acid) and B (acetonitrile). The flow rate was set at 6.0 mL/min with a gradient of B from 5% to 21% in 21 min, 100% B for 5 min, 5% B for 5 min. Manual injections (300 µL per injection) were performed, and the separation was monitored at 254 nm and 280 nm simultaneously.

NMR analysis of enzymatic products and isolated compounds

NMR measurements of enzymatically produced compounds were carried out on a 700 MHz Bruker Avance III HD spectrometer (Bruker Biospin GmbH, Rheinstetten, Germany), equipped with a TCI cryoprobe using standard pulse sequences as implemented in Bruker Topspin ver. 3.6.1. (Bruker Biospin GmbH, Rheinstetten, Germany). Chemical shifts were referenced to the residual solvent signals of methanol-d_3 (δ_H 3.31/δ_C 49.0) and acetone-d_6 (δ_H 2.05/δ_C 29.84), respectively. All NMR data are given in SI data set 2.

ECD measurement and data comparison

ECD spectra of HBOA-Glc, DIBOA-Glc, TRIBOA-Glc, and DIMBOA-Glc were measured at 25°C on a JASCO J-810 spectropolarimeter (JASCO cooperation, Tokyo, Japan) using a 350 µL cell. Instrument control and data processing was accomplished using JASCO Spectra Manager II. Experimentally measured ECD data and calculated data were compared using SpecDis ver.1.71 (https://specdis-software.jimdo.com) and indicates *R* configuration for all compounds tested (SI Fig. 10). In addition, *R* configuration of DIBOA-Glc was further proved by measuring the specific optical rotation on a Jasco P-1030 polarimeter.

Supplementary material and methods references

1. R. D. Gietz, R. A. Woods, Yeast Transformation by the LiAc/SS Carrier DNA/PEG Method. *Methods Mol Biology Clifton N J* 313, 107–120 (2005).

2. M. O. Kamileen, *et al.*, Recycling Upstream Redox Enzymes Expands the Regioselectivity of Cycloaddition in Pseudo-Aspidosperma Alkaloid Biosynthesis. *J. Am. Chem. Soc.* 144, 19673–19679 (2022).

3. C. Schotte, *et al.*, Directed Biosynthesis of Mitragynine Stereoisomers. *J. Am. Chem. Soc.* 145, 4957–4963 (2023).

4. C. Förster, *et al.*, Biosynthesis and antifungal activity of fungus-induced O-methylated flavonoids in maize. *Plant Physiol* 188, kiab496 (2021).

5. M. Balha, S. C. Pan, Organocatalytic Asymmetric Synthesis of Bridged Acetals with Spirooxindole Skeleton. *J Org Chem* 83, 14703–14712 (2018).

6. D. Sicker*, H. Hartenstein, A New General Approach to the 2-Hydroxy-2H-1,4-benzoxazin-3(4H)-one Skeleton via Diisobutylaluminum Hydride Reduction of 2,3-Dioxo-1,4-benzoxazines. *Synthesis* 1993, 771–772 (1993).

7. D. Xie, *et al.*, Quinoxaline-2,3-diones: potential d-amino acid oxidase (DAAO) inhibitors. *Med Chem Res* 23, 4977–4989 (2014).

8. F. A. Macías, *et al.*, Isolation and Synthesis of Allelochemicals from Gramineae: Benzoxazinones and Related Compounds. *J Agr Food Chem* 54, 991–1000 (2006).

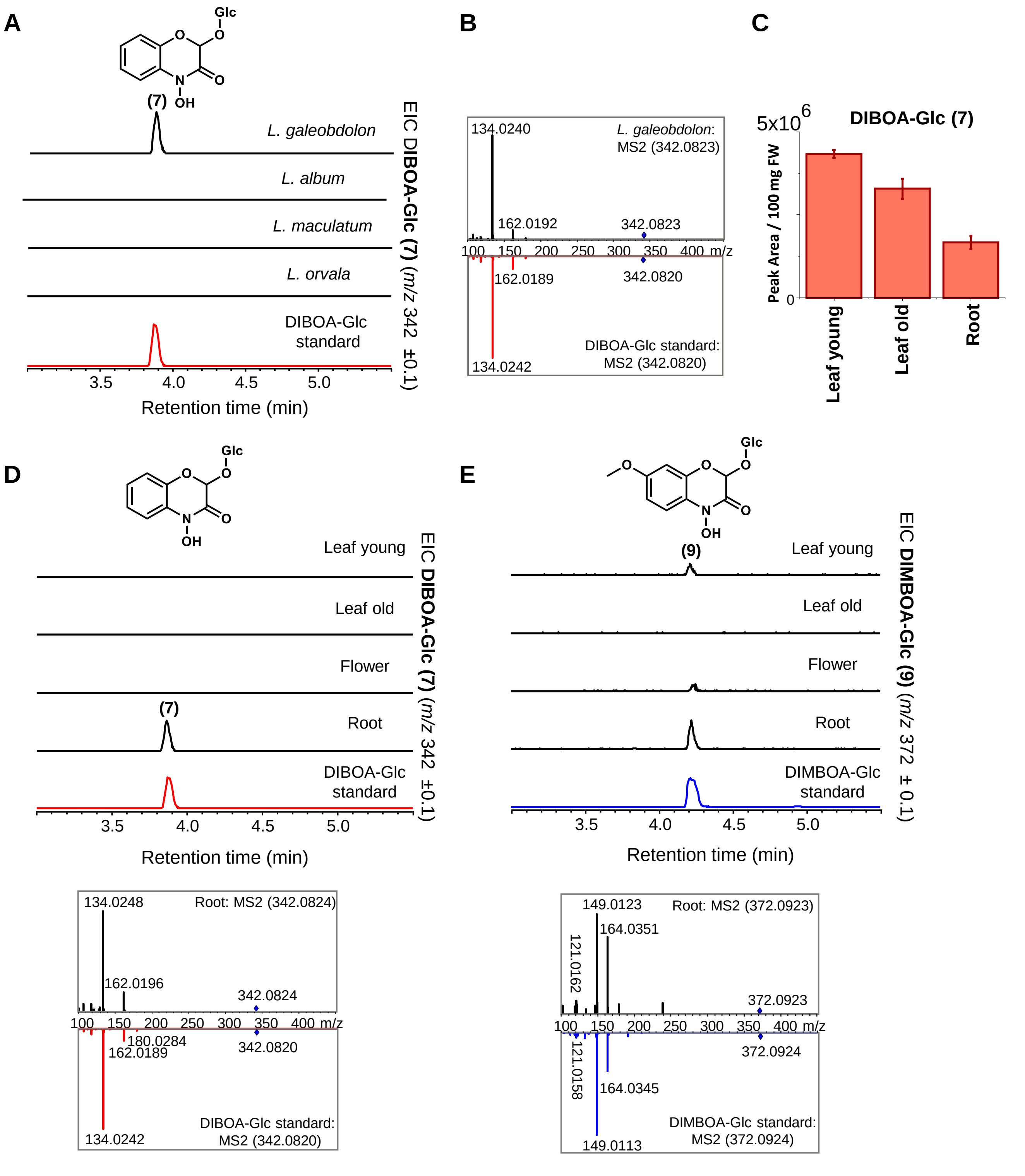

SI Figure 1: Metabolic profiling of *A. squarrosa* and *L. galeobdolon*. A) LC-qTOF analysis of *Lamium sp.* methanol leaf extracts. DIBOA-Glc presence was confirmed with an authentic standard. B) MS2 fragmentation spectra of DIBOA-Glc in *L. galeobdolon* matches the MS2 fragmentation spectra of the reference standard. C) Tissue distribution of DIBOA-Glc accumulation in *L. galeobdolon*. Means ± SE (n = 3 biological replicates) are shown. D and E) DIBOA-Glc and DIMBOA-Glc content in *A. squarrosa* organs. Plant material was extracted with methanol and analyzed using LC-qTOF. MS2 fragmentation spectra of DIBOA-Glc and DIMBOA-Glc matched those of the corresponding authentic standards.

A

Root	Young leaf	Flower	Fully developed leaf	Gene	Annotation	LogFC
1.0	0.1	0.4	0.2	TRINITY_DN4480_c0_g1_i1	Cytochrome P450 CYP75	2.45
0.8	1.0	0.2	0.1	TRINITY_DN6556_c0_g1_i1	Flavin-containing monooxygenase	3.28
0.7	1.0	0.1	0.1	TRINITY_DN5839_c0_g1_i1	O-methyltransferase	3.91
0.1	1.0	0.8	0.0	TRINITY_DN13400_c0_g1_i1	2-oxoglutarate dependent dioxygenase	6.33
1.0	0.3	0.3	0.0	TRINITY_DN1230_c0_g1_i1	Polyphenol oxidase	6.53
0.4	0.1	1.0	0.3	TRINITY_DN5296_c0_g1_i1	Cytochrome P450 CYP94	1.81
1.0	0.1	0.2	0.0	TRINITY_DN2569_c0_g1_i1	Cytochrome P450 CYP81	21.18
1.0	0.0	0.0	0.0	TRINITY_DN12675_c0_g1_i1	Cytochrome P450 CYP71	9.30
0.4	1.0	0.5	0.4	TRINITY_DN5057_c0_g1_i2	Cytochrome P450 CYP98	1.31
1.0	0.0	0.0	0.0	TRINITY_DN5547_c0_g1_i2	Cytochrome P450 CYP71	7.66
1.0	0.4	0.2	0.1	TRINITY_DN15617_c0_g1_i1	Polyphenol oxidase	2.76
1.0	0.1	0.7	0.0	TRINITY_DN2569_c0_g1_i2	Cytochrome P450 CYP81	10.89
0.8	1.0	0.8	0.4	TRINITY_DN6106_c0_g1_i1	O-methyltransferase	1.45
1.0	0.0	0.0	0.0	TRINITY_DN1798_c0_g1_i1	O-methyltransferase	20.32
1.0	0.3	0.3	0.3	TRINITY_DN3039_c0_g1_i1	UDP-glucose glucosyltransferase	1.68
1.0	0.0	0.0	0.0	TRINITY_DN11257_c0_g1_i2	Cytochrome P450 CYP71	4.48
1.0	0.3	0.2	0.5	TRINITY_DN1237_c0_g1_i2	UDP-glucose glucosyltransferase	1.12
0.2	1.0	0.5	0.4	TRINITY_DN2282_c0_g1_i2	Cytochrome P450 CYP97	1.39
0.5	1.0	0.8	0.1	TRINITY_DN2711_c0_g1_i2	Cytochrome P450 CYP51	2.75
1.0	0.0	0.1	0.0	TRINITY_DN4762_c0_g1_i1	Polyphenol oxidase	7.61
1.0	0.6	0.0	0.2	TRINITY_DN11613_c0_g2_i1	Cytochrome P450 CYP78	2.69
1.0	0.1	0.2	0.3	TRINITY_DN2021_c0_g1_i4	Cytochrome P450 CYP72	1.62
1.0	0.2	0.5	0.0	TRINITY_DN13074_c0_g1_i1	Cytochrome P450 CYP72	6.15
1.0	0.6	0.5	0.5	TRINITY_DN2396_c1_g2_i2	UDP-glucose glucosyltransferase	1.02
1.0	0.0	0.0	0.0	TRINITY_DN4025_c0_g1_i2	Cytochrome P450 CYP71	7.91
1.0	0.0	0.5	0.1	TRINITY_DN9900_c0_g1_i2	Cytochrome P450 CYP749	3.04
1.0	0.0	0.0	0.4	TRINITY_DN2173_c0_g1_i3	Cytochrome P450 CYP76	1.25
1.0	0.3	0.2	0.0	TRINITY_DN238_c0_g1_i2	O-methyltransferase	7.58
1.0	0.0	0.0	0.0	TRINITY_DN121516_c0_g1_i1	Dioxygenase	10.98
1.0	0.4	0.6	0.0	TRINITY_DN13074_c0_g1_i2	O-methyltransferase	4.61
1.0	0.3	0.4	0.3	TRINITY_DN13693_c0_g1_i1	2-oxoglutarate dependent dioxygenase	1.56
1.0	0.5	0.5	0.5	TRINITY_DN1155_c0_g2_i3	Cytochrome P450 CYP72	1.12
1.0	0.0	0.0	0.0	TRINITY_DN12179_c0_g1_i3	Cytochrome P450 CYP76	7.13
1.0	0.3	0.2	0.4	TRINITY_DN1237_c0_g1_i1	UDP-glucose glucosyltransferase	1.41
1.0	0.0	0.0	0.0	TRINITY_DN9958_c0_g1_i1	O-methyltransferase	18.48
1.0	0.2	0.0	0.0	TRINITY_DN15617_c1_g1_i3	Polyphenol oxidase	18.47
1.0	0.0	0.1	0.0	TRINITY_DN7424_c0_g1_i3	Cytochrome P450 CYP71	18.45
1.0	0.0	0.0	0.0	TRINITY_DN14114_c0_g2_i3	2-oxoglutarate dependent dioxygenase	7.81
1.0	0.1	0.1	0.1	TRINITY_DN6380_c0_g1_i11	Cytochrome P450 CYP76	3.60
1.0	0.0	0.0	0.1	TRINITY_DN14457_c0_g1_i3	Cytochrome P450 CYP71	4.05
1.0	0.0	0.0	0.0	TRINITY_DN60557_c0_g1_i1	2-oxoglutarate dependent dioxygenase	9.37
1.0	0.3	0.0	0.0	TRINITY_DN10769_c0_g2_i1	Cytochrome P450 CYP87	8.43
1.0	0.0	0.0	0.0	TRINITY_DN10533_c0_g1_i3	2-oxoglutarate dependent dioxygenase	18.01
1.0	0.0	0.0	0.0	TRINITY_DN2404_c0_g2_i1	Cytochrome P450 CYP749	17.93
1.0	0.0	0.2	0.0	TRINITY_DN4762_c0_g1_i3	Polyphenol oxidase	6.33
1.0	0.1	0.0	0.0	TRINITY_DN9958_c0_g1_i4	O-methyltransferase	7.99
1.0	0.0	0.0	0.0	TRINITY_DN1798_c0_g2_i4	O-methyltransferase	5.99
1.0	0.0	0.0	0.0	TRINITY_DN238_c0_g2_i2	O-methyltransferase	17.88
1.0	0.0	0.6	0.0	TRINITY_DN13254_c0_g1_i1	Cytochrome P450 CYP76	5.63
1.0	0.2	0.2	0.2	TRINITY_DN801_c0_g1_i13	Cytochrome P450 CYP749	2.49
1.0	0.2	0.2	0.0	TRINITY_DN10074_c0_g1_i1	Cytochrome P450 CYP71	4.69
1.0	0.0	0.0	0.0	TRINITY_DN11835_c0_g1_i1	Cytochrome P450 CYP86	6.03
1.0	0.4	0.2	0.1	TRINITY_DN311_c0_g1_i4	2-oxoglutarate dependent dioxygenase	3.28
1.0	0.0	0.0	0.0	TRINITY_DN41106_c0_g1_i1	2-oxoglutarate dependent dioxygenase	17.76
1.0	0.3	0.2	0.4	TRINITY_DN4825_c0_g1_i2	Cytochrome P450 CYP71	1.32
1.0	0.2	0.2	0.3	TRINITY_DN801_c0_g1_i14	Cytochrome P450 CYP749	1.99
1.0	0.7	0.1	0.0	TRINITY_DN8686_c0_g1_i1	O-methyltransferase	10.08
1.0	0.1	0.0	0.0	TRINITY_DN5839_c1_g1_i1	O-methyltransferase	8.26
1.0	0.0	0.0	0.0	TRINITY_DN151_c1_g1_i1	Cytochrome P450 CYP716	17.65
1.0	0.4	0.1	0.2	TRINITY_DN21159_c0_g1_i1	O-methyltransferase	2.15
1.0	0.0	0.0	0.0	TRINITY_DN19747_c0_g1_i1	2-oxoglutarate dependent dioxygenase	7.73
1.0	0.1	0.0	0.0	TRINITY_DN5839_c0_g2_i1	O-methyltransferase	17.54
1.0	0.3	0.4	0.4	TRINITY_DN9349_c0_g1_i1	UDP-glucose glucosyltransferase	1.36
1.0	0.0	0.0	0.0	TRINITY_DN13750_c0_g1_i1	2-oxoglutarate dependent dioxygenase	17.50
1.0	1.0	0.1	0.1	TRINITY_DN5839_c0_g1_i2	O-methyltransferase	3.72
1.0	0.6	0.0	0.1	TRINITY_DN11613_c0_g3_i1	Cytochrome P450 CYP78	3.08
1.0	0.0	0.0	0.0	TRINITY_DN9958_c0_g1_i2	O-methyltransferase	9.15
1.0	0.1	0.0	0.0	TRINITY_DN238_c0_g2_i1	O-methyltransferase	6.59
1.0	0.4	0.0	0.2	TRINITY_DN14053_c0_g1_i1	Cytochrome P450 CYP80	2.43
1.0	0.0	0.0	0.0	TRINITY_DN4282_c0_g2_i1	Hydroxylase	17.34
1.0	0.0	0.2	0.0	TRINITY_DN10530_c0_g1_i1	Cytochrome P450 CYP86	5.65
1.0	0.0	0.1	0.1	TRINITY_DN13419_c0_g3_i1	UDP-glucose glucosyltransferase	2.90
1.0	0.0	0.0	0.0	TRINITY_DN908_c0_g1_i8	Dioxygenase	17.29
1.0	0.0	0.0	0.0	TRINITY_DN2404_c0_g1_i4	Cytochrome P450 CYP749	17.16
1.0	0.0	0.0	0.0	TRINITY_DN908_c0_g1_i3	Dioxygenase	7.21
1.0	0.0	0.2	0.3	TRINITY_DN8603_c0_g1_i2	Cytochrome P450 CYP83	1.77
1.0	0.6	0.7	0.1	TRINITY_DN51606_c0_g1_i1	O-methyltransferase	2.83
1.0	0.3	0.3	0.3	TRINITY_DN801_c0_g1_i10	Cytochrome P450 CYP749	1.98
1.0	0.1	0.5	0.0	TRINITY_DN12172_c0_g1_i1	O-methyltransferase	4.71
1.0	0.0	0.0	0.0	TRINITY_DN8982_c0_g1_i2	O-methyltransferase	16.96
1.0	0.0	0.1	0.0	TRINITY_DN6380_c0_g1_i1	Cytochrome P450 CYP76	4.79
1.0	0.1	0.0	0.0	TRINITY_DN908_c0_g1_i17	Dioxygenase	6.27
1.0	0.0	0.1	0.0	TRINITY_DN6380_c0_g1_i10	Cytochrome P450 CYP76	4.85
0.9	0.6	1.0	0.3	TRINITY_DN5058_c0_g1_i1	Indole-3-glycerol phosphate lyase	1.97
1.0	0.0	0.0	0.0	TRINITY_DN4025_c0_g1_i1	Cytochrome P450 CYP71	16.87
1.0	0.0	0.1	0.0	TRINITY_DN22668_c0_g1_i1	Cytochrome P450 CYP96	4.71
1.0	0.0	0.0	0.0	TRINITY_DN9958_c0_g1_i3	O-methyltransferase	16.76
1.0	0.0	0.1	0.0	TRINITY_DN134475_c0_g1_i1	O-methyltransferase	16.75
1.0	0.0	0.0	0.0	TRINITY_DN4282_c0_g1_i1	Hydroxylase	16.72
1.0	0.5	0.5	0.4	TRINITY_DN971_c0_g1_i3	Dioxygenase	1.34
1.0	0.0	0.0	0.1	TRINITY_DN14799_c0_g1_i4	2-oxoglutarate dependent dioxygenase	3.66

B

L. galeobdolon	*L. album*	*L. maculatum*	*L. orvala*	Gene	Annotation	LogFC
1.0	0.0	0.1	0.1	Contig_31519	Cytochrome P450 CYP72	3.95
1.0	0.1	0.1	0.2	Contig_13076	Cytochrome P450 CYP92	3.51
1.0	0.1	0.4	0.0	Contig_17476	Cytochrome P450 CYP72	2.70
1.0	0.0	0.0	0.0	Contig_31522	Cytochrome P450 CYP72	6.88
1.0	0.4	0.4	0.1	Contig_16829	Cytochrome P450 CYP72	1.67
1.0	0.0	0.0	0.0	Contig_8868	Cytochrome P450 CYP71	8.81
1.0	0.0	0.0	0.0	Contig_11095	Flavin-containing monooxygenase	7.79
1.0	0.0	0.0	0.0	Contig_30622	Cytochrome P450 CYP72	8.33
1.0	0.0	0.0	0.0	Contig_2002	Cytochrome P450 CYP72	8.31
1.0	0.0	0.0	0.0	Contig_14367	Cytochrome P450 CYP71	9.85
1.0	0.0	0.0	0.0	Contig_10649	UDP-glucosyltransferase	7.07
1.0	0.4	0.1	0.2	Contig_31446	Cytochrome P450 CYP83	2.19
1.0	0.0	0.2	0.2	Contig_2707	Cytochrome P450 CYP72	2.69
1.0	0.3	0.0	0.3	Contig_2935	Cytochrome P450 CYP71	2.33
1.0	0.2	0.3	0.2	Contig_6935	Indole-3-glycerol-phosphate lyase	2.14
1.0	0.0	0.3	0.0	Contig_30857	Dioxygenase	3.14
1.0	1.0	0.2	0.3	Contig_32831	UDP-glucosyltransferase	1.02
1.0	0.1	0.1	0.2	Contig_19787	Dioxygenase	2.55
1.0	0.2	0.2	0.4	Contig_5376	UDP-glucosyltransferase	1.92
1.0	0.3	0.3	0.3	Contig_1708	Cytochrome P450 CYP76	1.65
1.0	0.2	0.1	0.0	Contig_712	Flavin-containing monooxygenase	3.20
1.0	0.1	0.5	0.2	Contig_5450	Cytochrome P450 CYP714	2.01
1.0	0.0	0.1	0.8	Contig_14493	Cytochrome P450 CYP83	1.76
1.0	0.5	0.3	0.1	Contig_2780	Cytochrome P450 CYP82	1.86
0.9	1.0	0.3	0.0	Contig_11857	Cytochrome P450 CYP79	1.09
1.0	0.2	0.1	0.0	Contig_30397	Cytochrome P450 CYP76	3.02
1.0	0.3	0.3	0.4	Contig_8970	2-oxoglutarate-dependent dioxygenase	1.56
1.0	0.1	0.4	0.0	Contig_11225	Cytochrome P450 CYP76	2.51
1.0	0.4	0.6	0.2	Contig_11634	UDP-glucosyltransferase	1.37
1.0	0.1	0.1	0.0	Contig_15518	UDP-glucosyltransferase	3.79
1.0	0.4	0.0	0.0	Contig_11628	UDP-glucosyltransferase	2.86
1.0	0.6	0.5	0.2	Contig_22511	Cytochrome P450 CYP78	1.20
1.0	0.0	0.0	0.0	Contig_17353	Cytochrome P450 CYP716	7.37
1.0	0.5	0.6	0.3	Contig_22248	UDP-glucosyltransferase	1.21
1.0	0.5	0.1	0.4	Contig_830	UDP-glucosyltransferase	1.76
1.0	1.0	0.4	0.1	Contig_9571	Cytochrome P450 CYP96	1.00
1.0	0.6	0.0	0.0	Contig_20947	Cytochrome P450 CYP76	2.20
1.0	0.4	0.1	0.9	Contig_675	Cytochrome P450 CYP75	1.12
1.0	0.2	0.4	0.5	Contig_31681	Cytochrome P450 CYP75	1.62
1.0	0.1	0.1	0.0	Contig_27203	Cytochrome P450 CYP76	3.93
0.9	0.0	0.0	1.0	Contig_6474	Cytochrome P450 CYP704	1.47
1.0	0.0	0.0	0.0	Contig_32789	Cytochrome P450 CYP716	5.80
1.0	0.0	0.0	0.0	Contig_23988	Cytochrome P450 CYP76	9.33
1.0	0.1	0.0	0.0	Contig_18314	Cytochrome P450 CYP87	3.95
1.0	0.4	0.4	0.1	Contig_1641	Cytochrome P450 CYP78	1.70
1.0	0.1	0.5	0.6	Contig_4024	Cytochrome P450 CYP71	1.23
1.0	0.0	0.1	0.0	Contig_5757	Cytochrome P450 CYP72	5.82
1.0	0.2	0.1	0.0	Contig_10832	UDP-glucosyltransferase	3.60
1.0	0.7	0.7	0.0	Contig_21178	Dioxygenase	1.03
1.0	0.3	0.4	0.4	Contig_4269	Cytochrome P450 CYP72	1.41
1.0	0.3	0.1	0.0	Contig_30050	Cytochrome P450 CYP75	2.66
1.0	0.1	0.1	0.0	Contig_23142	Cytochrome P450 CYP71	3.46
1.0	0.1	0.3	0.0	Contig_15395	Cytochrome P450 CYP736	2.68
1.0	0.4	0.1	0.1	Contig_14423	UDP-glucosyltransferase	2.16
1.0	0.8	0.2	0.3	Contig_10495	Cytochrome P450 CYP734	1.20
1.0	0.2	0.2	0.5	Contig_27454	Cytochrome P450 CYP71	1.86
1.0	0.5	0.0	0.0	Contig_19157	Cytochrome P450 CYP87	2.61
1.0	0.1	0.2	0.5	Contig_403	Indole-3-glycerol-phosphate lyase	1.85

SI Figure 2: Complete list of BXD gene candidates from the transcriptomic analysis of *A. squarrosa* and *L. galeobdolon* A) 90 candidates were retrieved from *A. squarrosa* transcriptomic analysis comprising cytochrome P450s, 2-oxoglutarate-dependent dioxygenases, flavin-containing monooxygenases, polyphenol oxidases, O-methyltransferases, and UDP-glycosyltransferases. B) 57 candidates were retrieved from *L. galeobdolon* including cytochrome P450s, 2-oxoglutarate-dependent dioxygenases, flavin-containing monooxygenases, and UDP-glycosyltransferases. Values from 0 (lowest) to 1 (highest), indicate the relative expression of each gene among the tested conditions.

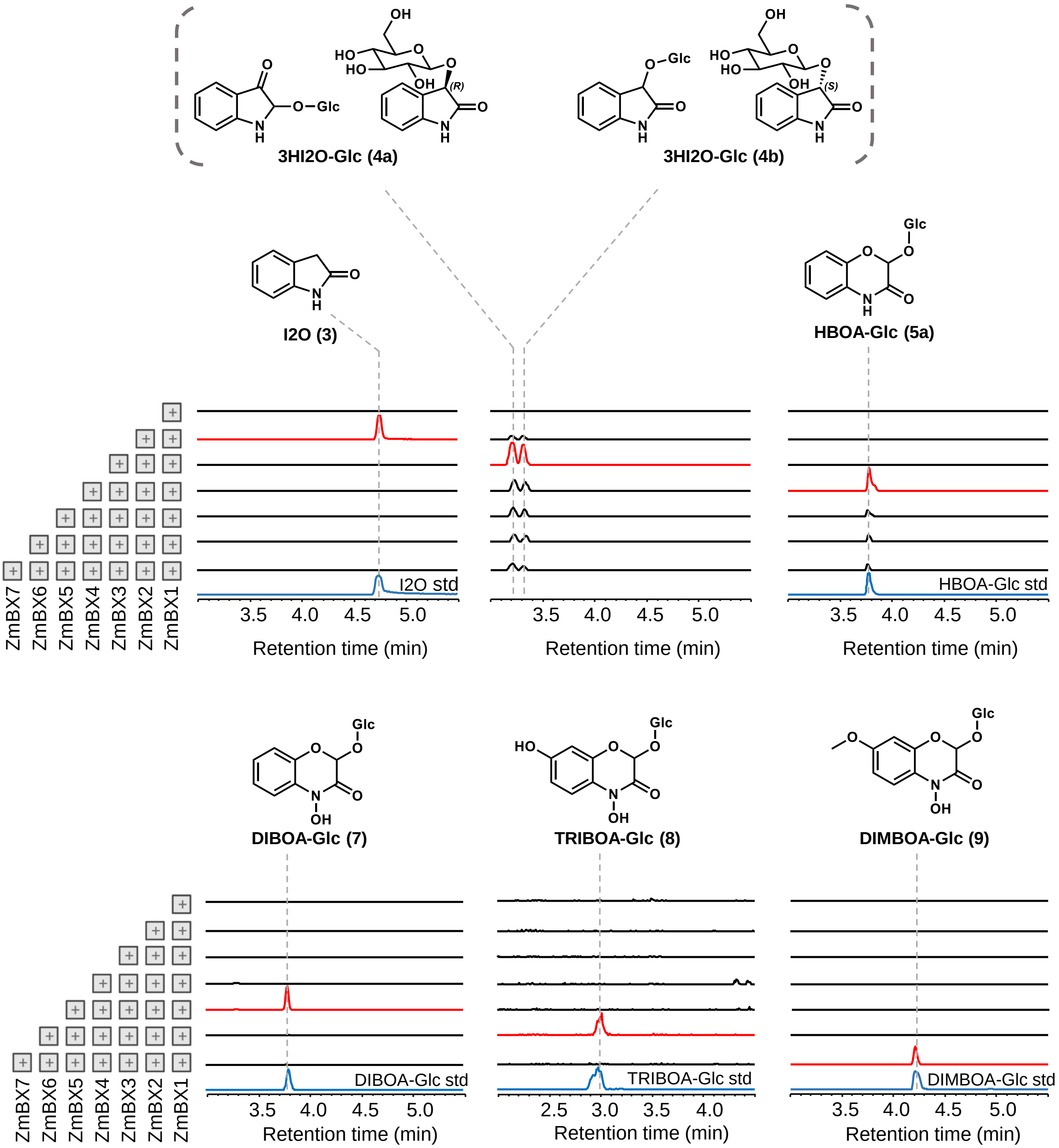

SI Figure 3: Establishing *Nicotiana benthamiana* as a platform for candidate gene screening. Combinatorial reconstitution of the *Zea mays* BXD pathway by *Agrobacterium tumefaciens*-mediated transient transformation of *N. benthamiana*. Different combinations of the maize *Bx* genes were expressed and LC-qTOF traces of leaf methanol extracts are displayed as extracted ion chromatograms for I2O (3) (M+H, *m/z* 134 ± 0.1), 3HI2O-Glc (4a, 4b) (M-H, *m/z* 310 ± 0.1), HBOA-Glc (5a) (M-H, *m/z* 326 ± 0.1), DIBOA-Glc (7) (M-H, *m/z* 342 ± 0.1), TRIBOA-Glc (8) (M+H, *m/z* 360 ± 0.1), and DIMBOA-Glc (9) (M-H, *m/z* 372 ± 0.1). We hypothesize that the two peaks detected for 3HI2O-Glc represent two isomers of 3HI2O-Glc (4a, 4b). The isomers were not characterized but the possible structures are shown. Compounds are numbered according to Fig. 1A.

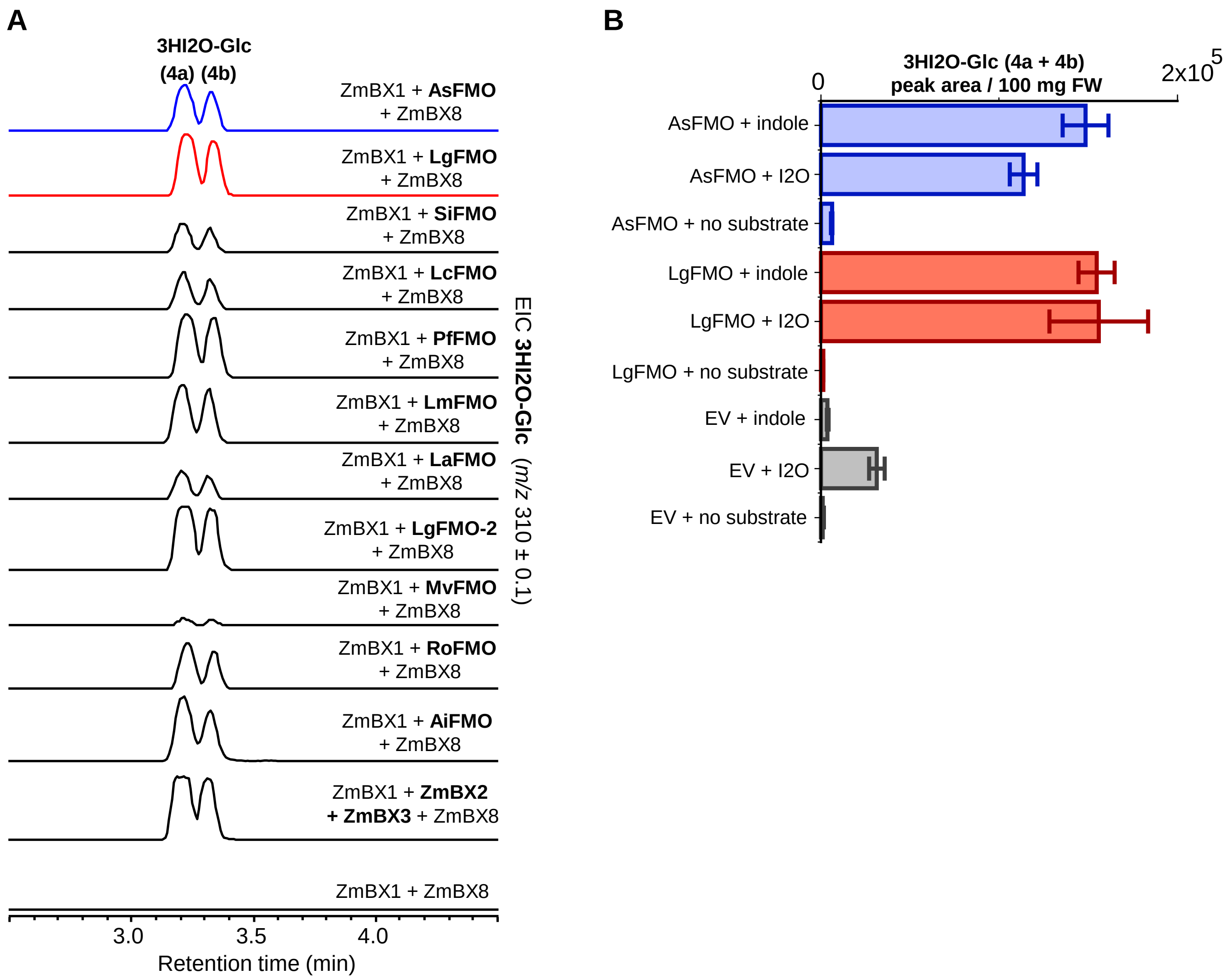

SI Figure 4: FMOs related to AsFMO and LgFMO from several dicot families display BX2/BX3 activity. A) Candidate FMO genes were transiently expressed with *ZmBx1* and *ZmBx8* in *N. benthamiana* and leaf methanol extracts were analyzed using LC-qTOF. AsFMO (*Aphelandra squarrosa*), LgFMO (*Lamium galeobdolon*), SiFMO (*Sesamum indicum* XM011080249.2), Lc (*Leonurus cardiac* c33258_g1_i2), Pf (*Phlomis fruticosa* c68310_g1_i1), Lm (*Lamium maculatum* Cluster-9204.5432), La (*Lamium album* Cluster-2496.6023), Mv (*Marrubium vulgare* c26472_g1_i2), Ro (*Rosmarinus officinalis* c40410_g1_i1), Ai (*Acanthus ilicifolius* TRINITY DN1050 c0 g1 i3). EIC, extracted ion chromatogram. B) AsFMO and LgFMO use both indole (2) and I2O (3) as substrate for 3HI2O-Glc (4a, 4b) formation. *AsFMO* and *LgFMO* were transiently expressed in *N. benthamiana* and leaves were infiltrated with indole (2) or I2O (3) as FMO substrates. Leaf methanol extracts were analyzed using LC-qTOF. Means ± SE (n = 3) are shown.

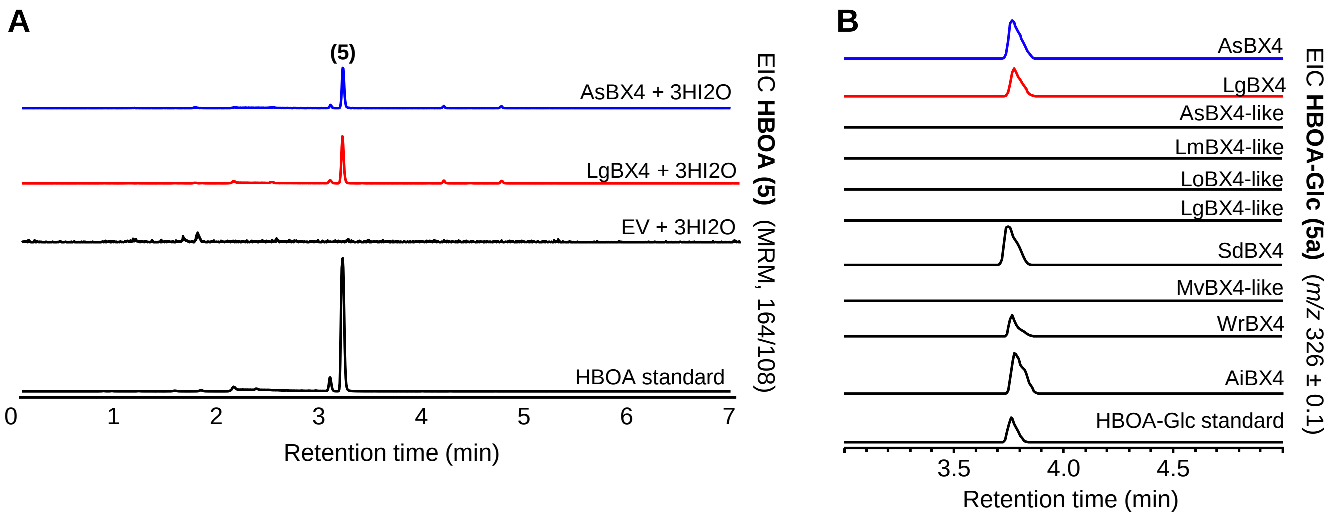

SI Figure 5: Microsome preparations of *AsBx4*- or *LgBx4*-expressing *Saccharomyces cerevisiae* showed conversion of 3HI2O (4) to HBOA (5). A) *AsBx4* and *LgBx4* were expressed in *S. cerevisiae* and yeast microsomes were incubated with 3HI2O (4) and NADPH. Enzyme products were analyzed using LC-MS/MS (MRM). HBOA formation was confirmed with an authentic standard. EIC, extracted ion chromatogram; EV, empty vector control. B) For testing potential BX4 activity of BX4-like enzymes from different eudicot species, the respective gene was transiently coexpressed with maize *ZmBx1-3* and *ZmBx8* in *N. benthamiana*. Leaf methanol extracts were analyzed using LC-qTOF. AsBX4-like (*A. squarrosa* TRINITY_DN4049_c0_g1_i2), LmBX4-like (*L. maculatum* Cluster-9204.7837), LoBX4-like (*L. orvala* Cluster-2218.4195), LgBX4-like (*L. galeobdolon* TRINITY_DN9871_c0_g1_i7), SdBX4 (*Scoparia dulcis* CYP92A46), MvBX4-like (*Marrubium vulgare* c20842_g1_i1), WrBX4 (*Wrightia religiosa* TRINITY_DN979_c0_g1_i1), AiBX4 (*Acanthus ilicifolius* TRINITY_DN9057_c0_g1_i13).

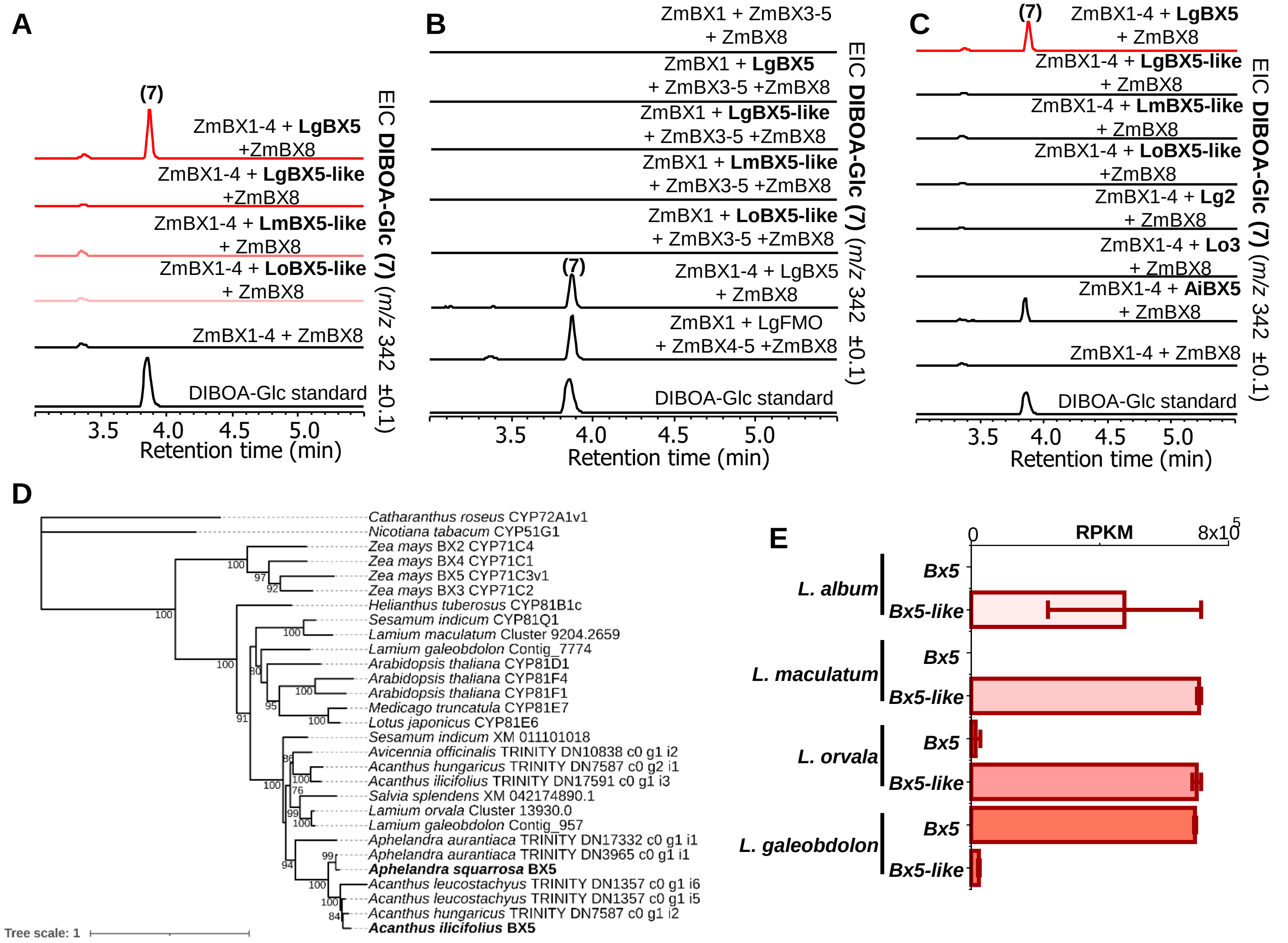

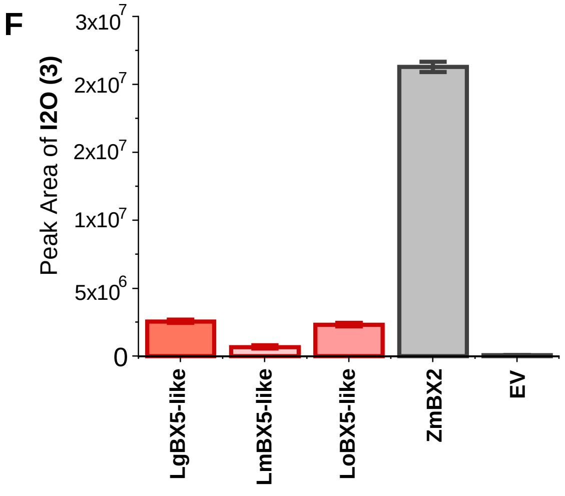

SI Figure 6: Characterization of BX5 and BX5-like proteins from Lamiaceae and Acanthaceae species. A) BX5-like from different *Lamium sp.* show no BX5 activity in *N. benthamiana*. *N. benthamiana* was transiently transformed with *ZmBx1*, *ZmBx2*, *ZmBx3*, *ZmBx4*, *ZmBx8*, and *L. galeobdolon*, *L. maculatum,* or *L. orvala Bx5-like* and methanol extracts of treated leaves were analyzed using LC-qTOF. EIC, extracted ion chromatogram. B) BX5-like from different *Lamium sp.* show no BX2 activity in *N. benthamiana*. LC-qTOF traces of methanolic extracts of *N. benthamiana* transiently transformed with *ZmBx1, ZmBx3, ZmBx4, ZmBx5, ZmBx8*, and *Bx5-like* from *L. galeobdolon*, *L. maculatum,* and *L.orvala* are shown. LgFMO was included as positive control. C) LC-qTOF analysis of methanol extracts of *N. benthamiana* leaves transiently expressing sequences close to *LgBx5* along with *ZmBx1, ZmBx2, ZmBx3, ZmBx4*, and *ZmBx8*. Sequence abbreviations: LgBX5 (*L. galeobdolon* BX5), LgBX5-like (*L. galeobdolon* BX5-like), LoBX5 (*L. orvala* BX5-like), Lg2 (*L. galobdolon* TRINITY DN4514 c0 g1 i3), Lo3 (*L. orvala* Cluster 10786.0), AiBX5 (*Acanthus ilicifolius* BX5). D) BX5 activity has likely a monophyletic origin in the Acanthaceae. *A. ilicifolius* BX5, which showed BX5 activity (SI Fig. 6C), clusters together with AsBX5. E) Expression of *Lamium Bx5 and Bx5-like* genes in mature leaves of different *Lamium sp.*. Expression was analyzed by RNAseq (n = 3 biological replicates ± SE) and RPKM values are given. F) Comparative activity of Lamium BX5 and ZmBX2 microsome preparation. Microsome preparations were incubated with indole and NADPH for 2h. I2O formation was measured trough targeted LC-MS. n = 4, SE is displayed.

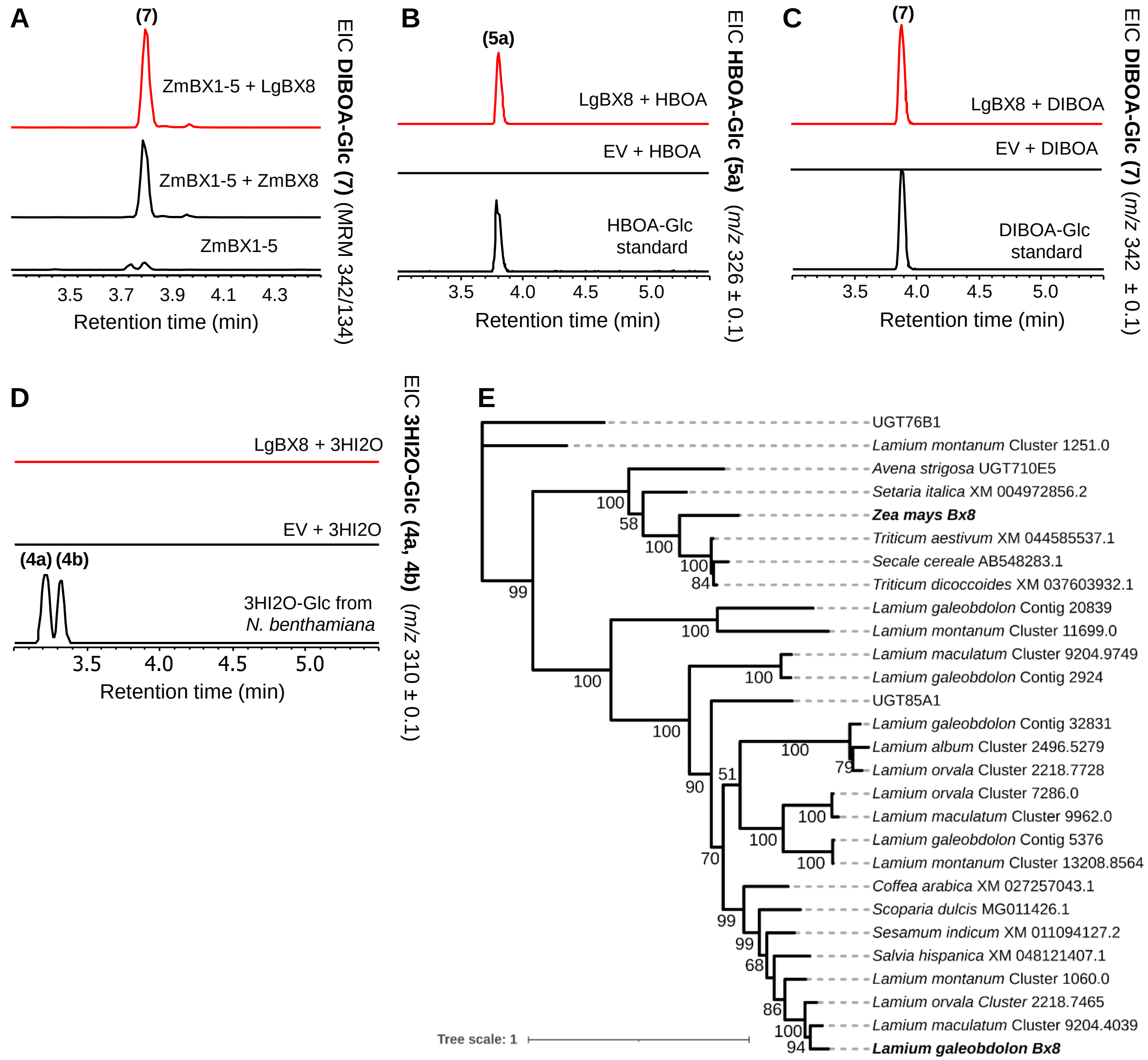

SI Figure 7: *In vivo* and *in vitro* characterization and phylogenetic analysis of LgBX8. A) *LgBx8* was expressed with *ZmBx1-5* in *Nicotiana benthamiana* and leaf extracts were analyzed using LC-MS/MS (MRM). B, C and D) Purified LgBX8 heterologously expressed in *Escherichia coli* was incubated with the potential substrates HBOA (5) (B), DIBOA (6) (C) and 3HI2O (4) (D). Enzyme products were analyzed using LC-qTOF. Accumulation of HBOA-Glc (5a) and DIBOA-Glc (7) was confirmed with authentic standards. In absence of 3HI2O-Glc (4a, 4b) authentic standard, 3HI2O-Glc (4a, 4b) traces from *N. benthamiana* ZmBX1-BX3 reconstitution were used as putative standards. EIC, extracted ion chromatogram. EV, empty vector control. E) Maximum likelihood phylogenetic analysis of LgBX5, ZmBX5, and related proteins from monocot and Lamiales species.

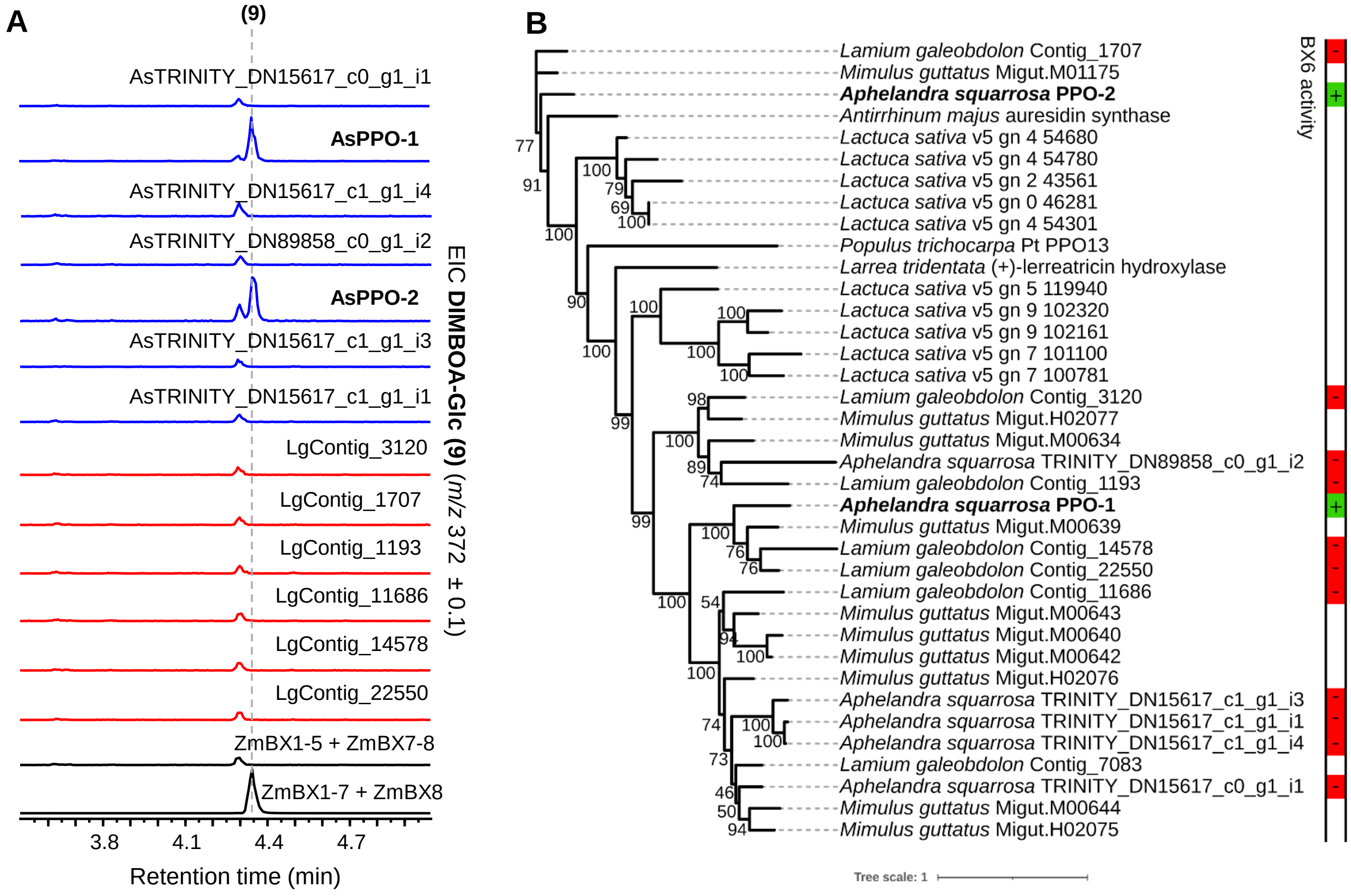

SI Figure 8: Two polyphenol oxidases (PPOs) from *Aphelandra squarrosa* have BX6 activity. A) Candidate genes were transiently expressed with maize *ZmBx1-5* + *ZmBx7-8* in *N. benthamiana* and methanolic leaf extracts were analyzed using LC-qTOF to measure DIMBOA-Glc accumulation (9). EIC, extracted ion chromatogram. B) Maximum likelihood phylogenetic analysis of *A. squarrosa* and *L. galeobdolon* PPOs that were assessed for BX6 activity (green (+) = active, red (-) = inactive).

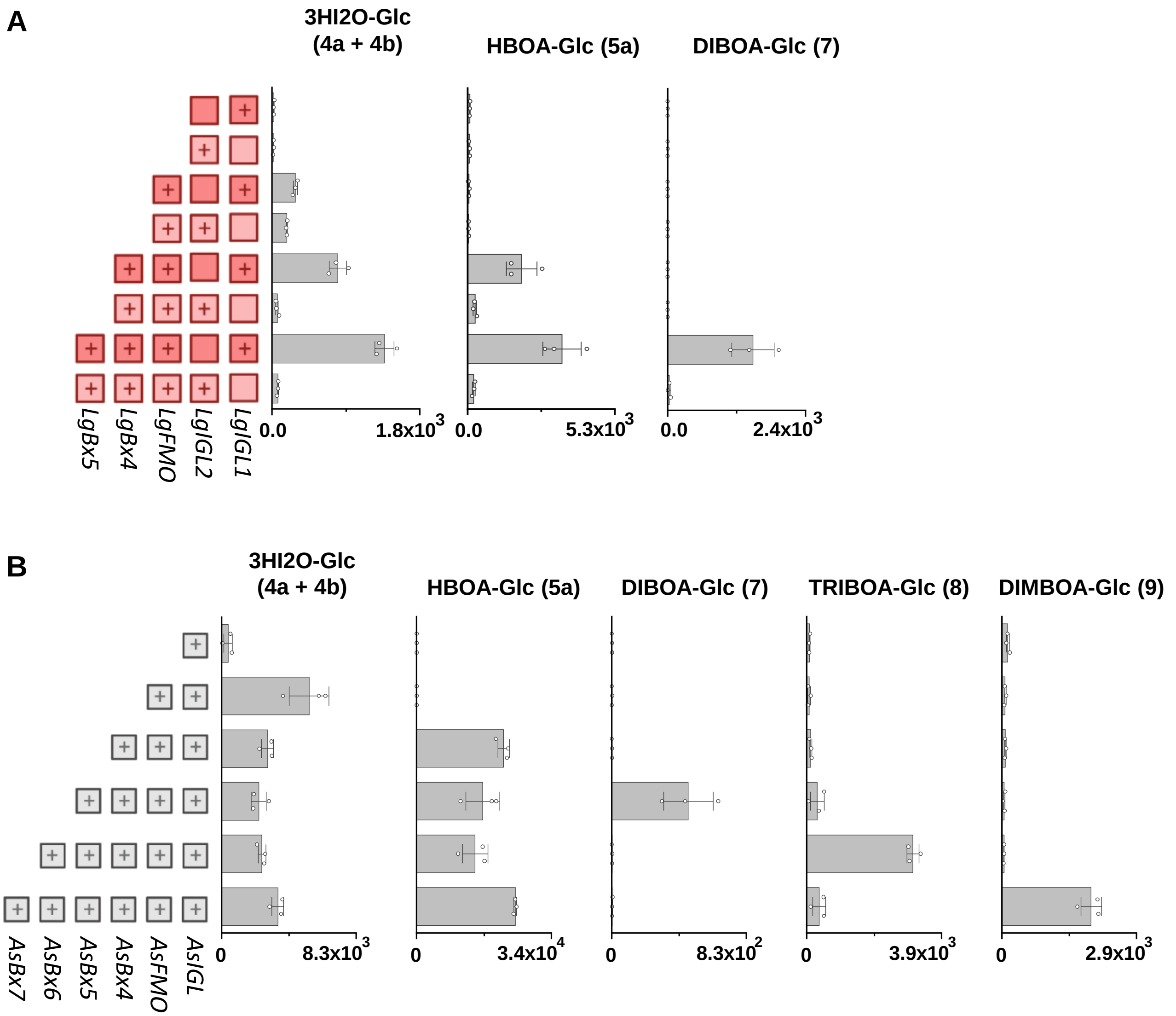

SI Figure 9: Expression of *LgBx* and *AsBx* genes, respectively, in *Nicotiana benthamiana* lead to the functional reconstitution of the *Aphelandra squarrosa* and *Lamium galeobdolon* BXD pathways. *L. galeobdolon Bx* genes (A) and *A. squarrosa Bx* genes (B), respectively, were transiently expressed in leaves of *N. benthamiana* and leaf methanol extracts were analyzed using LC-qTOF. Squares containing a '+' symbol indicate the presence of the respective gene in the transformation mixture. All combinations of *Lamium Bx* genes tested contained *LgBx8*, while *Aphelandra Bx* genes were always co-expressed with maize *ZmBx8*. Shown are averaged peak areas / mg fresh weight ± SE (n = 3 biological replicates).

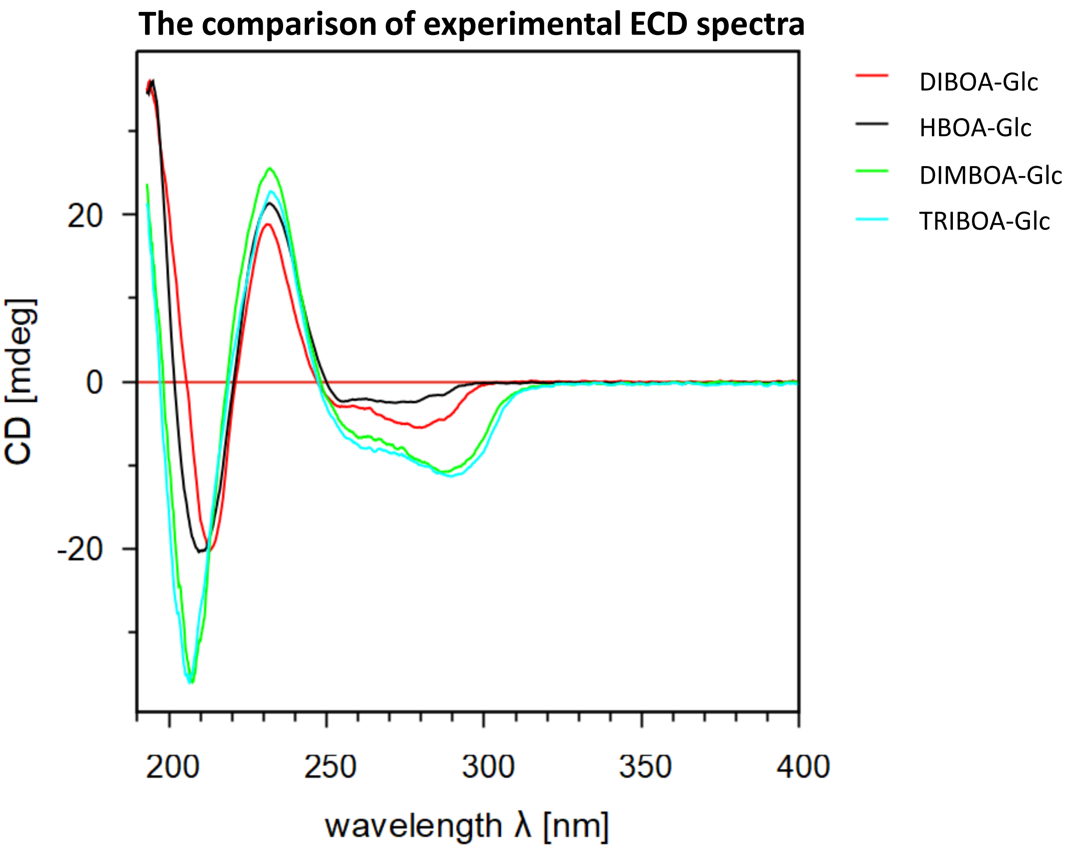

SI Figure 10: Comparison of experimental ECD spectra for HBOA-Glc, DIBOA-Glc, TRIBOA-Glc, and DIMBOA-Glc. (2*R*)-DIBOA-Glc and (2*R*)-DIMBOA-Glc exhibited positive Cotton effects around 230 nm (Nagao *et al.,*1985). A positive Cotton effect at 228 nm and a negative at 291 nm have been observed in (2*R*)-DIMBOA-Glc (Hartenstein *et al.,* 1993)). The optical rotation for DIBOA-Glc was determined as $[a]_D^{20}$ +66.1 (c 0.47, H_2O). This is in accordance with literature values for the (2*R*)-isomer (Hartenstein *et al.,* 1994)). We therefore concluded TRIBOA to have also a (2*R*)-configuration.

Nagao T, Otsuka H, Kohda H, Sato T, Yamasaki K (1985) Benzoxazinones from *Coix lachryma-jobi* var. ma-yuen. *Phytochemistry* 24: 2959-2962

Hartenstein H, Klein J, Sicker D (1993) Efficient isolation procedure for (2*R*)-b-D-glucopyranosyloxy-4-hydroxy-7-methoxy-2*H*-1,4-benzoxazin-3(4*H*)-one from maize. *Indian J. Heterocyclic Chem* 2: 151-153

Hartenstein H, Sicker D (1994) (2*R*)-2-b-D-glucopyranosyloxy-4-hydroxy-2*H*-1,4-benzoxazin-3(4*H*)-one from *Secale cereale*. *Phytochemistry* 35: 827-828

Supplemental Table 1: Percentages of amino acid sequence identities between tested enzymes. The tables display the percentage of AA sequence identity between enzymes used in phylogenetic inference. Sequences were aligned with MUSCLE 5.1. The results are reported for FMOs (A), BX4 (B), BX5 (C), BX6 (D), BX7 (E), and BX8 (F). Green or red coloring, respectively, indicates presence or absence of the activity indicated in the title of the table.

A Percentage of AA seq identity among functionally tested FMOs

	Achantus ilicifolius TRINITY_DN1050_c0_g1_i3	Aphelandra squarrosa Bx2/Bx3	Lamium album Cluster-2496.6023	Lamium galeobdolon Contig_33225	Lamium galeobdolon Bx2/Bx3	Lamium maculatum Cluster-9204.5432	Leonurus cardiaca c33258_g1_i2	Marrubium vulgare c26472_g1_i2	Phlomis fruticosa c68310_g1_i1	Rosmarinus officinalis c40410_g1_i1	Sesamum indicum XM_011080249.2
Achantus ilicifolius TRINITY_DN1050_c0_g1_i3		81.4	60.7	62.1	63.7	59.9	61.2	64.4	59.2	60.1	67.6
Aphelandra squarrosa_Bx2/Bx3	81.4		62.4	63.2	65	61.7	64.2	67.3	61.5	63.1	70.4
Lamium album Cluster-2496.6023	60.7	62.4		88.5	87.1	72.1	83.2	74	81.3	68.6	72.3
Lamium galeobdolon Contig_33225	62.1	63.2	88.5		89.2	72.4	84.1	75.8	79.7	69.7	74.7
Lamium galeobdolon_Bx2/Bx3	63.7	65	87.1	89.2		73.2	82.7	74.9	77.5	68.9	72.6
Lamium maculatum Cluster-9204.5432	59.9	61.7	72.1	72.4	73.2		71.6	70.9	68.4	65.6	70.9
Leonurus cardiaca c33258_g1_i2	61.2	64.2	83.2	84.1	82.7	71.6		75.9	81.9	68.9	74.1
Marrubium vulgare c26472_g1_i2	64.4	67.3	74	75.8	74.9	70.9	75.9		73.1	71	75.8
Phlomis fruticosa c68310_g1_i1	59.2	61.5	81.3	79.7	77.5	68.4	81.9	73.1		67.3	72.7
Rosmarinus officinalis c40410_g1_i1	60.1	63.1	68.6	69.7	68.9	65.6	68.9	71	67.3		69.6
Sesamum indicum XM_011080249.2	67.6	70.4	72.3	74.7	72.6	70.9	74.1	75.8	72.7	69.6	

B Percentage of AA seq identity among functionally tested BX4

	Achantus ilicifolius TRINITY_DN9057_c0_g1_i13	Aphelandra squarrosa BX4	Aphelandra squarrosa TRINITY DN4049 c0 g1 i2	Lamium galeobdolon Contig_675	Lamium galeobdolon BX4	Lamium maculatum Cluster-9204.7837	Lamium orvala Cluster-2218.4195	Marrubium vulgare c20842_g1_i1	Scoparia dulcis CYP92A46	Wrightia_religiosa scaffold_2074295	Zea mays BX4
Achantus ilicifolius TRINITY_DN9057_c0_g1_i13		83.9	59.6	57.1	60	56.4	59.5	52	57.1	53.2	30.2
Aphelandra squarrosa BX4	83.9		59.4	57	59.5	57.1	58.2	51.2	55.8	52.5	31.4
Aphelandra squarrosa TRINITY DN4049 c0 g1 i2	59.6	59.4		59.2	60	61.9	60.6	54	55.3	52.9	29.3
Lamium galeobdolon Contig_675	57.1	57	59.2		79.1	79.3	81.5	60.7	57.2	55.5	29.2
Lamium galeobdolon BX4	60	59.5	60	79.1		85.7	86.2	61	60.5	56.7	29.3
Lamium maculatum Cluster-9204.7837	56.4	57.1	61.9	79.3	85.7		88.9	60.1	59.1	57.3	28.9
Lamium orvala Cluster-2218.4195	59.5	58.2	60.6	81.5	86.2	88.9		60.6	59	56.7	30
Marrubium vulgare c20842_g1_i1	52	51.2	54	60.7	61	60.1	60.6		55.8	55	28.6
Scoparia dulcis CYP92A46	57.1	55.8	55.3	57.2	60.5	59.1	59	55.8		54.8	29.9
Wrightia_religiosa scaffold_2074295	53.2	52.5	52.9	55.5	56.7	57.3	56.7	55	54.8		30.8
Zea mays BX4	30.2	31.4	29.3	29.2	29.3	28.9	30	28.6	29.9	30.8	

C Percentage of AA seq identity among functionally tested BX5

	Acanthus ilicifolius BX5	Aphelandra squarrosa BX5	Lamium galeobdolon BX5	Lamium galeobdolon BX5-like	Lamium galeobdolon Contig_32188	Lamium maculatum BX5-like	Lamium orvala BX5-like	Lamium orvala Cluster-10786.0	Zea mays BX5
Acanthus ilicifolius BX5		77.5	28.7	28.8	30.9	28.2	28	26.8	25.2
Aphelandra squarrosa BX5	77.5		29.5	29.1	29.4	28.5	28.5	28.4	25.6
Lamium galeobdolon BX5	28.7	29.5		87.1	60.6	84.5	85.3	68.1	32.9
Lamium galeobdolon BX5-like	28.8	29.1	87.1		65.7	93.5	95	72.6	33.3
Lamium galeobdolon Contig_32188	30.9	29.4	60.6	65.7		64.9	65.1	62.4	32
Lamium maculatum BX5-like	28.2	28.5	84.5	93.5	64.9		95.4	71.6	32.8
Lamium orvala BX5-like	28	28.5	85.3	95	65.1	95.4		70.8	32.6
Lamium orvala Cluster-10786.0	26.8	28.4	68.1	72.6	62.4	71.6	70.8		32.5
Zea mays BX5	25.2	25.6	32.9	33.3	32	32.8	32.6	32.5	

D Percentage of AA seq identity among functionally tested BX6

	Aphelandra squarrosa BX6	Zea mays BX6
Aphelandra squarrosa BX6		29.6
Zea mays BX6	29.6	

E Percentage of AA seq identity among functionally tested BX7

	Aphelandra squarrosa BX7	Zea mays BX7
Aphelandra squarrosa BX7		27.5
Zea mays BX7	27.5	

F Percentage of AA seq identity among functionally tested BX8

	Lamium galeobdolon BX8	Zea mays BX8
Lamium galeobdolon BX8		31.4
Zea mays BX8	31.4	

Supplemental Table 2: Primers used in this study.

Accession	Species	Forward primer	Reverse primer	Vector	Host
TRINITY_DN10074_c0_g1_i1	*Aphelandra squarrosa*	ATATAGCGGCCGCAATGTCGCCTACCTTGATCTTG	TTAATTAATCACATATAAGGTTTGGCAATC	pCambia2300	*N. benthamiana*
TRINITY_DN10074_c0_g1_i2	*Aphelandra squarrosa*	ATATAGCGGCCGCAATGGAATTCGAATTTCCTTTCAAC	TTAATTAATCACATATAAGGTTTGGCAATC	pCambia2300	*N. benthamiana*
TRINITY_DN5474_c0_g1_i7	*Aphelandra squarrosa*	ATATAGCGGCCGCAATGGAGATCATCCAGTTGTTG	TTAATTAATTAGGTCACCGGTCTTTTAATC	pCambia2300	*N. benthamiana*
TRINITY_DN2050_c0_g1_i5	*Aphelandra squarrosa*	TTATGAATTTTGCAGATGGAACTGCTGACCAGCTCA	GACAACCACAACAAGCACCGTCAATAGAGGTGCGGGGATAGTCT	3Ω1	*N. benthamiana*
TRINITY_DN1895_c0_g1_i6	*Aphelandra squarrosa*	TTTATGAATTTTGCAGCTCGATGGAGATACTGTCCATCACTGTAG	GACAACCACAACAAGCACCGTTAAATTCGTTCCAAGATTAACTGAG	3Ω1	*N. benthamiana*
TRINITY_DN2050_c0_g1_i6	*Aphelandra squarrosa*	TTTATGAATTTTGCAGCTCGATGGAAGTGGTGACCAGCTC	GACAACCACAACAAGCACCGCTAATAGAGATGAGAAGAGAGC	3Ω1	*N. benthamiana*
TRINITY_DN2050_c0_g1_i1	*Aphelandra squarrosa*	TTTATGAATTTTGCAGCTCGATGGAACTGCTGACCAGCTC	GACAACCACAACAAGCACCGTTAATAGAGGTGCGGGGAGAG	3Ω1	*N. benthamiana*
TRINITY_DN4480_c0_g1_i1	*Aphelandra squarrosa*	TTTATGAATTTTGCAGCTCGATGGCCAAAGTCTGTAACCAAATTC	GACAACCACAACAAGCACCGTTAGTAAAGGTGGGGTGGGAG	3Ω1	*N. benthamiana*
		CAACCCTCACTAAAGGGCATGGCCAAAGTCTGTAACCAAATTC	CTCTGGCGAAGAATTGTTAATTTAGTAAAGGTGGGGTGGGAG	pESC-Leu	*S. cerevisiae*
TRINITY_DN6380_c0_g1_i11	*Aphelandra squarrosa*	TTTATGAATTTTGCAGCTCGATGAATCTTCCACAGTTGGCC	GACAACCACAACAAGCACCGTCAATGGTAGAGCTTTGGATTGG	3Ω1	*N. benthamiana*
TRINITY_DN5373_c0_g1_i2	*Aphelandra squarrosa*	TTTATGAATTTTGCAGCTCGATGATGCTCGAAACACCCCTAC	GACAACCACAACAAGCACCGCTAATTCGGCAACGAAGCCAC	3Ω1	*N. benthamiana*
TRINITY_DN1999_c0_g1_i4	*Aphelandra squarrosa*	TTTATGAATTTTGCAGCTCGATGGCCTGGATTTGGACGG	GACAACCACAACAAGCACCGTTAATTGCACAACCGATAGTTAGGAAT	3Ω1	*N. benthamiana*
TRINITY_DN3965_c0_g1_i1	*Aphelandra squarrosa*	TTATGAATTTTGCAGATGGACGTGTCAATAACCGAAATC	GACAACCACAACAAGCACCGTCAGGCAATGACGGTAGCC	3Ω1	*N. benthamiana*
TRINITY_DN990_c0_g1_i4	*Aphelandra squarrosa*	TTTATGAATTTTGCAGCTCGATGGGTGGTCTCTTGCCCA	GACAACCACAACAAGCACCGCTACAGATGATGCAGAACAACG	3Ω1	*N. benthamiana*
TRINITY_DN2021_c0_g1_i2	*Aphelandra squarrosa*	TTTATGAATTTTGCAGCTCGATGGAGGTCTCGCACCTG	GACAACCACAACAAGCACCGTCACTTAATTTTCCGCATGGTTAAG	3Ω1	*N. benthamiana*
TRINITY_DN3629_c1_g2_i1	*Aphelandra squarrosa*	TTTATGAATTTTGCAGCTCGATGTGCATTGTCATTAAAACACACAG	GACAACCACAACAAGCACCGCTAAACAGGGGCAGTCTTAG	3Ω1	*N. benthamiana*
TRINITY_DN5057_c0_g1_i2	*Aphelandra squarrosa*	TTTATGAATTTTGCAGCTCGATGGCTATCTTCTTCTTCCTCC	GACAACCACAACAAGCACCGTTACATGTCGACAGCCAAACGTTTG	3Ω1	*N. benthamiana*
TRINITY_DN14457_c0_g1_i3	*Aphelandra squarrosa*	TTATGAATTTTGCAGATGGAGAACCCACTGCTG	GACAACCACAACAAGCACCGTTATGAGTTGTGGAGTATGG	3Ω1	*N. benthamiana*
TRINITY_DN2569_c0_g1_i1	*Aphelandra squarrosa*	TTTATGAATTTTGCAGCTCGATGGAAGGCAGTCTGCTGTACAC	GACAACCACAACAAGCACCGCTAATCCACATTATCTGATTCTTGAATAAC	3Ω1	*N. benthamiana*
		CAACCCTCACTAAAGGGCATGGAAGGCAGTCTGCTGTACAC	CTCTGGCGAAGAATTGTTAATCTAATCCACATTATCTGATTCTTGAATAAC	pESC-Leu	*S. cerevisiae*
TRINITY_DN4049_c0_g1_i2	*Aphelandra squarrosa*	TTTATGAATTTTGCAGCTCGATGTTCATGAACATGGTGGACTTC	GACAACCACAACAAGCACCGTCAGTAAAGGTGAGGTGGAAGTG	3Ω1	*N. benthamiana*
TRINITY_DN37698_c0_g1_i1	*Aphelandra squarrosa*	TTTATGAATTTTGCAGCTCGATGGACGTGTGGAGCATCAAAC	GACAACCACAACAAGCACCGTCACAAGGAATACATTTCAAGTGAAAG	3Ω1	*N. benthamiana*

TRINITY_DN84281_c0_g1_i1	*Aphelandra squarrosa*	TTTATGAATTTTGCAGCTCGATGAGCGCTGCAGCCTTGATC	GACAACCACAACAAGCACCGTCACGGCTGAGTTTGGTAGAC	3Ω1	*N. benthamiana*
TRINITY_DN3189_c0_g1_i3	*Aphelandra squarrosa*	TTTATGAATTTTGCAGCTCGATGGAGATTCTCACCCACTACC	GACAACCACAACAAGCACCGTCAAAGCTGTGACAGAAGATGGC	3Ω1	*N. benthamiana*
TRINITY_DN6845_c0_g1_i8	*Aphelandra squarrosa*	TTTATGAATTTTGCAGCTCGATGGAGATCCATCTACCTTCAAACT	GACAACCACAACAAGCACCGTCAGTCCAAAGACTTGTAGACAAC	3Ω1	*N. benthamiana*
TRINITY_DN1895_c0_g1_i9	*Aphelandra squarrosa*	TTTATGAATTTTGCAGCTCGATGGAGATACTGTCCATCACTGTAG	GACAACCACAACAAGCACCGTTAAATTCGTTCCAAGATTAACTGAGC	3Ω1	*N. benthamiana*
TRINITY_DN4349_c0_g1_i1	*Aphelandra squarrosa*	TTTATGAATTTTGCAGCTCGATGGCTGCCGCTAACTCTCTC	GACAACCACAACAAGCACCGTTAGAAGCCGTTAGAAGCCGG	3Ω1	*N. benthamiana*
TRINITY_DN5746_c0_g1_i1	*Aphelandra squarrosa*	TTTATGAATTTTGCAGCTCGATGTCTTCATATTTGGACAAGGCTTC	GACAACCACAACAAGCACCGTCATTTAGAATGAGCGATCAAATTAG	3Ω1	*N. benthamiana*
TRINITY_DN3189_c0_g1_i2	*Aphelandra squarrosa*	TTTATGAATTTTGCAGCTCGATGGAGATTCTCACCCACTACC	GACAACCACAACAAGCACCGTCAAAGCTGTGACAGAAGATGGC	3Ω1	*N. benthamiana*
TRINITY_DN476_c0_g1_i6	*Aphelandra squarrosa*	TTTATGAATTTTGCAGCTCGATGATGAGGTTGGTTTCAGTTGG	GACAACCACAACAAGCACCGCTACTTTTGGTACATTGATGCAGAC	3Ω1	*N. benthamiana*
TRINITY_DN16988_c0_g1_i5	*Aphelandra squarrosa*	TTTATGAATTTTGCAGCTCGATGGATGGTTACCAACAGCTGATC	GACAACCACAACAAGCACCGTTAATTCTCATATGAGGTAACCGACAC	3Ω1	*N. benthamiana*
TRINITY_DN9249_c0_g1_i3	*Aphelandra squarrosa*	TTTATGAATTTTGCAGCTCGATGGCACCTGAATTCTCCCTC	GACAACCACAACAAGCACCGTTAACCTCGCCTGTCTCGG	3Ω1	*N. benthamiana*
TRINITY_DN20009_c0_g1_i1	*Aphelandra squarrosa*	TTTATGAATTTTGCAGCTCGATGTTCGTCTATATAACAAGTTTATCC	GACAACCACAACAAGCACCGCTAATTCACCAATGTAGCCACCAAG	3Ω1	*N. benthamiana*
TRINITY_DN13750_c0_g1_i1	*Aphelandra squarrosa*	TTTATGAATTTTGCAGCTCGATGGCACCCACTGTTACAACTTC	GACAACCACAACAAGCACCGTCATATCCTAAGCAAAGAAAGAGCC	3Ω1	*N. benthamiana*
TRINITY_DN4513_c0_g1_i5	*Aphelandra squarrosa*	TTTATGAATTTTGCAGCTCGATGGATCTTCTCCTCGTCGAG	GACAACCACAACAAGCACCGTTATTTTCGGGCTGAAGATCTTGG	3Ω1	*N. benthamiana*
TRINITY_DN2050_c0_g1_i4	*Aphelandra squarrosa*	ATGGAAGTGGTGACCAGC	TTAATAGAGTTGAGAAGAGAGCCT	pCambia2300	*N. benthamiana*
TRINITY_DN14367_c0_g1_i2	*Aphelandra squarrosa*	ATGTATAATTCAGTCAAGGATGATC	TTATGGCGGCAAGGGA	pCambia2300	*N. benthamiana*
TRINITY_DN476_c0_g1_i8	*Aphelandra squarrosa*	ATGATCATCCCTTCAGATCTATT	TTAGCGAGTTTGGTACATTGA	pCambia2300	*N. benthamiana*
TRINITY_DN5474_c0_g1_i4	*Aphelandra squarrosa*	ATGCCAATGGAGATCATCC	TCACCAATGAATTGCATCG	pCambia2300	*N. benthamiana*
TRINITY_DN4025_c0_g1_i2	*Aphelandra squarrosa*	ATGGAGATCCAGCTACCTT	TTAATTGGAAGCGTTGTAGAG	pCambia2300	*N. benthamiana*
TRINITY_DN4265_c0_g1_i1	*Aphelandra squarrosa*	ATGGAAATTATTGGCATGGAG	TCAGTTGACATGAGTGAGAAG	pCambia2300	*N. benthamiana*
TRINITY_DN28850_c0_g1_i2	*Aphelandra squarrosa*	ATGGAAACTTTGTTGGTTCTC	CTAAAGGTTGCGGAGAATGA	pCambia2300	*N. benthamiana*
TRINITY_DN33184_c0_g1_i1	*Aphelandra squarrosa*	ATGGAAGCAAAACTAGGGT	TCAGGATATAGACAGTGGTGG	pCambia2300	*N. benthamiana*
TRINITY_DN11835_c0_g1_i1	*Aphelandra squarrosa*	ATGGACAATGTAGTAATGTTGTT	TTATTTAAGCGGCGGCA	pCambia2300	*N. benthamiana*
TRINITY_DN7413_c0_g2_i3	*Aphelandra squarrosa*	ATGGATTTGTGGGTGGTG	TCAAACCCCATCTCCGG	pCambia2300	*N. benthamiana*
TRINITY_DN111532_c0_g1_i1	*Aphelandra squarrosa*	ATGATCTCTATTGCAATATGCAT	TCACACCCATCTACTCAAAA	pCambia2300	*N. benthamiana*
TRINITY_DN5547_c0_g1_i1	*Aphelandra squarrosa*	ATGTCCAAATTACATCTGTTATTACA	TCAACAATGATGATCAGAAGC	pCambia2300	*N. benthamiana*
TRINITY_DN151_c1_g1_i1	*Aphelandra squarrosa*	ATGAGCTTACTAGAAATCGTGA	TTAAGATGGGGTGGCATATC	pCambia2300	*N. benthamiana*

TRINITY_DN151_c1_g1_i1	*Aphelandra squarrosa*	ATGGCAACTGCCTTTCA	TTAAATCAAAGCCGGCATAAG	pCambia2300	*N. benthamiana*
TRINITY_DN36592_c0_g1_i1	*Aphelandra squarrosa*	ATGGAGTTTCACTACATCCTC	CTAACTCAATTGGACAACCC	pCambia2300	*N. benthamiana*
TRINITY_DN8666_c0_g1_i2	*Aphelandra squarrosa*	TTTATGAATTTTGCAGCTCGATGCACACCAAATCCCTCTTCTT	GACAACCACAACAAGCACCGCTAGTCAGCCTTTTGGGTAAAC	3Ω1	*N. benthamiana*
TRINITY_DN8666_c0_g1_i3	*Aphelandra squarrosa*	TTTATGAATTTTGCAGCTCGATGCACACCAAATCCCTCTTCTTTAT	GACAACCACAACAAGCACCGCTAGTCAGCCTTTTGGGTAAACTT	3Ω1	*N. benthamiana*
TRINITY_DN6556_c0_g1_i1	*Aphelandra squarrosa*	TTTATGAATTTTGCAGCTCGATGCAGATGAAGAAAACGGTGG	GACAACCACAACAAGCACCGTCACTTGGCAAAATAACTGGGGTTG	3Ω1	*N. benthamiana*
		AAGTTCTGTTTCAGGGCCCGCAGATGAAGAAAACGGTGG	ATGGTCTAGAAAGCTTTACTTGGCAAAATAACTGGGGTTG	pOPINF	*E. coli*
		AAGTTCTGTTTCAGGGCCCGAAATATGCCCTCTCCATCGGC	ATGGTCTAGAAAGCTTTACTTGGCAAAATAACTGGGGTTG	pOPINF	*E. coli*
		CAACCCTCACTAAAGGGCATGCAGATGAAGAAAACGGTGG	CTCTGGCGAAGAATTGTTAATTCACTTGGCAAAATAACTGGGGTTG	pESC-Leu	*S. cerevisiae*
TRINITY_DN20_c7_g1_i2	*Aphelandra squarrosa*	TTTATGAATTTTGCAGCTCGATGTGGAGGACAGCGTCGTTAAAC	GACAACCACAACAAGCACCGTCACTTAGCTTGAGATGATTCCATAAAC	3Ω1	*N. benthamiana*
TRINITY_DN13400_c0_g1_i1	*Aphelandra squarrosa*	TTTATGAATTTTGCAGCTCGATGGCGGCAGCAAACCTC	GACAACCACAACAAGCACCGTTAATTCTTGAAGAGGTCTAAGCC	3Ω1	*N. benthamiana*
		AAGTTCTGTTTCAGGGCCCGATGGCGGCAGCAAACCTC	ATGGTCTAGAAAGCTTTATTAATTCTTGAAGAGGTCTAAGCC	pOPINF	*E. coli*
TRINITY_DN3886_c0_g1_i4	*Aphelandra squarrosa*	TTTATGAATTTTGCAGCTCGATGAATAGCCCTAGGGATTGG	GACAACCACAACAAGCACCGTTACTTCTCCAAATTTGAGGATCCTTC	3Ω1	*N. benthamiana*
TRINITY_DN20430_c0_g1_i1	*Aphelandra squarrosa*	TTTATGAATTTTGCAGCTATGTCTACTCCTATTCCTTCTCC	GACAACCACAACAAGCACCGTCAATGCTCTTTCTTTTGCAGCA	3Ω1	*N. benthamiana*
TRINITY_DN14690_c0_g1_i1	*Aphelandra squarrosa*	TTTATGAATTTTGCAGCTCGATGGCTGCCACGCCTCCACC	GACAACCACAACAAGCACCGCTAATTCTGAATATGGACCTGTTTG	3Ω1	*N. benthamiana*
TRINITY_DN52892_c0_g1_i1	*Aphelandra squarrosa*	TTTATGAATTTTGCAGCTCGATGAACTGCTTGATGCAAGGTTG	GACAACCACAACAAGCACCGCTAATCATCACCATTAACAGTAGCAG	3Ω1	*N. benthamiana*
TRINITY_DN5839_c0_g1_i1	*Aphelandra squarrosa*	TTTATGAATTTTGCAGCTCGATGGATCATCATGAGTCCGATGAG	GACAACCACAACAAGCACCGTCATTTAAGAAATTCCATGATCCAGTATC	3Ω1	*N. benthamiana*
		AAGTTCTGTTTCAGGGCCCGATGGATCATCATGAGTCCGATG	ATGGTCTAGAAAGCTTTATCATTTAAGAAATTCCATGATCCAG	pOPINF	*E.coli*
TRINITY_DN21159_c0_g1_i1	*Aphelandra squarrosa*	TTTATGAATTTTGCAGCTCGATGGATGCAACAAAAGCTGAGG	GACAACCACAACAAGCACCGTCAAGGATAGGCCTCAATGAC	3Ω1	*N. benthamiana*
TRINITY_DN6106_c0_g1_i1	*Aphelandra squarrosa*	TTTATGAATTTTGCAGCTCGATGGGCTCGACCAACAAGAAC	GACAACCACAACAAGCACCGTCATTTATATAACTCGATGATCCAAC	3Ω1	*N. benthamiana*
TRINITY_DN238_c0_g1_i2	*Aphelandra squarrosa*	TTTATGAATTTTGCAGCTCGATGAAAGAGAAGTTCTTCGGCAC	GACAACCACAACAAGCACCGTCAGGTGAGACGCTTGCAGAG	3Ω1	*N. benthamiana*
TRINITY_DN16034_c0_g1_i1	*Aphelandra squarrosa*	TTTATGAATTTTGCAGCTCGATGGAGTTCAAGGGGAACAAGAAC	GACAACCACAACAAGCACCGTCACAAACGAGAGATCAATAGAGG	3Ω1	*N. benthamiana*
TRINITY_DN15617_c1_g1_i4	*Aphelandra squarrosa*	TTTATGAATTTTGCAGCTCGATGGCTTCTCTTCATGCTCCTC	GACAACCACAACAAGCACCGTTAACTGGATTTCTGCGGCGG	3Ω1	*N. benthamiana*
TRINITY_DN1230_c0_g1_i1	*Aphelandra squarrosa*	TTTATGAATTTTGCAGCTCGATGGCTTCTCTTGGATGTCCTTC	GACAACCACAACAAGCACCGCTAAGATGCTGCCCCTGATCTG	3Ω1	*N. benthamiana*

TRINITY_DN15617_c0_g1_i1	*Aphelandra squarrosa*	TTTATGAATTTTGCAGCTCGATGGCATCCCTTCCTAATCTCC	GACAACCACAACAAGCACCGTCAGGAAGACGTGGCGGC	3Ω1	*N. benthamiana*
TRINITY_DN89858_c0_g1_i2	*Aphelandra squarrosa*	TTTATGAATTTTGCAGCTCGATGGCATCTACTCTCCTTCC	GACAACCACAACAAGCACCGTCAGACATCATCATACACAATTTTGATAC	3Ω1	*N. benthamiana*
TRINITY_DN4762_c0_g1_i3	*Aphelandra squarrosa*	TTTATGAATTTTGCAGCTCGATGGCTTCTTCCATCCCCTCC	GACAACCACAACAAGCACCGTTAGTCATCAAGCTCAATCTTGATGC	3Ω1	*N. benthamiana*
TRINITY_DN15617_c1_g1_i3	*Aphelandra squarrosa*	TTTATGAATTTTGCAGCTCGATGGCTTCTCTTCATGCTCCTC	GACAACCACAACAAGCACCGTTAACTGGATTTCTGCGGCG	3Ω1	*N. benthamiana*
TRINITY_DN15617_c1_g1_i1	*Aphelandra squarrosa*	TTTATGAATTTTGCAGCTCGATGGCTTCTCATCATCATGTTCC	GACAACCACAACAAGCACCGTTACCTGGATGGCGGCGG	3Ω1	*N. benthamiana*
TRINITY_DN12379_c0_g1_i2	*Aphelandra squarrosa*	TTTATGAATTTTGCAGCTCGATGGAGTTGGGCCACGTCC	GACAACCACAACAAGCACCGTCAACATCCAACGGCGCC	3Ω1	*N. benthamiana*
TRINITY_DN9860_c0_g1_i10	*Aphelandra squarrosa*	TTTATGAATTTTGCAGCTCGATGGATCCAGAAACCCCACAG	GACAACCACAACAAGCACCGTTAGCATGTTGCAGTTGATCCA	3Ω1	*N. benthamiana*
TRINITY_DN16027_c0_g1_i3	*Aphelandra squarrosa*	TTTATGAATTTTGCAGCTCGATGAAGCCGCATGCTGTCC	GACAACCACAACAAGCACCGCTAATTTTTAAGGAGAATCTCGTTG	3Ω1	*N. benthamiana*
TRINITY_DN1399_c1_g1_i2	*Aphelandra squarrosa*	TTTATGAATTTTGCAGCTCGATGGGGAGCGGTGGTGG	GACAACCACAACAAGCACCGCTAGTCTTGTTCATCATATCTTAC	3Ω1	*N. benthamiana*
TRINITY_DN334_c0_g1_i2	*Aphelandra squarrosa*	TTTATGAATTTTGCAGCTCGATGGCCGCCGCCGCTCT	GACAACCACAACAAGCACCGTTAATTCCTCGGTGCATTGGC	3Ω1	*N. benthamiana*
TRINITY_DN7511_c0_g1_i1	*Aphelandra squarrosa*	TTTATGAATTTTGCAGCTCGATGGCTGCCGCTCACCC	GACAACCACAACAAGCACCGCTATCTCCTAATATGGGCAACAA	3Ω1	*N. benthamiana*
TRINITY_DN3039_c0_g1_i1	*Aphelandra squarrosa*	TTTATGAATTTTGCAGCTCGATGGCTGAGAAGTCCTCCC	GACAACCACAACAAGCACCGTTATATTATGGTCATGGATTGGATG	3Ω1	*N. benthamiana*
TRINITY_DN2103_c0_g2_i4	*Aphelandra squarrosa*	TTTATGAATTTTGCAGCTCGATGGAGTCACAAGTGACCG	GACAACCACAACAAGCACCGTTATCTAGTGATATGTTTTATGAATGAATC	3Ω1	*N. benthamiana*
TRINITY_DN1286_c0_g1_i3	*Aphelandra squarrosa*	TTTATGAATTTTGCAGCTCGATGGAAGGTCCCGTAGATCTG	GACAACCACAACAAGCACCGCTAGGAACAACCAAAGCAAGTC	3Ω1	*N. benthamiana*
TRINITY_DN11457_c0_g1_i1	*Aphelandra squarrosa*	TTTATGAATTTTGCAGCTCGATGAATCCGAAACTTGACAGAC	GACAACCACAACAAGCACCGTCATTTGATACAGACACCATGC	3Ω1	*N. benthamiana*
TRINITY_DN334_c0_g1_i4	*Aphelandra squarrosa*	TTTATGAATTTTGCAGCTCGATGGCCGCCGCCGC	GACAACCACAACAAGCACCGTTAATTCCTCGGTGCATTGG	3Ω1	*N. benthamiana*
TRINITY_DN5184_c0_g1_i2	*Aphelandra squarrosa*	TTTATGAATTTTGCAGCTCGATGGATCCAGAAACCCCAC	GACAACCACAACAAGCACCGTTAAGTTGCAGTTGATCCATATTG	3Ω1	*N. benthamiana*
TRINITY_DN212_c0_g2_i1	*Aphelandra squarrosa*	TTTATGAATTTTGCAGCTCGATGGAAACTTTCACTGAGGATG	GACAACCACAACAAGCACCGCTACTCCTCAGCAAGTGGAAC	3Ω1	*N. benthamiana*
TRINITY_DN11987_c0_g1_i3	*Aphelandra squarrosa*	TTTATGAATTTTGCAGCTCGATGGAGGAGAAGATGAATTGTG	GACAACCACAACAAGCACCGCTAGTTGGACGAACACCAC	3Ω1	*N. benthamiana*
TRINITY_DN12379_c0_g1_i1	*Aphelandra squarrosa*	TTTATGAATTTTGCAGCTCGATGGAGTTGGGCCACGTC	GACAACCACAACAAGCACCGTCAACATCCAACGGCGCCAC	3Ω1	*N. benthamiana*
TRINITY_DN4525_c0_g2_i2	*Aphelandra squarrosa*	TTTATGAATTTTGCAGCTCGATGGCAGGGCAAGAGGCG	GACAACCACAACAAGCACCGTCACGCACTCTTGATGGTCTTC	3Ω1	*N. benthamiana*
TRINITY_DN40246_c0_g2_i1	*Aphelandra squarrosa*	TTTATGAATTTTGCAGCTCGATGGGGGATATGCCACGG	GACAACCACAACAAGCACCGTCAGGTTGCCAAGACACAC	3Ω1	*N. benthamiana*
TRINITY_DN41_c0_g1_i3	*Aphelandra squarrosa*	TTTATGAATTTTGCAGCTCGATGGCGAAGCAGAGAAAATTC	GACAACCACAACAAGCACCGTCAGTACTTCCAGTCATGGC	3Ω1	*N. benthamiana*

TRINITY_DN41_c0_g1_i4	*Aphelandra squarrosa*	TTTATGAATTTTGCAGCTCGATGGAGAAGCAGAGGAAG	GACAACCACAACAAGCACCGTCATGCCTTCGTTTTCTCCTTC	3Ω1	*N. benthamiana*
Contig_10649	*Lamium galeobdolon*	TTTATGAATTTTGCAGCTCGATGAGTTCATTTTCAAAAGCAGAAAGTAG	GACAACCACAACAAGCACCGTTATGGCAAAAGCTCCTTGATC	3Ω1	*N. benthamiana*
		AAGTTCTGTTTCAGGGCCCGATGAGTTCATTTTCAAAAGCAGAAAG	ATGGTCTAGAAAGCTTTATTATGGCAAAAGCTCCTTGATC	pOPINF	*E. coli*
Contig_6177	*Lamium galeobdolon*	TTTATGAATTTTGCAGCTCGATGGGTGGTGCAAGTGAAAGTG	GACAACCACAACAAGCACCGTTAATTCGTTTCATCGAAATTTGCC	3Ω1	*N. benthamiana*
Contig_3786	*Lamium galeobdolon*	TTTATGAATTTTGCAGCTCGATGGAAGAAGAAGAGGGGAG	GACAACCACAACAAGCACCGTCATTTTCTAAGATAATCAATGAATCTC	3Ω1	*N. benthamiana*
Contig_15618	*Lamium galeobdolon*	TTTATGAATTTTGCAGCTCGATGTCTCAAAACCACCCAATCAC	GACAACCACAACAAGCACCGCTAAGTGAATGAAGAAATATCTCTGAG	3Ω1	*N. benthamiana*
Contig_20398	*Lamium galeobdolon*	TTTATGAATTTTGCAGCTCGATGGCACCCAAAACCCTACAC	GACAACCACAACAAGCACCGTTAGCATCTTTGATGATTTTTTAGAAAATC	3Ω1	*N. benthamiana*
Contig_9495	*Lamium galeobdolon*	TTTATGAATTTTGCAGCTCGATGCCCATCCCTCGAGTCG	GACAACCACAACAAGCACCGTTAAGGTAGAAGAAGGGTGTGC	3Ω1	*N. benthamiana*
Contig_3120	*Lamium galeobdolon*	TTTATGAATTTTGCAGCTCGATGGTTACTCTTGCACTTTCATGG	GACAACCACAACAAGCACCGTCAATCGGAATCATAGATGATCTTG	3Ω1	*N. benthamiana*
Contig_1707	*Lamium galeobdolon*	TTTATGAATTTTGCAGCTCGATGGCTTCTCCCTTTATCTCAC	GACAACCACAACAAGCACCGTTAATCATCAAGCACAATCTTGACG	3Ω1	*N. benthamiana*
Contig_1193	*Lamium galeobdolon*	TTTATGAATTTTGCAGCTCGATGGCTTCTCTTCCCCTTTGTTC	GACAACCACAACAAGCACCGTTAATTTTCCGCCGCGTAATCAAC	3Ω1	*N. benthamiana*
contig_11686	*Lamium galeobdolon*	TTTATGAATTTTGCAGCTCGATGGCATCTCTTCCACTTCTCTG	GACAACCACAACAAGCACCGTTATGCTTTTTTGTTGGGTGGGTTC	3Ω1	*N. benthamiana*
Contig_14578	*Lamium galeobdolon*	TTTATGAATTTTGCAGCTCGATGGCTTCCCTTCAGTCTTCATG	GACAACCACAACAAGCACCGTCAGGGTGGCGCGGGAATG	3Ω1	*N. benthamiana*
Contig_22550	*Lamium galeobdolon*	TTTATGAATTTTGCAGCTCGATGGCTTCCCTTCAATCTTCATGC	GACAACCACAACAAGCACCGTCACGCCGATTTGGGCGGC	3Ω1	*N. benthamiana*
Contig_5273	*Lamium galeobdolon*	TTTATGAATTTTGCAGCTCGATGGACATGCAACTCCTCACC	GACAACCACAACAAGCACCGTCAAGTTGGGAACTTAAGATTCATTGG	3Ω1	*N. benthamiana*
Contig_32188	*Lamium galeobdolon*	TTTATGAATTTTGCAGCTCGATGGATATATTCCACCAAATACC	GACAACCACAACAAGCACCGTCAATTAGAAGGATTGTGTATAGTAG	3Ω1	*N. benthamiana*
Contig_28595	*Lamium galeobdolon*	TTTATGAATTTTGCAGCTCGATGGAGAGCTTGAGCACCC	GACAACCACAACAAGCACCGTCAGTTGGAAGAAGGATTGTATAGT	3Ω1	*N. benthamiana*
Contig_33465	*Lamium galeobdolon*	TTTATGAATTTTGCAGCTCGATGGAATTATCTGCACTTTACTTGC	GACAACCACAACAAGCACCGTTACAAGCTTGTTGATTTCTCTATG	3Ω1	*N. benthamiana*
Contig_31519	*Lamium galeobdolon*	TTTATGAATTTTGCAGCTCGATGTACCAGAAAGCCTACGACAAAC	GACAACCACAACAAGCACCGCTAGAGTTTGTTGACGATGAGAGG	3Ω1	*N. benthamiana*
Contig_31446	*Lamium galeobdolon*	TTTATGAATTTTGCAGCTCGATGAAAATGGCAAACCAAATACTCAAG	GACAACCACAACAAGCACCGTTAATTAGGTAGGTGATAGTGATAGG	3Ω1	*N. benthamiana*
Contig_14367	*Lamium galeobdolon*	TTTATGAATTTTGCAGCTCGATGGAGGTGATAGACTTCACCAC	GACAACCACAACAAGCACCGTCAAAGAGGCTGCTTATATGGTG	3Ω1	*N. benthamiana*
		CAACCCTCACTAAAGGGCATGGAGGTGATAGACTTCACCAC	CTCTGGCGAAGAATTGTTAATTCAAAGAGGCTGCTTATATGGTG	pESC-Leu	*S. cerevisiae*
Contig_30050	*Lamium galeobdolon*	TTTATGAATTTTGCAGCTCGATGGAGATTTCATGGTTACTATTAAC	GACAACCACAACAAGCACCGTCAATAAAGGTGGGGCGG	3Ω1	*N. benthamiana*

Contig_11095	*Lamium galeobdolon*	TTTATGAATTTTGCAGCTCGATGTTAATGGAGAAAAGAGTAGCC	GACAACCACAACAAGCACCGCTATAGTCCAACATAGTCAGCC	3Ω1	*N. benthamiana*
Contig_18238	*Lamium galeobdolon*	TTTATGAATTTTGCAGCTCGATGGAGAAAAGAGTGGCCATTG	GACAACCACAACAAGCACCGCTAGGAGTAGCCTATTACTGC	3Ω1	*N. benthamiana*
Contig_712	*Lamium galeobdolon*	TTTATGAATTTTGCAGCTCGATGGAGAAAAGAGTAGCCATTATTGG	GACAACCACAACAAGCACCGTCAGTTGCCATGCATTCCAAC	3Ω1	*N. benthamiana*
Contig_13076	*Lamium galeobdolon*	TTTATGAATTTTGCAGCTCGATGGAGATCATTTCTTGGTTTCTATTG	GACAACCACAACAAGCACCGTTAATAAAGGTGGGGTGGAAGTG	3Ω1	*N. benthamiana*
		CAACCCTCACTAAAGGGCATGGAGATCATTTCTTGGTTTCTATTG	CTCTGGCGAAGAATTGTTAATTTAATAAAGGTGGGGTGGAAGTG	pESC-Leu	*S. cerevisiae*
Contig_3786	*Lamium galeobdolon*	TTTATGAATTTTGCAGCTCGATGGAAGAAGAAGAGGGGAG	GACAACCACAACAAGCACCGTCATTTTCTAAGATAATCAATGAATCTC	3Ω1	*N. benthamiana*
Contig_15618	*Lamium galeobdolon*	TTTATGAATTTTGCAGCTCGATGTCTCAAAACCACCCAATCAC	GACAACCACAACAAGCACCGCTAAGTGAATGAAGAAATATCTCTGAG	3Ω1	*N. benthamiana*
Contig_20398	*Lamium galeobdolon*	TTTATGAATTTTGCAGCTCGATGGCACCCAAAACCCTACAC	GACAACCACAACAAGCACCGTTAGCATCTTTGATGATTTTTTAGAAAATC	3Ω1	*N. benthamiana*
Contig_9495	*Lamium galeobdolon*	TTTATGAATTTTGCAGCTCGATGCCCATCCCTCGAGTCG	GACAACCACAACAAGCACCGTTAAGGTAGAAGAAGGGTGTGC	3Ω1	*N. benthamiana*
TRINITY_DN1050_c0_g1_i3	*Acanthus ilicifolius*	TTTATGAATTTTGCAGCTCGATGGCGAAAAGAGTAGCCATC	GACAACCACAACAAGCACCGTCACTTGTTGAAATAACTCGAC	3Ω1	*N. benthamiana*
TRINITY_DN9057_c0_g1_i13	*Acanthus ilicifolius*	TTTATGAATTTTGCAGCTCGATGGAGTGGGTGTTGTGG	GACAACCACAACAAGCACCGTCAGTATAGGTGAGATGGG	3Ω1	*N. benthamiana*
TRINITY_DN1412_c0_g1_i3	*Acanthus ilicifolius*	TTTATGAATTTTGCAGCTCGATGGATCTGTTCACCGTCG	GACAACCACAACAAGCACCGCTAATCATCATTACTTGATTCTTG	3Ω1	*N. benthamiana*
Cluster-9204.7837	*Lamium maculatum*	TTTATGAATTTTGCAGCTCGATGGAGATCATTTCATGGTTAC	GACAACCACAACAAGCACCGTCAGTAAAGGTGGGGTGGAAG	3Ω1	*N. benthamiana*
Contig_33225	*Lamium galeobdolon*	TTTATGAATTTTGCAGCTCGATGGAGAAAAGAGTAGCCATTATTG	GACAACCACAACAAGCACCGTCAGTTGCCATGCATTCCAAC	3Ω1	*N. benthamiana*
Contig_675	*Lamium galeobdolon*	TTTATGAATTTTGCAGCTCGATGGAGATTTCATGGTTACTATTAAC	GACAACCACAACAAGCACCGTCAATAAAGGTGGGGCGG	3Ω1	*N. benthamiana*
Cluster-2496.6023	*Lamium album*	TTTATGAATTTTGCAGCTCGATGGAGAAAAGAATAGCCATTATTGG	GACAACCACAACAAGCACCGTTAGCTAGTTCCCATTCCAACATAG	3Ω1	*N. benthamiana*
LECA_c33258_g1_i2	*Leonurus cardiaca*	TTTATGAATTTTGCAGCTCGATGGAGAAAAACAGAGTAGCCATTATTG	GACAACCACAACAAGCACCGTTAGGGTAATCCATCATAGTCTTTG	3Ω1	*N. benthamiana*
PHFR_c68310_g1_i1	*Phlomis fruticosa*	TTTATGAATTTTGCAGCTCGATGGAGAAAAGAGTAGCCATTATTG	GACAACCACAACAAGCACCGTTAGGTTAATCCATTATAGTCTGC	3Ω1	*N. benthamiana*
ROOF_c40410_g1_i1	*Rosmarinus officinalis*	TTTATGAATTTTGCAGCTCGATGGAGAAACGAGTAGCCATC	GACAACCACAACAAGCACCGTCAGACTGCAGAATTGCTGG	3Ω1	*N. benthamiana*
MAVU_c26472_g1_i2	*Marrubium vulgare*	TTTATGAATTTTGCAGCTCGATGATGGAGAAAAGAGTGGG	GACAACCACAACAAGCACCGTCAGATCCCAGGAGCCATTC	3Ω1	*N. benthamiana*
Cluster-2218.4195	*Lamium orvala*	TTTATGAATTTTGCAGCTCGATGGAGATCATTTCCTGGTTAC	GACAACCACAACAAGCACCGTCAATAAAGGTGGGGTGAAAGTG	3Ω1	*N. benthamiana*
Cluster-9204.7837	*Lamium maculatum*	TTTATGAATTTTGCAGCTCGATGGAGATCATTTCATGGTTAC	GACAACCACAACAAGCACCGTCAGTAAAGGTGGGGTGGAAG	3Ω1	*N. benthamiana*
MAVU_c20842_g1_i1	*Marrubium vulgare*	TTTATGAATTTTGCAGCTCGATGGAGAATTCCCTTTCAATAC	GACAACCACAACAAGCACCGCTAGTAAAGGTAGTCGGGG	3Ω1	*N. benthamiana*

CYP92A46	*Scoparia dulcis*	TTTATGAATTTTGCAGCTCGATGGAGAGCTCCTCGGC	GACAACCACAACAAGCACCGCTAGTAAAGGTGGAGTGGAAG	3Ω1	*N. benthamiana*
Scaffold 2074295	*Wrightia religiosa*	TTTATGAATTTTGCAGCTCGATGGAAGTTTTAAATAGCACCAGC	GACAACCACAACAAGCACCGCTAATAGAGATGGAGAGGTAATC	3Ω1	*N. benthamiana*
LGBX5-like	*Lamium galeobdolon*	TTATGAATTTTGCAGATGGAGGTGATAGACTTCACCAC	GACAACCACAACAAGCACCGTCAAAGGGAGGGGTTGTATGGTG	3Ω1	*N. benthamiana*
		CAACCCTCACTAAAGGGCATGGAGGTGATAGACTTCACCAC	CTCTGGCGAAGAATTGTTAATTCAAAGGGAGGGGTTGTATGGTG	pESC-Leu	*S. cerevisiae*
LMBx5-like	*Lamium maculatum*	TTTATGAATTTTGCAGCTCGATGGAGGTGATAAACTTCACC	GACAACCACAACAAGCACCGTCAAAGAGGCTGCTCATATGG	3Ω1	*N. benthamiana*
		CAACCCTCACTAAAGGGCATGGAGGTGATAAACTTCACC	CTCTGGCGAAGAATTGTTAATTCAAAGAGGCTGCTCATATGG	pESC-Leu	*S. cerevisiae*
LOBx5-like	*Lamium orvala*	TTTATGAATTTTGCAGCTCGATGGAGGTGATAAACTTCACC	GACAACCACAACAAGCACCGTCAAAGAGGCTGCTCATATGG	3Ω1	*N. benthamiana*
		CAACCCTCACTAAAGGGCATGGAGGTGATAAACTTCACC	CTCTGGCGAAGAATTGTTAATTCAAAGAGGCTGCTCATATGG	pESC-Leu	*S. cerevisiae*
Zm_Bx1	*Zea mays*	TGTTGTTTTTATGAATTTTGCAGATGGCTTTCGCGCCCAAAA	CAGACAACCACAACAAGCTCATGGCAGCGCGTTCTT	3Ω1	*N. benthamiana*
Zm_Bx2	*Zea mays*	TGTTGTTTTTATGAATTTTGCAGATGGCTGCTCAACTGCATCA	CAGACAACCACAACAAGCTCACGCAGCCTGTGGGACTA	3Ω1	*N. benthamiana*
Zm_Bx3	*Zea mays*	TGTTGTTTTTATGAATTTTGCAGATGGCCCTTGGAGCTGC	CAGACAACCACAACAAGCTCAGGAAGCAATCCTTGGAACA	3Ω1	*N. benthamiana*
Zm_Bx4	*Zea mays*	TGTTGTTTTTATGAATTTTGCAGATGGCTCTCGAAGCAGCGTA	CAGACAACCACAACAAGCTCATTTGGGAATTCTAGGAACAAGG	3Ω1	*N. benthamiana*
Zm_Bx5	*Zea mays*	TGTTGTTTTTATGAATTTTGCAGATGGCACTCCAGGCAGC	CAGACAACCACAACAAGCCTAGACGGCCCTAGGAACAAG	3Ω1	*N. benthamiana*
Zm_Bx6	*Zea mays*	TTTATGAATTTTGCAGCTCGATGGCTCCAACGACCGCCAC	GACAACCACAACAAGCACCGCTAGAGCCTGAAGTGGTCGAG	3Ω1	*N. benthamiana*
Zm_Bx7	*Zea mays*	TTTATGAATTTTGCAGCTCGATGGGGCACCAGGCGCAG	GACAACCACAACAAGCACCGTCACGGGAAGACCTCGATGATG	3Ω1	*N. benthamiana*
Zm_Bx8	*Zea mays*	TTTATGAATTTTGCAGCTCGATGGCAGCATCGTGCGGC	GACAACCACAACAAGCACCGTCAGTAGGAGTTTATGAGATGAACC	3Ω1	*N. benthamiana*
EU747715	*Lamium galeobdolon*	TTTATGAATTTTGCAGCTCGATGGCTTCTTCTCTCAAGGCAAC	GACAACCACAACAAGCACCGTCAAGAAAGTGCAGATTTCAAACTTTTG	3Ω1	*N. benthamiana*
EU747716	*Lamium galeobdolon*	TTTATGAATTTTGCAGCTCGATGGCCGCTAATTCTCTCAAGTC	GACAACCACAACAAGCACCGTCAAACAAGTGCAGATTTTAAGGTTTTG	3Ω1	*N. benthamiana*
EU747711	*Aphelandra squarrosa*	TTTATGAATTTTGCAGCTCGATGGCTGCTGCTGCTCTCAAAG	GACAACCACAACAAGCACCGCTATAACAGAACTGAATCACCCCTC	3Ω1	*N. benthamiana*

Supplemental Table 3: Accession numbers (NCBI) for genes characterized in this study.

Gene name	Specie	NCBI Accession
AsFMO	*Aphelandra squarrosa*	OQ921353
AsBx4	*Aphelandra squarrosa*	OQ921354
AsBx5	*Aphelandra squarrosa*	OQ921355
AsBx6	*Aphelandra squarrosa*	OQ921356
AsBx7	*Aphelandra squarrosa*	OQ921357
AsPPO-1	*Aphelandra squarrosa*	OQ921358
AsPPO-2	*Lamium galeobdolon*	OQ921359
LgBx4	*Lamium galeobdolon*	OQ921360
LgFMO	*Lamium galeobdolon*	OQ921361
LgBx5	*Lamium galeobdolon*	OQ921362
LgBx8	*Lamium galeobdolon*	OQ921363
LgBx5-like	*Lamium galeobdolon*	OQ921364
LmBx5-like	*Lamium maculatum*	OQ921365
LoBx5-like	*Lamium orvala*	OQ921366
WrBx4	*Wrightia religiosa*	OQ921367
AiFMO	*Acanthus ilicifolius*	OQ921368
AiBx4	*Acanthus ilicifolius*	OQ921369
AiBx5	*Acanthus ilicifolius*	OQ921370
SiFMO	*Sesamum indicum*	OQ921371
MvFMO	*Marrubium vulgare*	OQ921372
RoFMO	*Rosmarinus officinalis*	OQ921373
PfFMO	*Phlomis fruticosa*	OQ921374
LcFMO	*Leonurus cardiaca*	OQ921375
LaFMO	*Lamium album*	OQ921376
LmFMO	*Lamium maculatum*	OQ921377
LgFMO_ctr33225	*Lamium galeobdolon*	OQ921378

Supplemental data set 1

Sequences used for FMO phylogeny

>Aphelandra squarrosa_FMO
MQMKKTVAIVGAGISGLLACKYALSIGLNPVVFEAESCVGGLWNHTIECTRLQSLKDYFQFSDFPWPSTIKTMFPKNEQVLEYVNSYADHFQLR
QYVRFNSQVMSIGYEGESKEEMGSWKSWGGTGMAFGSKGKWRLQVEAGGGMQEYHVADFVIVCIGKFSGLPAVPDFPPGSGPEVFSGKVL
HSMEYSDMDNASAAQFIKGKRIAVIGSGKSAIDIAFECGRANGRDTPCTVIHRNAHWMLPYEKPWLLAFALLCFTRFSELLVHKPGEGFFASVL
ATLLSPLRWLVSKFLEISLRWRLPLKKYDMIPSENFLEEANSCQVIMLQDNFFDKVVDGSIVFKKSEHFSFCKEGLIVEGEDSPVEVDIVIFATGY
KGDEKLKNIFASPAFQNHIFGSRNSTVPLFRQMIHPRIPQLAVVGYSESLSNLYTFEMRCRWLAFFLDRAFALPSIKEMEDDIKMWERYMKKYA
GNGEFKRGCIAGIHIWYNDQLCKDIGCSPRRKKGISSELFQPYGLEDYDGITPAQINPSYFAK
>Lamium galeobdolon_FMO
MLMEKRVAIIGAGISGLLACKYTASIGLKPIVFEEQDNVGGLWNHTIEITRLQQVKQSFQFSDFPWPSSADDNPKSAQLLEYLQSYAHNFDLLPFI
KFNSKVMNIDYVGESKEEMQSWDLWGGSGKAFGSKGRWILKVLHTKDDSIKVYESEFVVVCTGRFSGLPNIPEFAPGYGPEIFSGQALHSMD
YSNMDNASAAQFIKGKRIAIIGSGKSAFDIAFECANANGNDNPCTLVQRTIHWTFPSTKTWGLNFAFLIFTRFSELMVRKPGQGFVSSALTTLLT
PLRWGMSKYVESYLRWKLPLKKYGMVPKESFAAESSACQIFYLPRGNFYGKVEDGSIVLKKSQEFRFCKHGLIIDGDDKNPINADIVIFATGYK
GDEKLKSIFASPTFQNYIVGSPTSTVPLYRQMIQPRIPQLAVIGYSESFSNLFTFEMRCKWLAFFLDQAFELPSIRAMKKEVEAWETYMKRYAG
NNKFRRACIGRVHVWYNDQLCRDIGCNPRRKKGFLSELFEPYGSADYVGL
>Sesamum indicum_XM_011080249.2
MEKRVAIIGAGISGLLACKYAASRGFNPVVFEAEEQVGGLWNHTIESTRLQNIKDFFQFSDFPWPSSVQDALPTNAQLLEYLQSYARHFELLPYI
KFNSKVVSVSYVGESEEEMQSWELWGGSGKAFGSKGKWNLKVLHTKEESVEEYVAEFVVICIGRFSGLPEIPSFPPGYGPEVFSGKVLHSMD
YSAMDNASAAEFIKGKRIAVIGSGKSAIDVAFECAKANGNDIPCTVIQRTIHWMLPHDAQPWGVSLGFLCFSRFPELLVHKPGEGFLSSVLATSL
SPLRWGMSKFIESYLRWKLPLKKYNMIPKENVLQETSSCQILFLPPNFYDKVEDGSIVLKKPQHFSFCKEGLILDGEDDPVKADIVIYATGYKGD
EKLKNIFSSPTFQNCMAGSPTSAIPLYRQMIHPRIPQLAVISYSESLSNLYTFEMRCKWLGYFLDEAFKLPSIKEMEEDIKMWEKYMKRYSANGK
FRRACIGGVAIWYNDRLCKDIGCNPRRKKGFFADLFEPYGLADYKEL
>Sesamum indicum_XM_011080251.2
MRRISSIQAQATLKSSCKIQELLSSSMEKRVAIIGAGISGLLACKYAASRGFNPVVFEAEEQVGGLWNHTIESTRLQNIKDFFQFSDFPWPSSVQ
DAQPTNTQLLEYLQSYARHFELLPYIKFNSKVVSVSYVGECEEEIQSWELWGGSGKAFGSKGKWNLKVLHTKEESVEEYAAEFVVICIGRFSG
LPEIPSFPPGFGPEVFSGKVLHSMDYSAMDNASAAKFIKGKRIAIIGSGKSAIDIAFECAKANGNDIPCTVIQRTIHWMLPHDAQPLGLSFGFLCF
SRFAELLVHKPGEGFLSSILATSLSPLRWGMSKLIESYLRWKLPLKKYNMIPKEDVLQEKSSCQILFLPPNFYDKVEDGSIILKKSQCFSFCKEGL
ILDGEDDPVKADIVIYATGYKGDEKLKNIFSSPTFQNCIAGSPTSAIPLYRQMIHPRIPQLVVISYSESLSNLYTFEMRCKWLGYFLDEAFKLPSIKK
MEEDIKMWEKYMKKYSGNGKFRRACISGVPIWYNDQLCKDIGCNPRRKKGFFTDLFEPYRLADYKEL
>Coffea arabica_XR_003449871.1
MEKRIAIIGAGISGLLACKYALAKGYHPTVFEAQDCAGGLWNHTIESTKLQNPKEVFSFSDFPWPSSVKETFPRNTEVLKYVQSYAQHFGLLPYI
KFNTKVIGIDYVGVSREEMQAWDLWGGTGQAFGSKGKWNIKVQHTTEDKSIKEYEVEFVILCIGRFSGVPNIPEFPPNHGPEIFGGKVLHSMDY
SAMDNASAAALVEGKNIAIIGSEKSAVDLAAQCANVNGIDNPCTMVHRAAHWMLPSYYVWGGVRIDYLYYNRFAEILVHKPGEGFLHSALAFLL
SPLRWGIAKLVESYLRWKFPLKKYNMIPPHGFLQDVFSCEFLMLPENFFDRVVDGSIVLKKSKNISFSREGLMIDGETQPLKADVIILATGYRGD
QKLGNIFTCPSFQEYILGSTSSTIALYRQIIHPRIPQLAVIGYSESLNNLYTLEMKCRWLGHFLDQTFQLPSIKEMEKDVRMWDKYMKRYAGKYY
RRSCLGASHIWYNDQLCKDIGCNPRRKKGFFSELIEPYGPADYAELNPRS
>Aphelandra squarrosa_TRINITY_DN8200_c0_g1_i3
MEKRVAIIGAGISGLLACKYAVSVGLRPTVFEEQHHTGGLWNHTIESTRLQNGKEFFQFSDFPWPTSVEDLFPKNTQVLEYVQSYAHHFQLLQ
FIKFDSRVLSIDYVGESEEDMESWELWGGAGKAFGSKGKWNLKVFQTEEGGSIKEYVAEFVVLCIGRFSGLPAIPEVPPGHGPQVFSGKVLHS
LDYSAMDNAAAAQFIKGKRVAIIGSGKSAIDIAFECALANGSEKPCKVIQRTTHWMSPDAQPWGVSFGHLCFTRFSELMVHKPGEGFVTSVLAT
LLSPLRWGMSKFVESYLKWKLPLQKYAMIPEESFVQEASSCQIFFLPENFYDKVVDGSIVFKKSEHFSFCKEGLIIDGEDSPIEADIIIFATGYKGE
EKLKNIFASPTFQNYILGSPNTIVPLYRQMVHPRIPQLAVIGYSESLANLYTFEIRCKWLAFFLDQTFHFPSITEMEGDVKMWEKYMKKYAGNGK
FRRGCIGGVHIWYNDQLCKDIGCNPRRKKGVLSELFEPYGLADYSGISSGNRFMFNPPL
>Gmelina philippensis_GMPH_c34757_g4_i1_len_2278
MEKRVAIVGAGISGLLACKYTVSIGLKPVVFEAEDQVGGLWNHTIESTKLQVGREIFQFSDFPWPSSVGMLPSSSQLLEYLQSYAKKFELLQYIK
FNSKVINIDYVGESEEEMQCWELWGGAGKAFGSKGKWILKVLDTKEQSIKDYVAEFVVLCIGRFSGLPLCPEFPPGHGPEVFSGKVLHSMDFS
AMDNASAAELIKGKRIAIIGSGKSAVDIAFECANANGNDNPCTMIQRTIHWPLPDAKVWGISFRFLCFTRLSELVVHKPGEGFLSNVLATLLTPLQ
WAMSKFVESYVRWKLPLKKYGMIPKESFARDASSCQIFFLPENFYDKVEDGSIVFKKSQHFSFCREGLILDGEDNPLKADIVIFATGYKGDEKLK
NIFTSPTFQNYIAGSPTSTIPLYRQMIHPRIPQLAVIGYSESFSNLYTFEIRCKWLAYFLDQAFQLPGIKAMEKEIEMWERYMKRYAGNGQFRRA
CIGGVNIWYNDQLCKDIGCNPRRKKGFFSELFEPYGSGDYKELAPSSNYCNRSDI
>Ajuga reptans_AJRE_c45383_g1_i6_len_1829
MEKRVAIVGAGISGLLACKYTASKGFNPIVFEAQDNVGGLWNHTLESTRLQIVKETYQFSDFPWPSSADAFPPSAQLLDYFQSYANKFELFPYI
KFNSKVIGIDYVGESEEEMQSWQLWSGSGKAFGSKGKWILKVHDTKEDSIKEYEAEFVVICIGRFSGLPNIPEFPAGCSPEIFSGKVLHSLDYSL
MDNASAADLIKGKRVAIIGSAKTAMDIAFECANANGSEKPCTVIRRRNHWMLPDMQPWGVGFGFLCFTRFFELMVHKPGEGFLSSVLATLLSP
LRWAMSKFVESYARWKLPLKKYDLVPKESFVGEASSCQLFFLQDNFFDKVEDGSIVFKKSQSVRFCKDGLIIDGENDPVKADIVIFATGYKGDE
KLKNIFASPTFQNYILGSPNSIIPLYRQMINPRIPQLAVLGYSESLSNLNTFEMRCKWLVQFLDEAFQLPSVKDMEKEIEKWDKYMKKYAGNGKF
RRNCIGGLPVWYNDQLCKDLGCNPRRKKGFYAEFFEPYGLADYNGMAPGSN
>Cornutia pyramidata_COPY_c71621_g2_i5_len_1949
MEKRVAIVGAGISGLLACKYTVSIGLKPIVFEEQDQLGGLWNHTIESTRLQNVKESFQFSDFPWPSSDQDMFPHNTQVLEYLLSYAKKFELLQFI
KLNSKVINIDYVGESQEEMQSWDLWGGAGKAFGSKGKWILKVFDTKEESIKDYVAEFVVLCIGRFSGLPDIPDFPPGYGPEVFQGKVLHCMDY
SAMDNASAAEFIKEKRIAIVGSGKSAVDIAFECASANGNDNPCTMIQRTVHWTLPDVQPWGVNFGFFFANRFSELMLHKPGEGFLSSALATLLT
PLRWAMSKFVESYIRWKLPLKRYNMIPKESFVGEASSCQIILLPETFYDKVEDGSIVLKKSQHLRFCEEGLILDGEDNPLKADVVILATGYKGDK
KLKNIFASPTFQNCIEGSPTSIIPLYRQMIHPRIPQLAVIDYSESFSNLYTFEIRCKWLTFFLDQAIQLPRIKAMEEDVEMWEKYMKRYAGNRKFR
RGCIGSVHIWYNDQLCKDIGCNPRRKKGFLSDLFELYGMADYNALAPEL
>Congea tomentosa_COTO_c44193_g1_i1_len_1942

MEKRVAIVGAGISGLLACKHTASKGFKPVVFEEGDQIGGLWNRTSESTRLQVGREVYQFSDFPWPSSVDMLPTSNQLLDYLQSYAKRFELLPY
IKFNSKVININYVGESEEEMQSWELWGGAGKAFESKGKWILKVLDTKESSMKEYEAEFVVLCIGRFSGLPNNPEFPPGCGPEVFSGEVLHSMD
YSAMDNASAAELIKGKRVAIIGSGKSAVDTAFECANANGKDNPCTIVQRTIHWMLPDPHPWGVSFAFLCFTRFAELLVHKPGEGFFSSALATLL
TPLGWAMSKLIESYIRWKLPLKKYDMIPKESFVRDASSCQICFLPENFYDKVEDGIIVLKKSQHFKFCKQGLILDGEDNPVKADIVIFATGYKGDE
KLKNMFASKTFQNYIAGSPASIVPLYRQMIHGRIPQLAVIGYSESFSNLYTSEIRCKWLAYFLDQAFQLPSIKTMNKEIEMWEKYMKRYAGNGLF
RRACIGGVQIWYNDQLCRDIGCNPRRKKGFFSELFEPYGPLDYEDLAPEL
>Clerodendrum bungei_CLBU_c52150_g1_i2_len_2070
MEKRVAIVGAGISGLLACKYTVSIGLKPIVFEAQDHVGGLWNHTIESTRLQNFKFSFQFSDFAWPSSVEDMFPCSTQLVEYLQSYAQKFELLHYI
KFNCKVIDIDYVGESEEEMLSWELWGGAGKAFGSKGKWILRVLDTIGDSIKEYEAEFVILCIGRFSGLPNIPKFSPGCGPEIFSGKVLHSMDYAS
MDNASAAELIKGKRVTIIGSGRSAIDIAFECANVNGNDNPCTIIQRTTHWMLPDAHLWGVSFGFLFFTRFSELMVHKPGEGFFRSVLATLLTPLR
WGMSMFIERYLRWKLPLKKYGMIPKESFLGEASSCQLFFLPANFYDKVEEGSIVFKKSSNVGFCKEGLIINGDDNHVKADIVIFATGYKGDEKLK
NMFSCRTFQNCIIGSPTSTIPLYRQMIHPRIPQLAVIGYSESFSNLYTFEMRCKWLGFFLDQAFQLPCIKAMEKDIDKWEKHMKRYAGNGQFRR
SCIAGVHIWYNDQLCKDIGSNPRRKKGFFSELFEPYGLRDYNGLSL
>Leonurus cardiaca_LECA_c33258_g1_i2_len_1832
MEKNRVAIIGAGISGLLACKYIASIGLNPIVLEAQHNIGGLWNHTIESTRLQQNKQTFQFSDFPWPSSADDNPNSAQLLEYLQSYAQNFDLLRYV
KFDSKVMSVDYVGESQEEMQSWELWGGAGKAFGSKGRWILNVLDAKDNSIKEYEAEFLVLCIGRFSGLPNIPEFAPGYGPEIFSGQVLHSMD
YSAMDNATAAQLIKGKRIAIIGSGKSAFDIAFECANANGNDNPCTLVQRTIHWAFPDARPWGVDYGFLVLNRFSELMVHKPGEGILSSVLATLLT
PLRWGMSKFVESYLRWKLPLKKYGMIPKERFVAGASACQIFFLPHENFYDKVEDGSIVLRKSQQFSFCKEGLIINGDPINADIVIFATGYKGDEK
LKNMFASPSFQNYIIGSPNSAVPLYRQMIQPRIPQVAVIGYSESFSNLFTFEMRCKWLAFFLDQAFELPSIRAMETDIEAWEKYMKRYAGNHKF
RRACIGCVHVWYNDQLCRDIGCNSRRKKGLFSELFQPYGSKDYDGLP
>Holmskioldia sanguinea_HOSA_c50653_g1_i1_len_2042
MEKRVVIVGAGISGLLACKHTAFNGFKPIVFEVQDRIGGLWNHTIECTRLQVGREVFQFSDFPWPSSVDMFPHSTQLVEYLQSYAHKFELLPYI
KFNSKVISIDYVGESEKEMQSWELWGGDGKAFGSKGRWILKVFDTKEETVKEYEAEFVVLCIGRFSGFPNIPEFPPGYGPEIFSGKVLHSLDYS
AMDNASAAELIKGKRVAIIGSGKSATDIAFECAKTNVLSSGNDKPCTMIQRTIHWALPDPHPWGISFAFLCFTRFSELMVHKPGEGILASVLATLL
TPLGWVMSKFIESYIRWKLPLKKYNMIPKESFVRDASACQIFFLPDQNFYDKVEDGSIVFKKSQQFSFCKEGLMIDGEDNPLKADIVIFATGYKG
DEKLKNMFASPTFQNYIAGSPTSTVPLHREMIHPRIPQLAVIGYSESFSNLYTSEMRCKWLAHFLDQAFKLPSIKAMEKEIEMWEKYLKRYAGN
GQFRRACVGGVQIWYNDQLCKDIGCNPRRKKGFFSELFEPYLSVDYKGLAPEC
>Agastache foeniculum_AGFO_c16629_g1_i1_len_1805
MEKRVAIIGGGISGLLACKYAAAKGFAPTVFEEQDNVGGLWNHTLESTVLQVAKEFYQFSDFPWPDSADMFPHNTQLAQYLQSYAHKFQLLQ
FVNLNSKVMNINYVGESEQEMQSWDSWGGVGKAFGSKGKWILRVLHKEDDSIKEYEAEFLVLCIGRFSGLPNKPEFELGYGPEEFSGQVLHS
MDYANMENAAAADLIKGKRVVVIGSGKSAVDIAFECANVNGKDNPCTMIQRTVHWMLPHDSQPWGLSFGFLCFTRFAELMVHKPGESFLSSA
AATVLAPLRWGLNKLVEIYVKWRLPLKKYDMIPTESFVGEASSCQILFLQPHFYDKVEDGSILLRKSHNFTFCKEGVILDGEGTPLQADVVILATG
YKGDEKLKNMFASPTFQDYIEGSPTSILPLYRQMIHPRIPQLAVIGYSEALSNIFTFEMRCKWLAQFLDESFRLPSIGAMEKEIEMWDKYMKRYA
GNGKFRRSCIGGVHIWYNDQICRDIGSTPRRKKGFFSELFEPYGPADYHGL
>Lamium album_LAAL_c33795_g1_i2_len_1839
MEKRVAIIGAGISGLLACKHTVSKGFKPVVFEAQHCIGGLWNHTIESTRLQVGRQVFQFSDFPWPSSADKIPHSSQLVEYLQSYAQNFDLLPYIK
FNSKVMSIDYVGESEQEMGSWDLWGGSGKAFGSKGKWILKVFDTKEDSTKEYEAEFVIMCIGRFSGLPNIPQFSPECGPEIFSGKVLHSMDYS
AMENQDAAQFIKGKRIVIIGSGKSATDIAFECANANGNDKPCTMIQRSAHWGLPNLYPLGIEFSFMFFTRFSELMVHKPGEGFLANVLATLLTPL
GWLVSKLIESYIRWKLPLKKYGMIPKESFARDGSSCQILLLPHENFYDKVEDGSIVLRKSQQFSFCKQGLVIDGDDKNPINADIVIFATGYKGDEK
LKNIFASPTFQTYITGSPTSILPLYRQMIHPRIPQLAVIGYSESFSNLYTSEIRSIWLAHFLDEAFQFPSIKAMEEEIETWEKYMKTYAGNGGFRRA
CIAGVQVWYNDQLCIDIGCNPRRKKGFFSELFEPYISVDYKDLAPN
>Teucrium canadense_TECA_c6261_g1_i1_len_1861
MERRVAIIGAGISGLLACKYSVSIGLTPVVFEAEDVVGGLWNRTTESTRLQNVRQTFQFSDHPWPPSVEDMFPRSAQLVEYLQSYAHKFQLFP
YVRFNSKVVSVDYVGESEEEMQSWEMWGGDGKAFGSKGKWLLKVLDTKENSTEEYVAEFIVLCIGRFSGLPIIPEFSPGCGPEVFSGKVLHS
MDYAAMDNASAAELIKGKRIAIIGSGRSAVDITFECANANGNDNPCTMIQRTTHWALPDPKPWGVNFGYLCFTRFSELMVHKPGEGILDCILAT
LLTPLRWGMSKFVESYLWWKLPLKKYDMIPKESFVREASSCQTFFLPENFYDKVEDGSIMIKKSKQFSFCKEGLMLEGEDNPVKADIVIFATGY
KGDEKIKNMFASPKLQNYIAGSTSSIIPLYRQMIHPIIPQLAVIGYSESFSNLNTFELRCKWLAFFLDQAFQLPSIRVMEKEIQTWEKYMKRYAGN
GKFRRACIGGIHIWYNDQLCKDMGYSPRRKKGFFSELFEPYGSADYKGLDPRI
>Tectona grandis_TEGR_c41247_g1_i3_len_1839
MGKRVAIVGAGIGGLLACKYTASIGLKPILFEEQDQIGGLWNHTIESTRLQVGREVFQFSDFPWPSSADKIPHSPQLLEYLRSYAHNFELLQYIKF
NSKVINIDYVGESEEEMQCWELWGGVGKAFESKGKWILKVFHTKEDTTEEYEAEFVVLCIGRFSGLPDIPELSPGCGPEIFSGEVLHSMDYSA
MDNATAAELIQGKRVAIIGSGKSAIDIAFECAKANGNDKPCTVIQRTIHWMLPDIKVWGVSYEFLCFTRVIELMVHKPGEGFLSSVVATLLTPLRW
AASKFVESYARWKLPLRKYNMIPKESFVRDASSCQAFFLPHENFYDKVEDGSIVLRKSQHFRFCKEGLILDGEDDPLKADIVIFATGYKGDEKLK
NMFASPTLQNYIAGTPTSTIPLYRQMIHPRIPQLAAIGYSESLSNLYTFEIRCKWLAYFLDQVFHLPRIKAMEKEIETWEKYMKRYTGNSMFRRS
CIGLIHIWYNDQLCKDIGCNPRRKKGFFAELFKPYGSVDYKELAPGL
>Rosmarinus officinalis_ROOF_c40410_g1_i1_len_1849
MEKRVAIVGAGISGLLACKYAVARGFTPIVFEEQDHVGGLWNHTLESTTLQVPRQLYEFSDFPWPSSIEEMFPHNTQLTEYLRSYAQKFQLLQ
YVNFKCKVIDVDYVGESEDEIKCWDLWGGDGKAFGSKGKWILRVSHAADESIKEHEAEFLVLCIGRFSGLPNIPEFATGYGSDLFSGEVMHSM
DFANMENAAAAQFIKGKRVVVIGSGKSAVDIAYECANVNGRDNPCTMIQRTIHWMLPHDAKPWGLSLGFLCFTRFAELMVHKPCEGFLYSAAA
TMMSPLRWVVNKLVESYLKWRLPLKKYDMVPTESFVGEASSCQILFLQPNFYDRVEDGSIVMRKSQNFTFCKEGVILDGEGVPLQADIVILATG
YKGDEKLKNLFASPTFQNYIFGSPTSTIPLYRQMIHPRIPQLAVIGYSESLSNIFTFEMRSIWLSHFLDGAFALPSIGAMEKEIETWERYLKRYSGN
NSFRRSCIGGVPIWYNDQICRDIGSTPRRKKGFFSELFEPYGPADYQGL
>Salvia hispanica_SAHI_c28219_g1_i1_len_1864
MDEKRVAIVGAGISGLLACKYAAAKGFTPVVLEEQDKVGGLWNHTLESTTLQVPKQLYEFSDFPWPSSVQELRPHNTQLVEYLQSYAHKFQLL
HYVKFNTKVIDVDYVGGSKEEMNCWGGDGKAFASKGKWILRVSHSGDHHDSIKEYEAEFLVLCIGKFSGLPNIPDFAPGYGSDAFSGKVMHS
MDLAKMENAAAAQLIKGKRIVIIGSGKSAVDIAYECANLNGRDNPCTMVQRRIHWMIPHDAQPWGLSFSFLFFTRFAELMLHKPGQGFFFTAAT
ILLTPLRWVVNKLVEIYLKWRLPIKKYDMVPTENFIGQASSCQIHFLQPNFYDRVEDGSILMKKSKTFTFCKEGLILDGEALPLHADVVIFATGYK
GKEKLKNMFASPTYQNYIFGSPNSIVPLYRQMIHPRIPQLAVIGYSESLSCLFTFEMRCKWLSHFLDGEFGLPNIRDMEKEMEGWEKYMKRYA
GKGRSCIGGVPIWYNDQLCRDIGSTPKRKNGFVSELFQPYGPADYHHL
>Scutellaria baicalensis_SCBA_c33275_g1_i2_len_1802
MAKRVAIVGAGISGLLACKYVVSIGLNPIVFEADDEAGGLWNHTIESTRLQVGRHAYQFSDFPWPPSVQDMFPRSSQVVEYFNSYAHHFKLFP
YIKFNSKVMKIDYVGESEEEMQSWELWGGAGRAFGSKGKWILQVFHAKEDAMKEYEAEFVVVCIGRFSGYPNIPESSSGIFSGKVLHSSEYSA

MENASAREVIRGKRVVIVGSSKSAMDIAYECANVNGNEKGCTMIQRTVHWALPDPYPLGINFLFLFYIRFPELTIHKPGEGILAALLATLLTPLGW
LMFKLIEMYIRWKLPLKKYDMIPKESLLRDAASCRILFLPHPNFYDKVEDGSIVIKKSQHFSFCEEGLMIDGESDLLKADVVILATGFKGDEKLKNI
FASPTFQNYIAGSSPTSIIPLYRQMIHPRIPQLAVIGYSESFSNLYTSEIRCKWLARLLDQGFELPGMKEMKKEVETWEKFMKGYSSKGECRRA
CIGLVQIWYNDQLCKDIGINPRRKKGVLSELFQPYFAMDYKD
>Marrubium vulgare_MAVU_c26472_g1_i2_len_1907
MMEKRVGIVGAGISGLLACKYTASKGFNPIVFEEQQNVGGLWNHTLESTRLQIPKQTYQFSDFPWPSSADTFPHNTQVVDYFQSYALNFDLLP
YIKFNSKVMTIDYVGESKEEMQSWELWGGHGKAFGSKGKWLLNVLHSKDDSIKEFCLQEYEVEFLVLCIGRFSGLPNIPEFPPGCGPEIFSGKV
LHSLDFSTMDNASAAELIKGKRIAVIGSAKSAIDIAFECANTNGNDIPCTVIQRTNHWMLPDAQPWGVGFGFLCFTRFFELMVHKPGEGFFSSVL
ATLLSPLRWSMSKFVESYARWKLPLKKYGLIPKESFVGEASSCQLFFLQDNFFDKVEDGSILFKKSQCFSFCKEGLILDGEDDPVKADIVILATG
FKGDEKLKNMFANSTFQNYIVGSPNSIIPLYRQMIHPRIPQLAVIGYSESLSNLYTFEMRSKWLAFFLDQAFELPSIKAMDEEIKTWETYMKRYA
GNGKFRRACIGGISIWYNDQLCRDIGCNPRRKKGLFSELFEPYGLPDYDGMAPGI
>Phlomis fruticosa_PHFR_c67194_g1_i1_len_1785
MERRVAIVGAGIAGLVACKYTASQGFNPVVFEEQDNVGGLWNHTLQSTRLQIAKQTYQFSDFPWPPSADTFPHNTQVVDYLQAYAHKFHLSP
YIKFNSKVMSIDYVGESQQEMQTWELWGGSGKAFGSKGKWLLKVLHTKHHSIEEYEAEFIVVCTGRFSGLPNIPEFPKGCGPEIFSGKVLHSL
DYSSMDNASAAELIKGKRITIIGSAKSAIDIAYECASTNGNDIPCTVIRRTNHWMLPDAHPWGVGFDFLCFTRFFELMVHKPGEGFLLYVLAALLS
PLRWMTSKFVESYARWKLPLKKYDLIPKESFVGEASSCQLFFLQENFFDKVEDGSIVFKKSQHFSFCREGLVLDGEDNPIKTDIVILATGYKGDE
KLKNLFANPTFQNLIAGSPNCIIPLYRQMIHPRIPQLAVIGYSESLSNLYTFEMRSKWLAFYLDGAFELPSIKAMDKEIEMWETYMKRYAGNSKFR
RACIGGIPIWYHDQLCKDIGCNPRRKKRFLSELFEPYGLQDYDGIAPGS
>Perovskia atriplicifolia_PEAT_c40405_g1_i1_len_1929
MEKRVAIIGAGISGLLACKYSAARGFTPVVFEEQDHVGGLWNHTLESTTLQVPRQLYEFSDFPWPSSMEEMFPHNTQLAEYLQSYAQKFQLL
QYMNFKCKVIDVDYVGESKDEIKSWDLWGGDGKAFGSKGKWILRVAHAGDDSIKEYEAEFLVLCIGRFSGLPNIPEFAPGYGSDVFSGRVMH
SMDFANMENAAAAEFIKGKRIVVIGSGKSAVDLAYECANANGRDNPCTMIQRTIHWMLPHDAQPWGLSLGFLCFTRFAELMVHKPGEGFLSS
AAATLLTPLRWVVNKLVESYLKWRLPLKKYNMIPAENFVGEASSCQILFLQPNFYDKVEDGSIVMRKSQNFTFCKEGLILDGEDVPLQADVVIFA
TGYKGDEKLKNMFASPTLQNYIFGSPTSTIPLYRQMIHPRIPQLAVIGYSESLSNIFTFEMRCKWLSHFLDVAFTLPSIRAMEKEIEAWGRYMKR
YAGNDSFRRSCIGGVPIWYNDQICRDIGSTPRRKKGFFSELFEPYGPADYHGL
>Melissa officinalis_MEOF_c11019_g2_i1_len_1784
MEKRVAIVGAGISGLLACKYAAAKGFNPIVFEQQDHVGGVWNHTLESTALQVAKESYQFSDFPWPPSVQEMFPHNTQVTEYLQSYAEKFQLL
QFVNFNSKVIDVDYVGETKEEIEAWNFWGGDGKAFGSKGKWIIRVFHTQDDSIKEYEAEFLVVCTGRFSGLPNIPEFPPGYGSDVFSGKVVHS
LDYSNMENAAAAEFIKGKRVVIVGSGKSAVDIAFECANVNGRDNPCTMIQRTIHWMLPHDAQPWGLSFGFLCFTRFVELMIHKPGEGFLYSAA
ATLLSPLRWGLNKAVESYVKWRLPLKKYDMIPSETFVGEASSCQILFLQPHFYDKVEDGSIIMRKSRSFTFCKEGLILDGEGAPLQADVVILATG
YKGDEKLKNLFASPTFQNYIAGSPTSTIPLYRQIIHPKIPQMAVIGYSESLSNTFTFEMRCKWLAHFLDEGFKLPSIGAMEKEIEAWDKHIKRYSG
NGKFRRSCVGGVPIWYNDLICRDIGSKPRRKKGFFSELFQPYAPADYLGI
>Phlomis fruticosa_PHFR_c68310_g1_i1_len_1826
MEKRVAIIGAGISGLLACKYTASIGLKPMVLEEQDNVGGLWNHTIESTRLQQTKQSFEFSDFPWPSAADMNPRSSQLLEYLKSYAQNFDLLPYI
KFNSRVMNMDYVGESEEEMQSWELWGGSGKAFGSKGRWVLKVLHTKDDSVKEYEAEFVVLCIGKFSGLPNMPKFSPGHGPEIFSGQVLHS
MDYSAMDNVSAAELIKGKRIAIIGAGKSAVDITYECANANGNDNPCTLIQRTIHWMLPDARPWGVNYGFLVLNRFSELMIHKPGEGFLSSVMAT
LLTPLRWGVSKFVESYLRWRLPLKKYGMIPKQRFVTGASGCSIFFLPNENFYDKVEDGSIILRKSKCFTFCKQGLILDEENDPIKADIVIFATGYK
GDEKLKNMFASPSFQNYIAGSPNSAISLYRQMIQPRIPQVAMIGYSESFSNLFTFEMRCKWLSFFLDQAFELPSIKVMEKEAEVWEKYMERYA
GKHRRRRACIGCAHIWYTDQLCKDIGCNSRRKRGFFSELFEPYGIADYNGLT
>Origanum vulgare_ORVU_c49187_g2_i1_len_1842
MERRVAIVGAGISGLLACKYAAAKGFNPIVFEQQDNVGGLWNHTFETTTLQVPRVLYQFSDFPWPSSVQEMFPHNSQLSDYLQSYAQNFQIS
QYVNFNSKVIDVDYVGESKEEEKLSWESGDGKAFGSKGKWKLRVSHKGDDSVKEYEAEFLVLCIGRFSGLPNIPEFSPGSGPEVFSGRVLHS
VDYSNMENAEAKELIKGKKVVIIGSGKSAVDLAYECATVNGKENPCSMVQRTVHWMLPHDAKPFGISVGFLCFTRFAELMVHKPGEGFLTAVI
ATLLTPLRWAVNKMVETYLKWRLPLKKYGMVPAGSFVGEASSCQILFLQPQFYDKVEDGSILLKKSKSFTFCKEGVILDNGGGAPLQADVVILA
TGYKGDEKLKNMFASPTFQEYIEGSSSSIIPLYRQMIHPRIPQMAVIGYSESLSNIFTFEMRSKWLAEFLDGSFRLPSIKAMEKEIETWDKYMKR
YAGNNMFRRACVGGVPIWYNDQICRDIGSTPKRKKGFYSELFEPYGPADYLGL
>Mentha spicata_MESP_c44958_g1_i2_len_1833
MEKRVAIVGGGISGLLACKYAASKGFNPIVFEQQDNVGGLWNHTFETTTLQVPKVLYQFSDFPWPSSVEEMFPHNTQLSEYLQSYAQKFEILQ
YVNFNSKVINVDYVGGSKEEMPSWESGDGKAFGSKGKWILRVSHKGDDSVKEYEAEFLILCIGRFSGLTNVPEFSPGCGPEVFSGIVVHSMDY
SNMENAEATKLIKGKRVVIIGSGKSAADLAYECATVNGKGNPCRMVQRTIHWMLPHDAKPFGMSFGFLCFTRFAESMVHKPGEGFLTGVAAT
LLTPLRWAVNKMVESYLKWRLPLKKYGMIPTENFVGEASSCQILYLQPHFFDKVEDKSIILKKSKSFTFCEEGIILDAEGSTPLQADVVIFATGYK
GDEKLKNMFASPTFQEYIEGSSTSIIPLYRQMIHPRIPQMAVIGYSESLSNIFTFEMRSKWLAEFLDETFRLPSMKVMEKEIEMWEKYMKRYAGN
KMFRRACVGGVPIWYNDQICRDIGSTPKRKKGFYSEFFEPYGPADYLGL
>Monarda didyma_MODI_c39639_g1_i1_len_1716
MEKRVAIVGAGVSGLLACKYAASKGFNPIVFEQQDNVGGVWNHTFGTTTLQVPKALYQFSDFPWPSSVEEMYPHNTQLSQYLQSYAQKFEIL
QHVKFNSKVVNVDYVGGSKEDTLLSWESGDGKAFGSKGKWILRVSHKGDDSIKEYEAEFLVVCTGRFSGLPNIPEFSPGCGPEMFSGKVLHS
MDYSEMENAEAAEVIKGKRVVIIGSGKSAVDLAYECATVNGKGNPCTMVQRTIHWMLPHDAKPFGLSLGFLCFTRFAELMVHKPGEGFFTSVA
ATLLTPLRWAMNKLVETYLKWRLPLKKYDMIPTESFVREASSCRILYLPPHFYDKVEDSSIIFKKSNSFTFCKEGIVLDAEGSTPLQADVVIFATG
YKGDEKLKNLFASPTFQEYIEGSPSSIIPLYRQMVHPRIPQMAVIGYSESISNIFTFEMRSKWLAEYLDEGFRLPSIGAMEKEIAMWEKYMKRYG
GNKTVRRACIGGVPIWYNDQICRDIGSTPKRKKGFYLEFFEPYCPADYLGL
>Pogostemon cablin_POCA_c42188_g1_i1_len_1837
MKFIACQNQQLLSHLQRTHHLSLSLSSMEKRVAIVGAGISGHAACKHIASKGLKPMVFEAQDQIGGLWNQTLESTRLQVSRQLFQFSDFPWPS
SADVHPNSTQLVDYLQSYAQKFDLLQYMKFNCKVMNMEYVGEYEEEMQSWEMWGGSGKAFGSKGKWIIKVLHTNEDSIKDYEAEFVVLCVG
RFSGLPNIPECNPEKFSGKVLHSMDYSAMDNSSAAQFIKGKRVAIIGAGKSATDIAFECANANGNEKPCTMIQRSSSIAWALPNLNPWGISFGF
LCFTRFSELMIHKPGEGFLSAVMAWLLTPLAWFLSKFIEMYVKMKLPLKKHGMIPKESFALSASSCRIFILPDENFYDRVEDGSIVLKKSQHFSF
CREGLILDGEHDVLKADVVIFATGFKGDEKLKNIFSSPTFQNYIVGTPTSSVPLYREMIHPRIPGVVVIGYSESFSNLYTAEMKSIWLAHFLDGAF
RLPSIKAMEEDIKIWEKYMKRYGGKAHFRRSCIGNVHIWYNDQLCKDIGCNPRRKKGFFSELFEPYHAIDYKEVAPANYKYCK
>Verbena hastata_GCFE_scaffold_2056413
MEKRVAIIGAGISGLLACKYTASKGFNPVVFEAQDQVGGLWNHTLESTRLQNVKEFYQFSDFPWPSSVEDMFPKNTQLLEYLQSYAQHFELLP
YIKFNSKVMSIDYVGESEEEMQSWVLWGGVGKAFGSKGKWNLRVLDTKEDLVKEYAAEFVVICSGRFSGLPDIPEFPSGFGPEIFSGKVLHSM
DLSAMDNESAAAFIKGKKVAVIGSGKSAVDVAYECAKANGNNIPCTVVQRTVHWMLPDAQPWGVSFGFLCFSRFAELLVHKPGEGFLSSVLA
KLFSPLRWMISKFVESYVRWKLPLKKYGMIPKESFVQEASSCQITFLLENFYDKVEDGSIVFRKSQKFIFCKEGLIIDGEENPIKADIVIFATGYKG

DEKLKNIFASPTFQQCISGSPTSIIPLYRQMINPRIPQLAVIGYSESLSNLYTFEMRCKWLSFFLDQTFHLPSIKEMEEDITMWEEYMKKYAGNGK
FRRSCIGGIPIWYNDQLCKDIGCNPRRKKGFFSDLFQTYGLADYDGISPNHHF
>Ruellia brittoniana_AYIY_scaffold_2088915
MEKRVAVIGAGISGLLACKYAASKGFTPIVFEEQSQVGGLWNHTIEITRLQNVRYFFEFSDFPWPPSVQDLFPTSTQLLEYVQSYANHFQLLPYI
KFNSRVISVDYVGESEEEMSTWGLWGGSGKAFGSKGKWNLTVVDINDQSVKEYVAEFLVQCIGRFSGLPFIPENFSPGEGPQVFSGKVLHSM
DYSAMDNAAAAEFVRGKRVAIIGSGKSAVDLAFQCAMANGSDIPCTVIQRTIHWMFPDPEPWGVNFGYLCFTRFAELMVHKPGEGFFSSALAT
VLSPLRWAVSKFVESYLRWKLPLKKYDMIPKASFYDETSSCRILFLPENFYDKVEDGSIVFRKSEQFSFCKEGLMIEGQDEPIKADIVIYATGYKG
DEKLRDIFASPTFQNYILGSPKSILPLYRQIIHPRIPQLAVIGYSESLSNLYTFEMRCKWLSFFLEQTFNLPSIKEMEDDIDKWDKYMKKYAGNGH
FRRSCIVGVQIWYNDQLCKDIGCNPRRKKGFWSDLFEPYRLKDYDGITPHQI
>Gardenia jasminoides_CNA0013746_23071
MEKRIAIVGAGISGLLACKYALAKGYHPTVFEAQDSVGGLWNHTIESTKLQNPKELFLFSDFPWPPSVKEAFPCNTQVLDYVQSYAQHFGLLPY
IKFNSKVIGIDYVGVSPEEMQAWDLWGGTGQPFGSKGKWNVKVQHTIEDNSIKEYEVEFVILCIGRFSGVPNVPEFPPYGGPEIFGGKVLHSM
DYSALDNASAVALIKGKNIAIIGSQKSAVDLAAQCADVNGIDNPCTMVHRTAHWMLPSYYVWGGVRLDYLYLNRFAELLVHKPGEGFLHSALAF
LLSPLRWGISKLVESYLRWKLPLKKYNMIPPHSFLQDMSSCQILILPENFYDKVEEGSIVLKKSKKISFSSEGLMIDGEISQPLKADVIILATGYQG
DQKLKNIFTCPSFQKYMLGSTSSTISLYRQIIHPRIPQLAVIGYSESLNNLYTLEMKCRWLGHFLDQTFQLPSINEMEKDVGMWDKYMKRYAGK
YYRRSCVAVLHIWYNDQLCKDIGCNPRRKKGFFSELIEPYGPADYMELNPQS
>Rauvolfia tetraphyla_QEHE_scaffold_2013071
MEKRVAIVGAGISGLLACKYAIAKGLRPTVFEALANVGGLWNHTMESTRLQTPKQVFQFSDFPWPSSVKEDFPLDTQLLDYLQSYAEHFGLFP
YIKFNSQVIGIDYVGPSHEEMQSWELWGGIGKAFSSKGKWNIKVRHAEESSIKEYTAEFVILCTGRFSGLPNIPEFPQDRGPEVFDGKVLHSMD
YSAMDNAEAAELIRGKKVAIIGALKSAVDAAAICANINGSDNPCTVIARTPHWMCPSYNIWGVHLSWLYLNRFSELLVHKPGEGLLLSALASLLS
PLRWGISKFCESYLRWKLSLKKYDMIPQQSFLQDAVACTFLILPENFYNKVEEGSIRFKRSKSIGFCKEGVTLGGKAQTVKADIVILATGYKGNE
KLRNIFTSPTFQNYITSSPSSAIPLYRQMIFPRIPQLAAIGYSESLNNLYTFEMRCRWLAHFLDETFQLPSIKEMEEDVLMWEKYMKRYAGHYYG
RSCIGVLHICYNDQLCKDIGCNPRRKKGFISELFQPYGPADYLELTPNS
>Psychotria marginata_PCNH_scaffold_2072826
MENRIAIVGAGCSGLLACKHAVAKGFHPVVFEAQGCVGGVWNQTFESTQLQNTKDGYQFSDFPWPPSVQEMCPHNTQVLEYMQSYAQHFG
LIPYIKFNSKVIGIDYVGVPNEEMQAWDLWGGTGKPFGSKGKWNIKVQHADQEGSVKEYVVEFVILCIGRFSGLPYIPEFPEDHGPEVFAGKTL
HSMEYSAMDNAKAAELIKGKKIAIIGSQKSAVDLAAECADANGNDNPCTMVQRTVQWMLPSAHIWGVRIDSLYYKRISELWVHKPGEGFLDSAI
AFLLSPLRWGTEKFVESYLRWNLPLKKYKMIPSHGFFQALSSCQMLLLPENFYDKVENGSIVLKKSSSVSFCREGLIIDGEAQPLKADIVFLATG
YKGDEKLKNIFTSPTFQNYITGSPESTISLYRQILHPRIPQLSIIGYAESTNNLYTYEMKCQWLAHFLDQTFLLPSIKEMEKDAIEWNKYMKRYAG
KDYRRSCVGALYACHIDQLCKDIGCSPGRKKGI
>Wrightia natalensis_EDEQ_scaffold_2006749
MEKRVAIIGAGISGVLACKYALSKGFRPTVYEAQGKLGGLWNNTLKDTRLQNPKEVYQFSDFPWPASADPFPLDIQVLDYLQSYAEHFGLLPYI
KFHSKVLGIDYAGESHEEMQAWELWGGTGKAFGSNGKWHIKVQHTEDKSIKEYTAEFVILCIGRFSGLPNVPDFPQGCGPEVFHGEVLHSMD
FGAMDNDDAIELIRGKKVVIMGSQKSAIDVAALCATVNGTENPCTMIGRTPHWMLPGYNIWGVHLGFLYFNRFSELLVHKPGEGLFFGALASLF
APLRWGVSKFVESYLRWKLPLKKYDMIPPHSFLQEMSSCGILILPDHFYDKMEEGSIIFKKTESFEFCKQGLILSGEAEPLEADIVILATGYRGDQ
KLRNIFTSPTFQKHISNSPSYSIPLYRQIIHPRIPQLAAIGYSESLANLYTFEMRCKWLAYFLDGNIQLPSIRDMEKAALTWEKYMKRYSGPHYKR
SCVGALHICHNDHLCKDMGRNPKRKKGFVAELFEPYGPLDYSGLVP
>Catharanthus roseus_UOYN_scaffold_2005286
MEKRVAIIGAGISGLLACKLQTPKQVFEFSDFPWPSSVKEDFPLDTQLLDYLQSYAENFGLLPYIKFNSQVIGIDYEGESDLEMQSSELWAGTGK
AFGSKGKWKIEVQVQHDEHSSIQEYTAEFVILCIGRFSGLPSIPEFPQDHGPEVFYGKMLHSMDFSAMDNAEAVELIKGKKVAIVGAQKSALDV
AALCANVNGSDNPCTIIVRTPHWMLPSYYVWGVPLALLYLSRFSELLIHKPGEGLLFGALASLLSPLRWGIAKFCESYLRWKLPLKKYDMMPPH
SFLQDVSACTIVVLQDNFYSKVEEGCIRFKRSKSIGFCKEGLTLGGEGATQTVKADIVILATGYKGDEKLRNIFTSPTFQKCITGSSNSAIPLYRQ
MIHPRIPQLAAIGYSESLNNLYTTEMRCRWLVHFLDETIQLPSIKEMEKDVLMWENYMKSYAGNYYRRSCVGALHICYNDQLLKDIGCNPKRKK
GFIAELFQPYGPADYLELTSNS
>Lamium maculatum_Cluster-9204.5432
MVFEAQDNVGGLWNHTIESTRLQVGREVFQFSDFPWPSSADMIPHNSQLVEYLQSYAQNFDLLPYIKFNSKVMSIDYVGESEQEMQSWELW
GGAAKAFGSKGKWHLKVLDTKENSVKDYETEFVILCIGRFSGLPNIPQLSPECGPEIFSGKVMHSMDYSAMKNSDAAQFIKGKRIAIIGSGKSAT
DIAFECANANGNDKPCTMIQRNVHWGLPNLYPLGIKFTYVFFTRFPELMVHKPGEGFLANILATLLTPLNMLMSKLVESHIRWKLPLKKHGMIPK
ESIARDAASSCRALVLPDEKFYDKVEDGSIVLKKSQQFSFCKQGLIIDGDDKNPIYADIVIFATGYKGDEKLKNIFASPTFQTYITASPTSILPLYRQ
MIHPRIPQLAVIGYSESFSNLYTSEIRSIWLAHFLDEVFQFPSIKAMEEEIETWENYMKRYAGNGGFRRACIAGVQIWYNDQLCRDIGCNPRRKK
GFFSELFEPYTSVDYKDLAPNEPLIK
>Lamium orvala_TRINITY_DN6071_c0_g3_i2
MEKRVAIIGAGISGLLACKYTASIGLKPIVFEEQDNVGGLWNHTIESTRLQQIKHSFQFSDFPWPSSADDNPKSAQLVEYLQSYAQNFDLLPYIK
FNSRVMNIDYVGESKEEMQSWDLWGGAGKAFGSRGKWLLKVLDIKEGSVKEYEAEFVVLCTGRFSGLPNIPEFAPGYGPEMFSGQVLHSMD
YAAMENASAAEFIKGKRIAIIGSGKSAFDIAFECAKANGNDNPCTLIQRTINWTFPNAQPWGMNYNFLVLTRFSELMVHKPGEEFLYSALATLLT
PLRWGISKLVENYLRWKLPLKKYGMVPKESFVAGASACQIFLLPHENFYDKVEDGSILLKKSQQFRFCKQGLIIDGDEKNPINADIVIFATGYKG
DEKLKNIFASPTFQNYIAGSPTSTIPLYRQMIQPRIPQVAVIGYSESFSNLFTFEMRCKWLAFFLDQALELPSIASMEKEVEAWETYMKTYAGKH
KFRRACIGCVHVWYNDQLCRDIGCNPRRKKGFFSELFEPYGSADYVGMGTS
>Persicaria tinctoria_LC585869
MERKVGIIGAGISGLLACKHALSKGFHPVVLEAQPDIGGVWANALETTRLQTPKDFYQFSDFPWPSSVQDMHPTGEQVMEYIRSYADHFGLLK
HVRFNTKVLSISFEGTSDQEMEAWSHWNGTGEGFANKGKWTLLLQDVPTQSQQVMEFNFVILGIGKFSDRMRMPEFPAGEGPEVFDGKVIH
SKDYSEKSYGDARRLVKGKRVVVVGFQKSAIDIANECSSANGKEVPCNLIYRTPPWNIPDFFIWGLPLPYLYFNRFSELLLHKPGEGLFLSLLATI
LSPLRWLIAKFVESNIKYRHPLKKYGLVPEHGFGEAISSCVLSVLPKGFYENLEKGSILLNKAEKFRFCKEGIVMEGKPKPLEADLVIFATGFEAD
EKLRDIFASPKFQGHIMGSPHSILPLYRQCIHPQIPQLAIIGYSESFANLYTSEMRCRWIVELLDGTFKLPGFKEMEKETANWNKFIKGNAPKHYR
RSCIGQLHIWYNDQLCKDMGWNPRRKKGFISEWFQPHGPLDYIG
>Achantus ilicifolius_TRINITY_DN1050_c0_g1_i3
MAKRVAIIGAGISGLLACKYALSIGLDPVVFEAESYVGGLWNHTIECTRLQSHKDYFQFSDFPWPNSIKSNYPKNEQVLEYVNSYADHFNLFQYI
HFNSRVTSISYEGESKDEMAKWTSWGGAGTAFGSQGKWRLQVHVQPSGDFQFHDADFVIVCIGKFSGLPSVPEFPPGRGPEVFSGKVLHSM
DYSDMDNGSAAEFIKGKRVAVIGSGKSAIDIAYECGRANGSEIPCTVIHRNAHWMLPYDLPWVVAFGLLCFTRFSEMMVHKPGETFFATILATL
LLPLRWLMSKILEISLQWKLPLKKYDMIPKENFLEEANSCQVIMLQDNFFDWVVNESIVFKKIENFSFCKEGLIVDGEDKTIEVDIVIFATGYKGDE
KLKSIFASHTFQNNIFGLPNSTVPLYRQMINPRIPRLAVVGYSESLSNLYTFEMRCRWLAFFLSGAFPLPSIKEMEDDIRMWERYMKKYAGNGE
FKRGCIAGIHIWYNDQLCRDIGCNPMRKSGILAELFEPYGMADYDGITPARWMKSSYFNK

>Achantus ilicifolius_TRINITY_DN6034_c0_g1_i4
MAKRVGIVGAGMSGLLACKYAVSVGLRPMVFEAQQRVGGLWNQAMESTRLQIDKAHYQFSDFPWPSSVEGDFPKNTQVSEYAESYAHHFQ
LLDYVKFDSRVISIDYVGESHDEMERWELWGGSGKAFASKGKWILKVEQPGSVQEYEVEFVVLCTGRFSGLPDIPEFPPGHGPDVFTGEVIHS
MDYSAMDNARAAEFIKGKRIAVIGSGKSAIDLAFECACANGKSYRQFSCHCSKLKAYTFTSLPGRDQPCILIQRTSHWMFPSTRLWLFLYGFLF
FIRFSELMVHKPGEGLFFSILATVLSPLRWLLSNCIESGLRWVLPLKKFGMIPNESFVQEASSCQLLFLPENFFRKVEDGSIVVKKLQHFSFCKE
GLIIDGIKQPIEADIVIFATGYKGDEKLKNIFASPTFQSYIFGSPNSIFPLYRQMVHPLIPQLAVLGYSESLSNLYTFEIRSKWLAFFLDQAFHLPSIQ
QMEEEIKMWDRYMKKYAGNSKFRRNCIGGIHIWYNDQLCRDMGFNPRRKNGVFSELFETYGPEDYSEINPAQQIL
>Achantus leucostachyus_TRINITY_DN9077_c1_g1_i1
MATAKRVAIIGAGISGLLACKYALSIGLDPVVFEAESSVGGLWKHTIECTRLQSHKDYFQFSDFPWPDSIKSNFPKNEQVLEYINSYADHFKLVH
YVRFNSRVTSIAYEGESKDEMAAWTYWGGAGTAFGSQGKWRLQVDASSGDVQYHDADFVIVCIGKFSGLPSVPEFPPGRGPEVFSGKVLHS
MDYSDMDNASAAEFIKGKRIAVVGSGKSAIDIAFECGRANGGDKPCTVIHRNAHWMLPYDLPWVVAFGLLCFTRFSEMMVHKPGEGFFASILA
TLLLPLRWLMSKILEMSLEWKLPLKKYDMIPKERFLEEANSCQVIMLQDNFFDWVVNGSIIFKKIENFSFCKQGLIVEGEEKPIEVDVVIFATGYK
GDEKLKNIFASQTFQNSIFGLPNSTVPLYRQMINPRIPRLAVVGYSESLSNLYTFEMRCRWLAFFLSGAFPLPSIREMEDDIRMWERYMKKYAG
NGEFKRGCIAGIHIWYNDQLCKDIGCNPMRKNGFLAELFEPYGLADYDGITPPTWIT
>Achantus leucostachyus_TRINITY_DN3391_c0_g2_i5
MERRVGIIGAGISGLLACKYAVSVGLHPMVFEAQHHTGGLWNHTIQSTRLQNVKEFFQFSDFPWSSSVKDLFPTNTQVLEYVQSYAHHFQLLR
YIKFDSRVISIDYVGESEEDMENWELWGGAGKAFGSKGKWILRVIHAEDQASIKEYVVEFVVVCIGRFSGLPDIPEFPPGRGPEAFSGKVLHSL
DYSAMDNAAAAQFIKGKRIAIIGSGKSATDIAFECALANGSEKPCKVIQRTIHWMCPDAQPWGVSFGHLFFTRFTELMVHKPGEGFVTSAVATL
LSPLRWVMSKFVESYLRWKLPLQKYGMIPEESFVQEASSCQISFLADNFYDKVVDGSIVFKKSEHFSFCKEGLVIDGESNPIEADVIIFATGYKG
DEKLKNIIASPTFQKYILGSPNTIIPLYRQMVHPRIPQLAVIGYSESLSNLYTFEIRCKWLAFFLDQAFQFPSIKEMENEVKLWEKYMKKYSGNGK
FRRGCIGGVHIYYNDQLCKDIVCNPRRKKGVFSELFEPYGLADYNGIVPGKASSLMPL
>Achantus hungaricus_TRINITY_DN4324_c0_g1_i27
MAKRVGIVGAGLSGLLACKYAVSVGLRPVVFEAQQRVGGLWNHTLESTRLQIDKAHYRFSDFPWPSSVEGDFPTNTQVVEYAVSYARHFQLL
PYIKFGSRVTTIDYVGESHEEIESWESWGGSGEAFASKGKWILEVEEAGSVQEYEVEFVVLCIGRFSGLPDIPEFPPGHGPEVFAGKVLHSMD
YSDMDNARAAEFIEGKRIAVIGSSKSAIDVAFECASANGSEKPCTMIQRTSHWMKGLFSSLLASVLSPLRWGVSKCIEMCLRWVLPLKKFGMIP
NDSFVQEASSCQLLFLPEKFFNKVEDGSIVVKKSQFFSFCKEGLIIEGTEQPVEADIVVFATGYKGDEKLKNIFASPTFQSYIFGSPSSIVPLYRQ
MVHPRIPQLAVLGYSESLANLYMFEIRSKWLAFFLDQAFQLPSIQQMEEETKMWDRHMKKYAGNGMFRRNCIAGAHVWYNDQLCKDMGCNP
RRKNGMLSELFEPYGPEDYTGIDPAQHLL
>Aphelandra aurantiaca_TRINITY_DN13240_c0_g1_i3
MEMKKTVAIVGAGISGLLACKYALSIGLNPVVFEAESCVGGLWNHTIECTRLQSIKDYFQFSDFPWPSTIKTMFPKNEQVLEYVNSYADHFQLR
QYVTFNSKVMSIGYEGESKEEMGSWKSWGGTGMAFGSKGKWRLQVEAGGGMQEYHVADFVIVCIGKFSGLPAVPDFPPGRGPEVFPGKVL
HSMEYSDMDNASAAQFIKGKRIAVIGSGKSAIDIAFECGRANGSDTPCTVIHRNAHWMLPYEKPWLLAFALLCFTRFSELLVHKPGEGFFASVL
ATLLSPLRWLVSKFLEISLRWRLPLKKYDMIPSENFLEEAHSCQVIMLQDNFFNKVVDGSIVFKKSEHFSFCKEGLIVEGKDSPVEVDIVILATGY
KGDEKLKNIFASPTFQNHIFGSPNSTVPLFRQMIHPRIPQLAVVGYSESLSNLYTFEMRCRWLAFFLYRAFALPSIKEMEDDIKMWERYMKKYA
GNGEFKRGCIAGIHIWYNDQLCKDMGCSPRRKKGILSELFQPYGLEDYDGITPVRIHPSYFAK
>Lamium album_Cluster-2496.6023
MEKRIAIIGAGISGLLACKYTASIGLNPMVFEEQDNVGGLWNHTIESTRLQQIKQSFQFSDFPWPSCADDNPKSAQLVEYLQSYAQNFDLLPYIK
FNSRVMNIDYVGESKEEMQSWDLWGGSGKAFGSKGRWILKVVHTKDDDDSIKEYEAEFVVLCTGRFSGLPNIPEFAPGYGPEIFSGQVLHSM
DYSAMENGSAAEFIKGKRIAIIGSGKSAYDIAFECANANGNDNPCTLIQRTINWTFPDAKPWAMNYNFLVLTRFSELMIHKPGEGFLSRALATFL
TPLRWGISKSVESYLRWKLPLKKYGMVPKESFVAGASACQIFLLPRENFYDKVEDGSIVLKKSQQFRFCKQGLIIDGDEKNPINADIVIFATGYK
GDEKLKNIFASPTFQNYIVGSPTSTIPLYRQMIQPRIPQVAIIGYSESFSNLFTFEMRCKWLAFFLDQAFELPSIGSMEREVESWETYMKMYAGK
HKFRRACIGCVHVWYNDQLCRDIGCNPRRKKGFFSELFQPYGSADYVGMGTS
>Thunbergia alata_TRINITY_DN8382_c0_g1_i1
MYKTQIFQRVEINGGKPNQLLRINISLNTLESSFMEKRVAIIGAGISGLVACKHLASAGLRPLVFEAAAGIGGVWNRTMACTRLQSEKEFYCFSD
FPWPSSVPDLFPKNTQILDYIHSYAHHFDLFQYIRFNSTVTRIEYVGEDMPLWPSSGKAFASNGKWILHVLQQNSTKEYVFEFVVLCIGRFSGLA
DIPKFPPGCGPEVFTGEVLHSMDYTAMDHTDAARLVKGKRVAIIGSGKSAIDLAFECANANGNEKPCTVIQRSIHWMFLDAHAWGVPFSFLCFT
RLGELMLHKPGESFFSSLLATLLSPVRWGLSKFVESYLKWRFPLKKHNMIPKESLIQEVSSCQILFLPSENFYDKVEDGSIHLKRSNQIRFYKQG
LIIDDTTKVEADLVVFATGFKGDEKLKNIFASPTFRNIISGSPDSIVPLYRQMIHPRIPQLAVIGYSESLANLYTSELRSKWLAFFLDEAFSLPSIKEM
EEEIEEMEKFLKKYSGNGQFRRSCIASAQIWYNDQLCKDMGFNPNRKKGVLLNLFQPYGPSDYIDIDPAH
>Lamium galeobdolon_Contig_33225
MEKRVAIIGAGISGLLACKYTASIGLKPIVFEEQDNVGGLWNHTTESTRLQQIKQSFQFSDFPWPSSADDNPNSAQLLEYLQSYARNFDLLPYIK
FNSKVINIDYLGESKQEMQSWDLWGGSGKAFGSKGKWILKVLHTKDDSIKEYEAEFVVVCTGRFSGLPNIPEFAPGYGPEMFSGQVLHSMGY
SAMDNASAAEFIKGKRIVIIGSGKSAFDIAFECANANGNDNPSTLIQRTVHWTFPDAQPWGVNYAFFVLTRFSELMVHKPGEGFFSSALATLLTP
LRWGMSKFVESYLRWKLPLQKYGMVPKESFVAGASACQIFFLPDENFYDKVEDGSIVLKKSQQFRFCKHGLIIDGDDKNPINADIVIFATGYKG
DEKLKNIFTSLTFQNYIAGSPTSTVPLYRQMIPPRIPQVAVIGYSESFSNLFTFEMRCKWLAFFLDQAFELPSIRGMEKEVEAWETYMKRYTGNN
KFRRACIGCVHVWYNDQLCRDIGCNPRRKKGFFSELFEPYGSADYVGMHGN
>Lamium galeobdolon_Contig_712
MEKRVAIIGAGISGLLACKYTASIGLKPIVFEEQDNVGGLWNHTTESTRLQQIKQSFQFSDFPWPSSADDNPNSAQLLEYLQSYAQNFDLLPYIK
FNSKVINIDYVGESKQEMQSWDLWGGSGKAFGSKGKWILKVLHTKDDSIKEYEAEFVVVCTGRFSGLPNIPEFAPGYGPEIFSGQVLHSMGYS
AMDNASAAEFIKGKRIVIIGSGKSAFDIAFECANANGNDNPCTLIQRTIHWTFPDAQPWGVNYAFFVLTRFSELMVHKPGEGFFSSALATLLTPL
RWGMSKFVESYLRWKLPLQKYGMVPKESFVAGASACQIFFLPDENFYDKVEDGSIVLKKSQQFRFCKHGLIIDGDDKNPINSDIVIFATGYKGD
EKLKNIFTSLTFQNYIAGSPTSTVPLYRQMIPPRIPQVAVIGYSESFSNLFTFEMRCKWLAFFLDQAFELPSIRGMEKEVEAWETYMKRYTGNNK
FRRACIGCVHVWYNDQLCRDIGCNPRRKKGFFSELFEPYGSADYVGMHGN
>Anisacanthus_quadrifidas_PCGJ_scaffold_2003382
MYNTDHSTIFIMIAIIGAGISGLLACKYILSKGYNPIVFESQADIGGVWTKTIGTTKLQTPKSLFQFSDFPWPPTVDNFPTQQQVLQYLQSYAHHH
GLLPYINLNSRVLSLSYHGPISEEEMARWKWWGGTGEPFSPKGAKWKVTVQDTQTLSTQVHQVDFVIMCAGMYSGCPNIPEFPPGKGPKVF
QGKVIHSMDYSAMDSAAAAKLVKGKRVAVVGFQKSGMDISMECSSLNGTKHPCTLLYRTQHWNMPDHLPWGVPLAYLYQTRFSELLLHKPG
EGILLSLLATMFSPLRLGISKLVESHIKKKLKLTKHGMLPKHSFLREVNSCSIATVPESFYDGVEEGSIILKKSNQFSFCSEGVLIDDATEPLKIDLM
ILATGFRFVDKLKAAFASSPFHSLMDKDDTGLPLYRQCIHPRIPQLAFIGFSESIANLFTSEMTCRWLAELIDGTFKLPSIKEMEDDVCKWEKYKK
KSLGEHHSRSCLGAVQIWFNDQLCKDMGWKPMRKKGLIKELFEPYGPMDYA

Sequences used for BX4 phylogenetic analysis

>Sesamum_1 indicum_LC199945.1
MPLMENSSWALLALLHLAAALAFLSKIFLRPHPKRNFPPGPKPWPVIGNLNLVGSIPHQSLHFLSQKYGDIMQLKFGKFPVVVASSPEMAKQFL
RVHDTVFASRPALAAGKYTSYNYSDMTWAPYGPFWRQARKIYLSEVFSAKKLESLEHIRIEERHNFLARLYSLSGKPVVLREHLSRYTLSNISR
MVLSSKYFSESEHDKSVVKLDELQEMLDEWFLLNGVFNIGDWIPWLSFLDLQGYVKRMKALYKKFDRFHNYVIDDHQTRMATEKDSAPGDVV
DALLQLAEDPNLEVKLTRDGVKGLLQDLLAGGTDTSATTIEWAIHELLKHPHVIEKAKEELNRVIGRNRWVEENDFPNLPYIDAIIMESMRLHPLA
TFLAPHYAMEDCKVAGYDISKGTTILINTWSIGRDPNSWDAPNEFLPERFVGKEIDLTGSNFALLPFGSGRRRCPGYNLGLKIVRTILANLLHGF
DLKLVDGMRPEDICMKELYGLTTHPKKPLAIIMEPILPKNFY
>Sesamum_1 indicum_LC199946.1
MENSSWPLLALLHLAALAFLSKIVFRRHPKPNLPPGPKPWPVIGNLNLLGSIPHRSLHLLSQKYGDIMQLKFGKFPVVVASSPEMAKQFLKVHD
TVFASRPALAAGKYTSYNYSDMTWAPYGPFWRQARKIYLSEVFSAKKLESWEHIRIEERHNFLARLYSLSGKPVILRPHLYRYNLSSISRMVLS
SKYFSESEHDKSVVKLDELQEMVEEWFLLNGVFNIGDWIPWLSFLDLQGYVKRMKALQKKLDKFFSYVINDHQTRIATEKDPVQGDVVDALLQ
LAEDPNLEVKLTSDRIKGLLQGLVGAGTDTAATIIEWTIHELVKNPHVIEKGKEELDRVIGRNRWVEENDSSNLPYIDAIIMESMRLHPLSTILAPH
YAMEDCKVAGYDISKGTAILINTWSIGRDPNSWDAPNEFLPERFVGKEIDPTGSNFALLPFGSGRRRCPGYNLGLKLVRTTFANLLHGFNFKLV
DGMRPKDVCMEELYGLTAPPKEPLALIMEPILPKNFY
>Sesamum_1 indicum_LC199944.1
MPLMENSAWALLALLCLAALAFLIPIINFRQHPKRKFPPGPKPWPVIGNLHLVGSHPHRSFHVLSQKYGDIMQLKYGKFPVVVASSPEMAKQFL
RLHDKVFASRPALAAGKYTSFDYSDVTWAPYGPHWRQARKIYLSEVFNAKKLESFEYIRIEERRNLLSRLHSLSGKPVVLKDHLSRYSLSIISRM
VFGDGYISESGPNKSIVTLDELHELLDEWFLLNGVFNFGDWIPWLNFLDLQGCVKKMKGLYQKLDRFLNYVIDDHQTRRAIGKDSIQGDAVDML
LQLAEDDNLEIKLTRDRIKGLIQDLLGGGADTASTTLEWAIHEILRNPRVIEKAKVELDRVIGRNRWVEEEDTSKLPYIDGIIMESMRLHPLGTLLIP
HYAMEDCKVAGYDISKGTTILINTWSMGRNPNIWDAPEEFLPERFLDKDFDVTGGNFSLLPFGSGRRRCPGYNLGLKMVRPTLANLLHGFNW
NLVDGMRPEDVSVEEFYGLATQPKKPVTIIMNPALPDHLY
>Leonurus_japonicus_SNNC_scaffold_2006976_1
MEINSWLILALVYVASLALISKIIENRRPLNNRNLPPGPKPWPIIGNLNLLGSVPHQSLHNLSQKYGEIMLLKFGKFPVLVASSPYMAKQFLKVHD
ADFASRPLLAAGKYTSYNYSDMTWAPYGPYWRQARKIYLSEVFNAQKLDFFEQIRVQETRNFLSRLHPLTGKPVMLRDQLQRYTLSTISRMLS
RDVYFTSSEEDELRGMVDEWFFLGGVFNIGDWIPWLRFLDLQGYVKRMKILKKKLDRVFNYVIDEHTAKRAKDNDFAAKDIVDALLQISEDPNL
DVELTRDCVKALIQNLIVGATDTSATTIEWAINEVLRHPHIIKKAKEELDRIIGRNRWVQEADLSQLPYIESIIMETFRLHPLATFLAPHCAINDCNV
AGYDIAKGTTILINTWSIGRDSNSWDEPQKFLPERFMGKEVDALGSNFSLLPFGSGRRRCPGYKLALKMVPTAFANLLHGFNFKLVEGMRPQD
ICMEEEYGLTTHPRLPLSIIMEPTLPPHLY
>Uncarina_grandidieri_ZRIN_scaffold_2006324_1
MENSSWALLALLYLAALAFLSKIIFFRRHPKPKLPPGPKPWPIIGNLNLVGSIPHQSLHFLSQNYGDIMQLKFGKFPVVVASSPEMARQFLRVHD
TVFASRPALAAGKYTAYNYSDMTWAPYGPHWRQARKIYLTEVFNAKKLESFEHIRIEERRNFLSRLHSLSGKPVVLRDHLSRFTLSTISRMVLG
GKYFSESEDDKSIVKPDELQGMLDEWFLLNGVFNIGDWIPWLSFLDLQGYVKKMKALYKKLDRFHNYVIDDHQTRRATEKESISGDVVDALLQ
LADDPNLEVRLTRDCIKGLLQDLLAGGTDTSATTIEWAIHELLKNPRVIEKAKEELNGVIGRNRWVEEKDFSNLPYIDAIIMESVRLHPLATFLAPH
YAMEDCKVAGYDVSKGTTILINTWSIGRDPKSWDAPQEFLPERFVGKEIDMTGSNFALLPFGSGRRRCPGYSLGLKIVRTTLANLLHGFNLKLV
DGMRPEDICMEELYGLTTHPKEPLAIIMEPTLPDNFY
>Melissa_officinalis_TAGM_scaffold_2006990_1
MLLKFGKFPVVVASSPKMAEQFLKVHDAVFASRPPGLAAGKYISYDNSDISFSPYGPHWREARKILMSEVLSSKRLENFEHVRVEERRRFFSC
LKALSGKPVELREHLSRYILSSICRMVITDTYFSESEEESSIVDKYDLAEMMREWFVLNGVFNIGDWIPWLSSLDLQGYVKRMKALRKKTDRFF
GFVIDDHLARKDGSNKDFVDALLREAEDPNAEVEFTRDRMKSLIMNVLLGGPDTSITTIEWAMHEMLRQPHIIERATEELGRVIGRNRWVEEDD
FTQLPYIDAILMETFRLHPIATFLAPHYSMQDCNVAGYHIPKGTTILVNTWSIGRDLDAWDAAEEFLPERFLGKQTDMTGGNFALLPFGSGRRIC
PGFNLALKIVRTTLANLLHGFELKLVEGMKLEDISMEEQYGLATYPKHPVSIVLKPTLPDHLY
>Thymus_vulgaris_IYDF_scaffold_2077296_1
MEIQWLLLALISAAAAILISKFFETQQKLPPGPRPWPIIGNLNLLGSIPHQSLHHLSQKYGELMLLKFGKFPVLVASSPEMAKKFLKEHDAVFASR
PMFAAYKHVSYNYSDVSFSPYGPYWREVRKIFMTKVLSPRKLEESKRIRWDERITLLSRLKSETGKPVELRKHLSRFTLSTICKMVFKKKGLSE
REEDESMEELGEMVHEWFMLNGAFNIGDWVPWLDFLDLQGYVKKMKALRGKMDRFCDSVIRDHLAGTGAGEGEDFVDFLLQKVGESEGEV
EFTLDCVKALIMNMLVAASDTSATTVEWAIHEILRQPHMIEKAKAEINSVVGRNRWVEESDFTQFPYIEAIIMETMRLHPLATLLAPHCAIEDCNV
SGYNVSKGTVVLVNVWSIGRDPKSWDSPLEFKPERFLGKEIDMTGSDFTLLPFGSGRRRCPGYNLGLKIFRTTLANLLHGFELKFGEGMGAED
VSIEENYGLTTNPKHPVSIFMEPTLPHYLY
>Wrightia_religiosa_TRINITY_DN979_c0_g1_i1
MEVLNSTSLAHRFSIKMENSWLVLAFSLLAVLVFLSKLTNHSSRQKKKLPPGPKPWPIIGNLNLLGSIPHQSLHLLAEKYGDLMCLKFGSKPVLV
ASSPEMAKLFLRTHDNVFASRPPTASGKYTAYDYTDVTWAPYGPYWRHARRIYLNHIFNPKSLDSFEYLRVQERKALISGLHVLSGKPFVLRE
HLSRYSVSSLSRMVMGNKYFSETKVIRDESICTLEKLQDMLDQWFLLNGVFNIGDWIPSLSRFDLQGYVKQMKELYKNFEIFNNHVLDDHLTKR
NADKDNFIPKDMLDIILKIAEDPNLEIKLTRDNVKALLQELLVGGSDTSVTTVEWAMNELLRHPHLIKKATEELDRVIGRDRWVEEGDYSKLPFIE
AIIKETMRIHPLATLLAPHYAIEDCNVAGYDVSEGTTVLINIWSMGRNPKYWDSPEEFLPERFLEKDIDVKGQNNFVLIPFGSGRRRCPGYTLGL
RIVRATLANLLHGFNWKLPPGMKPEDVCVEELYGLTTRPKISLSVIVEPRLPLHLY
>Digitalis_purpurea_GNRI_scaffold_2033892_1
MENSWAVLALAVLVTLGLGFISKLILKHRHPKRRSPPGPIPWPVIGNLNLIGPTTHISLHALSQKYGEIMRLKFGKFPVVVASSPEMAKQFLKTHD
RVFASRPTGLAAGKYIGYNYSNMSWAPYGPYLRQARKIYLTEVFNTRALEFYEHTRTEERRNLFSRLNALSGEKVVMREHLSRYTLTMISRMV
FSNRYFGESDGEKSIMKLEEFQGMIDEWFSLNRAFNIGDWVTGLGCFDLQGYVKKMKALRMRFDDFFNHVIDDHRARRVADKDDFVPKDVLD
SLLHLADDPNLEVELTSDCIKGLLHDLLVGGTDTSTIEGAMLALIKQPCIMEKAKEELDRVIGRNRWVEEKDASQLPYINAIITETLRLHSNATFLG
PHFATEDCNVAGYDISKGTIVLVNTWSIGRDPNTWDAAEEFVPERFLGKEVDVLGNNFALLPFGAGRRMCPGYNLGFRINRTTVANLLHGFNW
KLHEGMMPEYASTLALVMEPTLSVPF
>Leonurus_1 cardiaca L._LECA_c30211_g1_i3_len_1627
MDINSWLLLALVYVASLALISKIIENQRPRNRNLPPGPKPWPIIGNLNLLGSIPHQSLHNLSQKYGELMLLKFGKFPVLVASSPYMAKQFLKVHDA
DFASRPLLAAGKYTSYNYSDMTWAPYGPYWRQTRKIYLSEVFNAQKLNFFEQIRVQETRNFLSRLRLSSGKPVTLRDQLQRYTLSTISRMLST
NVYFTSLEEDELRGMVDEWFFLGGVFNIGDWIPWLRFLDLQGYVKRMKELNKKLDRVYNYVIDEHLAKRAKDKDIVDAFLQISEDPNLDVELTR
DNVKALIQNLIVGATDTSATTIEWAINEVLMHPHIIKKAKEELDTIIGRNRWVQEADLSQLPYIESIIMETFRLHPLATFLAPHCAINDCNVAGYDIAK

GTTILINTWSIGRDPNSWDEPQKFLPERFMGKEIDALGSNFSFLPFGSGRRRCPGYKLALKMVPTTFANLLHGFDFKLIEGMEPQDICMKEEYG
LTTH
PKLPLSIIMEPTLPLHLY
>Clerodendrum_1 bungei Steud._CLBU_c53030_g1_i4_len_1907
MENSWIFLSFLSAAALIFFSKLITNQFQPKKTLPPGPKPWPIIGNLNLLGSLPHQSLHHLSRKYGEIMLLKFGKFPVVVASSPEMAKQFLRIHDTV
FASRPALASGYHLSFNYSDMSFSPYGPYWRQTRKIYLSEVFNAKKLDSFEHIRVDERRAFFSRLRGLSGKPIVLRDHLSRYALSTINRILTGEKY
FSESEEENSVVNMNELRGMVDEWFLLTGVFNIGDWIPWLAFLDLQGYVGKMKALRKKIDRFYDHVIDDHLARKGVEQGSEGKDLVDVLLEMA
EDPNIEVPLTRDGVKALIQNLFLGGTDTSTATVEWAILEILRQPHVMEKAKEELNKVIGRNKWVEENDFSELPYINAIIAETFRLHPLTTLLVPHYAI
EDCKVAGYDLSKGTTVLVNTWAMGRDPNVWDAHEEFLPERFLGKEIDLTGNNFAMLPFGSGRRRCPGYNLGLKMVQTTLANLLHGFELKLIE
GMGPKDICMEEEYGLTTHAKHPISIVMEPTLPLHLY
>Salvia_1 officinalis L._SAOF_c32330_g1_i1_len_1882
MLLKFGKFPVFVASSPEMAKQFLKVHDTVFASRPTFAAYKHISFDYSDVSFSPYGPYWRDARKIFMTKVLSARKVESFELIRVEERRCLVSRLS
SLSGKPVQLREHLLRLTLSTMCKMIFKEEYFKEKEDHGVGESILDIDQMQEMVREWFVLNGAFNIGDWIPWLDFLDLQGHVKKMKALRKKMDK
FCDHVIDDHVAKRVAGANKDFVDVLLQIAEDEDEVEFTRDSVKALIMNLLVAATDTSATAVEWAIHEMLKEAGTIEKAKAELNRVIGRDRWVEE
KDLTQLPYINAIVMETLRLHPIATLLAPHCAIEDCNVAGYDISKGTMVLINVWSMGRDPNLWDGPQQFLPERFVGKDIEMMTGSNFALLPFGSG
RRRCPGISLALKTVPTTLANLLHGFEVKLEEGMRVEDISMEEHYGLTTVPKHPLSIIMEPTLPNHLY
>Marrubium_1 vulgare L._MAVU_c20842_g1_i1_len_1608
MENSLSILVLLAALALFSKLFIQKNKKRPPPGPTPWPIIGNFNLIGPIPHQSLHSLSKKYGDLMQLKLGKFPVVVGSSPDMAKQLLRIHDAKFASR
PTLAAGKYTSYEYSDIALAPYGPYWRQARKIFHTEVLNETRLETSEHIRVDERRIFVSSLRALSGKPVVVRDHILGYTISSISRTLFSNKYYGESG
VVSLRELQGMIDEWLSLNSVSNIGDWVPWLRFVDLQGYVKRMKDLNNKFDRFNEYVIDDHLARRDFVAKDMVDVLLKLAEDPNRKVEMTRDN
MKGLLQGLLIAGADPSATTIEWTIHELLRHPRITKRAKEELDRVIGRKRWIEEKDLSQLPYINAIVMESLRLHPIGTLLIPHYAIEDCNVAGYDIAKG
TIVRVNVWSIGRDTRIWERAEDFLPERFLGKEIDLRGNDFSLLTFGSGRRRCPGYNLGLRVVRTTLANLLHGFELRLVEGMRPQDVCMEEMYG
LSTRP
KVPISVLMEPTLPDYLY
>Aphelandra_1 squarrosa_TRINITY_DN4049_c0_g1_i2
MFMNMVDFWALVLIFLLAYSAASFIFRKHPKHKLPPGPKPWPVIGNLNLIGSIPHQSLHLLSKRYGEIMLLRFGKFPVIVASTPEMAEQFLKTHDS
VCASRPALDAGKYTSYGYSDMVWAPYGPYWRQLRKLYFSEVFSAKMAQSKMFEQIRAEERRNLFSHLHSLSGNPVFLRGHLFRHMLSTISK
MVMSKTYYHEAGDENSSVVELKEFQGMLNEWFFLSGAFNIGDWIPWLNFLDLQGYVKRMKALHKKLDPIFDFVIDDHEGRRKGEKNFGSGKD
FVDKLLELAQDSNAEIKLSRNCVKALIQNLIVGGTDTAPTMVEWTIREVMKQPRLIEKAKEELDRVVGRKRWVEEDDCSQLPYIDAIIKESMRLH
PLATLLGPHYTLDDFSVTGYHVPKGSMVLINTWSIGRDPNSWDAPKVFFPERFMGKDIDVLGSNFSLLPFGSGRRRCPGYDLGLRTVRSIFAN
LLHGFNLKLVDGMRAEDIGMEEEYGLTTHPKEDLPVVMEPALPPHLY
>Lamium_1 galeobdolon_Contig_30050
MEINSWLLLALLYVASLALISKIIENRRPSNRNLPPGPKPWPIIGNLNLLGPIPHQSLHYLSQKYGEIMLLKFGKFPVLVASSPNMAKQFLKVHDAH
FASRPLLSAGKYTSYNYSDLVWAPHGPYWRQARKIFLHEVFSAKKLDFFAHIRAHEARSLLSRLLPLSGKAVVLRDELQRYTLSTISRMLSREC
FTSLEDDELRGMIAEWFFLTGVFNIGDWIPSLRFLDLQGYVKRMKVLHEKLDRVFNYVIDQHLMAKRDKGKDFTAKDIVDEFLQISEDPNLDVEL
TRDCVKALIQNLIVGGTDTSATTIEWAMNEVLRHPHVLEKAKEELNRIIEKNKWVQESDLSQLPYIESFIMETFRLHPVGTLLAPHCAINDCNVAG
YDIAKGTTVLINVWSIGRDPNLWDEPQKFLPERFMGKEIDAT
>Lamium_1 maculatum_Cluster-9204.7837
MEIISWLLLVLLYVASLTLISKILENRRPLNRNLPPGPKPWPIFGNLNLLGSLPHQSLHYLSQKYGEIMLLKFGKFPVIVASSPNMAKQFLKVHDAD
FASRPLLAAGKYVSYNYSDMSFAPYGLYWRQARKIFLSEVFNAKKLDLFEQIRVQEGLNLLSRLRNLSGNPVVLRDELQRYTLSTISRMLSRDV
YFTTGEDELRGMIDEWFFLTGVFNIGDWIPWLSFLDLQGYVKRMKVLNKKLDRVFDHVIDEHLAKRAKDKDFVAKDIVDVFLEISEDPNLDVELT
KDCVKALLQNLLVGGTDTSATTVEWAISEVLRHPHTIKKAKEELDEIIGRNRWVQDTDLSQLPYIESIIMETFRFHPLTTLLAPHCAINDCKVAGY
DITKGTMVLINTWSIGRDPNSWDAPEKFLPERFMGGKIDALGSNFSLLPFGSGRRRCPGYNLGLKMALTTFANLLHGFNFKLVEGMKTEDICIE
EEYGLTTHPKLPLSIIMEPALPPHLY
>Lamium_1 orvala_Cluster-2218.4195
MEINSWLLLVLVYVASVALISKILENRRLPNRNLPPGPKPWPIIGNLNLLGSLPHESLHYLSQKYGEIMLLKFGKFPVIVASSPNMAKQFLKVHDA
DFASRPLLAAGKYVSYNYSDMSLAPYGPYWRQACKIFLSEVFNAKKLDFFEQIRVQEGHNLLSHLRTLSGKPVVLRDELQRYTLATISRMLSRD
VYFTSEEDELRKMIDEWFFLTGVFNIGDWIPWLSFLDLQGYVKRMKVLSKKLDRVFNYVVDEHLAERAKDKDFEAKDIVDAFLQISEDPNLDVE
LTRDCVKALIQNLIVGGTDTSATTIEWAINEVLRHPHIIKKAKEELDGIIGRNRWVQETDLSQLPYIESIIMETFRLHPLATFLAPHCAINDCNVAGY
DIAKGTTILINTWSIGRDPNSWDAPEKFLPERFIGQEIDALGSNFSLLPFGPGRRRCPGYNLALKMLPTTFANLLHGFNFKLVGGMKPQDICMEE
EYGLTTHPK
>Aphelandra_1 aurantica_TRINITY_DN27487_c0_g1_i1
MGMMEGGLGLWWVLAVALAYVVASFFFNNGKKPKLPPGPKPWPIIGNLNLIGPIPHQSLHDLFQKYGELMHLKFGHFPVVVASTPEMAKQFLK
THDTAFASRPALDAGRLTSYNYTDLTWAPYGPYWRLARKIYVVDVFSAKKLDSDAFHQIRVEERQTFFSLMHSLAGKPVVLRGHLGRAAVITIS
RMVMGKAYFGEDESCIIGTDGFQELLDEWFFLNGVFSICDWIPWLRFIDPQGYVRRMKNLHKELDRIFDFVVADHEATRKKDSDSCSEKDVVY
KMLDMTEEPTLGVALSREHVKALLQNLILGGTDTTPTSVEWILCELMRNPHIVRKAKEELNRAVGRDRWVEESDFPKLPYLDAIIKETMRLHPIA
TFLAPHCAMEDCKVAGYDIPKGATVFINSWSLGRDPDSWDRPLEFLPERFMEKEIDIMGSNFAMLPFGAGRRRCPGYGHALKNIRAIVANLLH
GFELKLPNGMKPSDVDMEEEYGLTTHPKEPLALIMEPTLPPHLY
>Achantus_1 ilicifolius_TRINITY_DN9057_c0_g1_i13
MEWVLWWLLALPLAYIVSSLFSKPSHSKRAKLPPGPKPWPIIGNLNLIGSIPHQSLHQLFKKYGELMHLKFGQFPVVVASTPEMAKQFLKTHDT
AFASRPALDAGRYTSYNYTDLTWAPYGPYWRVARKIYVVDVFSSKKLDSEAFEKIRVEERRVFFSVMHKLAGKPVVLRGHLARAAVTTISRMV
MGKMYFGEEEGCIIGTDDFQELLDEWFFLNGVFSICDWIPWLRFLDPQGYVRRMKKLHKELDRIFDYVVADHEAQRMKAKESTCADKDVVYK
MLDMTEDPNLGFVLEREHVKALLQNLILGGTDTTPTSVEWILCELMRNPGIVGKAKEELDRAVGRDRWVEESDFPNLPYLDAIIKEQMRLHPIA
TFLAPHCAMEDCNVAGYDIPKGTTILINSWSLGRDPNYWENPDDFLPDRFIQNDIDIMGSNFAMLPFGSGRRRCPGYGHALKNIRALVANMLH
GFELSLPNGMKPQDVDMEEEYGLTTHPKHPLALLMNPTLPSHLY
>Achantus_leucostachyus_TRINITY_DN3801_c0_g1_i1_1
MEGVLWILALALTYVLASFVKKPKLPPGPKPWPIIGNLNLISSIPHQSLHHLFKKYGQLMHLKFGKFPVVVASTPEMAKLFLKTHDTVFASRPALD
AGRYMSYNYRDLGWAPYGPYWRTARKVYAVDVFSPKNLNSEAFQKIRVEERQVFFSVMHKLAGKPVELRGHLFRAALTTMSRMVMGKMYF
GEEEGCIIGMEEFQKLLDEWFFLSGVFNICDWIPWLRFLDPQGYVRRMKKVHKELDRIFGFVVADHQAQRIKDRESDVVHKILDMTEDPNLGF
PLERDHVKALLQNLILGADTTPTAVEWILCELMRNPRIIQKAKEELDRIVGRDRWVEESDFPKLPYLDAIIKEQMRLHPIATLLAPHFAMEDCNVA
GYDIPKGATILINSWSLGRDPNSWDTPEEFWPERFIEKDIDIMGSNFAMLPFGAGRRRCPGYGHALKHMRALVANMLHGFELKLPNGMKPED
VDMEEKYGLATHPMVPLTVIVEPTLPPHRY

>Achantus_ungaricus_TRINITY_DN7030_c0_g1_i1_1
MEGFLWWLLVLALAYVLSSFISKIGNANKPKLPPGPKPWPIIGNLNLVGPIPHQSLHHLFKKYGELMHLKFGQFPVVVASTPEMAKLFLKTHDTA
FASRPALDAGRYTSYNYTDLTWAPYGPYWRVARKIYVVDVFSPKKLDSEAFEKIRVEERRVFFSAMHKLAGKPVVLRGHLARAAVTTISRMVM
GKMYFGQEEGCIIGTDEFQELLDEWFFLNGVFSICDWIPWLRFLDPQGYVRRMKKLHKELDRIFDFVVADHEALRMKDRESCTEKDVVYKMLD
MTEDSNLGFVLEREHVKALLQNLILGGTDTTPTSVEWILCELMRNPRIVRKAKEELDRAVGRDRWVEESDFHNLPYLDAIIKEQMRLHPIATFLA
PHCAMEDCNVAGYDIPKGATILINSWSLGRDPDSWEKPEEFLPERFMENEIDIMGSNFAMLPFGAGRRRCPGYGHALKNIRALVANMLHGFEL
KLPNGMKAEDVDMEEEYGLTTHPKEPLALVMEPTLPSHLY
>Lamium_1 album_Cluster-10884.0
MLLKFGKFPVVVASSPEMAKQFLKVHDAVFASRPAFAAGKYISYNYSDMLWAPYGPYWRQARKIFHSKVFSAQTLEFSERIRVEERRNLLSRL
SSQAGKPVVLRDQLLHFNLSIISRMVFGKKYFSEGEEDNRINKIEELKGMLDEWLLLTGAKNIGYWIPWLSFLDLQGYVKRMKVLFKKFDRFYD
NVIDDHQASRVAEIEQFDIVDVLLQEAEDPNLEVELTRDRIKGLLHDQLGGGTDTAATIIEWAILEILRDPRIMKKAKDELNRIIGRNRWVEENDFS
QLPYIEAIIMEAFRLHPIATLLAPHYATEDCNVSGYDIAKGTVIVINTWSIGRDPKSWDVPQDFLPERFIGNKIDMMGSNFALLPFGSGRRRCPGY
SLALKVVRTTLANLVHGFEMKLVEGMRVEDICMDEVFGLTIQRPKQSISIIMEPTLPPHLY
>Lamium_1 galeobdolon_Contig_13076
MEIISWFLLALLYVASLALISKIRRPPIQNLPPGPKPWPIIGNLNLIGSIPHQSLHYLSQKYGEIMLLKFGKFPVLVASSPNMAKQFLRVHDVDFASR
RLLAAGKYISYNYSDLSWAPYGPYWRQARKIYLSEVFKPKKLEFFEQVRVQETRNFLSRLYTLSGKPVVLRDQLQRYTLSTICRMLSRDVYFTS
SEEDELRGMAEEWYFLTGVFNIGDWIPWLSFLDLQGYVKRMKVLNKKLDRVFDYVIDEHLAKRAKDKDFVAKDIVDVFLQISEDPNLHVELTRY
HVKALLQNLMVGITDTSATTIEWALNEVLRHPLIMKKAKEEIDGVIGRNRWVQETDVSQLPYIESIIMETFRLHPLATFLAPHCAINDCNVAGYDIA
KGTMVLINTWSIGRDPNSWDAPVEFLPERFMGKEIDATGSNFSFLPFGSGRRRCPGYKLALKMIPTTFANLLHGFNFKLVEGMKLQDICMEEE
YGLTTHPKRPLSIIMEPSLPPHLY
>Lamium_1 galeobdolon_Contig_675
MEISWLLLTLVYVATLALISKIIKKQRPPKSNLPPGPKPWPIIGNLNLLGSIPHQSLHHLSKKYGEIMLLKFGKFPVLVASSPNMAKQFLKVHDAHF
ASRPLLSAGKYTSYNYSDLVWAPHGPYWRQARKIFLHEVFSAKKLDFFAHIRAHEARSLLSRLLPLSGKAVVLRDELQRYTLSTISRMLSRECF
ASLEDDELRGMIAEWFFLTGVFNIGDWIPSLRFLDLQGYVKRMKVLHEKLDRVFNYVIDQHLMAKRDKGKDFTAKDIVDAFLHISEDPNLDVELT
RDCVKALIQNLIVGGTDTSATTIEWAMNEVLRHPHVLEKAKEELNRIIEKNKWVQESDLSQLPYIESIVMETFRLHPVGTLLAPHCAINDCKVAGY
DIAKGTTVLINVWSIGRDRNSWDAPEKFLPERFVGKEIDVLGSNFSLLPFGSGRRKCPGYNLALKMVPLTIANLLLGFNFKLVKGMEPQDICVEE
EYGLSTHPKVPLSIIMEPTLPPHLY
>Lamium_1 galeobdolon_Contig_31681
MELSSVLFALVYAVAIAFISKIFTKSKQKIPPGPKPWPILGNLLQISSIPHQSFHYLSQKYGEIMLLKIGSKPIVIASSPEMAKQFLKIHDASFASRPPI
AAGKYISYNYSFVALAPFGPYWRQTRKICLSEVLNAKKLESFVQAHAEERSHFISRLRVLSETGKQVVLRHHLARHTLAITSRMLFRDECYEDK
KMDELHMMVDEWFVLAGAYNIGDWIPWLSFLDLQGIVRRMKALNKKFDEFYDNVIEDHLARRGKGKDFATKDVMDAVLEIADDPNLEVKLTRN
CVKALIQNLLLGGIDTSASTVEWAICEILRQPRIMKKAKDELDRVVGRKRWVEENDFSELPYIDAIIKETFRLHPPSTLIPHYAINDCNVAGYDVVK
GTTVLINAWSIGRDPKSWDAPKKFMPERFMGKEIDVAGNHFVLLPFGSGRRGCPGYRLGLKMVRVALANLVHGFEVKLVQGMEPKDICMEEE
YGLIVYPKQPLPIIMKPTLPAHLY
>Lamium_1 maculatum_Cluster-2414.0
MEIISWLLLALIFGTTLTLISKLVENRRPSNRKLPPGPKPWPIIGNLNLIGSIPHQSLHHLSLKYGEIMLLKFGKFPVVVASSPEMAKQFLKVHDAVF
ASRPAFAAGKYISYNYSDMLWAPYGPYWRQARKIFHTKVFNAQTLEFSEKIRVEERRNLLSHLSSQAGKPVELRDQLLHFNLSIISRMVFGKKY
FSEREEDNRFNKIEELKGMLDEWFLLTGAQNVGDWIPWLSFLDLQGYVKRMKVLFKKFDRFYDNVIDDHQATRVAEMEQFDMVDVLLQEAED
PNLEVELTRDRIKGLLHDQLGGGTDTAATIIEWAILEILRDPRIMKKAKDELDRVIGRNRWVEENDYSQLPYIEAIIMETFRLHPIATLLAPHYATED
CNVSGYDIAKGTIIVINTWSIGRDPKSWDAPLEFLPERFIGNKIDMMGSNFALLPFGSGRRRCPGYSLALKVVRTALANLVHGFEMKLVEGMRV
EDICMDEVFGLTIQRPKQSISIIMEPTLPPHLY
>Lamium_1 orvala_Cluster-10329.0
MELSSLLLLALIYAVAIAFISKIFTKTKQKIPPGPKPWPIIGNILQIGSIPHQSFHYLSQKYGEIMLLKIGSKPIVIASSPEMAKQFLKIHDASFASRPPIA
AGKYISYNYSFVALAPFGPYWRQSRKICLSEVLNAKKLESFEQAHAEERCHFISRLRFLSETGKQVVLRHHLARHTLAITSRMLFRDECYEEEK
MDELHMMVDEWFVLAGAFNIGDWIPWLSFLDLQGIVRRMKALNKKFDEFYDNVIEDHLARRGKGEDFATKNVMDAVLEIADDPNLEVKLTRNC
VKALIQNLLLGGIDTSACTVEWAICEILRQPRIMKKAKDELDRVVGRKRWVEENDFSELPYIDAIIKETFRLHPPSTLIPHYAINDCNVAGYDIVKG
TTVLINAWSIGRDPKSWDAPEKFMPERFMGKEIDVAGNHFELLPFGSGRRGCPGYRLGFKMVRVALANLVHGFEVKLVQGIKPQNICMEEEN
GLIVYPKKPFPIIMEPALPAHLY
>Aphelandra_squarrosa_Bx4
MAKVCNQILHTGSSFSMGMQMEGLLWWVLAVALAYIVASFFFNNGKKPKLPPGPKPWPIIGNLNLIGPIPHQSLHDLFQKYGELMHLKFGHFP
VVVASTPEMAKQFLKTHDTAFASRPALDAGRLTSYNYTDLTWAPYGPYWRLARKIYVVDVFSAKKLDSDAFHQIRVEERQTFFSLMHSLAGKP
VVLRGHLGRAAVITISRMVMGKAYFGEDESCLIGTDGFQELLDEWFFLNGVFSICDWIPWLRFIDPQGYVRRMKNLHKELDRIFDFVVADHEAT
RMKDTDSCSEKDVVYKMLDMTEEPTLGVALSREHVKALLQNLILGGTDTTPTSVEWILCELMKNPPIVRKAKEELNRAVGRDRWVEESDFPEL
PYLDAIIKETMRLHPIATFLAPHCAMEDCKVAGYDIPKGATVFINSWSLGRDPDSWDRPLEFLPERFMEKEIDIMGSNFAMLPFGAGRRRCPGY
GHALKNIRAIVANLLHGFELKLPNGMKPSDVNMEEEYGLTTHPKEPLALIMEPTLPPHLY
>Lamium galeobdolon_BX4
MEIISWFLLALLYVASLALISKIRRPPIQNLPPGPKPWPIIGNLNLIGSIPHQSLHYLSQKYGEIMLLKFGKFPVIVASSPNMAKLFLKVHDADFASR
PLLAAGKYASYNYSDLTWAPYGPYWRQARKIYLSEVFNTKKFEFFEQIRVQEASNLLSRLRQLSGKPVVLRDELQRYTLSFISRMLSRDVCFTS
EQDDLRGLIDEWFFLTGVFNIGDWIPWLSFLDLQGYVKRMKVLNKKLDRVFDYVIDEHLAKRAKDKDFVAKDIVDVFLQISEDPNLHVELTRYHV
KALLQNLMVGITDTSATTIEWALNEVLRHPLIMKKAKEEIDGVIGRNRWVQETDVSQLPYIESIIMETFRLHPLATFLAPHCAINDCNVAGYDIAKG
TMVLINTWSIGRDSNSWDAPEKFLPERFMREKIDALGNNFSFLPFGSGRRRCPGYKLALKMIPTTFANLLHGFNFKLVEGMKLQDICMEEEYGL
TTHPKVPLSIIMEPTLPPHLY
>Scoparia dulcis _CYP92A46
MESSSALLLLAFIAAVAFISRIINRKPPTKLKHPPGPKPWPIIGNMHLIGSIPHQSLHYLSQKYGELMLLKFGKFPVVVAASPEMAKQFLKIYDTSF
ASRPALAAGKYTSFNYSDLTWAPYGPYWRQARKIYLTEVLNAKAIKTYEHVRMEERRTFFSRLHSLSGKSIVLREQLCRYTLSTMSNVIFGRLV
FDSHEAKGGNAVKLDELQAMLDDWFFFGGALNLGDWIPWLNYIDPQGYVKRMKALNRKFDRFYTSVFSHHLANRDPSAPKIPLDSLLGMPGD
PNLDVKLTEENVKGLLMDLLAGGTDTSATTVEWAVHELLRHPHVYSKVKEEIDRVVGRNRWVEEEDFPQLTYLDAVIKESMRLHPLATFLAPH
CAMEDINIAGYDISKGTMILVNTWSLGRDPKVWDNPEKFSPERFLVEDIDILGSNFALLPFGSGRRRCPGYKLGLKLVRSTLSNLVHGFNWRLP
EGMTVKDVCMEELYGLTTRPKISLEIILEPSLPLHLY
>Nicotiana tabacum_CYP92B2v1
MENSWVFLALAGLSALAFLCKIITCRRPVNRKIPPGPKPWPIIGNLNLLGPIPHQSFDLLSKKYGELMLLKFGSRPVLVASSAEMAKQFLKVHDA
NFASRPMLAGGKYTSYNYCDMTWAPYGPYWRQARRRIYLNQIFTPKRLDSFEYIRVEERQALISQLNSLAGKPFFLKDHLSRFSLCSMTRMVL

SNKYFGESTVRVEDLQYLVDQWFLLNGAFNIGDWIPWLSFLDLQGYVKQMKALKRTFDKFHNIVLDDHRAKKNAEKNFVPKDMVDVLLKMAE
DPNLEVKLTNDCVKGLMQDLLTGGTDSLTAAVQWAFQELLRQPRVIEKATEELDRIVGKERWVEEKDCSQLSYVEAILKETLRLHPLGTMLAP
HCAIEDCNVAGYDIQKGTTVLVNVWTIGRDPKYWDRAQEFLPERFLENDIDMDGHNFAFLPFGSGRRRCPGYSLGLKVIRVTLANMLHGFNW
KLPEGMKPEDISVEEHYGLTTHPKFPVPVILESRLSSDLYSPIT
>Nicotiana tabacum_CYP92B3
MENSWVVLALTGLLTLVFLSKFLHSPRRKQNLPPGPKPWPIVGNIHLLGSTPHRSLHELAKRYGDLMLLKFGSRNVLILSSPDMAREFLKTNDAI
WASRPELAAGKYTAYNYCDMTWARYGPFWRQARRIYLNEIFNPKRLDSFEYIRIEERHNLISRLFVLSGKPILLRDHLTRYTLTSISRTVLSGKYF
SESPGQNSMITLKQLQDMLDKWFLLNGVINIGDWIPWLAFLDLQGYVKQMKELHRNFDKFHNFVLDDHKANRGEKNFVPRDMVDVLLQQAED
PNLEVKLTNDCVKGLMQDLLAGGTDTSATTVEWAFYELLRQPKIMKKAQQELDLVISQDRWVQEKDYTQLPYIESIIKETLRLHPVSTMLPPRIA
LEDCHVAGYDIPKGTILIVNTWSIGRNSQHWESPEEFLPERFEGKNIGVTGQHFALLPFGAGRRKCPGYSLGIRIIRATLANLLHGFNWRLPNG
MSPEDISMEEIYGLITHPKVALDVMMEPRLPNHLYK
>Achantus_leucostachyus_TRINITY_DN2411_c0_g1_i3
MEGILWWLLALPLAYVLASIFSKTTNSKKPKLPPGPKPWPIIGNLNLIGPIPHQSLHHLFNKYGELMHLKFGQFPVVVASTPEMAKQFLKTHDTA
FASRPALDAGRYTSYNYTDLTWAPYGPYWRVARKIYVVDVFSTKKLESEAFEKIRVEERRVFYSVMHKLAGKPVVLRGHLARAAVTTISRMVM
GKMYFGEEEGCIIGTDEFQELLDEWFFLNGVFSICDWIPWLRFLDPQGYVRRMKKLHQELDRIFDFVVADHEAQRVKVGESCSEKDVVYKML
DMTEDPNLGFVLEREHVKALLQNLILGGTDTTPTSVEWILCELMRNPRIVRKVKEELDRAVGRDRWVEESDFPNLPYLDAVIKEQMRLHPIATF
LAPHCAMEDCNVAGYDIPKGATILINSWSLGRDPNSWDKPEEFLPERFIEQDIDIMGSNFAMLPFGAGRRRCPGYGHALKNIRALVANMLHGF
ELKLPNGMKPEDVDMEEEYGLTTHPKEPLALIMEPTLPSHLY
>Aphelandra_aurantiaca_TRINITY_DN1567_c0_g1_i5
MFVNMVDFWALVLIFLLAYSVASFIFRKHPKHKLPPGPKPWPVIGNLNLIGSIPHQSLHLLSKRYGEIMLLRFGKFPVIVASTPEMAKQFLKIHDS
VCASRPALDAGKYTSYGYSDMVWAPYGPYWRQLRKLYFSEVFSAKMAQSKMFEQIRAEERRNLFSHLHSLSGNPVFLRGHLFRYMLSTISKM
AMSKNYYDEAGDENSSVVELKEFQGMLNEWFFLSGVFNIGDWIPWLRFLDLQGYVKRMKALHKKLDPIFDFVIDDHEDRRKGEKNFGSGKDF
VDKLLELAQDSNAEIKLSRNCVKALIQNLIVGGTDTAPTMVEWTIREVMKQPRLIEKAKEELDRVVGRNRWVEEDDCSQLPYIEAIIKESMRLHP
LATLLGPHYTLDDFSVTGYHVPKGSMVLINTWSIGRDPNLWDAPEEFFPERFMGKDIDVLGSNFSLLPFGSGRRRCPGYDLGLRTVRSIFANLL
HGFNLKLVDGMRAEDIGMEEEYGLTTHPKEDLPVVMEPALPPHLY
>Erythranthe guttatus_XM_012999283
MDSLLLVLICTAAIIAFISKTISKLRQPRRNTPPGPKPWPIIGNLNLIGPIPHQSFHFLSRKYGEIMQLKFGKYPVVVASSPEMAKQFLRVHDTVFAT
RPPLAAGKYTAYNYSDMTWAPYGPQWRLARKIYLSEVFSAKKIESFEHVRIEETRNLVSRLRSVSGDPVVLRDHLMHYTVSATCRMILGKKYF
CDSEDDKFVTLQDMLDEWFLLNGAFVISDWIPWLKFLDLQGYVKRMKTLYNRLDNFHNYVIDDHQTRQKGAAAEEEKDFTLVDKLLKMADDP
NLEVKLTRDCVKGLLQDLLAGGTDTSAATVEWALCEILRQPRIIKKAKEELDRVIGRKRWVEENDFSQLPYVEAIIMESMRLHPLATLLAPHYAID
DCKVAGFDISKGTTVLINTWSIGRDPKSWDSPEEFLPERFLGKETDMLGSNFALLPFGSGRRRCPGYNLGLKMVRTMLANLLHGFEFQLAEDV
CLEEMYGLTTHPKKPLVVVMEPTLPSQLY
>Nicotiana tabacum_CYP51G1
MELGDNKILNAGLLLVATLVVAKLISALIMPRSKKRLPPVIKSWPILGGLLRFLKGPVVMLREEYPKLGSVFTLNLLNKNITFFVGPEVSAHFFKAP
ETDLSQQEVYQFNVPTFGPGVVFDVDYTIRQEQFRFFTESLRVNKLKGYVDQMVMEAEEYFSKWGDSGEVDLKYELEHLIILTASRCLLGEEV
RNKLFEDVSALFHDLDNGMLPISVIFPYLPIPAHRRRDNARKKLAEIFANIIDSRKRTGKAESDMLQCFIDSKYKDGRATTESEITGLLIAALFAGQ
HTSSITSTWAGAYLLCNNKYMSAVVDEQKNLMKKHGNKVDHDILSEMEVLYRCIKEALRLHPPLIMLLRSSHSDFTVKTREGKEYDIPKGHIVAT
SPAFANRLPHVYKNPDTYDPDRFAPGRDEDKVAGAFSYISFGGGRHGCLGEPFAYLQIKAIWSHLLRNFEFELISPFPEIDWNAMVVGVKGKV
MVKYKRRKLSTE
>Catharanthus roseus_CYP72A1v1
MEMDMYTIRKAIAATIFALVVAWAWRVLDWAWFTPKRIEKRLRQQGFRGNPYRFLVGDVKESGKMHQEALSKPMEFNNDIVPRLMPHINHTIN
TYGGNSFTWMGRIPRIHVMEPELIKEVLTHSSKYQKNFDVHNPLVKFLLTGVGSFEGAKWSKHRRIISPAFTLEKLKSMLPAFAICYHDMLTKW
EKIAEKQGSHEIDIFPTFDVLTSDVISKVAFGSTYEEGGKIFRLLKELMDLTIDCMRDVYIPGWSYLPTKRNKRMKEINKEITDMLRFIINKRMKAL
KAGEPGEDDLLGVLLESNIQEIQKQGNKKDGGMSINDVIEECKLFYFAGQETTGVLLTWTTILLSKHPEWQERAREEVLQAFGKNKPEFERLNH
LKYVSMILYEVLRLYPPVIDLTKIVHEDTKLGPYTIPAGTQVMLPTVMLHREKSIWGEDATEFNPMRFADGVANATKNNVTYLPFSWGPRVCLG
QNFALLQAKLGLAMILQRFTFDVAPSYVHAPFTILTVQPQFGSHVIYKKLES
>Strobilanthes_dyerianus_WEAC_scaffold_2012786
MMWVFWLLLALVSITVPFIFKKHPKKRLPPGPKPWPIIGNLNLIGSMPHQSLHLLSQKYGEIMFLKFGQFPVVVASTPDMARLFLRTHDTVFASR
PALSVGKYTSYNHSDMAWAPYGPHWRQARKLYLTEVFSPRRMESPPFVQIRVEERHNFISRLHALSGKPVFLRDHLFRYALATINRMVLGEKY
FSEFEGDKGNIWLPAFQEMMDEWFFLGGVFNIGDWIPWLNILDLQGYVKRMKSLHKNLDRMNELVLNDHLKKRAAAENNTDARIDFVDKLLEL
ADDGSLEVKLTRDHVKALTQNLLVGGADTTPTTVEWIVHELMKNPSIYEKAKAEVDTVICRNRWVEENDASQLPYMEAIIMETLRLHPITTLLLP
HYATEECQVAGYDIPKGTTVLVNTWSIGRDPNIWDAPEVFSPERFLGKEVDMKGGNFALLPFGSGRRRCPGYSLGLKSVRAIVGNLLHGFNW
KLEDGMKPEDVTLEEMYRLTTYPKQALHIILEPALPAHLY
>Strobilanthes_dyerianus_WEAC_scaffold_2083039
MMFPIMDGWWALLLVTMSFIATASIIFKNHRNTRLPPGPKPWPIIGNLNLVGSIPHQSLHLLSQKYGGIMHLKFGQRSVVVASTPHMAKEFLKTH
DAVFASRPPLSAGKYTSYNYSDLVWAPYGPHFRQARKIYLLEMFSSKKLESRVFEQIGIEERCNFIARLHSLSGKPVSLRDHLFHYNFSTLHRM
VLGRKYFTEPGGKTEEGVVSLEEFEGLLDEWFFLSGVFNIGDWIPWLHFLDVQGYVKRMKALNKKLDRIYEYVIHDHLNRRRVAEIDDGDDSK
DLVDKLLQLAQDSSLEVKLTRDCVKALIQNLIIGGADTAPTTIEWIIHELMRHPSIYKKAKEELEKEIERNRWVEEDDFSRLPYIEAIIIEAMRLHPIG
TFLAPHYAIEDCQVVGYDIPKGTTVLINTWSIGRDPNTWDAPDEFLPERFVGKVTDITGGNFGLLPFGSGRRRCPGYNLGLKTVREIVANLLHG
FNLKLGDGMNPEDVSMEEKYRLSTHPKQDLELILEPTLPAYLYSRI
>Anisacanthus_quadrifidas_PCGJ_scaffold_2006835
MMDGFWPLIFVLPYIAILAIVIFTKHPKKCLPPGPKPWPIIGNLDLIGPLPHQSLHTLSKKYGEIMHVKFGQVPVVVASSPEMAREFLKTHDLIFAS
RPLLSVGKYTSYNYSDMTWAPYGPYWRQARKIYLTEMFNTKKLGSKKFEQIRVEERCSLMGRLHSLSGKPSMLRDHLFRYALSTINRMVMGK
RYFREANEDEEGAVVQWEEFQEIVDEWFLLGGVSNVGDWIPWLNFLDLQGYIKRMKLLSVKLDYIYEYVINDHLRRRAAEKIADASEDVVDKLL
DLAEDPNLEIKITRDCVKALVQNLIFGGADTTPTTIEWIVLELLRHPRIYKKAKDELDQVIGKKKWVKKMGGRRRLSLSTLCKSHYYGDYEITSAY
YFISSALRHRGLPGCKL
>Ruellia_brittoniana_AYIY_scaffold_2009930
MAKIFLQTMDVTFASRPKTAAGKYTTYNYSNITWSPYGPYWQQARKLCLMELFSAKRLESYEYIRKEELNSLLKEVFESSGKPFLLKDYLSTLS
LNVISRMVMGKSAAENGIVTPDEFKRMVDELFLLNGVINIGDLIPWIGFLDLQGYVKRMKIVSKKFDRFLEHVLDEHAARRQNTEGFVCKDMVD
VLLDLAGDPNLEVKLERHGVKAFTLDMLAGGTETSAVTVEWAISELLKKPQIFEMAMEELDRVIGQNNWVKEGDIPHLPFIDAIVKETMRLHPVV
PMLVPRMSREDCKVAGYDIQKGTQVLVNVWMIGRDPSLWENPSEFCPERFMGKDIDVKGHHFELLPFGAGRRMCPGYNLGLKVIQSSLANLL
HGFKWRLPHGMTVEDLNMEEIFGLTTPRKIPLATVAEPRLPLEMFSM

>Strobilanthes_dyerianus_WEAC_scaffold_2083831
MTTNLLLCTSILALSLCFLFVSRLRRGKEAGLPPGPKPWPIVGNLLQLGPKPHQSMAALARVYGSLVHLRMGMVHVVVAASAGVAEQFLKVHD
GNFANRPPNAGAKHMAYNYQDLVFAPYGPRWRLLRKICAVHLFSTKALDDFRHVRQEETGILTNAIASVGQTPVCIGHMVNVCATNAIARVML
GRRVVSHESGGGDTKAEEFKAMVVELMVLAGVFNIGDFIPALNGFDLQGVAAKMKKLHARFDAFFSKIVEEHKMKQGSIDDVKEHEDLLSMLI
SLKDADSGEGGRLTDTEIKALLLNLFTAGTDTTSSTVEWAIAEVIRHPIILAQAQKEVDSVVGKDRLVVEDDLSQLTFLQAIVKENFRLHPSTPLSL
PRIAQENCEINGYLIPKGSTLLVNIWAIARDPNVWDDPLEFRPQRFLTGGEKSNVDIKGNDFELIPFGAGRRICAGMSLGIRMVQLLVATLIHAFD
FELANGKSIQDLNMEEAYGLTLQRAEPLLVHPKPRLAPHVYKARFG
>Anisacanthus_quadrifidas_PCGJ_scaffold_2009060
MQMMNPVVFICTLILGLSVCFLLLRRRLHGGKGRQHHLPLPPGPRPWPIVGNLLQLGQKPHQSMAALARVYGPIMHLKMGFVHVVVAASAGV
AEQLLKAHDTNFSNRPPNAGSKYISYNSKGLVFEPYGPRWRLLRKICAFHLFSNKALDSFSHVRQEEVGTLTCIIASVGKTPVHIGHMINICATN
AISRVMLGQSLVRYESGGGIANAKEFKAMVVEAMELAGVFNIGDFVPALEGLDLQGVVAKMKKLHLRFDAFLTKILEEHKMKGWDGVKEHVDL
LSKLISLKDEDGESLTDTEIKALLLNLFIAGTDTTSSTVEWALAELIRHPKILAQAQKELDSIVGKDRLVTEDDLSRLTFLQAIIKENFRLHPSTPLSL
PRIAQENCEINGYLIPKGSTLLFNVWAIGRDPNTWVEPLEFRPQRFLIGGERPNADIKGNDFELIPFGAGRRICAGLGLGIRMVQLLTATLIHAFD
FELADGKSTQNLNMEEAYGLTLQRAEPLMVHPKPRLASHVYNT

Sequences used for BX5 phylogenetic analysis

>Lamium maculatum_BX5-like
MEVINFTTLVLFSSVIFLLVKAWRKPPNPNPQNLPPSPPSLPVIGHLHLLGGGAVPALARLKKKYGPIISLKLGEVTAVVISSREATKEALKIHDPA
CADRPDSTALEIMFYSYGDIAFCPYNEYWRQMRKICILEMLSAKNVKSYGYIRAEEIDSLVESLRLSSGEAVNITENISSLTFAVTCRASFGRVLQ
GHAKLIALLKELSTMAGGFEVADLFPSLKFLHPFSLNKYRLLRMRREMDAILDPAVEEHKLKKSGEFEGEDFVDVLLRMQKNKELQFPITTDNIK
SVILDMFAGGIETTATTTDWAMAELMRNPRVMAKLQSEIREVLKGKTTVENRDVQGLKYLKLVVKESLRLHPPIPVLPRKCRQECRVGGFTIPN
KAKVMIDVHSLGRDPQYWNDPETFLPERFENSSLDFLGSEYEFLPFGAGKRNCPGLNFGIANVEFTLAQLLYHFDWKLPQGMSLADVDMTEIE
GLAVLRKNPLMVIPTPYEQPL
>Lamium galeobdolon_BX5-like
MEVIDFTTLLLFSSLIFLLIKAWRKPQNPNPQNLPPSPPSLPVIGHLHLLGGGTVPALARIRKKYGPIVSLKLGEVTAVVISSRETVKEALKIHDPAC
ADRPDSTALEIMFYSYGDIAFCPYNEYWRQMRKICILEMLSAKNVKSFGYIRVEEIDSLVESLRLSSGEAVNITEKISSLTFAVTCRASFGRVLKG
HGKLIALLKELSTMAAGFEVADLFPSLKFLHPFSLNKYRLLRMRREMDDILDPAVEEHKLKRSGEFEGEDFVDVLLRVQKNKELQFPITTDNIKS
VILDMFAGGIETTATTTDWAMAELMRNPRVMAKVQSEIREALKGKTTVENKDVQNLKYLKLVVKESLRLHPPIPLLPRKCRQECIVGGFTIPNKA
KVMIDVHSLGRDPQYWNDPETFLPERFENSSLDFLGSDYEFLPFGAGKRNCPGLNFGIANVEFPLAQLLYHFDWKLPQGMSHADVDMTEIEG
LAVLRKNPLMVIPTPYNPSL
>Lamium orvala_BX5-like
MEVINFTTLLLFSSLIFLLIKAWRKPQNPNPQNLPPSPPSLPVIGHLHLLGGGAVPALARLKKKYGPIISLKLGEVTAVVISSREATKEALKIHDPAC
ADRPDSTALEIMFYSYGDIAFCPYNEYWRQMRKICILEMLSAKNVKSYGYIRAEEIDSLVESLRLSSGEAVNITEKISSLTFAITCRASFGRVLKGH
AKLIALLKELSTMAGGFEVADLFPSLTFLHPFSLNKYRLLRMRREMDAILDPAVEEHKLKKSGEFEGEDFVDVLLRVQKNKELQFPITTDNIKSIIL
DMFAGGIETTATTTDWAMAELMRNPRVMTKVQSEIREALKGKTTVENKDVQNLKYLKLVVKESLRLHPPIPLLPRKCRQECKVGAFTIPNKAKV
MIDVHSLGRDPQYWNDPETFLPERFEESSLDFLGSNYEFLPFGAGKRNCPGINFGIANVEFPLAQLLYHFDWKLPQGMSHADVDMTEIEGLAV
LRKNPLMVIPTPYEQPL
>Lamium galeobdolon_Bx5
MEVIDFTTLLAFSSLIFLLIKAWRNPNPQNLPPSPPSLPVIGHLHLLGGGAIQALARIRKKYGPIVSLKLGEVTTVVISSREATKEALKIHDPACADR
PDSTAVKIMFYGCEDISFGPYNENWRQLRKICIMEMLSPKNVKSFGYIREEEIDSLLESLRLSSGEAVNLTEKISTLTFAVTCRASFGRVLKGHGT
VVALLKELSPMSTGFQVADLFPSMKFLHPFSLNKYRAQQMRREMDDILDTAVEEQKLKKSGEFEAENFVDVLLRVQKNKELKFPITTDNIKSVIL
DIFAGGIETTATTNDWAMAELMRNPRVMAKVQSEIREALKGKTTVEEKDIQSLKYLKLVVKESLRLHPPVPLLPRKCRQECRVGGFTIPNKAKV
LINVHSLGRDPLYWNDPETFLPERFENSSLDFLGNDCEFLPFGAGKRNCPGLNFGIANVEFPLAQLLYHFDWKLPQGMSHADVDMTEAEGMA
LLRKSPLMVIPTPYKQPL
>Lamium galeobdolon_Contig_32188
MDIFHQIPLNFTTAVLIASFVFLLIKAITKKSPQNLPPSPPSLPVIGHLHHLAGGLLHEGLDKLSKTYGPIISLNLGQVRAVAISSPELAKEVTKVLDP
ACADRPESIALRTMFYENTDIAFCAYNDYWRQMRKICILELLSAKNVKSFGYIRQDETNTLIESLKFVSGKTVNLTEKVFVFSCTVTCRATFGNVL
GDRDKFIVLVKKLAHMAGGMELADLFPSIKFLTVFTWNRFQLWKMKRGMDAILDPILAEHKLKRSGEGGSEDIVDVLLRVKKSGQLQFPITNDN
VKAVILDMFAAGTESSATTIDWAMAELMRDPRVMTRVQSEIRETLKGKTTVEESDVQGLTYLKLVIKETLRLHPPFPLLPRQCKDEFKVDGYTIP
VKTKVMVNVWGIARDPKYWEDAESFKPERFENSSIDFLGSNYEFLPFGAGRRNCPGLSFGIANLELPLAQLLFHFDWKLPQGMNPSDVDMTG
VEGLAVGRKTPLVLIPTIHNPSN
>Lamium album_Cluster-2496.9342
MEIISLTTLLLLSTFIFFLIKTIKKPKPSENEGKLPPSPPKLPLIGHLHHLTGDLPHHALSKISKKYGDIVSLKLGETNAVIVSSRELAKEAMKVHDLH
FADRPNSVSVDILWYNNSGLAFCPYGEEWRQMRKICMLEMLSPKNVKSFGYIREDEIINLVKSLRSLSSGESVDFTETISSFNCSITCRAALGKV
LGDRDTLIPLIKTAVGMSGGFEVADLFPSFKLLHFLSVKKYMLPGMRRKIDAILDRMVEDHKVKKSGEYDGEDIVDVLLRMQETGELRFPVTTE
NIKAIILDMFAAGTETVSTSMDWIMTELMRHPRVMTKLQEEIRGALKGKTRLEESDVQGLKYMKSVIKETMRLHPPVPIIPRKCREECRIGGYSIP
LNSSLIINVWSLGRDPKYWNDPETFLPERFDENSLDMLGHDFEFLPFGSGRRICPGLNFGLTNVQFTLAQLLYNFDWKLPDGMKPSDVDMSEL
DGLAVGRKTPLLMVPTPYNHHS
>Lamium maculatum_Cluster-9868.0
MESLSTLILLLFSSSIVLLLLIKSWRKDKNPHNFPPSPPALPLIGHLHHLSGTLVHHAMTRINKKYGPVVTLKLGEVTTVVISSRETTKEALRTNDP
ACADRPDSGVLEIMFYNYSDIAFGAYSEYWRQMRKICILEMLSAKNVKSFGYIRAEEMDGAVESVRSRSGEPLNFTEMIFMMTCAVTCRATFG
KVLKGRETFIKLLKKMATMAGGYELTDLFPSIKILNLVSKNKAKLLKMRDEMDAILDPAVEEHRSNKSGEFEGEDIVDVLLRMQKNKELQFPITTD
NIKAVILDMFTAGTETSATTTDWTMALLMKNPRVMAKLQSEIREAFKGKSSVEEKDVQKLKYLKNVVKESLRLHPPFPLLPRKCRQECKVGGYT
IPNKAKVMINVGSLCRDPLYWEDPEAFQPERFDNSSIDFLGNDYEYLPFGSGKRNCPGLNFGIANVEFPLAQLLFHFDWKLPQGMAPADVDMT
EQEGQPIARKNPLILLPTLYNPPSSN
>Lamium maculatum_Cluster-9204.9250
MEIITFTVLLLLPTFIFFLIKTIKKRKPEEKLPPSPPKLPLIGHLHHLAGGLPHQALSQISKKYGDIVSLKLGETNAVIVSSRELAKEAMKVHDLHFAD
RPNSVSVDILWYNNSGLAFSPYGEEWRQMRKICMLEMLSPKNVKSFGHIREDEIINLVKTLRSLPSGESVDFTETISIFNSSITCRAALGKVLGD

RDTLIPLIKMAVGMSGGFEVADLFPSFKLLHFLSVKKYKLPGMRRKIDGILDRMVEDHKVKKSGEYDGEDIVDVLLRMQETGELRFPVTTENIKA
IILDMFAAGTETVSTSMDWIMTELMRHPRVMTKLQEEIRGALKGKTRLEESDVQELKYMKLVIKETMRLHPPVPIIPRKCREECRIGGYSIPLNSS
LIINVWSLGRDPKYWNDPETFLPERFEENSRDMLGHDFEFLPFGSGRRICPGLNFGLANVQFTLAQLIYNFDWKLPNGMKPSDVDMSELDGLA
VGRKTPLLLVPTPYNPSK
>Lamium orvala_Cluster-10786.0
MESLSTLILVLFSSSIVLLLLIKSWRKEKNPQNFPPSPPALPLIGHLHHLGGTLVHHAMTRINKKYGPVVTLKLGEVTTVVISSRETTKEALRTNDP
ACADRPDSGVLEIMFYNYSDIAFGAYSEYWRQMRKICILEMLSAKNVKSFGYIRAEEMDSAVESVRSRSGEPLNFTEMIFMMTCAVTCRATFG
KVLKGRETFIKLLKKMATMAGGYELTDLFPSIKILNLVSRNKAKLLKMRDEMDAILDPAVDEHRLNKSGEFEGEDIVDVLLRMQKNKELQFPITTD
NIKAVILDMFTAGTETSATTTDWTMALLMKNPRVMAKLQSEIRDTFKGKSSVEEKDVHKLKYLKMVVKESLRLHPPFPLLPRKCRQECKVGGY
TIPNKAKVMINVGSLCRDPLYWEDPEAFQPERFDNSSIDFLGNDYEYLPFGSGKRNCPGLNFGIANVEFPLAQLLFHFDWKLPQGMTPADVDL
TEQEGQPIARKNPLILLPTLCNPSSN
>Lamium orvala_Cluster-6989.0
MEIISFTTLLLLSTFIFFLVKTIKKPKPSKNEEKLPPSPPKLPLIGHLHHLAGGLPHQALSKISKKYGDIVSLKLGETNAVIVSSRELAKEAMKVHDLH
FADRPNSVLVDILWYNNSGLAFCPYGEEWRQMRKICMVEMLSPKNVKSFGYIREDEIINLVKTLRSLSSGESVDFTETISRFNSSITCRAALGKV
LGDRDTLIPLIKTAVGMSGGFEIADLFPSLKLLHLFSVGKYKLPGMRRKIDAILDLMVEDHKLKKSGEYDGEDIVDVLLRMQETGELKFPVTTENI
KAIILDMFAAGTETVSTSMDWIMTELMRHPRVMVKLQEEIRGALKGKTRLEESDVQELKYMKLVIKETMRLHPPVPIIPRKCREECRIGGYSIPLN
TSLIINVWSLGRDPKYWNDPETFLPERFEENSLDLLGHDFEFLPFGSGRRICPGLNFGLTNVHFTLAQLLYNFDWKLPNGMKPSDVDMSELDG
LAVGRNTPLLLVPTPYNHS
>Salvia miltiorrhiza_KP337668.1
MEMDLPFNFTTTLLLSSFIFFLLKAWTKPKSPKAHQNLPPSPPKLPVIGHIHLLVGDLPHKALQRQAQKHGPVMHLQLGEVPAVVISSREATRE
VLKVQDPACADRPESIASKILWYDYTDIAFSAYNEYWRQMRKICIVELLSSKNVKSFGHIRRDESSRLMKSLECSSGNAIDLTDKIFTFTSTITCR
AAFGKVMTDRDGLIALFKEAVAMAGGFELADLFPSWKLLNVLSWSKYRLWRMRGKLDAILDGIIDEHKLKQSGEFGGEDIVDVLIRMQQTGELK
FPITTDNIKAVIFDMFVAGTETSSTTTVWAMSEMMRNPRVMEKAQAEVRAALKGKAAVEESDVQELKYLKLVIKETFRLHPPIPLLPRQTREECK
VDGYSIPIKTKVMLNIWSMGRDPQYWEQPEKFQPERFENSPKDFIGNDFEYIPFGAGRRICPGLNFGLANIELPLAKILYHFDWKLPKGMSYER
LDMSEGEGLTVSRKKPLIIPTLCNPVN
>Lavandula angustifolia_MN822899.1
MAVELPFTFTALLLFSSFIFFLIKAWSTKPKPHQNLPPSPPKLPVIGHLHHLVGDLPHKQLKKLSTKHGPVIHLQLGEVPAVVISSREATKEVLKVH
DPACADRPESIASKILWYDYTDIAFSAYNEYWRQMRKICIVELLSTKNVKSFGYIRQDEAVRLIKSLQSSSGQVVDLTDKIFTFTGTITCRAAFGR
VMTDREGLIDLFKKAVAMAGGFELADLFPSWKALHLLSWNKYKLLRMRGKLDAILDRIVDEHKLKKSGEFGGEDIVDVLLRMQRTGELKFPITT
DNIKAVIFDMFVAGTETSSTMTIWAMSELMKNPEAMQKAQAEVREALKGKTAVEESDVVELKYLKLVIKETFRLHPPIPLLPRQSREECKASGY
SIPVKTKVMLNIWSMGRDPLYWEEPEMFRPERFENSPKDFIGNDFEYIPFGAGRRICPGLNFGLANIELPLAKLLYHFDWKLSKGTSSDLDMSE
GEGLTVSRKNPLLLVPTVCYPAN
>Mentha spicata_AY622319.1
MELQISSAIIILVATFVASLLIKQWRKSESRQNLPPGPPKLPLVGHLHLLWGKLPQHAMADMAKKYGPVTHVQLGEVFSVVLSSREATKEAMKLL
DPACADRFESIGTRIMWYDNDDIIFSPYSDHWRQMRKICVSELLSARNVRSFGFIRQDEMSRLLRHLQSSAGETVDMTERIATLTCSIICRAAFG
AIINDHEELVELVKDSLSMASGFELADLFPSSKLLNLLCWNKSKLWRMRRRVDTILEAIVDEHKLKKSGEFGGEDIIDVLFRMQKDSQIKVPITTN
AIKAFIFDTFSAGTETSSTTTLWVMAELMRNPAVMAKAQAEVRAALKGKTSVDVDDVQELKYMKSVVKETMRMHPPIPLIPRSCREECEVNGY
KIPNKARIMINVWSMGRNPLYWEKPETFWPERFDQVSRDFMGSDFEFIPFGAGRRICPGLNFGLANVEVPLAQLLYHFDWKLAEGMKPSDMD
MSEAEGLTGIRKNNLLLVPTLYKSP
>Agastache rugosa_OK649356.1
MEVVEVCWALILAVSSYIILKQWRELKSQEKYSKLPPSPPKVPVIGHLHLFWRELPQHALWRVAEKYGPVAHVQLGEVFTVVLSSAETAKEALQ
TLDPACADRFDSISSKIMWYDNKDIIFSPFNEHWRHMRKICVSELLSARNVRSFGFIRQDEMSRLILSLQSSSGSAVDMTEKICTMTCSIICRAAF
GSVIKDYEVLVDLVKQALSMASGFELPDLFPSSKLVNMLSWNKSRLWKMRRRVDAIVEAIVEEHRLKQSGEFGGEDIIDVLLRMEKDSNVKIPIT
INAIKAFIFDTFSAGTETSSTTTLWVMAELMKNPAVMAKAQAEVRAALKGKSSVDVDDVQELKYLKSVVKETMRMHPPIPLLPRQCREECEVN
GYTIPKKARVVVNIWSMGRDPKYWDEPHTFRPERFDHVSKDFMGNDFELIPFGAGRRICPGLNFGLANVEVPMAQLLYHFDWKLPPGVNLAD
IDMDETPGLTGVRRENLVLIPTPYNP
>Triticum aestivum_Bx5
MAFEGAYHFLQLAVGHATSSPAALLLVVVPLLLLLLASVRRSTMTGRKLRLPPSPPGSLPIIGHLHHIGAQTHISLQHLVDKYGHNGLLFLRAGA
VPTVIVSSPSAAEAVMRTHDHILASRPWSMASHILRYNTTDVAFSPLGEYWQHTRKLVNTHLLSAKKVHSFANGRQEEVCLVVNKIREAATTAP
STAVDMSEFLAAYTNDVVSRSVLGATHRKKGRNTLFREMTETNVDLLVGFNLENFIPRWPLTEVLFRLVCWKVQRHLNKWDALLEEVIKEHINL
KQDNSADFIHVFLSLQQEYGLTDDNVKSLLMNIFEAAIETSYLVLEYAMAELINNRHVMKKLQTEVRTFASSKGKKLDMITEEDLSSLPYLKATM
KEALRLHPPGPLLLPHYSTADCSIDGYDIPAKTRILVNGWAIGRDPKAWERPEEFMPERFLQDGQEKSSNLGQDFKYLPFGSGRRICPGANFA
LATMEIMLVNLMYHFDWEVPNEKDGAGGKVSMAETFGLMLRRNEKLYLVPRIA
>Secale cereale_Bx5
MALEAAHHYLQLAVGHGTSTPAALLLVLVPLLLLLLASVRTSASTRKLRLPPSPPGSLPIIGHLHHIGAQTHISLQHLVDKYGHNGLLFLRAGAVP
TVIVSSPSAAEAVMRTHDHILASRPWSMASHILRYNTTDVAFSPLGEYWQHTRKLVNTHLLNAKKVHSFRHGRQEEVCLVVNKIREAATNAPS
TAVDMSEFLAAYTNDVVSRSVLGATHRKKGRNTLFREMTETNVDLLVGFNLENFIPRWPLTEVLFLRLVCWKVQRHLNKWDALLEEVIKEHMN
LKQDNSADFIHVFLSLQQEYGLTDDNVKSLLMNIFEAAIETSYLVLEYAMAELINNRHVMKKLQTEVRTFASSKGKKLDMITEEDLSSLPYLKATM
KEALRLHPPGPLLLPHYSTADCNIDGYDIPAKTRILVNGWAIGRDPTAWEKPEDFMPERFLQDGQEKSSNLGQDFKYLPFGSGRRICPGANFA
LATMEIMLVNLMYHFDWEVPNEKDGTGGKVSMAETFGLMLRRNEKLYLVPRIV
>Hordeum lecheri_Bx5
MALEAAHHYLRHAVGHGTSAPAALLLVCVPLLLLLLLFASLRTSASTRKLRLPPSPPGSLPIIGHLHHIGAQTHISLQHLVDKYGHNGLLFLRAGA
VPTVIVSSPSAAEAVMRTHDHILASRPWSMASHILRYNTTDVAFSPLGEYWQHTRKLVNTHLLSAKKVHSFRHGRQEEVSLVVDKIREAATNAP
STVVDMSEFLAAYTNDVVSRSVLGATHRKKGRNTLFREMTETNVDLLVGFNLENFIPRWPLTEVLFRLVCWKVQRHLNKWDALLEEVIKEHINL
KQDNSADFIHVFLSLQQEYGLTDDNVKSLLMNIFEAAIETSYLVLEYAMAELINNRHVMKKLQTEVRTFASSKGKKLDMITEEDLSSLPYLKATM
KEALRLHPPGPLLLPHYSTADCNIDGYDIPAKTRILVNGWAIGRDPTAWERQEDFMPERFLQEGQEKSSNLGQDFKYLPFGSGRRICPGANFA
LATMEIMLVNLMYHFDWEVPNEKEGTGGKVSMAETFGLMLRRNEKLYLVPRIVE
>Zea mays_Bx5_CYP71C3v1
MALQAAYEYLQQAVGHGAWSSTQTLTLLLIAVPTVLLLLASLAKSTSSSGRGKPPLPPSPPGTLPIVGHLHHIGPQTHISLQELVAKYGHNGFLF
LRAGAVPTLIVSSPSAAEAVMRTHDHICASRPWSMASHILRYNTCDVAFSPLGEYWQQTRKLMNTHLLSNKKVYSFRHGREEEVCLVVDNLR
EAAAKSPSTAVDMSEVLAAYTNDVVSRSVLGSTHRKKGRNTLFREMTMTNVDLLVGFNLEYYIPRWPLTDLLFRLVCWKVTRHLKRWDALLE
EVIHEHVEMRKLSGDKEKESDDFIDIFLSRYEEYGFTMDNVKSLLMNVFEAAIETSYLVLESAMAELMNHRRVMKKLQAEVRAYGAEKKLDMIR

EDDLSSLPYLKASMKEALRLHPPGPLLLPHYSTADCQIDGYHIPANPRVLVNGWAIGRDPAVWEKPEEFMPERFMRDGWDKSNSYSGQDFR
YLPFGSGRRICPGANFGLATMEIMLANLMYHFDWEVPNEKEDGCWKVSMDEKFGLMLRRNELLYLVPRASS
>Mentha spicata_CYP71D18
MELDLLSAIIILVATYIVSLLINQWRKSKSQQNLPPSPPKLPVIGHLHFLWGGLPQHVFRSIAQKYGPVAHVQLGEVYSVVLSSAEAAKQAMKVL
DPNFADRFDGIGSRTMWYDKDDIIFSPYNDHWRQMRRICVTELLSPKNVRSFGYIRQEEIERLIRLLGSSGGAPVDVTEEVSKMSCVVVCRAAF
GSVLKDQGSLAELVKESLALASGFELADLYPSSWLLNLLSLNKYRLQRMRRRLDHILDGFLEEHREKKSGEFGGEDIVDVLFRMQKGSDIKIPIT
SNCIKGFIFDTFSAGAETSSTTISWALSELMRNPAKMAKVQAEVREALKGKTVVDLSEVQELKYLRSVLKETLRLHPPFPLIPRQSREECEVNGY
TIPAKTRIFINVWAIGRDPQYWEDPDTFRPERFDEVSRDFMGNDFEFIPFGAGRRICPGLHFGLANVEIPLAQLLYHFDWKLPQGMTDADLDMT
ETPGLSGPKKKNVCLVPTLYKSP
>Zea mays_BX3_CYP71C2
MALGAAYHHYLQLAGDHGTATHALLLGVLIFLVIRLVSARRTGTTSANKRKQQQRLPLPPWPPGKLPIIGHLHLIGAETHISIRDLDAKHGRNGLL
LLRIGAVPTLFVSSPSAADAVLRTQDHIFASRPPWMAAEIIRYGPSDVAFVPYGEYGRQGRKLLTTHMLSTKKVQSFRHGRQEEVRLVMDKIRA
AATAAPPAAVDLSDLLSGYTNDVVSRAVLGASHRNQGRNRLFSELTEINVSLLAGFNLEDYFPPNMAMADVLLRLVSVKARRLNQRWNDVFD
ELIQEHVQSRPSGESEESEADFIHVLLSIQQEYGLTTDNLKAILVDMFEAGIETSYLTLEYGMAELINNRHVMEKLQTEVRTTMGSPDGKKLDML
AEEDLGSMPYLKATIKETLRLHPPAPFLLPHYSTADSEIDGYFVPAGTRVLVHAWALGRDRTTWEKPEEFMPERFVQEPGAVDVHMKGKDLR
FIPFGSGRRICPGMNFGFATMEVMLANLMYHFDWEVPGSGAGVSMEESFGLTLRRKEKLLLVPRIAS
>Triticum aestivum_Bx2
MAHVHVDEMLHEAAAAAPRSLLIATAVLFSLVVLPLLLRIIAKQGAASDAKLLSLLPSPPTRLPIIGHLHLMGDLPYVSLAGLAAKYGPELMLVHLG
AVPTAVVSSPRTAEAVLRTHDHIFASRPRSMVFDIIMYGQTDSCFSPYGEHFRKARKLVTVHMLNARKIRSQRPAREEEVRQVIGKIAKAAAAR
EAVDMSELLHSYVNDLVCRAVSGKFSQEEGRNKLFRELTDINAALLGGFNILDYFPSLGRFELVCKVACAKARRVRKRWDLLLDKLIDDHAAR
MVSREGEAQPEQEEDKDFIDVSLSLQQEYGLTRDHIKAILIDMFEAGTDTSYMTLEFAMAELIRKPHLMKKLQEEVRRNVPNGQEMAAEDDLP
NMTYLKAVIKETLRLHPPVPLMIPHFSLDACTVDGYTIPANTRVVINAWALGRHSGYWENENEFQPERFMNGAGVDLKPNEFHYLPFGFGRRM
CPGVHSASATVETMLANLMYRFDWKLPPGLKEENIDMTEVFGITVSRKEKLILVPVTA
>Triticum aestivum_Bx3
MALEAAFHYLQLAGVHGTSTPALLLTVLLLLIIRLAWVRTTTVSTRFGKQQQLPPSPPGKLPIIGHLHLLGSQTHISIRDLDAKHGRNGLLLLRIGA
VPTLFVSSPSAAEAVLRTHDQIFASRPPSMAADIIRYGPTDIAFAPYGEYWRQARKLLTTHMLSAKVVHSFRHGRQEEVRLVINKIREAATRGTV
VDMSDLLSGYTNDVVCRAVLGESHRKAGRNRLFSELTEINVSLLGGFSLENYIPPNMVMADVLLRLVSVKAQRLNKRWDDLFNEIIEEHLHPSK
PSSGEQQAADFIDLLLSLKEEYGLTTDNIKAILVDMFEAGIETSYLTLEYGMAELMNNRHILKKLQEEVRSQGKKLDMITEEDLSSMAYLRATIKE
TLRMHPPAPFLLPHFSTADCKVDGYLIPANTRVLVNAWALGRDPSSWERPDDFWPERFLQDQAGDVDTQMRGKDLRFLPFGFGRRICPGMN
FGFATMEVMLANLMYHFDWDVPNMMGTGAGVDMAESFGLTLRRKEKLQLVAQIP
>Triticum aestivum_Bx4
MALEAAYHYLQRAVGHGTSTEALLLTVLLLLIIRLAWVRAFATTTASTKCKQQLPPTPPGKLPIIGHLHLIGSHPHVSFRDLAAKHGRDGLMLVHV
GAVPTVVVSTPQAAEAVLRTHDHVFASRPRNPVADIIRYNSTDIAFAPYGDYWRRARKVVNTHLLSVKMVYSKRHDREEEVRLVVAKIRELAM
AAPGKALDMTELLGGYASDFVCRAVLGESHRKHGRNELFRELTEISASLLGGFNLEDYFPRLANLDVFLRVVCSKAMGVSKRWDNLFNELIAE
YEHGKEDNAEDFVHLLLSLKKEYGLSTDNVKAILVNMFEAAIETSFLVLEYSMAELINNRHVMAKVQKEVRESTPHGEKLDLIMEEDLSRMPYLK
ATIKEAMRIHPPAPFLLPHFSTNDCEINGYTIPAGTRVIVNAWALARDPSHWERAEEFYPERFLQEGRDAEVDMYGKDIRFVPFGAGRRICAGA
TFAIATVEVMLANLIYHFDWELPSEMEAIGAKVDMSDQFGMTLRRTERLHLVPKIYK
>Secale cereale_Bx2
MLHEAAAAAPRSLLVATAVLFSLVVLPLLLRIITKQGAASDAKLLSLLPSPPTKLPIIGHLHLMGDLPYVSLAGLAAKYGPELMLVHLGAVPTAVVS
SPRTAEAILRTHDHIFASRPRSMVFDIIMYGQTDSCFAPYGEHFRKARKLVTVHMLNARKIRSQRPAREEEVRLVIGKVAKAAAARESVDMSEL
LHSYVNDLVCRAVSGKFSQEEGRNKLFRELTDINAALLGGFNILDYFPSLGRFELVCKVACAKARRVRKRWDLLLDKLIDDHAARMVSREDEA
QAEQEEDKDFIDVSLSLQQEYGLTRDHIKAILIDMFEAGTDTSYMTLEFAMAELIRKPHLMKKLQEEVRRNVPNGQEMVAEDDLPNMTYLKAVI
KETLRLHPPVPLMIPHFSLDACTVDGYTIPANTRVVVNAWALGRHSGYWENENEFQPERFMNGAGVDLKPNEFHYLPFGFGRRMCPGVHSA
SATVETMLANLMYRFDWKLPPGLKEEDIDMTEVFGITVSRKEKLILVPVAM
>Secale cereale_Bx3
MALEAAYHYLQLAGIHGTSTPAVLLTVLLLLIVRLAWVRTTTASTRFGKQQQLPPSPPGKLPIIGHLHLLGSQTHISIRDLDAKHGRNGLLLLRIGA
VPTLFVSSPSAAEAVLRTHDQIFASRPPSMAADIIRYGPTDIAFAPYGEYWRQARKLLTTHMLSAKVVHSFRHGRQEEVRLVINKIRAAATRGTA
VDMSELLSGYTNDVVCRAVLGESHRKAGRNRLFSELTEINVSLLGGFSLENYIPPNMVMADVLLRLVSVKAQRLNKRWDELFNEIIEEHLHPSK
PSSGEQQAADFIDLLLSLKEEYGLTTDNIKAILVDMFEAGIETSYLTLEYGMAELMNNRHILKKLQEEVRSQGKKLDMITEEDLSSMAYLRATIKE
TLRLHPPAPFLLPHFSTADCKIDGYLIPSNTRVLVNAWALGRDPSSWERPEDFWPERFLQDQAGDVDTQMRGKDLRFLPFGFGRRICPGMNF
GFATMEVMLANLMYHFDWDVPNIMGTGAGVDMAESFGLTLRRKEKLQLVPQIP
>Secale cereale_Bx4
MALEAAYHYLLLIIRLAWVRAFATTAASTKGKQQLPPTPPGKLPIIGHLHLIGSHPHVSFRDLAAKHGRDGLMLVHVGAVPTVVVSTPQAAEAVL
RTHDHVFASRPRNPVADIIRYNSTDIAFAPYGDYWRRARKVVNTHLLSVKMVYSKRHDREEEVRLVVGKIRELAAAAPGKALDMTELLGGYAS
DFVRRAVLGESHRKHGRNELFRELTEISASLLGGFNLEDYFPRLANLDVFLRVVCSKAMGVSKRWDNLFNELIAEYEGGKEDNAEDFVHLLLS
LKKEYGLSTDNVKAILVNMFEAAIETSFLVLEYSMAELINNRHVMAKVQKEVRESTPNGEKLDLIMEEDLSRMPYLKATIKEAMRIHPPAPFLLPH
FSTNDCEINGYTIPAGTRVIVNAWALARDPLHWERAEEFYPERFLQEGRDAEVDMYGKDIRFVPFGAGRRICAGATFAIATVEVMLANLIYHFD
WALPSEMEAIGAKVDMSDQFGMTLRRTERLHLVPKIYK
>Hordeum lecheri_Bx2
MAHVHVDEMLHGAAAAPRSLLIATAVLFSLVVLPLLLRIITKQGAASDAKLLSLLPSPPSKLPIIGHLHLMGDLPYVSLAGLAAKYGPELMLVRLGA
VPTAVVSSPRTAEAVLRTHDHVFASRPRSMVFDIIMYGQTDSCFAPYGDHFRKARKLVTVHMLNARKIRSQRPAREEEVRLVIGKIAKAAAARE
AVDMSELLHSYVNDLVCRAVSGKFSQEEGRNKLFRELTDINAALLGGFNILDYFPSLGRFELVCKVACAKARRVRKRWDLLLDKLIDDHAARM
VSREDEAQGEQEEDKDFIDVSLSLQQEYGLTRDHIKAILIDMFEAGTDTSYMTLEFAMAELIRKPHLLNKLQEEVRRNVPNGQEMVAEDDLPNM
TYLKAVIKETLRLHPPVPLMIPHFSLDACTVDGYTIPANTRVVINAWALGRHSSYWENENEFQPERFMNGAGVDLKPNEFHYLPFGFGRRMCP
GVHSASATVETMLANLMYRFDWKLPPGLKEEDIDMTEVFGITVSRKEKLILVPVTV
>Hordeum lecheri_Bx3
MALEAAYHYLQIAVGHGTSTPAALLTVLLLLIIRLAWVRTTTASTRLSKQQQLPPSPPGKLPIIGHLHLLGSQTHISIRDLDAKHGRNGLLLLRIGAV
PTLFVSSPSAAEAVLRTHDQIFASRPPSMAAAIIRYGLTDIAFAPYGEYWRQARKLLTTHMLSAKVVHSFRHGRQEEVRLVINKTREAATRGTAV
DMSELLSGYTNDVVCRAVLGESHRKEGRNRLFSELTEINVSLLGGFSLENYIPPNMIMADVLLRLVSVKAQRLNKRWDDLFNEIIEEHLHPSKPS
SGEQQAADFIDLLLSLKEEYGLTTDNIKAILVDMFEAGIETSYLTLEYGMAELMNNRHILTKLQEEVRSQGKKLDMITEEDVSSMAYLRATIKETS
RLHPPAPFLLPHFSTADCNIDGYVVPSNTRVLVNAWALGRDLSSWERPDDFLPERFLQDQAGDVDTQMRGKDLRFLPFGFGRRICPGMNFG
FATMEIMLANLMYHFDWDVPNMMGTGAGVDMAESFGLTLRRKEKLQLVPQIP

>Hordeum lecheri_Bx4
MALEAAYHYLQRAVGHGTTTEALLLTVLLLLIIRLAWVRAFTSTTTSTKFKQQLPPTPPGKLPIIGHLHLIGSHPHVTFRDLAAKHGRDGLMLVHV
GAVPTVVVSTPQAAEAVLRTHDHVFASRPRNPVADIIRYGSTDIAFAPYGDYWRRARKVVNTHLLSVKMVYSKRHDREEEVRLVVAKIHELAM
AAPGKALDMTELLGGYASDFVCRAVLGESHRKHGRNELFRELTEISASLLGGFNLEDYFPRLANLDVFLRVVCSKAMGVSKRWDNLFNELIAE
YEGGKEDNAEDFVHLLLSLKKEYNLSTDNVKAILVNMFEAAIETSFLVLEYSMAELINNRHVMTKVQKEVRESTPEGGKLDLIMEEDLSRMPYLK
ATIKEAMRVHPPAPFLLPHFSTNDCEVNGYTIPAGTRVIVNAWALARDPSHWERAEEFYPERFLQEGRDAEVDMYGKDIRFVPFGAGRRICAG
ATFAIATVEVMLANLIYHFDWELPSEMEAVGAKVDMSDQFGMTLRRTQRLHLVPKIYK
>Zea mays_Bx2_CYP71C4
MAAQLHHALYELLHEAAAAQRALLLAIPFSLLLLPLLLRYLAASASASATKNDGAAPASDPDKLLSLLPSPPMKLPIIGHLHLMGDIPYVSLAALAT
RYGPDLMLLRLGAVPTVVVSSPRVAEAVLRTYDHVFSSRPRSLVSDIIMYGATDSCFAPYGDHFRKARKLVTVHLLNASKVRSQRPAREEEVR
GALDRVRRAAAAREPVDMSELLHSFVNNLVCRAVSGKFSMEEGRNRLFRELTDINAGLLGGFHIQDYFPRLGRIELVRKVACAKTRRVRKRW
DDLLDKLIDDHAARMATHQDEDDDKDFIYVLLSLQKEYGLTRDHIKAILIDMFEAGTDTSYMTLEFAMTELIRKPHLMKKLQEEVRRNVPAGQEM
VTEDNLPGMTDLKAVIKETLRLHPPVPLLLPHYSLDACEVAGYTIPANTRVVVNAWALGRHSGYWERENEFVPERFLSGDVAGGVDLKPNEFQ
FLAFGSGRRMCPGVHSASATIEAMLSNLMYRFDWQLPAGMKAEDVDMTEVFGITVSRKEKLLLVPQAA
>Zea mays_Bx4_CYP71C1
MALEAGYDYLHVAVVQCTPTQAAAVLGVLLLLAIRLAAAARSSSATSPKWKQHRLPPTPPGKLPIIGHLHLIGSHPHVSFRDLHAKYGHNGLML
VQVGAVPTIVVSTPQAAEAVLRTHDHVLASRPRNPVADIIRYNSTDVAFAPYGVYWRTARKVVNTHLLSAKMVFSKRREREEEVRLVVARIRDA
AEASPGTALDMTELLGGYASDFVCRAVLGESHRKQGRNKLFRELTETSAALLGGFNVEDYFPKLADVDLFLRIICAKAKSVSKRWDSLFNELLS
EYALSGGKQGDHNSEDFVHLLLSLQKDYGLTTDNIKGILVNMFEAAIETSFLVLEYSMSELMNNRHVLAKLQKEVRTATPDGRMVMEEDLSRM
PYLKATIKESMRIHPPAPFLLPHFSTHDCEINGYTIPAGTRVIVNAWALARDPTCWDKAEEFFPERFLEQGRDAEVDMYGKDIRFVPFGAGRRIC
AGATFAIATVEIMLANLIYHFDWEMPAEMERTGAKVDMSDQFGMTLRRTQKLYLVPRIPKCVSSS
>Leonotis leonurus_LELE_c25490_g1_i1
MEIISFTTLVLLSSFIFILIKTLKKPKPAESGENLIPSPPKLPVIGHLHHLAGDLPHQALGKIYKKYGEIVSLKLGQTSAVIISSRELAKEVMKVHDLHF
ADRPNSISVEILWYNYSDLAFCPYGEEWRQMRKICMVEMLSPKNVKSFGYIREDEIITLTKTLRASSGEPVDFTETISSFNCSITCRAALGKVLG
DRDTLIPLIKTAVGMSGGFELADLFPSFKLLHLFSVRKYKLMGMRRKIDVILDRMVEDHKVKKSGEYDGEDIVDVLLRMQETGELKFPITTENIKA
IILDMFAAGTETVSTSMDWIMTELMRHPRVMAKLQEEIRGALKGKTRLEESDVQDLKYMKLVIKETMRLHPPVPIIPRQCREECQIGGYSIPLKT
RLIVNVWSLGRDPKYWNDPETFMPERFEGNSPDLLGHDFEFLPFGSGRRICPGLNFGLANVQFPLAQLLYHFDWKLPKGMGPNDVDMGELD
GLAVGRKTPLVLVPTPYVPSN
>Leonotis leonurus_LELE_c28478_g1_i1
MEIFQLIPLNFTTVVLISSFVFFLIKLIIKPKSPQNLPPAPPGLPVIGHLHHLAGGLVHEGLAKLSKKYGPIVSLNLGQVPAVAISSRELAKEVLKVLD
PACADRPESIALRTMFYGNTDIAFSPYNEYWRQMRKICILEMLSAKNVKSFGYIRQDETNNLVNSLKSVSGKAINLTERMFVFSCTVTCRATFG
NVMRDRDLFIVLVKKLAHMAGGMELADLFPSIKSLTVFTWNRFQLWKMKRGMDAILDPIVAEHRLKRSGEGDSEDIVDVLLRVQESGQLQFPIT
NDNVKAVILDMFAAGTESSATTTDWAMAELMRNPRVMARVQSEIRETLKGKTTVEESDVPKLTYLKLVIKETLRLHPAFPLLPRQCKDEFEVGG
YTIPVKTKVMINVWALARDPKYWEDAESFKPERFENNPIDFLGSDYEFLPFGAGRRNCPGLSFGIANLELPLAQLLFHFDWKLPQGMRPEDVD
MAAVEGLAVGRKTPLVLIPTVHNPSN
>Lamium album_LAAL_c18029_g1_i2
MEIISLTTLLLLSTFIFFLIKTIKKPKPSENEGKLPPSPPKLPLIGHLHHLAGDLPHHALSKISKKYGDIVSLKLGETNAVIVSSRELAKEAMKVHDLH
FADRPNSVSVDILWYNNSGLAFCPYGEEWRQMRKICMLEMLSPKNVKSFGYIREDEIINLVKSLRSLSSGEAVDFTETISSFNCSITCRAALGKV
LGDRDTLIPLIKTAVGMSGGFEVADLFPSFKLLHFLSVKKYMLPGMRRKIDAILDRMVEDHKVKKSGEYDGEDIVDVLLRMQETGELRFPVTTE
NIKAIILDMFAAGTETVSTSMDWIMTELMRHPRVMTKLQEEIRGALKGKTRLEESDVQGLKYMKSVIKETMRLHPPVPIIPRKCREECRIGGYSIP
LNSSLIINVWSLGRDPKYWNDPETFLPERFDENSLDMLGHDFEFLPFGSGRRICPGLNFGLTNVQFTLAQLLYNFDWKLPDGMKPSDVDMSEL
DGLAVGRKTPLLMVPTPYNHHS
>Leonurus cardiaca_LECA_c32589_g1_i2
MEMIQLSPFNVTSLLLLASFIFLLIKATKKPKPPINLPPSPPKLPVIGHLHHLAGELGHRALRKISQNYGPIVRLKLGEIESVVISSKEASKEVLKTHD
PAFADRPGSIAIKILWYNQTDLAFCAYDDYWRQMRKICILEMLSAKNVKSFGYIREDEISRLVESLQSRAGQTVNFSEKISAFMSSITCRAAFGKV
LGDRDTLIALVNTASSMAGGFELADFFPSFTLLHLFSWRKFTLLRMRRKIDAILDRIVEDHRLKRSGEYDGEDIVDVFLRIQNTGELKFPITTDNIK
AIILDVFAAGTETSSITVGWIMTELMRNPRVMAKLQQEIRGVLKGKMRLEESDVQGLKYMKLVIKESMRIHPPVPLIPRQCKDECEVGGYTIPVK
TRVMVNVWAMGRNSEYWDDPESFYPERFENVSTELLGNDFELTPFGAGRRICPGLNFGLANVEFPLAQLLYHFDWKLPEGMSPADVDTSEV
EALTVGRKNPLMLIPTPYNPTDNS
>Catharanthus roseus_CYP72A1v1
MEMDMYTIRKAIAATIFALVVAWAWRVLDWAWFTPKRIEKRLRQQGFRGNPYRFLVGDVKESGKMHQEALSKPMEFNNDIVPRLMPHINHTIN
TYGGNSFTWMGRIPRIHVMEPELIKEVLTHSSKYQKNFDVHNPLVKFLLTGVGSFEGAKWSKHRRIISPAFTLEKLKSMLPAFAICYHDMLTKW
EKIAEKQGSHEIDIFPTFDVLTSDVISKVAFGSTYEEGGKIFRLLKELMDLTIDCMRDVYIPGWSYLPTKRNKRMKEINKEITDMLRFIINKRMKAL
KAGEPGEDDLLGVLLESNIQEIQKQGNKKDGGMSINDVIEECKLFYFAGQETTGVLLTWTTILLSKHPEWQERAREEVLQAFGKNKPEFERLNH
LKYVSMILYEVLRLYPPVIDLTKIVHEDTKLGPYTIPAGTQVMLPTVMLHREKSIWGEDATEFNPMRFADGVANATKNNVTYLPFSWGPRVCLG
QNFALLQAKLGLAMILQRFTFDVAPSYVHAPFTILTVQPQFGSHVIYKKLES
>Salvia pomifera_CYP71BE52
MEIHIPSLVLCISFFIFFKIVSKLKTKTSNRKHLPLPPGPWKLPLIGNLHNLVGALPHHTLRRLSRKFGPMMSLQLGELSAVIISSADAAKEIMKTHD
LNFASRPQVAAADIIGYGSTNITFSPYGGHWRQLRKICTLELLSAKRVQSFRPLRERVFVDLCRRFADHGSSAVNFSEEFMSATYTLISRAVLGE
EAEQHEGLLPNVKEMPELTAGFDISEVFPSVGLFKVMSRLRKRIVAVHKDTDRILDDVIHQHRAAKSEEHKDLLDVLLQLQEDGLELPLTDENIK
SVLVDMLVAGSETSSTVIEWAMAEMLKNPRILEKAQEEVRRVFDKEGTVDESHIHELKYLKSVVKETLRVHPPAPLILPRICGETCEINGYEIPAE
TKIIVNAWAVNRDPKYWEDSDCFKPERFLDNLVDFRGNHFQYIPFGAGRRMCPGIGFGLANVELPLAMFMYHFDWELDGGMKPQDLDMEEK
FGASAKKLKDLFLIPAIKRTLPTK
>Jatropha curcas_CYP71D495
MLFFITVLFIFIALRIWKKSKANSTPNLPPGPNKLPLIGNVHNLVGDLPYHRLRDLSKKYGPIMHLQLGENTTVVISSPELAQEVMKTHDVNFAQR
PFVLAGDIVSYKCKDIAFAPYGEYWRQLRKMCSLELLTAKRVQSFKSIREEEVSKLVESISSSSGSPINFSKMASSLTYAIISRAVCGKVSRGEEV
FVPAVEKLVEAGRSISLADLYPSVKLFNALSVVRRRVEKIHGEVDKIIENIVIEHRERKRMAHAGINSKEEEDLVDVLLKFQENGDLDSYLSNDGI
KAVILDMFIAGSDTSSTTIEWAISEMVKNPSIMEKAQAEVREVFGSKGKVDEADLHELNYLKLVIKETLRLHPAVPLLLPRQSREDCVIEGYNIAT
KSTVIVNAWAIARDPKYWDEAERFYPERFINSSIDFKGTNFEFIPFGAGRRMCPGMLFGLASVELPLAQLLYHFDWKLPGGQKPEDLDMSDDL
DGTATRRHALYLTATPYLPSAVGKISR
>Nicotiana tabacum_CYP51G1

MELGDNKILNAGLLLVATLVVAKLISALIMPRSKKRLPPVIKSWPILGGLLRFLKGPVVMLREEYPKLGSVFTLNLLNKNITFFVGPEVSAHFFKAP
ETDLSQQEVYQFNVPTFGPGVVFDVDYTIRQEQFRFFTESLRVNKLKGYVDQMVMEAEEYFSKWGDSGEVDLKYELEHLIILTASRCLLGEEV
RNKLFEDVSALFHDLDNGMLPISVIFPYLPIPAHRRRDNARKKLAEIFANIIDSRKRTGKAESDMLQCFIDSKYKDGRATTESEITGLLIAALFAGQ
HTSSITSTWAGAYLLCNNKYMSAVVDEQKNLMKKHGNKVDHDILSEMEVLYRCIKEALRLHPPLIMLLRSSHSDFTVKTREGKEYDIPKGHIVAT
SPAFANRLPHVYKNPDTYDPDRFAPGRDEDKVAGAFSYISFGGGRHGCLGEPFAYLQIKAIWSHLLRNFEFELISPFPEIDWNAMVVGVKGKV
MVKYKRRKLSTE
>Sorghum bicolor_CYP71E1
MATTATPQLLGGSVPQQWQTCLLVLLPVLLVSYYLLTSRSRNRSRSGKLGGAPRLPPGPAQLPILGNLHLLGPLPHKNLRELARRYGPVMQLR
LGTVPTVVVSSAEAAREVLKVHDVDCCSRPASPGPKRLSYDLKNVGFAPYGEYWREMRKLFALELLSMRRVKAACYAREQEMDRLVADLDR
AAASKASIVLNDHVFALTDGIIGTVAFGNIYASKQFAHKERFQHVLDDAMDMMASFSAEDFFPNAAGRLADRLSGFLARRERIFNELDVFFEKVI
DQHMDPARPVPDNGGDLVDVLINLCKEHDGTLRFTRDHVKAIVLDTFIGAIDTSSVTILWAMSELMRKPQVLRKAQAEVRAAVGDDKPRVNSE
DAAKIPYLKMVVKETLRLHPPATLLVPRETMRDTTICGYDVPANTRVFVNAWAIGRDPASWPAPDEFNPDRFVGSDVDYYGSHFELIPFGAGR
RICPGLTMGETNVTFTLANLLYCYDWALPGAMKPEDVSMEETGALTFHRKTPLVVVPTKYKNRRAA
>Popolus trichocarpa_CYP71B63
MALYVVPLWLPLILLLALLLLFMKKMEVKRQSEQLLPPSPPKLPILGNLHQLGSLPHQSLWQLSKKYGPVMLIRLGRIPTVVISSAEAAREVLKVH
DLAFCSRPLLAGTGRLTYNYLDIAFSPYSDHWRNMRKVLTLELFSLKRVQSFRFIREEEVSLLVNFISESSALAAPVDLTQKLYALVANITFRMAY
GFNYRGTSFDRDKFHEVVHDTEAVAGSISADESIPYLGWIVDRLTGHRARTERVFHEVDTFFQHLIDNHLKPGRIKEHDDMVDVLLRIEKDQTE
LGASQFTKDNIKAILLNLFLGGVDTISLTVNWAMAELVRNPRVMKKVQDEVRKCVGNKGRVTESDIDQLEYLRMVIKETLRLHPPAPLLIPRETM
SHCKVSGHNIYPKMLVQINVWAIGRDPTYWKDPEEFFPERFLDSSIDYKGQSFEYLPFGSGRRICPGIHMGSITMEIILANLLYCFDWVFPDGM
KKEDINMEEKAGVSLTTSKKTPLILVPVNYLQ
>Popolus trichocarpa_CYP71B40v1
MDSLVQSLQASPMSFFLIAITSLFFLGLLSRLRRRLPYPPGPKGLPLVGSMHMMDQITHRGLAKLAKQYGGLFHMRMGYLHMVTVSSPEIARQ
VLQVQDNIFSNRPANIAIRYLTYDRADMAFAHYGPFWRQMRKLCVMKLFSRKRAESWESVRDEVDSMLKTVEANIGKPVNLGELIFTLTMNITY
RAAFGAKNEGQDEFIKILQEFSKLFGAFNMSDFIPWLGWIDPQGLSARLVKARKALDKFIDSIIDDHIQKRKQNNFSEDAETDMVDDMLAFYSEE
ARKVDESDDLQKAISLTKDNIKAIIMDVMFGGTETVASAIEWVMAELMKSPEDQKRVQQELADVVGLERRVEESDIEKLTFLKCALKETLRMHP
PIPLLLHETSEDAEVAGYFIPKQTRVMINAYAIGRDKNSWEDPDAFKPSRFLKPGVPDFKGNHFEFIPFGSGRRSCPGMQLGLYTLDLAVAHLL
HCFTWELPDGMKPSELDMTDMFGLTAPRATRLVAVPSKRVLCPL
>Arabidopsis thaliana_CYP71A12
MEMILMVSLCLTTLITLFLLKQFLKRTANKVNLPPSPWRLPLIGNLHQLSLHPHRSLHSLSLRYGPLMLLHFGRVPILVVSSGEAAQEVLKTHDLK
FANRPRSKAVHGLMNGGRDVVFGPYGEYWRQMKSVCILNLLTNKMVASFEKIREEELNEMIKKLEKASSSSSSENLSELFVTLPSDVTSRIALG
RKHSEDETARDLKKRVRQIMELLGEFPIGDYVPALAWIDRINGFNARIKEVSQGFSDLMDKVVQEHLEAGNHKEDFVDILLSIESEKSIGFQAQR
DDIKFMILDMFIGGTSTSSTLLEWIMTELIRNPNVMKKLQDEIRSTIRPHGSYIKEKDVENMKYLKAVIKEVFRVHPPLPLILPRLLSEDVKVKGYNI
AAGTEVIINAWAIQRDPAIWGPDAEEFKPERHLDSTLDYHGKDLNFIPFGSGRRICPGINLALGLVEVTVANLVGRFDWRAEAGPNGDQPDLTE
AFGLDVCRKFPLIAFPSSVI
>Arabidopsis thaliana_CYP71B15
MSVFLCFLVLLPLILIFLNVLKPSKYKLPPGPKKLPIIGNLHQRRTLHPRNRRNLAEMYGPVALLQYGFVPVVAISSKEAAEEVLKINDLECCSRPE
AAGMRATFYNFKDIGMAPFGDEWSLMRKLSVVELFSVKKLQSFKYIIEEENNLCVKKLSEFATRQSPVNLERAIFTLVGNIVCRIGYGINLYECDF
FEADRVVDLVLKAEAVIRETVFSDFFPGRIGRFIDCISGQNRRLKNNFSVVDTFFQNVLNEHLKPGRESSTIVDLMIDMKKKQENDGDALKFTTD
HLKGMISDIFVAGIGGVAGITLWGMTELIRNPRVMKKVQDEIRTTLGDKKERIKEEDLNQLHYFKLVVKETLRLHPTTPLLLPRQTMSHIKIQGYD
VPAKTQILVNVYAMGRDPKLWENADEFNPDRFLDSSVDFKGKNYEFIPFGSGRRICPGMTMGTILVEMALLNLLYFFDWGLAKQEEAKEIINGE
ENFLAFFQVLHH
>Phalaenopsis equestris_XP_020599078.1
MDPMLLAALFLIPLFFLLVEQSKRKSISKKTPHPPSPSKLPFIGNLHQLGLLPHRSLFELSKKYGPLMLLHLGSIPTLVVSSPSSAQEILRTHDLVF
ASRPSLKASRILLYNNNDMAFAPYGEYWRQIRKLCVTNLLSLKMIKSFSLVRMEEVSLMIEGISRKASTNNGVVCMSEILNSLTCDVLCKSLFGS
SVILEKRKLLCELIRKNVAFFQEASLEDYFPRLRWLDVLFGLEGRLRRHSEKWDALLGDLIQDHIRQLNEGQSTSSTDFIDVMLQLQKNSGLEFV
LTNEQIKAILMDMIAAGTDTSFAVLEWSMAELIRNPNVMAKVQNEVREIAHGKEIVGEELLDKLNYMRAVIKEVLRLHPSAPLLLPHESLHDCQIQ
GYHISKETRVLINAWAIGRDEVYWKEAQDFKPERFLSSDVDYKGNDFHYIPFGSGRRICPGMQFAVAIIEISLANLLHRFNWTLPSGISYESLDM
QEAAGITTPRKEKLELVPLAVKIF
>Dendrobium catenatum_XP_020696583.1
MEKGTTSSTPGAWGAPPSNTAVSLKGRSKRKSISKKIPLIPSPPGLPFIGNLHQIILLPHRSLFELSKKHGPLMLLQLGSIPTLVVSSPSTTREIMR
THDLVFASWPSLKASRILLYNNNDMGLAPYGEYWRQTRKICVTNLLSIKMIQSFSLMRMEEVSLMIERISRMASANNGVVCVSEILNKLTRDVLC
KSVLGSSLSEERRKLLCELIRMNSIFFGKFFLEDYFPQLRWLDVLFGLERELIRHSKKWDALLGEFVEEHVSQLAEGSTYFIDVLLQLQKDPSMP
LVLTNEKIKALLLDMIVAGTETSLALLDWCMVELIRNPMVMEKVQSEVRRIAHGEEIVREEYLGKLSYMKAVIKEVLRLHPPASLLLPRVSLQDCQ
IQEYNIPKKTRVLINAWAIGRDEDYWKDPQEFKPERFLGDDVDYKGNDFHYIPFGASRRICPGIQFAISIIELALANLVYRFNWKLPSGISCDGMD
MEDGAGLTTPRKEKLYLVPLAI
>Aphelandra squarrosa _Bx5
MEGSLLYTIITAALVIIFVLFLSHRRRSRGYQLPPSPAAALPIIGHLHLLKEPLHQTLQRFSEACGPIFLLRLGVRKVVVVSSPELLEECYTAYDVAL
SNRPRIMSDKYIGYDHTTMAGAPYGDRWRSLRRLAAQEVLSTARINSFTEIRHDEAKRTLQSMVSRVGESGKLQLSLRPELYDLIFNVIMRMLT
GKRYIKGEYLSHDRLADGYREIMSEFFENAQTSNPEDFLPFLKWVDYRGLKKKMASLGGRLDNFYQSLLQEHRQEKRNTIIGHLLSLQESDPQ
FYDDQLVKGFMTNMIVGSLDTSVVTIEWAMCLLLNNPNIFKTARQELDSQIGLDRLVEEQDLSNLPYLHGIIMETLRMFPPGPLMLPRESSTDCV
IGGYDVPNGTIVLVNAYAIQRDPKSWDDPASFKPERFIGKDVELRKMLPFGVGRRSCPGSVLAHRMLGLILGSLIQCFDWERMNSEKVDLTEG
AGLTLPKLQPLEAICKPRVSMVKVIQESDNVD
>Lamium galeobdolon_Contig_957
MEVFLLYFSLALCLIFLIAKHFTRKHSRLPPSPRPALPILGHLNLLKPPLHRTFHRLSQTHGPIFSLKLGVRRAVVVSSPNLVEECFTVNDTVFSNR
PWVMVDKYIGYNHTTIAGAPYGQQWRNLRRLGAQEVLSASRLNSLSEIRQDEMRRTLQTLTQSEEFEKLQLRPKLFELIFNLIMRIVAGKRYST
GKKDDQQLGERIREMVSEVFEHAQSSNPEDFLPFLKWIDYRGLKKNLTNLGKKLDDFYQSLLEEHRKEKRNTIIGHLLSLQQSDPDFYTDQTIK
GFITNMIIGGTDTSVVTIEWAMSLLLNHPDVLQKAKQELDSQVGHQRLVEEKDLPNLHYLQNIISETFRLFPAGPLGVPRESSADCRVGGYDIPR
GTILLVNAWAIHRDPDVWDEPMSFKPERFEGREVETQTLMPFGMGRRGCPGAGLGQRMVGLCLGSLIQCFEWERVSLEEIDLAEGVGLTMP
KLKPLEAMCKPREIMLKTLRGATNFTT
>Lamium galeobdolon_Contig_7774
METNFLYALFFFFFFIFLVMAFNYSSKRKLPPSPRPTLPLLGHLHLLKFPVHRTYHNLSLKLGPIFSLQLGSRLVVVVSSPAIVEECFTKNDVVLA
NRPRFIIGKYTGYNYSSLVAAPYGDYWRNLRRLTTMEIFSNSRLSLFQSIRHDEVTLMLKKLYGKSCRVELRPLLSELTFNNIMRMVCGKRYFG

VDEGNDEANEFRRLIDEVFARSGSNPADFFPLFRWIDYKDFEKNMKRVSERMDVFLQGLIDEHRRRKGGNTMIDHLLSLQDSEPESYTDVIIKS
LIMVMLLAGTDTSSVTIEWAMSALLNHPEKLEKARAEVDNIVGDNRLVDESDLQKLPYLHNIISETMRLYPAAPLLVPHQASADIKLAGYDVPRG
TILLVNAWAVHRDPAVWDDPTSFKPERFEAGEFGPSKLLPFGMGRRSCPGNGLAQRVVGLAIASLIQGFEWQRVDEDLVDLTEEKKGLTMRK
KIPLEATCKARRVLHKVLAAGED
>Lamium maculatum_Cluster-9204.2659
MESQIILYTSLITLVISMIIIHKIVSQKKACPNLPPSPPGWLPIVGHIHLLKNLLHRKLYAFSLKVGPIFSVRFGRRLVVVVSSSALAEECLNENDLVL
ANRPIASVDRSSLGYSTTTVIGAPYGPHWRNLRKLCDIEVFAPTRLASFVWVRRDERDRMISRLFEMSSSSGEGLRKVNVECMIVELMFNNIM
RMVAGKRYYGEEAEDDEEAKEFREITREALELTSASGLGEVFPALRWVGCNEVEKKLKSHRRKMDEFMQGLIDEHRRRKRQNTMVDHLLSL
QESQPQYYTDEIITGIIVALIIAGTDASVVTTEWMMTLLLNHPNVLTKAREEVDTVVGHDRLVEEHDLPKLAYLHCIVLETLRLFPSVPLLVPHVPS
QDVRIGGYDIPKDTMVLINAWAIHRDPKVWHNPLSFEPERFETMEVETHKLLPFGMGRRACPGAGLAHKFVGLAVGSLIQCFDWERPSADKID
MTEGSGTTLPKAKILEALCKPRKVVQKLLQQLPNHAS
>Lamium orvala _Cluster-13930.0
MVMEMEVSLLYIFVALCLIFVIVIAKRFTRKHSRLPPSPRPALPILGHLHLLKPPLHRTFHRLSQTHGPIFSLKLGVRRVVVVSSPNLVEECFTLNDI
VFSNRPWVMVDKYIGYNHTTIAGAPYGQQWRNLRRLGAQEVLSASRLNSLSEIRQDEMRRTLQTLTQSEEFEKLQLRPKLFELIFNLIMRIVAG
KRYSTGKGDDEQLGERIREMVSEVFEHAQSSNPEDFLPFLKWIDYRGLKKNLTNLGNKLDDFYQSLLEEHRKEKRNTIIGHLLSLQQSDPNFYT
DQIIKGFITNMIIAGTDTSVVTIEWAMSLLLNHPNVLQKAKQELDSQVGHQRLVEEQDLPNLHYLQNIISETFRLFPAGPLVVPRESSADCRVGGY
DIPRGTILLVNAWAIHRDPDVWDEPMSFKPERFEGREVETQTLMPFGMGRRACPGAGLGQRMVGLCLGSLIQCFEWERVCLEEIDLAEGVGL
TMPKLKPLEAMCKPREIMLKALRGATNFTT
>Sesamum indicum_XM_011101018.2
MELITLIYTSLALFSIFLLLKYLWPENYRLPPTPAPALPIIGHLHLLSPPLHRTLQRFSQTHGPIFSLKLGSRRVVVVSSPELVEECFTTNDIILSNRP
RILADKYVGYNHSTTVGANYGALWRRLRRLGAQEVLSSARVNAFSWIRQDEMRRTLQSLVMNYDGNESVKLDLRPKLFDLIFNIIMRMLAGKR
FCSTEGEHEQSGEQWREVISEVLENAEATNPEDFLPFLLWIDYKGLKKKMAGLAEKMDGFYNGLMDEHRQEKRNTILGHLLSLQESDPEFYT
DQLIKGFMANTIIAGTDTSAVTIEWAMSLLLNHPEVLQKAKLEIDSRVGHQRLVEEQDLPNLPYLHSIIFETFRLFPAGPLVVPRESSADCRVGGY
DIPCGTMLLVNAWAIHRDPKVWPQPTSFKPERLDRREVETHKLMPFGLGRRACPGAGLGQRMVGLALASFIQCFEWERVSSEEVDLTEGVGL
TMPKLKPLEAKCRPRQIMLKVLQESANFTS
>Achantus ilicifolius_TRINITY_DN1412_c0_g1_i3
MDLFTVGAAVLVAGVLFFRYRSRSRGYRLPPIPVPPLPIIGHLHLLKEPLHQTLQKYSHACGPIFSLKLGFRRVVVVSSPELLEECYTSHDVALS
NRPRIMSDKYIGYDHTTMAGAPYGPRWRSLRRLAAQEILSPTRINMFTQIRQDENNRKMRSMAAEAGKAVEIRPVLYDLIFNVIMRMLTGKRYT
KGEYLSHDRLADGYREIMSEFFENAQTSNPEDFLPFLKWIDYRGLKKKMAGLGSKLDNFYQSLLQEHRQENRNTTIIGHLLSLQDSHPQFYDD
QLVKGFMTNMIVGSLDTSVVTIEWAMCQLLNHPDIFRAAREELDSQVGLDRLIEEKDIPNLPYLHSIIMETLRMFPPGPLMLPRETSVDCTIGGY
NVPNGTIVLVNSYAIQRDPKYWDDPSCFKPDRFFGKEVELRKMLPFGVGRRSCPGSALAHRMLGLILGSLIQCFDWERMNQEKIDLTEGAGLT
LPKLKPLVAICKPRAEMLKILQESSNDD
>Achantus ilicifolius_TRINITY_DN17591_c0_g1_i3
MEEVAIVTATISCLFLILLVYLRYVSRRRPYRLPPSPAPSFPILGHLYLLKEPLHRTFQRFSQAHGAIFSLKLGSRRVVVVSSPDLVSECFTTNDVV
LSNRPRVLVDNYIGYGHTTVAGEPYGPQWRSLRRLAAQEVLSPARLAATARIRHDETCRMLRNLIHCQESPKFELRTKLFELIFNVIMRMLTGR
RFSTNELEGDQLGEQFREMVSEVFEHAQASNPEDFLPFLLWIDYRGLKKKMAALATRLDNFYQSLLEEHRREKRNTIIGHLLSLQGTHPEFYTD
KIIKGFMTNMIIAGTDTSVVTIEWAMSLLLNHPEVLDKARLELDSQVGHHRFVEEQDLPNLHYLHSIIWETFRLFPPGPLVVPRESSSDCKIGGYD
VPRGTILLVNAWAIQRDPNVWDDPTTFRPERFEGKEMESQKLMPFGVGRRACPGSGLGQRMVGLALGSLIQCFQWKSVDSEEVDLAEGVGL
TMPKLRPLEVVCRTRHA
>Achantus leucostachyus_TRINITY_DN1357_c0_g1_i5
MDATLLFTVAAAVFLAGLLFWRYRSSASRGYNLPPSPVAPLPIIGHLHLLKEPLHQTLQHYSDAFGPIFSLKLGFRRVVVVSSPDLLEECYTSHD
VALSNRPRIMSDKYIGYDHTTMAGAPYGPRWRSLRRLAAQEILSPARINMFTQIRHDENNRKMRSMAVEAAGGGKAVEIRPVLYDLIFNVIMRM
LTGKRYTKGEYLSHDRLADGYREIMSEFFENAQTSNPEDFLPFLKWIDYRGLKKKMAGLGAKLDNFYQSLLQEHRQEKRNTIIGHLLSLQESDP
EFYDDQLVKGFMTNMIVGSLDTSVVTIEWAMCQLLNHPDIFRAAREELDSRVGLDRLVEEQDIPNLPYLHGIIMETLRMFPPGPLMLPRETSVD
CVIGGYDVPNETIVLVNSYAIQRDPKHWDEPSSFKPERFVGKDVEIRKMLPFGVGRRSCPGSALAHRMLGLILGSLIQCFDWERMNKEKVDLT
EGAGLTLPKLKPLVAICKPRAEMLKILLESSNID
>Achantus leucostachyus_TRINITY_DN1357_c0_g1_i6
MNELGDSTQIKQIEGRWRSNMDVPLLFTVAAAVFAAGLLFWRYRSRSRDYHLPPSPVAPLPIIGHLHLLKEPLHHTLQHYSNACGPIFSLKFGF
RRAVVISSPELLEEWYASHDITLSNRPRVMSDKYTGYDHTTINGAPYGPRWRSLRRMATQEVLSQANMNMFAQIRYDENKKTMRSMAAEAAA
GRAVEIRPVVYELIFNIVMRMLTGKRYGKQEYLSQYRDSMSDFFENAQTSNPEDFLPFLKWIDYRGLKKKLIRLGANLDNFYQSLLQENNQDKN
TIIGHLLSLQKSDPEFYDDRLIKGFMTIMVSGSLDTSVVAIEWIMSQLLNYPDIFRAAREELDSQIGLDRLIEETDIPNLPYLNAVIMETLRLFPPAPL
LLPRESSVDCVIGGYDLPKGTIVLVNAYAIQRDPKHWDMPLSFKPERFIGKDVDMCKMLPFGVGRRACAGSTLARRAVGLIIGSLIQCFDWETV
NKEKVDLTEGVGLTLPKLKPLVAICKPRAEMLKFLQESSNAN
>Achantus hungaricus_TRINITY_DN7587_c0_g1_i2
MDAALLFTVAAAVVLAALLFWRYRSRSRAYHLPPSPVPPLPIIGHLHLLKEPLHQTLQQYSHACGPIFSLKLGFRRVVVVSSPELLEECYTSYDV
ALSNRPRIMSDKYIGYDHTTMAGAPYGPRWKSLRRLAAQEILSPARINMFSQIRHDENNRKMRSMAVDAAAAGGAKAVEIRPVLYDLIFNVIMR
MLTGKRYTKGEYLSHDRLADGYREIMSEFFENAQTSNPEDFLPFLKWIDYRGLKKKMAGLGAKLDSFYQSLLQEHRQEKRNTIIGHLLSLQESD
PEFYDDQLVKGFMTNMIVGSLDTSVVTIEWAMCQLLNNPDIFRAGREELDSRIGVDRMIEEQDIPNLPYLHGIIMEALRMFPPGPLMLPRETSAD
CVIGGYDVPNGTIVLVNSYAIQRDPKYWDEPSSFKPERFVGKDVELRKMLPFGVGRRSCPGSALAHRMLGLILGSLIQCFDWERENQEKVDLT
EGAGLTLPKLKPLVAICKPRAEMLKILKESSNVD
>Achantus hungaricus_TRINITY_DN7587_c0_g2_i1
MEEMALVTTTISCLFLILLLYLKYVSHRRPYRLPPSPALPLPILGHLHLLREPLHRTFHRFSQTHGPIFSLKLGSRRVVVVSSPDLVSECFSTNDV
VLSNRPRVLVDKYIGYGHTTVAGEPYGRQWRSLRRLAAQEVLSPARLGATARIRHDETRRMLKTLVHCQGSPKFELRPKLFELIFNIIMRMLTG
RRFSTEEPDGDQLGERFREMVSEVFEHAQASNPEDFLPFLLWIDYRGLKKKMAALAAELDNFYQSLLEEHRREKRNTIIGHLLSLQETDPEFYT
DQIIKGFITNMIIAGTDTSVVTVEWAMSLLLKHPDVLDKATLELHSQVGHDRFLEEQDLPNLPYLNTIISETFRMFPPGPLLVPRESSTHCKIGQY
DVPPGTMLLVNAWAIQRDPDVWDHPTHFRPERFDQQKKNETTLLLMPFGVGRRACPGAGLGQRLVGLALGSLIQCFEWSAAEEVDLAEGVG
LTMPKLKPLEVVCRAREHMLHLISRAEGGIN
>Avicennia officinalis_TRINITY_DN10838_c0_g1_i2
MELTLLYASCALFVVSVILIKPLSRKRGYRLPPTPALALPIFGHIHLLKQPLHRTFHRFSQTLGPIFSLKLGVRRLVVVSSPDLVEECYTTNDVILCN
RPRVLVDKYIGYNHTTIAGAPYGHLWRRLRRLGAQEVLSPARLSAFSQIREDEIRRTLQSLIYRRETEKLELRPMVFELIFNVIMKLLAGKSCSGD
EKGDDELGERFREMVSQVFEHAQSSNPEDFLPFLLWIDYRGLKKNMAALGEKLDKFYQSLLEEHRVEKRNTIVGHLLSLQESDPEFYTDQIIKG
FMTNMIIAGTDTSAVTIEWAMSLLLNHPDVLQKAKLELDSQVGRHRLVEEQDLPNLHYLRNIIDETFRMFPAGPLLLPRESSADCKIGGYDVPQG

TILLVNAWAIQRDPRVWDDPTCFRPERFEGKDVETQKLMPFGMGRRACPGSGLGQRMAGLALGSLIQCFEWERVSSEEVDLAEGVGLTMPK
LKPLEAMCRPREAMLKVLQDLPN
>Aphelandra aurantiaca_TRINITY_DN3965_c0_g1_i1
MEGSLLYTITAALVVIFLLFLRHRSRSRGYHLPPSPAALPIIGHLHLLKEPLHHTLQRFSEACGPIFLLRLGVRKVVVVSSPELLEECYTAYDVAL
SNRPRIMSDKYIGYDHTTMAGAPYGDRWRSLRRLAAQEVLSTARINSFTQIRHDEAKRTLQSMVSRVGESGKLQLSLRPELYDLIFNVIMRMLT
GKRYIKGEYLSHDRLADGYREIMSEFFENAQTSNPEDFLPFLKWIDYRGLKKKMASLGSRLDNFYQSLLQEHRQEKRNTIIGHLLSLQESDPQF
YDDQLVKGFMTNMIVGSLDTSVVTIEWAMCLLLNNPNIFETARQELDSQIGLDRLVEEQDLSNLPYLHGIIMETLRMFPPGPLMLPRESSTDCVI
GGYDVPNGTIVLVNAYAIQRDPKSWDNPSSFKPERFVGKDVELRKMLPFGVGRRSCPGSVLAHRMLGVILGSLIQCFDWERVNSEKVDLTEG
AGLTLPKLQPLEAICKPRVSMLKVIQESSNVD
>Aphelandra aurantiaca_TRINITY_DN17332_c0_g1_i1
MEVTLLHAIAIIFTFLTILRHVLRSRDYRLPPSPALALPILGHLYLLTKPPLHRTLQRFSHTLGPIISLQFGVRRIVVVSSYDAAEECFTTHDVALANR
PSFLAIKYLGYDFTTIASSSFGHHWRSLRRLGAQEMFSTARLNAFSELRHEVVKKAMQNLVASTKENGSKLELRPKLGALIFNVVMRMLAGKQ
YFDNDNSNGLSGHRFQEMMVEFFQYMETSNPEDFLPFLLWIDYRGLKKKMVALAQRLDDYFQSLVDEHRRDKLNTVLGHLSSLQESKPDSCT
DLTMKGFINNVLLAGTESSATTIEWAMSLLLNHPEVLEKVKIEIDSKVGHQRLIEEQDLPNLLHLQSIILETLRMFAPGPLGLPHESSTDCKIGGYD
VPRGTILMVNVWAIHRDPKVWDEPNSFKPERFQGKEFETHRMLPFGMGRRACPGSGLGLRMVGLALGSLIQCFEWERVSSEKVDLTEGAGL
TMPKLKPLEAMVKPREAMLKALHESVA
>Arabidopsis thaliana_CYP81D1
MSLRLGSRLVYVVSSHKVAAEECFGKNDVVLANRPQVIIGKHVGYNNANMIAAPYGDHWRNLRRLCTIEIFSTHRLNCFLYVRTDEVRRLISRL
SRLAGTKKTVVELKPMLMDLTFNNIMRMMTGKRYYGEETTDEEEAKRVRKLVADVGANTSSGNAVDYVPILRLFSSYENRVKKLGEETDKFLQ
GLIDDKRGQQETGTTMIDHLLVLQKSDIEYYTDQIIKGIILIMVIAGTNTSAVTLEWALSNLLNHPDVISKARDEIDNRVGLDRLIEEADLSELPYLKN
IVLETLRLHPATPLLVPHMASEDCKIGSYDMPRGTTLLVNAWAIHRDPNTWDDPDSFKPERFEKEEEAQKLLAFGLGRRACPGSGLAQRIVGL
ALGSLIQCFEWERVGNVEVDMKEGVGNTVPKAIPLKAICKARPFLHKIIS
>Lotus japonicus_CYP81E6
MDIISFLYYSLFYVALFAIIKLFLGSRKFKNLPPGPTSLPIIGNLHHLKRPLHRTFRALSEKYGDVFSLWFGNRLVVVVSSFADVQECFTKNDVVLA
NRPRFLSGKYIFYNYTTLGSTSYGEHWRNLRRITSLDVLSNHRINSFSPIRRDETTRLIRKLAEDSAKNFSEVELTSRFFDMTFNNIMRMISGKRY
YGEDCDMTELQEASEFRDMVTELLQLSGANNKADFMPILRLVDFEGLEKRVKGISSKTDRFLRGLLQEHRDKKQRTANTMIDHLLTLQESQPE
YYTDQIIKGLALAMLLAGTDSSAVTLEWSMCNVLNYPEVLEKIKAELDTHVGQDRLVDESDIPKLTYLKNVINETLRLYTPAPLLLPHSASDDCTI
GGYKVPRDTIVLINAWALHRDPQLWTEATTFKPERFDKKGELEKLIPFGLGRRACPGELLAIRAISMTLALLIQCFDWKRVSDEEIDMGERDGFV
LMKSIPVKAMCKSRPVINNVFK
>Arabidopsis thaliana_CYP81F1
MLYFILLPLLFLVISYKFLYSKTQRFNLPPGPPSRPFVGHLHLMKPPIHRLLQRYSNQYGPIFSLRFGSRRVVVITSPSLAQESFTGQNDIVLSSR
PLQLTAKYVVYNHTTVGTAPYGDHWRNLRRMCSQEILSSHRLIIFQHIRKDEILRMLTRLSRYTQTSNESNDFTHIELEPLLSDLTFNNIVRMVTG
KRYYGDDVNNKEEAELFKKLVYDIAMYSGANHSADYLPILKLFGNKFEKEVKAIGKSMDDILQRLLDECRRDKEGNTMVNHLISLQQQQPEYYT
DVIIKGLMMSMMLAGTETSAVTLEWAMANLLRNPEVLEKARSEIDEKIGKDRLIDESDIAVLPYLQNVVSETFRLFPVAPFLIPRSPTDDMKIGGY
DVPRDTIVMVNAWAIHRDPEIWEEPEKFNPDRYNDGCGSDYYVYKLMPFGNGRRTCPGAGLGQRIVTLALGTLIQCFEWENVKGEEMDMSE
STGLGMRKMDPLRAMCRPRPIMSKLLL
>Sesamum indicum_CYP81Q1
MEAEMLYSALALTFAIFMVYRILSNSQDKRSLTKLPPSPPGWLPVIGHAHLMKNLLHRTLYDFSQKLGPIFSIRFGSRLVVVVSSSSLVEECFTKY
DIVLANRPQASVDRRSLGFSTTSVIGAPYGDHWRNLRKLCDLEVFAPTRLASFLSIRLDERDRMISALYKISSAGFAKVNLEAKIVELTFNNIMRM
VAAKRYYGEEAEDDEEAKRFRDLTKEALELTSASNPGEIFPILRWLGCNGLEKKLAVHSRKTDEFMQGLLDEHRRGERQNTMVDHLLSLQES
QPEYYTDEIITGLIVALIIAGTDASVVTTEWAMSLLLNHPKVLEKARKELDTLVGHERMVDEHDLPKLRYLHCIVLETLRLFPSVPTLVPHEPSEDC
KIGGYNVPKGTMVLVNAWAIHRDPKVWDDPLSFKPDRFEIMEVETHKLLPFGMGRRACPGAGLAQKFVGLALGSLIQCFDWERTSPEKIDLNE
GSGITLPKAKTLEAMCKPRHVMEKVLRQVSNV
>Sesamum indicum_XM_011101018
MELITLIYTSLALFSIFLLLKYLWPENYRLPPTPAPALPIIGHLHLLSPPLHRTLQRFSQTHGPIFSLKLGSRRVVVVSSPELVEECFTTNDIILSNRP
RILADKYVGYNHSTTVGANYGALWRRLRRLGAQEVLSSARVNAFSWIRQDEMRRTLQSLVMNYDGNESVKLDLRPKLFDLIFNIIMRMLAGKR
FCSTEGEHEQSGEQWREVISEVLENAEATNPEDFLPFLLWIDYKGLKKKMAGLAEKMDGFYNGLMDEHRQEKRNTILGHLLSLQESDPEFYT
DQLIKGFMANTIIAGTDTSAVTIEWAMSLLLNHPEVLQKAKLEIDSRVGHQRLVEEQDLPNLPYLHSIIFETFRLFPAGPLVVPRESSADCRVGGY
DIPCGTMLLVNAWAIHRDPKVWPQPTSFKPERLDRREVETHKLMPFGLGRRACPGAGLGQRMVGLALASFIQCFEWERVSSEEVDLTEGVGL
TMPKLKPLEAKCRPRQIMLKVLQESANFTS
>Salvia splendens_XM_042174890.1
MEVSPLYYTCLALFLVILTFKLFNPKTHRRLPPAPSPPLPFVGHLHLLAPPLHRTFHRFSLTHGPIFSLKLGVRRVVVVSSPDLVDECFTRNDVVF
SNRPRVLVDKYIGYDHTTIAGAPYGPHWRGLRRLGAAEALSPARLNALSEARQDETRRALSRLAAAAAGGGGFSAVELRPRIFDVIFSIIMRTLA
GEKYSSSGAGEGEFGGEIQEMVSEVFESAQSSNPEDFVPILKWIDYRGLKRKLSELGKRLDDFYEELLAEQRRERRSDTVIGHLLAMQESDSQ
FYTDQTIKGFITNMIVAGTDTSVVTIEWAMSLLLNHPNVLEIARLELDSKVGPHRLVEERDLPNLNYLQNIISETFRMFPAGPLVVPRESSADCRV
GGYDIPSGTILLVNAWAIHRDPDVWEEPMRFKPERFEGGREVPTEKLLPFGMGRRACPGAGLGRRMVGLALASLLQCFDWERMGPDQVDLA
EGVGLTMPKLKPLEAMCRPRQIMLEAISTRNVTD
>Helianthus tuberosus_CYP81B1c
MEIPYLLTTTLLLLFTTLYLLLRRRSSTLPPTIFPSLPIIGHLYLLKKPPLYRTLAKLSAKHGQILRLQLGFRRVLIVSSPSAAEECFTKNDIVFANRPK
MLFGKIIGVNYTSLAWSPYGDNWRNLRRIASIEILSIHRLNEFHDIRVEETRLLIQKLLSACNSGSSQVTMKFSFYELTLNVMMRMISGKRYFGGD
NPELEEEGKRFRDMLDETFVLAGASNVGDYLPVLSWLGVKGLEKKLIKLQEKRDVFFQGLIDQLRKSKGTEDVNKKKTMIELLLSLQETEPEYY
TDAMIRSFVLVLLAAGSDTSAGTMEWVMSLLLNHPQVLKKAQNEIDSVIGKNCLVDESDIPNLPYLRCIINETLRLYPAGPLLVPHEASSDCVVG
GYNVPRGTILIVNQWAIHHDPKVWDEPETFKPERFEGLEGTRDGFKLLPFGSGRRSCPGEGLAVRMLGMTLGSIIQCFDWERTSEELVDMTE
GPGLTMPKAIPLVAKCKPRVEMTNLLSEL
>Medicago truncatula_CYP81E7
MGILSYLCYSLFYLSIFFIIRLLFQSRKFKNLPPGPTSLPIIGNLHHLKRPLNRTFKALTEKYGNVISLWFGSRLVVVVSSLSEFQECFTKNDVVLAN
RPRFLSGKYIFYNYTTLGSTSYGEHWRNLRRITSLDVLSNHRINNFAPIRRDETQRLIKKLAEDSSTKFAEVELTFRFFDMTFNNIMRMISGKRYY
GDDCDISEVQEASQFRDMVSELLQLSGANNKTDFMPLLKFLDFENLEKRVKRIGEKNDVFLSGLLQEQRSKKERTNTMIDHLLNMQESQPEYY
TDTIIKGLCLAMLLAGTDSSAVTLEWTMSNILNYPEVLKKVRDEVDTHVGQDRLVDESDLPKLTYLRNVIYETLRLYTPAPLLLPHSTADECIMGG
YKVPRDTIVLINAWAIHRDPETWSEATTFKPERFDKKGELEKMIAFGMGRRACPGEGLALRAISMTLALLVQCFDWKRINDEKIDMSERDGFTM
TKLLPLKAMCKTRPVVNKVFK
>Arabidopsis thaliana_CYP81F4

MFNYVIILPLALFLLAYKFFFTSKRQRYHLPPSPSYSLPILGHHLLIKPPVHRLFHRLSNIHGPIFYLRLGSRRAVVISSSSLARECFTGPNDVIVSN
RPRFLTSKYIAYNYTTIATTSYGDHWRNLRRICSLEIVSSKRLANFLHIRKEEIQRMLTRLSRDARVGKEVELESVLYDLTFNNIVRMVTGKIYYG
DYVSDKEEAELFKKLFTFITTNSGARHPGEYLPFMKIFGGSFEKEVKAAAKVIDEMLQRLLDECKSDKDGNTMVNHLLSLQQDDPEYYTDIIIKG
LMLGIMVASSETSALTIEWAMASLLNHPKVLEKVKLEIDEIIGQDRLIEESDIANLPYLQNVVSETLRLHPAAPVLVPRSTAEDIKIGGYDVPRDTM
VMVNAWAIHRDPDLWTEPERFNPERFNGGEGEKDDVRMLIAFGSGRRICPGVGLAHKIVTLALGSLIQCFDWKKVNEREIDMSEGPGMAMRM
MVPLRALCKTRSIMNKLPAYTKV
>Nicotiana tabacum_CYP51G1
MELGDNKILNAGLLLVATLVVAKLISALIMPRSKKRLPPVIKSWPILGGLLRFLKGPVVMLREEYPKLGSVFTLNLLNKNITFFVGPEVSAHFFKAP
ETDLSQQEVYQFNVPTFGPGVVFDVDYTIRQEQFRFFTESLRVNKLKGYVDQMVMEAEEYFSKWGDSGEVDLKYELEHLIILTASRCLLGEEV
RNKLFEDVSALFHDLDNGMLPISVIFPYLPIPAHRRRDNARKKLAEIFANIIDSRKRTGKAESDMLQCFIDSKYKDGRATTESEITGLLIAALFAGQ
HTSSITSTWAGAYLLCNNKYMSAVVDEQKNLMKKHGNKVDHDILSEMEVLYRCIKEALRLHPPLIMLLRSSHSDFTVKTREGKEYDIPKGHIVAT
SPAFANRLPHVYKNPDTYDPDRFAPGRDEDKVAGAFSYISFGGGRHGCLGEPFAYLQIKAIWSHLLRNFEFELISPFPEIDWNAMVVGVKGKV
MVKYKRRKLSTE
>Catharanthus roseus_CYP72A1v1
MEMDMYTIRKAIAATIFALVVAWAWRVLDWAWFTPKRIEKRLRQQGFRGNPYRFLVGDVKESGKMHQEALSKPMEFNNDIVPRLMPHINHTIN
TYGGNSFTWMGRIPRIHVMEPELIKEVLTHSSKYQKNFDVHNPLVKFLLTGVGSFEGAKWSKHRRIISPAFTLEKLKSMLPAFAICYHDMLTKW
EKIAEKQGSHEIDIFPTFDVLTSDVISKVAFGSTYEEGGKIFRLLKELMDLTIDCMRDVYIPGWSYLPTKRNKRMKEINKEITDMLRFIINKRMKAL
KAGEPGEDDLLGVLLESNIQEIQKQGNKKDGGMSINDVIEECKLFYFAGQETTGVLLTWTTILLSKHPEWQERAREEVLQAFGKNKPEFERLNH
LKYVSMILYEVLRLYPPVIDLTKIVHEDTKLGPYTIPAGTQVMLPTVMLHREKSIWGEDATEFNPMRFADGVANATKNNVTYLPFSWGPRVCLG
QNFALLQAKLGLAMILQRFTFDVAPSYVHAPFTILTVQPQFGSHVIYKKLES
>Zea mays_BX3_CYP71C2
MALGAAYHHYLQLAGDHGTATHALLLGVLIFLVIRLVSARRTGTTSANKRKQQQRLPLPPWPPGKLPIIGHLHLIGAETHISIRDLDAKHGRNGLL
LLRIGAVPTLFVSSPSAADAVLRTQDHIFASRPPWMAAEIIRYGPSDVAFVPYGEYGRQGRKLLTTHMLSTKKVQSFRHGRQEEVRLVMDKIRA
AATAAPPAAVDLSDLLSGYTNDVVSRAVLGASHRNQGRNRLFSELTEINVSLLAGFNLEDYFPPNMAMADVLLRLVSVKARRLNQRWNDVFD
ELIQEHVQSRPSGESEESEADFIHVLLSIQQEYGLTTDNLKAILVDMFEAGIETSYLTLEYGMAELINNRHVMEKLQTEVRTTMGSPDGKKLDML
AEEDLGSMPYLKATIKETLRLHPPAPFLLPHYSTADSEIDGYFVPAGTRVLVHAWALGRDRTTWEKPEEFMPERFVQEPGAVDVHMKGKDLR
FIPFGSGRRICPGMNFGFATMEVMLANLMYHFDWEVPGSGAGVSMEESFGLTLRRKEKLLLVPRIAS
>Zea mays_BX2_CYP71C4
MAAQLHHALYELLHEAAAAQRALLLAIPFSLLLLPLLLRYLAASASASATKNDGAAPASDPDKLLSLLPSPPMKLPIIGHLHLMGDIPYVSLAALAT
RYGPDLMLLRLGAVPTVVVSSPRVAEAVLRTYDHVFSSRPRSLVSDIIMYGATDSCFAPYGDHFRKARKLVTVHLLNASKVRSQRPAREEEVR
GALDRVRRAAAAREPVDMSELLHSFVNNLVCRAVSGKFSMEEGRNRLFRELTDINAGLLGGFHIQDYFPRLGRIELVRKVACAKTRRVRKRW
DDLLDKLIDDHAARMATHQDEDDDKDFIYVLLSLQKEYGLTRDHIKAILIDMFEAGTDTSYMTLEFAMTELIRKPHLMKKLQEEVRRNVPAGQEM
VTEDNLPGMTDLKAVIKETLRLHPPVPLLLPHYSLDACEVAGYTIPANTRVVVNAWALGRHSGYWERENEFVPERFLSGDVAGGVDLKPNEFQ
FLAFGSGRRMCPGVHSASATIEAMLSNLMYRFDWQLPAGMKAEDVDMTEVFGITVSRKEKLLLVPQAA
>Zea mays_Bx4_CYP71C1
MALEAGYDYLHVAVVQCTPTQAAAVLGVLLLLAIRLAAAARSSSATSPKWKQHRLPPTPPGKLPIIGHLHLIGSHPHVSFRDLHAKYGHNGLML
VQVGAVPTIVVSTPQAAEAVLRTHDHVLASRPRNPVADIIRYNSTDVAFAPYGVYWRTARKVVNTHLLSAKMVFSKRREREEEVRLVVARIRDA
AEASPGTALDMTELLGGYASDFVCRAVLGESHRKQGRNKLFRELTETSAALLGGFNVEDYFPKLADVDLFLRIICAKAKSVSKRWDSLFNELLS
EYALSGGKQGDHNSEDFVHLLLSLQKDYGLTTDNIKGILVNMFEAAIETSFLVLEYSMSELMNNRHVLAKLQKEVRTATPDGRMVMEEDLSRM
PYLKATIKESMRIHPPAPFLLPHFSTHDCEINGYTIPAGTRVIVNAWALARDPTCWDKAEEFFPERFLEQGRDAEVDMYGKDIRFVPFGAGRRIC
AGATFAIATVEIMLANLIYHFDWEMPAEMERTGAKVDMSDQFGMTLRRTQKLYLVPRIPKCVSSS
>Zea mays_Bx5_CYP71C3v1
MALQAAYEYLQQAVGHGAWSSTQTLTLLLIAVPTVLLLLASLAKSTSSSGRGKPPLPPSPPGTLPIVGHLHHIGPQTHISLQELVAKYGHNGFLF
LRAGAVPTLIVSSPSAAEAVMRTHDHICASRPWSMASHILRYNTCDVAFSPLGEYWQQTRKLMNTHLLSNKKVYSFRHGREEEVCLVVDNLR
EAAAKSPSTAVDMSEVLAAYTNDVVSRSVLGSTHRKKGRNTLFREMTMTNVDLLVGFNLEYYIPRWPLTDLLFRLVCWKVTRHLKRWDALLE
EVIHEHVEMRKLSGDKEKESDDFIDIFLSRYEEYGFTMDNVKSLLMNVFEAAIETSYLVLESAMAELMNHRRVMKKLQAEVRAYGAEKKLDMIR
EDDLSSLPYLKASMKEALRLHPPGPLLLPHYSTADCQIDGYHIPANPRVLVNGWAIGRDPAVWEKPEEFMPERFMRDGWDKSNSYSGQDFR
YLPFGSGRRICPGANFGLATMEIMLANLMYHFDWEVPNEKEDGCWKVSMDEKFGLMLRRNELLYLVPRASS
>Strobilanthes_dyerianus_WEAC_scaffold_2082634
MELTLLTSFFLFAIFIVIILKHVSPTSVHSRLPPTPAPPLPIIGHLHLLKQPLHRTLHRFAQLHGPIFSLKLGVRRLVVLSSPDLIEECFTTNDIVLSNR
PRGLVNKYIGYDHSTIAGERYGHQWRKLRRMGAEEVLSPTRLNAFSQIRQDEIRRTLENLIRTPNCAKLEMRPLLFNLIFNMIMTMLTGKRYSG
DGDGEQFREMVSEVFEHAGASNPEEFLPFLMWIDYRGLKKKLAALGGKLDTFYQNLLEEHRRQKRNSVIGHLLSLQESDPEFYTDLIIKGLITN
MIIAGTDTSVVTLEWAISLLLNHPDDVLHKAKSELDSVVGRHRLIEEQDLPNLHYLHCIILETFRMFPAGPLVVPRESSADCKIGGYDVPSGTIVLF
NAWFIHRDPKVWDEPTRFKPERFEGQKEVEKQKLMPFGVGRRACPGSGLGHRMVGLALGALIQCFEWERVGSEEVDLSEGHGLTMPKLKPL
EAMCRPRQDMVNVLSKVG
>Strobilanthes_dyerianus_WEAC_scaffold_2013968
MEASLLYTLFSLVLLILIAFRLSGRRKLPPSPWPALPFLGHLYLLKFPLHRNLHNLSHKLGPIFSLRFGNRLAVVVSSSAIAEECFTKNDVVLANRP
RLILPQYIGYNYSSLVAAPYGDNWRSLRRFTTLEIFSHARIFSHARLNVFQSIREDEIRHMLSKLSRNSCQDFETVELRPLLFDLTFNNIMRMVSG
KRYFGRDEDNEEAKRFRELKDGIFSVSGVSNPADFIPLLRWFDFGGLEKKIKGFAKQMDDFLQGLIEEHRGRKNSNTMIDHLLSLRESEPEFYT
DNVIKALIVVMLLAGTDTSSVSVEWAMSLLLNHPDKLEKARSEIDSIVGDDRLISESDISKLPYLQHIISETMRLFPAAPMLVPHEASADCKIGGYD
VPRGAMVMVNAWGIHRDPAVWDDPTSFRPERFECGEIGAPKLLPFGMGRRSCPGNVLALRMVTTTVGSLIQGFEWQRSGEDKVDLSEGKG
VSMPKAVALEAKCKAREVVRKVFDA
>Anisacanthus_quadrifidas_PCGJ_scaffold_2067481
MELHTLLYTAFSFFFSILILFKFLSSSKNHGKNLPPTAPGALPILGHVHHLTLLHRALYSFSLKMGPIFSVRLGSKPVVIISSSGLVEECFNKNDIIM
ANRPLPSVDRRSLGFSNTSVIGAPYGDHWRNLRKLCDLEVFSPTRLASFLTIRRDEMDRMISSLYKVSSAGFAKVNVETKIVELTFNNIMRMIAG
KRYYGEDAEDDEEAKMFRDLTKEALELTSAANPGEIFPFLGRIGCFGLEKKLALHAKKTDVFMQGLLDDHRRGKRQNTMVDHLLGLQISQPEY
YTDEIITGLIVALIIAGTDASVVTTEWVMTQLLNHPDVMEKLRQEIDDHVGHDRAVEEHDLPKLRYLHHVVQETLRLFPSVPMLVPHMPSQDVQI
GGYDVPKGTMVLVNAWAIHRDPKVWSEPLKFKPERFEGLEMETQKLLPFGMGRRACPGAGLAQKYVGLAVASLVQFFDWKRTSAEKIDESE
GSGTTLVKIKTLEAMCKPRDVMADVLAEVVSRV
>Ruellia_brittoniana_AYIY_scaffold_2010156
MDNLFQWLLYLPLLFLLYLYTKHWFNKLRNLPPSPFISLPFLGHLHLAKKPLFRAFANVSARHGPVVLLHLGSRPVLVISSPEAAEECFTKNDVV
FANRPHLLAGKHLGYNYTSLAWSSYGDHWRNLRKISSIEILSSHRLELLQHIRVDEVRAMAAAMNRAAGEGRPVDMKTALFEVTMNVMMRMIA

GKRYYGENVEEVETARKFRELTIETFRLGTSNIGDFLPAVKWLRLGGVEKALMDLQRRRDALMQELVEECKKSLASNDGAAAEGKTMIEMLLA
LQKKEPEYYTDSIIRSLMLVLLLAGSDTSAGTMEWAMSLLLNNPHVLKKAQAEIDSHVGHDRLFEESDVADLPYLRCIVNETLRLYPAGPLLVPH
ESSESCVVGGYRIPAGTMLMVNVWAIHNDPKNWKDARAFKPERFEGLQGHRDGFKLMPFGSGRRGCPGEGLAVRMVGFALGTLIQCFDWE
RVGKELIDMTEGTGLTLPRATPLMAICKARPFTDKLLSQN
>Strobilanthes_dyerianus_WEAC_scaffold_2083330
MENLYLYLFPAILVALYSISFHLLNKLRNLPPSPFPALPLIGHIYLLRNPFHRSLSEVARRYGPALFLQLGSRPVLLISSPSLAEECLSGKNDVIFAN
RPCFLNGRYFGYNYTSLSWAPYGDHWRNLRRISALELLSSKRIQMFSSIRADETLTLVKKLFYVANEKSQKTVEVKSTLFEYMFNVLTRMITGK
RFYGKNMEKSKDAKLLEEINTETSSIAFETNVVDFFPIMRWFWFKDVEKKLMSIQKKRDRFMQQVIDEHRQIMQSNDYNSSPTKKKAMVEVLL
DLQRSEPEYYTDEYIKNLLLVLLQGGASTSTIALEWAFTLLLHNPETLKRAQSEIDAHVGQDRLITESDVAELPYLRQIILETMRLHPPVSILMPHR
SSAECRVGGYRIPAGTVLLINIWDIHHSPELWEEAKEFRPERFEGIEGKREIGIRFLTFGLGRRACPGENLAFPNIGMALGALIQCFDWEKVGKID
MSEGAGTTTPKVEPLKAKCSPRPFIAKFLA

Sequences for BX6 phylogenetic analysis

>Aphelandra squarrosa_BX6
MAAANLVSGTSVTTIPSKYILSELDRPKLSQVIESNDNIPVIDLALGDDALITKQMGEAAREYGFFQIINHDVPKEVVEGIIGVAREFFSLPVEEKM
KLCSDDTSKPTRLSTSFNLNNESAQSWRDYLRIQCYPLEKNVDEWPSVPSSFKDIVSNYYKEVRQVAFKLEEYLSKSLGLEKDSLKTLLGEDE
QGQHMTINYYPPCPQPDLTLGIPPHTDPNTLTILLPDTDVSGLQVLKDGKWMAVKPCPGAFIINIGDQLQAMSNGQYKSVWHRAIVNIDKPRISI
PSFICPSNTAKIRAPDDLISKGQTAQYTEFTFAEYYDKFWKQRKEHQHGLDLFKN
>Zea mays_BX6
MAPTTATKDDSGYGDERRRELQAFDDTKLGVKGLVDSGVKSIPSIFHHPPEALSDIISPAPLPSSPPSGAAIPVVDLSVTRREDLVEQVRHAAG
TVGFFWLVNHGVAEELMGGMLRGVRQFNEGPVEAKQALYSRDLARNLRFASNFDLFKAAAADWRDTLFCEVAPNPPPREELPEPLRNVMLE
YGAAVTKLARFVFELLSESLGMPSDHLYEMECMQNLNVVCQYYPPCPEPHRTVGVKRHTDPGFFTILLQDGMGGLQVRLGNNGQSGGCWV
DIAPRPGALMVNIGDLLQLVTNDRFRSVEHRVPANKSSDTARVSVASFFNTDVRRSERMYGPIPDPSKPPLYRSVRARDFIAKFNTIGLDGRAL
DHFRL
>Triticum aestivum_Bx6a
MRVVAAGTDRIFQIIMPATQLPAGEAWDRRRELQAFDDTKAGVKGLVDAGVTALPPIFRHSPESLEGITSSNHVTGAIPTVDLSAARREDTVAL
VRRAAGTVGFFQVVNHGVPAELMAGMLEGVRRFNEGPAEAKQAIYSRDQARKVRFASNFDLFSSAAANWRDTLFFHLAPDPAPSQELPEAV
RDVVTEYGKAVTKVALSVLELLSESLGLSSDHLRDMGCAENLNAVCQYYPPCPEPYLTWGTKRHTDPGFLTVLLQDGMGGLQVLVDHKTWV
DVPPVPGAFIINIGDLLQLVSNDQFRSVEHRVLANKSKDTARVSVASFFNTDMERSTRLYGPITDGRNPPIYRSVTARDFIATFNRIGLDGRSLD
HYRLDQHTPTPAVEGCE
>Triticum aestivum_Bx6b
MPATQLPAGEAWDRRRELQAFDDTKAGVKGLVDAGVTALPPIFRHSPESLEGITSSNHVAGAIPTVDLAAPRREDAVALVRHAAGTVGFFQVV
NHGVPAELMAGMLEGVRRFNEGPAEAKQAIYSRDQARKVRFASNFDLFSSAAANWRDTLFFHLAPDPAPSEELPEAVRDVVTEYGHAVTKV
GLSVLELLSESLGLSSDHLRDMDCAENLNAVCQYYPPCPEPYLTWGTKRHTDPGFLTVLLQDGMGGLQVLVDGKTWVDVPPVPGAFIINIGDL
LQLVSNDQFRSVEHRVLANKSKDTARVSVASFFNTNIERSTRLYGPITDGRNPPIYRSVTAREFIATFNRIGLDGRSLDQYRLDRDTPTPAVEGC
E
>Triticum aestivum_Bx6c
MPSTESAAGVAWDRRRELQAFDDTKAGVKGLVDAGVTALPPIFRHSPESLESITSSNHVAGAIPTVDLAAPRREDAVALVRHAARTVGFFQVV
NHGVPAELMAGMLEGVRRFNEGPAEAKQAIYSRDQARKVRFASNFDLFSSAAANWRDTLFFHLAPDPAPSEELPEAVRDVVTEYGHAVTKV
GLSVLELLSESLGLSSDHLRDMDCAENLNAVCQYYPPCPEPYLTWGTKRHTDPGFLTVLLQDGMGGLQVLVDGKTWVDVPPVPGAFIINIGDL
LQLVSNDQFRSVEHRVLANKSKDTARVSVASFFNTNIERSTRLYGPITDGRNPPIYRSVTAREFIATFNRIGLDGRSLDQYRLDRDTPTPAVEGC
E
>Triticum aestivum_Bx6d
MPATENAAGEAWDRRRELQAFDDTKAGVKGLVDAGVTALPPIFRHSPESLEGITSSNHDAAAIPTVDLSAARREDTVALVRRAAATVGFFQVV
NHGVPAELMAGMLEAVRRFNEGPAEAKQAIYSRDQARKVRFASNFDLFSSAAANWRDTLFFHLAPDPAPSQDLPEAVRDVVTEYGKAVTKVA
LSVLELLSESLGLSSDHLRDMGCAENLNAVCQYYPPCPEPYLTWGTKRHTDPGFLTVLLQDGMGGLQVLVDRKTWVDVPPVPGAFIINIGDLL
QLVSNDQFRSVEHRVLANKSKDTARVSVASFFNTDMERSTRLYGPITDGRNPPIYRSVTARDFIATFNRIGLDGRSLDHYRLDQDTPAHAVEG
SE
>Secale cereale_Bx6
MPATKNSAGVAWDRRRELQAFDDTKAGVKGLVDAGVTTLPPIFRHSPESLEGITSSNHDPAAIPTVDLSAAQREDTVAMVRRAAGTVGFFQV
VNHGVPAELMAGMLEAVGRFNEGPVEAKQAIYSRDQARKVRFASNFDLFSSAAANWRDTLFFHLAPYPAPSEELPEAVRDVVTEYGHAVTKV
ALSVLELLSESLGLSSDHLRDMGCAENLNAVCQYYPPCPEPYLTWGTRRHTDPGFLTVLLQDGMGGLQVLVDGKTWVDVPPVPGAFIINIGDL
LQACTYYPPSLVSNDQFRSVEHRVLANKSKDTARVSVASFFNTDMERSARLYGPITDGRNPPIYRSVTARDFIATFNRIGLDGRSLDHYRLDQE
TPTPAAVEGCE
>Solanum lycopersicum_NM_001246911.3
METKVISSGINHSTLPQSYIRPESDRPRLSEVVDCENVPIIDLSCGDQAQIIRQIGEACQTYGFFQVINHGVPKEVVEKMLGVAGEFFNLPVEEKL
KLYSDDPSKTMRLSTSFNVKKETVHNWRDYLRLHCYPLEKYAPEWPSNPSSFREIVSRYCREIRQLGFRLEEAIAESLGLDKECIKDVLGEQG
QHMAINYYPPCPQPELTYGLPAHTDPNSLTILLQDLQVAGLQVLKDGKWLAVKPQPDAFVINLGDQLQAVSNGKYRSVWHRAIVNSDQARMS
VASFLCPCDSAKISAPKLLTEDGSPVIYQDFTYAEYYNKFWSRNLDQQHCLELFKN
>Olea europaea var. sylvestris_XM_023009175.1
MEAKVISSGIRHSSLPRSYIRPQSERPRLSEVADCENVPVIDLGCGDRSLIIKQIGDACCEYGFFQVINHGVSKETIQKMLAVGHEFFSLPVEEK
MKLYSDDPSKTVRLSTSFNVKKETVHNWRDYLRLHCYPLEKYVPEWPTTPSSFKDIASNYCMEVRHLGFRLQELIAESLGLEKESLKNVLGEQ
GQHMAVNYYPPCPEPELTYGLPAHTDPNALTILLQDLLVAGLQVLMDGKWLAIKPHPDAFVINIGDQLQALSNGKYRSVWHRAIVNSDKARLSV
ASFLCPCDSAMIVAPKDLINDGSPSIYRDFTYAEYYKKFWSRNLDQEHCLELFKN
>Achantus_ilicifolius_TRINITY_DN207_c0_g1_i4
MATKLISSAHFSALPSSYIRPESERPRKSEIVESADIRVIDLSSEDRELIIKQIGDACKEYGFFQVVNHGIPKQAVDDVLRVTREFYHQPVEEKMK
MYSEDMSKEQRLSTSYNINQESIHIWRDYMRLHCYPLEKYMPDWPSSPPAFKEIVSKYAKEVRELGFRLQELFSLSLGLEKDALRKIFEEDQAQ

HMAFNYYPPCPEPDLTIGLASHTDSDILTVLLPDSDVSGLQVLKDGKWLAVHPHPDALIINIADQVQVVSNDKYKSNWHRAVVNSDRARISIASF
ICPAYTAKIKAPAEISDEEGRHYRDFTYKEYYDVVWSQDLDQEHPLEYFRIRK
>Achantus_leucostachyus_TRINITY_DN17067_c0_g1_i1
MAMMISASALQTLPDGYIRRKPDRPRLSDVVTNCNTIPVIDLTTTHTQLVTQIAHACRHYGFFQVINHGVPTKVVDDLLCVAHEFFSLPPEEKMK
LYSDDPFKTVRLSTSFNINNETLHNWRDYLRLHCHPIHKFAPPWPTLPSSFKDVVSNYCKQVRELGFRLEEAISESLGLPKDRITNILGDQGQH
MAINYYPPCPEPDLTYGLPPHTDPNTLTILLQDHQVSGLQVLKDGDWLEVKPLRDAFVINIGDQIQALSNGRYKSVKHRAIVNADRARVSVPTFL
CPCDSARIRAPEELRGEGEGDQSVYREYTYAEYYNKFWSGNLDQEHGLEVFKNL
>Achantus_leucostachyus_TRINITY_DN24783_c0_g1_i1
MATKLISSAHFSALPSSYIRPESERPRKSEIVESADIPVIDLSSEDRELIIKQIGDACKEYGFFQVVNHGIPKQAVDDVLRVTREFYHQPVEEKMK
MYSEDMSKEQRLSTSYNINEEAIHIWRDYMRLHCYPLEKYMPDWPSSPPTFKNIVSKYAMEVRELGFRLQEMFSLSLGLEKDALRKIFEEDQA
QHMAFNYYPPCPEPDLTVGLASHTDSDILTVLLPDLDVSGLQVLKDGKWLAVHPHPDALIINIADQVQVVSNDKYKSNWHRAVVNSDRARISIA
SFICPAYSAKIKAPAEITDEEVRHYRDFTYKEYYDVVWSQDLDQEHPLEYFKVRK
>Achantus_ungaricus_TRINITY_DN6050_c0_g1_i5
MATKLISSAHFSTLPASYIRPESERPRKSEIVESADIPVIDLSGDREVIIKQIGDACQEFGFFQVVNHGIPKQAVEDVLWVTREFYHQPVEEKMKM
YSEDMSKEQRLSTSYNINEESLHIWRDYMRLHCYPLEKYMPDWPSSPPDFKDIVSNYAKEVRELGFRLQELFSLSLGLEKDALRKIFEEDQAQ
HMAFNYYPPCPEPDLTVGLATHTDSDILTVLLPDLDVSGLQVLKDGKWLAVHPHPGALIINIADQVQVVSNDKYKSNWHRAVVNSDQARISIAS
FICPAYGAKIKPPPEITDEEGRHYRDFTYKEYYDVVWSQDLDQEHPLEYFRIKK
>Achantus_ungaricus_TRINITY_DN7488_c0_g1_i1
MAARLISAGRPYSTLPASYVRPESDRPNLCDVSDYQNAPVVDLAAPDRNDVVRQIAQACTHSGFFQVINHGVREEVMENMLKLADEFFNLPP
EEKMKLYSNDPSRTIRLSTSSNPDKEKYHNWRDYLRLHCHPLEKYIEDWPQNPPSFKKIVSMYCTEIRQLGLRLQEAISESLGLDKDCINNLGE
QGQHMAINYYPPCPEPDLTYGLPAHTDPNTLTILLPDSQVPGLQLLKDGKWLSLKPVPNAFVVNIGDQLQALSNGRYKSSWHRAVVNENQARI
SIASFLCPSNVTMIKAPQQLIDADGSKAVYKDYTYAEYYDKFWTRNLDQGHCLDLFKIA
>Aphelandra_aurantiaca_TRINITY_DN736_c0_g1_i5
MATKLISGAHFSALPSSYIRPESERPRKSEIVESANIPVIDLSSEDRDLILKQIGDACQEYGFFQVINHGIPKEAVEKMLWVTREFYHQPVEEKMK
MYSEDMTIEQRLSTSYNINAEAVHIWRDYMRLHCYPLEKYMPDWPSSPPSFKEIVSNYSKEVRQLGFRLQELFSESLGLEKETLRKIFGEEQAQ
HMAFNYYPPCPEPDLTVGLASHTDSDILTVLLPDLEVTGLQVLKGNKWLAVNPHPDALVINIGDQVQVVSNDKYNSNWHRAVVNSDRPRISIAS
FVCPSYDAKIKPPPEITDEEGRHYKDYTYKEYYDVVWSQDLDQPHPLVCFRK
>Avicennia_officinalis_TRINITY_DN13184_c0_g1_i1
MEARLISAGLPYLTLPRSYVRPEPQRPKLSQVADYHNAPIVDLACPDWRFIVQQIEQACRQYGFFQVINHGVQKEIVENMLRVANEFFCLPVEE
KMKLYSNDPSKTTRLSTSSNPQKEKIHNWRDYLRLHCYPLEKYTEEWPVNPPSFKEIVSTYCTEIRQLGLRLEEAISESLGLDRDCINKSLGEQ
GQHMAINYYPPCPEPELTYGLPAHTDPNTLTILLQDTQVAGLQLFKDGKWLSVKPHPNAFVVNIGDQLQALSNAKYKSTWHRAVVNAEKARISI
ASFFCPCNSAVIKAPKELVADESPAIYKDYTYAEYYDKFWSRNLDQGHCLELFETA
>Avicennia_officinalis_TRINITY_DN376_c0_g1_i4
MEARLLSAGPRHLTLPPSYVRPEPQRPNLSDIADFDNAPVIDFGCADRAFVVRQIALACSLYGFFQVTNHGVPGEVMEKMLRVSNEFFCLPVE
EKMIFYSNDPSKTTRLSTSSNPHKEKIHSWRDYLRLHCYPVDKYVQEWPSNPPSFKEIVGNYCKEVRQFGMRLQEAISESLGLDKDYINRSTG
EQGQHMAINFYPLCPEPELTFGLPAHTDPHTLTILLQDTQVTGLQLLKDGKWLPIKPLPDALVVNVGDQLQALSNAKYKSAWHRVVVNGQKAR
LSVASFLCPSSAAVIKAPEELVGDESQRVYRDYTYAEYYDRFWSRNLDQGQTLDVFNAV
>Aphelandra squarrosa_TRINITY_DN10065_c0_g1_i1
MATKLISGAHFSTLPSSYIRPETERPRKSEIVESANIPVIDLSSEDRELILKQIGDACQEYGFFQVINHGIPKEAVDKMLWVTREFYHLPVEEKIKM
YSEDMTIEQRLSTSYNINEEAVHIWRDYMRLHCYPLEKYMPGWPSSPPSFKEIVSNYSEEVRQLGFRLQELFSESLGLERDELRKVFGEEQAQ
HMAFNYYPPCPEPDLTVGLASHTDSDILTVLLPDKEVTGLQVLKDNKWLAVNPHPDALVINIGDQVQVVSNDKYNSNWHRAVVNSDRPRISIAS
FVCPSYDAKIKPPPEITDEEGRHYKDYTYKEYYDVVWSQDLDQPHPLVCFRK
>Aphelandra squarrosa_TRINITY_DN8866_c0_g1_i1
MSPGMVMGGESSGANSELVESNLYQKGVKHMYDNGVEQIPGKYIFPADERPTIVTEKTKNMDDDGNDLNLPVIDFAELQGPNRKQALKSLAC
ACESYGFFQLVNHGVAEEMRKSMVDVSKRFFEMPIVEREKLMTADINSPVRYGTSFNQTNDGIFSWRDFLKLICHPLPDVLPHWPSSPHDFRE
LAVNYAKEMRSLFLKLVEVILESLGLKADNMVQDEDATEESFKDGSQIMVVNCYPPCPQPDLTLGMPPHSDYGFLTLLLQDEVKGLQIQHAGK
WVTVQPIPGSFIVNVGDHLEIFSNGRFKSVLHRVIVNPSHDRISVASLHSVPFTTAVRPATELIDEGNPRRYKDTSFANFLDYITTCDSKHKNFLD
SRKINLSN
>Aphelandra squarrosa_TRINITY_DN48359_c0_g1_i1
MFAVQSLTASCQLSPRMSPAAALATCSRKSYCIKNSLESSHVYQKGVKQMHENGIKHVPRKYILPDAERPNVIVSGPETMVKLPIIDFAELQGP
NRTQVVESLACACKNYGFFQLVNHGIENDSISDMVGVSRRFFEMPYSEREKYMLADVSSPVRYGTSFNQTNDGVFSWRDFLKLVCHPQPLP
EDTLSRWPSSPVGFRQSAVTYANETRTLFLNLAEAILESLGLHNCEKTESVTEDDEEAMLLKELQDGSQVMVLNCYPPCPQPDLTLGMPPHS
DYGFLTLLLQDEVDGLQIQHQDQWFTVRSIPGSFVVNVGDHLEIISNGKYKSVLHRVVVNPTEHRISVASLHSVPFTCTVRPAARLVTQTNPRR
FKDTNFANFLEYITSCDSRDKNFLESRKLV
>Rosa chinensis_XM_024338235.2
MVSDNSSNQDRLQQLKAFDESKAGVKGIVDAGITKIPPIFVRPPADYACEDQVSGKTTKTHFSIPVVDLADTAGRRPEVIDGVRRAAESVGFFQ
VVNHGIPKRVLEGMLEAARGFHELPREAKAEYYSRELMRKVKFGSNFDLYQSRFVNWRDTLFCVMGPEPLDPQELPLVCRDITMEYSDQVHK
LGVTLFELLSEALGLKPDHLLELDCAKGHLILSHYYPPCPEPELTLGTTKHSDPDFLTILLQDHIGGLQVLCQNQWIDVPPVPGALVVNIGDLLQLI
SNDRFKSVEHQVLANLEGPRVSIACFFTLHVYPSTRLYGPINELLSENRPPVYKETSLQDFIAYSYGKGLDGNSALTHFKCQR
>Phaseolus vulgaris_XM_007149235.1
MGFGVSVSEKVNTERVRELKEFDDTKAGVKGLVDQGITKIPRIFHHPPDEHVKVSTSGGEADNIPVIDLAEDDKDPSLRQGVIDRIREASEKWG
FFQVVNHGIPVTVLEDLKDGVCRFYEQDTEVKKDLYTRDQMKPFLYNSNFDIYSSPALNWRDTFFCYLAPNPPKPQDLPVVCRDILLEYGTCV
MKLGIALFELLSESLGLHPNHLKDMDCAKGLISLCHYYPACPEPELTLGTTKHSDNCFLTVLLQDHIGGLQVLYQNTWIDIAPEPGALVVNIGDLL
QLITNDRFRSVEHRVVANVTGPRISVACFFSEGLKSSGKLYGPIKELLSEDNPPKYRETTVEEYVRYFNEKGLDGTSALHHFRI
>Sesamum indicum_XM_020692686.1
MVVSRTVEETEAPAEPKYDRKSELQAFDDTKAGVKGLVDAGVTKLPRIFIHPPENLRDIKNPTRTHFDFPLIDLDGVDEDAGIREKIVDRVREAS
ETWGFFQIVNHGVPVSMLEEMLDGVRRFNEQETEIKKQFYSRDSTKKVTFNCNFDLFSSPAANWRDTIYCVISPNPAQPEELPTVCRDIIIEYSK
QVIRLGRCLFKLMSESLGLSPDHLIEMECAESLALIGHYYPPCPEPDLTFGISKHSDNNFITVLLQDNLGGLQVRHQNQWVDVPPLPGALVVNIG
DLFQLVSNDKLKSVEHRVLASNAGPRISVASFFGRDSGPSSKVYAPMKELLSEDDPPKYRPTTANEYTEFFRAKGLGGTSALLHFRL
>Nicotiana tabacum_XM_016593157.1
MDSSSNNEVFSAIESKYDRKRELKAFDDTKAGVKGLVDAGVSKVPKIFISPSDTTKKSNSSTEQFIFPVINLQGIIDGDQIKRKEVVEKVRDASET
WGFFQVVNHGIPSDVLEEMIRGVRSFHEQDTEIKKQWYTRQLTKRVVYNSNFDLYSAPATNWRDTFFCIMAPNPPSPEQLPPICRDIIIKYSEE

VKKLGSYLFELLAEALGLNRNHLSEMDCDKGLSVVCHYYPACPEPQLTLGASKHADDGFITLLLQDNIGGLQVLHQNHWVDVPPTLGALVVNIA
DLLQLISNDKFKSVEHRVLANHIGPRISIACFFSTFSLESSRLYGPIKELLLEDNPPKYRETTIQEYAAYFTEKGLDGTSALHHFRL
>Camellia sinensis_XM_028271462.1
MIATTKTSIQAAIETNYDRAQELKAFDDTKTGVKGLVDAGIARVPKIFIQPPDNLNKTTNPSNTQFNFPIIDLTVIDKDPIRRRQVIDEIREAAENWG
FFQVVNHGVPESVLEEMKKGVHRFYERDSEVRKEWYTRDVKKAVVYNSNFDLFSAPAANWRDTFNCSMAPKPPNPEELPPPCRDILMEYSK
QVIKLGCCLLELLSEALGLSPNHLKDIGCAEGLAVLCHYYPACPQPELTVGTTKHSDDDFLTVLLQDHIGGLQVLHQNHWVDCPPTPGALVVNI
GDLLQLVSNDKFKSIEHRVLANRVGPRVSVACFFSTDLQPSSKVYGPIKELLSEENPPKYRETTVRDYVVYFQAKGLDGNSVLSHFKL
>Arabidopsis thaliana_DAO1
MGELNGVIIPTIDLEEVSDKILNQKIREASERWGCFRVINHGVSLSLMAEMKKTVIDLFQRPYEVKVRNTDVLLGSGYRAPNEINPYYEALGLYD
MASPHAVNTFCDQLEASADQREIMVKYAKAINGLATDLARKLAESYGLVETDFFKEWPSQFRINKYHFKPETVGKLGVQLHTDSGFLTILQDDE
NVGGLEAMDNSSGTFFPIDPLPNTLAINLGDMATIWSNGRLCNVKHRVQCKEATMRYSIASFLLGPMDTDLEPPSEFVDAEHPRLYKPISHEGV
RNIRMTKKLHDGEALKLITHAGLDK
>Zea mays_FNSI
MATGCAASSIVSPLAMDMAASGALPVVDLAPFFTDGDEGGASRARATEAVRQACRTHGFFRVVNHGVPAHLMARALELSSAFFALPDDDKAR
ARAAEGSEAPLPAGYARQPAHSADKNEYLLVFGPKLGFNVYPAEPAGFREAVEECYGKLTELGLLVQEVLNECMDLPPGFLTDYNSDRSFDF
LAALRYFPATEEEDNGISAHEDGNCITFVIQDGVGGLEVLNDDGDWVPAEPVEGSIIVNLGDVIQVLSNNKLKSATHRVVRKPVHRHSFVFFFNI
HGAKWIEPLPEFTTKIDEPPRYKGFVYNEYMQLRMRNKTHPPARPEDVVHITHYAI
>Papaver somniferum_thebaine 6-O-demethylase
MEKAKLMKLGNGMEIPSVQELAKLTLAEIPSRYVCANENLLLPMGASVINDHETIPVIDIENLLSPEPIIGKLELDRLHFACKEWGFFQVVNHGVD
ASLVDSVKSEIQGFFNLSMDEKTKYEQEDGDVEGFGQGFIESEDQTLDWADIFMMFTLPLHLRKPHLFSKLPVPLRETIESYSSEMKKLSMVLF
NKMEKALQVQAAEIKGMSEVFIDGTQAMRMNYYPPCPQPNLAIGLTSHSDFGGLTILLQINEVEGLQIKREGTWISVKPLPNAFVVNVGDILEIM
TNGIYHSVDHRAVVNSTNERLSIATFHDPSLESVIGPISSLITPETPALFKSGSTYGDLVEECKTRKLDGKSFLDSMRI

Sequences for BX7 phylogenetic analysis

>Aphelandra squarrosa_BX7
MDHHESDEQEAAFSFAWHLSHGAVLPMAVKCAIELDLFQLIKKAGPDAAVSASQLAAQIPNTRNPDVVAMIEKLLRFLAANSVLSCQLTPMPDL
TLQRTYSLTPVCNYLIADEDGASLAPACLINHDKVSMEAWYHLKDAIVEGGIPYNRAYGMSSYEHLGKNPRTNQLFNQSMYGASTITMKRILEN
YPGFEGVKSLVDVGGGTGASLKLILAKHPSIKGINFDLPHVVQDAPSHPGIEHVGGDMFMSVPQGDAIFLKWICHNWSDSQCEKLLKNCYEAL
PENGKVIIVETILPNSDPKKSAPEVEKLAYQAAAGYDLIMFAFNPGRERSEEELEDLGKHAGFKHFHKVCHAFGYWIMEFLK
>Zea mays_Bx7
MGHQAQHGTDDTEELLAAHRQLWCHALGYVKSMALKCALDLRIPDTIDRCGGSATLGELLAASEIPASNHDYLRRVMRTLTAMRIFAVSHDDP
AKADDAAAISYQLTPASRLLVSSSSSSVDAAAAGAGASKENTTTPSILPNIAHLVRPNTISLLFSMGEWMKDESAASVSLYETVHRQGMWACVE
DDAANRASFYESMDADTRLVMQAVVRRCPHVFDGIKSLVDVGGGRGTAAAAVVAAFPHIQRCTVMDLPHVVAEAPAGTAGLSFHGGDMFEH
IPSADALMLKWILHDWDEDKCIKIMERCKEAIGGKEAGGKVIIIDTVLGSRADDDDDDKTCRETYVLDLHILSFVNGAEREEHEWRRIFLAAGFR
DYKITHTRGIPSIIEVFP
>Aphelandra squarrosa_TRINITY_DN5839_c0_g1_i2
MDHHESDEQEAAFSFAWHLSHGAVLPMAVKCAIELDLFQLIKKAGPDAAVSASQLAAQIPNTRNPDVVAMIEKLLRFLAANSVLSCQLTPMPDL
TLQRTYSLTPVCNYLIADEDGASLAPACLINHDKVSMEAWYHLKDAIVEGGIPYNRAYGMSSYEHLGKNPRTNQLFNQSMYGASTITMKRILEN
YPGFEGVKSLVDVGGGTGASLKLILAKHPSIKGINFDLPHVVQDAPSHPGTHIYIFLSSLPQETNSYFKPLIEHQSCAGIEHVGGDMFMSVPQGD
AIFLKWICHNWSDSQCEKLLKNCYEALPENGKVIIVETILPNSDPKKSAPEVEKLAYQAAAGYDLIMFAFNPGRERSEEELEDLGKHAGFKHFHK
VCHAFGYWIMEFLK
>Aphelandra squarrosa_TRINITY_DN5839_c0_g1_i4
MAVRCAVELDLLQLIKKAGPDAAVSASQLAAQIPSTNKNPDAVAMIDRLLRFLAANSVLNCHLTPMPGRALQRAYSLTPVCNYLTTDEDGASLA
PYSLINQDKVSMEAWYHLKDAIIEGGIPYNIAYGMCSYEHMAKNPRRNHLFNQAMYGASTITMKGILANYGGFDGVKTLVDVGGGTGASLKMIL
AKYPSIKGVNFDLPHVVQDVASHQGMEHVGGDMFVSVPQGDAIFLKFICHNWSDSQCEKLLKNCYEALPENGKVIIVDTIFSDCDPKRSVPEV
ERLSNQAAAVFDFIMFAYSSGRERSEEEFESLGKHAGFTQFHKVCHAFGYWIMEFLK
>Aphelandra squarrosa_TRINITY_DN5839_c0_g1_i3
MAVRCAVELDLLQLIKKAGPDAAVSASQLAAQIPSTNKNPDAVAMIDRLLRFLAANSVLNCHLTPMPGRALQRAYSLTPVCNYLTTDEDGASLA
PYSLINQDKVSMEAWYHLKDAIIEGGIPYNIAYGMCSYEHMAKNPRRNHLFNQAMYGASTITMKGILANYGGFDGVKTLVDVGGGTGASLKMIL
AKYPSIKGVNFDLPHVVQDVASHQGMEHVGGDMFVSVPQGDTIFLKWICHLWSDSQCEKLLKKCYEALPENGKLIIVECILPKGDPESCAPEVE
RLAYQAAAVTDFIMLGYNPGWERSAEEFEALGKRAGFTQFHKACYAFGVWVMELRK
>Achantus_ilicifolius_TRINITY_DN10004_c0_g1_i13
MDSSITSKCNELVGSSNKKMDDDGDDNEEKSYMLALDLATASTLPMALKCAIELNLLELIKAANPYPASASQLVAHMPTENPVAATDQIDRILRF
LAANSILNLDTETQPDGTLTRLYSLAPVCKYFTKDEDGVSLASTSLLIQDRVFMETWYHMKDAVLEGGIPFNRAYGMNAFEYPAKDPRFNMIFN
HAMHESSTIAMKKVLEIYKGFEGLRSLVDVGGGIGASLKMILAKYPSIKAINFDLPHVIQHALPQPGVEHVGGDMFVSVPKADAIFMKRICHDWS
DAHCQNLLKNCYEALPEHGKVIIVETILSNEKMVKTKSETLAFQRAIQGDMRMLAYNPGGRERSEQEFDALGKHAGFKQFHKVCYVPGYWIME
FIKGN
>Achantus_ilicifolius_TRINITY_DN10004_c0_g1_i4
MDSSITSKCNELVGSSNKKMDDDGDDNEEKSYMLALDLATASTLPMALKCAIELNLLELIKAANPYPASASQLVAHMPTENPVAATDQIDRILRF
LAANSILNLDTETQPDGTLTRLYSLAPVCKYFTKDEDGVSLASTSLLIQDRVFMETWYHMKDAVLEGGIPFNRAYGMNAFEYPAKDPRFNMIFN
HAMHESSTIAMKKVLEIYKGFEGLRSLVDVGGGIGASLKMILAKYPSIKAINFDLPHVIQHALPQPGVEHVGGDMFVSVPKADAIFMKRICHDWS
DAHCQNLLKNCYEALPEHGKVIIVETILSNEKMVKTKSETLAFQRAIQGDVLMPLFQTLS
>Achantus_leucostachyus_TRINITY_DN1158_c0_g1_i2
MDEHKKAVKMEEDQEETFMLAYDLATASTLPMVLRSAIELNLFELIKAAAPSPASATYLAARIPTNNLPVAADQIDRILRFLAAYSILNCHVETQA
DGGLKRLYSLAPVCNYFTKDEDGVSLAPVSLMVQDRVFMETWYHMKDAVMEGGIPFNRAHGMNAFEYPNKDPIFNRVFNSCMHDTSTMMM
KKFIEKYKGFEGLQTLVDVGGGTGASLKMILAKYPSIKAINFDLPHVIQHAPPHPGVEHVGGDMFVSVPKADAIFMKRICHDWSDAHCQNLLKN
WYQAIPQHGKVIIVEHILCNEKTIRTNAEVMALQRVTQSDVLMLAYNPGGRERSEQEFEALGKYVGFKHFHKVCYALGNWIMELSK
>Achantus_hungaricus_TRINITY_DN8052_c0_g1_i4

MEKKEMEEESFMLAMSLATASTLPMALRSAIELNLLELIKAATPSPVSASHLAARIPTTNPAAAADQIDRILRFLAANSILNLHTDGSGTSLTRRYS
LTPLCNYFVKDEDGVSLASTSLMIQDRVFMETWYHMKDAVFEGGVPFDRAHGMNAFEYPAKDPRFNMVFNRCMHDSSTIIMKKLVEIYRGFE
GVQSLVDMGGGIGASLKMILAKYPSIKAINFDLPHVIQHAPPHPGVEHVGGDMFVSVPKADAIFMKRICHDWSDAHCENLLKNCYEALPMHGK
VIIVENILANQEMVKTNSEMMAFHRTIRADVLMLAYNPGGRERSEQEFDALSKHAGFKEFRKVCYALGYWIMEFIK
>Aphelandra_aurantiaca_TRINITY_DN5987_c0_g1_i5
MGSIMDHDEEATAFSFAWHLVTSSVLPLALKVAIELDLFQLIKKAGPDAAVSASQLAAQIPNTRNPNVAAMIEKLLRFLAANSVLSCHHTPMPDL
TLQRTYSLMPVCNYLIADEDGASLAPACLINHDKVSMEAWYHLKDAIVEGGIPYNRAYGMSSYEHLGKNPRTNQLFNQSMYGASTITMKRILEN
YPGFEGVKSLVDVGGGTGASLKMILAKHPSIKGINFDLPHVVQDAPSHPGIEHVGGDMFMSVPQGDAIFLKWICHNWSDSQCEKLLKNCYKAL
PENGKVIIVETILPNSDPKRSAPEVEKLAYQAAAGYDLIMFAFNPGRERSEEELEGLGKHAGFKHFHKVCHAFGYWIMEFLK
>Aphelandra_aurantiaca_TRINITY_DN5987_c0_g1_i2
MGSIMDHDEEEAVFSFAWHLVTGSVLPMALKVAIELDLFQLIKKAGPDAAVSASQLAAQIPKANRNPDAATMIDRLLRVLAANSLLNCHLTPMP
DHTHQRAYSLTPVCKYLTTNEDGVSLTPASLINQDKAMMEAWYHLKDAIVEGGVPFDRACGMPAFEYQATNPRTSQLFNQYMYGTGTSTIIMKRI
LEKYGGFDGVKCLVDVGGGTGASLKMILAKHPSIKGINFDLPHVVQQAASHPGIENVGGDMFVSVPQGDAIFLKWICHDWDDSQCEKILKKCY
EALPENGKVIMVETIIPNGDIASESEKMGFRRAAVMDMLMLVCHSGRERSEEELEDLGKHAGFTQFHKVCHIFGNWIMEFHK
>Triticum aestivum_Bx7-like
MALAREQHILDQGLLDAQLELWHHTFGYVKSMALKSALELSIADAIHREGGAATLSQIAATATLHPSKISCLRRLMRVLTVSGVFGIEHSRDGGV
EGEAVYALTPASRLLVGSASVNMVHITKMLLHTNLVSPFSDLGTWFQLELPEPDLFKLKHGKTFWELANQDPSYNALVNDGMVSDSNFLMDIAI
RECGAVFQGIGSLVDVAGGHGGAAQAISNAFPDLQCSVMDLAHVVAKAPSGTGVEYIAGDMFESVPPADAVFLKWVMHDWGDEDCIKILKNC
KKAIAPKDVGGKVIIVDMVVGAGSRDQKHKETQVMFDLFIMFVNGIERDEREWKKIIFEAGFSDYKITPVLGVRSIIEVYP
>Secale cereale_Bx7
MATANQEDELTMSTDEFLEAQAELYHHCIAFVKSLTLKAATDLGIADAIHRRGGATTLSDLAAETGIHPTKLSNLRRIMRVLTTSGIFSVQHSDVV
SDDDAVYKLTRVSRLLVKSSSYPLSPVVPVLVDPFAWSALLKMPEWLATDERASLFDLAHGCPLWDTTVEDGGLFKDGMAADSRVAMQVLLA
RCGGVFEAVRESLVDVGGSHGTTAAAVANAFPHIKCTVLDLPDVVAGAPANDAALTFVAGDMFEYIPQADAILLKWILHDWKDGDCIKILRRCK
EAIPSRDAGGKVIIIETVVGSGSPGAQKIFPKEAQVLMDVFMMYIDGIEREEHEWSKIFFEAGFSDYKITPTNGVLSVIEVYP
>Sesamum indicum_XM_011086278.2
MSLSIKTQIPTASMADQSEEEEAFLYAMELASASALPMVLKSAIELDLLELIKKAGPGASVSPSQLAAQLPTKNPDAATMIDRMLRLLAAYSVVR
CSLKPLPDGGVERLYSLAPVCKFLTRNEDGVSVAPVALMLQDKVFMESWYHIKDTVLEGGIPFNRAYGMSAFEYPATDPRFNKVFNRAMYEQ
STIFMKQILEKYKGFEGVKSLVDVGGGIGASLKMILSKYPTIKGINFDLPHVIQDAPSFPGVEHVGGDMFVSVPKADAIFMKWICHDWSDAHCQK
LLKNCYDALPDNGKVIIADSIFPEDPNSGPAFKRVAHGDVIMLALNPGGKERSEKEFQSLAQYAGFREVIKVCSAFNIWIMEFHK
>Catharanthus roseus_AY028439.1
MGSANPDNKNSMTKEEEEACLSAMRLASASVLPMVLKSAIELDLLELIKKSGPGAYVSPSELAAQLPTQNPDAPVMLDRILRLLASYSVLNCTL
KDLPDGGIERLYSLAPVCKFLTKNEDGVSMAALLLMNQDKVLMESWYHLKDAVLEGGIPFNKAYGMTAFEYHGKDPRFNKVFNQGMSNHSTII
MKKILEIYQGFQGLKTVVDVGGGTGATLNMIVSKYPSIKGINFDLPHVIEDAPSYPGVDHVGGDMFVSVPKGDAIFMKWICHDWSDAHCLKFLK
NCHEALPENGKVILAECLLPEAPDSTLSTQNTVHVDVIMLAHNPGGKERTEKEFEALAKGAGFRGFIKVCCAYNSWIMELLK
>Rauvolfia serpentina_KX687825.1
MGSAAPEQSNSMAGDEEACLFAMQLASASVLPMVLKSAIELDLLELIKKAGQDAYVSPSELAAQLPTENPDAPVMLDRILRLLASYSVLNCTLR
NLPDGGVERLYGLAPVCKFLTKNEDGVSMAALLLMNQDKILMESWYHLKDAVLEGGIPFNKAYGMTAFEYHGTDPRFNKVFNQGMSNHSTII
MKKILEIYNGFEGLKTVVDVGGGTGATLSMIVSKYPAIKGINFDLPHVTGDAPTYPGIEHVGGDMFFSVPKGDAIFMKWICHDWSDEHCLKFLK
NCYQALPENGKVILAECLLPEAPDATLSTQNTVHVDVIMLAHNPGGKERTEKEFEALAKEAGFKGFRKICCAYNSWIMELLK
>Ocimum basilicum_HM990154.1
MSSTANNPQINSDEEENFLFAMQLASASVLPMVLKSAIELDLLELIKKAGAGAFVSPAELAAQLLTTNAEAHVMLDRILRLLTSYAILECRLKTLPD
GGVQRLYGLAPVCKFLTKNEDGVSMAPLALMNQDKVLMESWYHLKDAVLDGGIPFNKAYGMTAFEYHGTDPRFNKVFNQGMSNHSTITMKKI
LETYTGFDGLKTVVDVGGGTGATLNMIISKYPSIKGINFDLPHVVEDAPSYPGVEHVGGDMFVSVPKGDAIFMKWICHDWSDAHCVKFLKKCY
EALPENGKVILAECVLPEAPDTGLATKNVVHIDVIMLAHNPGGKERTEKEFQVLAKASGFKQFNKVCCAYNSWIMELLK
>Coffea arabica_XM_027254142.1
MAEEEACLFAMRLASASVLPMVLKSAIELDLLELIAKAGPGAYVSPSELAAQLPTHNPEAPIMLDRILRLLATYSVLDCKLNNLADGGVERLYGLA
PVCKFLTKNADGVSMAPLLLMNQDKVLMESWYHLKDAVLDGGIPFNKAYGMTAFEYHGTDPRFNKVFNQGMSNHSTITMKKILEVYRGFEGL
KTVVDVGGGTGATLNMIISKYPTIKGINFELPHVVEDAPSHPGVEHVGGDMFVSVPKGDAIFMKWICHDWSDDHCRKLLKNCYQALPDNGKVI
LAECVLPEAPDTSLATQNVVHVDVVMLAHNPGGKERTEKDFEALAKGAGFKEFRKVCSAVNTWIMELCK
>Vitis vinifera_XM_003634113.2
MGSTSMTPTTILESDEEASLFSMQLVSASVLPMVLKSALELDLLEIIAKAGPGAFVSTSEIAAQIPTHNPEAPVMLDRILRLLATYAVVKCSLRNLP
DGGVERLYGLAPVCKYLTRNEDGVSVAPLLLMNQDKVLMESWYYLKDAVLDGGIPFNKAYGMTAFEYHGTDPRFNKVFNNGMSGHSTITMK
KILEAYKGFEGLTSIVDVGGGTGATLNMIISKYPTIKGINFDLPHVIDDAPSYPGVENVGGDMFVSVPKGDAIFMKWICHDWSDAHCLKFLKNCY
QALPDNGKVIVAECILPVAPDTSLATKGVVHIDVIMLAHNPGGKERTEKEFEALAKGAGFQGFKVVCCAFNTWIMEFCKTA
>Nicotiana tabacum_XM_016609679.1
MGSTSESQSNSLTHTEDEAFLFAMQLCSASVLPMVLKSAVELDLLELMAKAGPGAAISPSELAAQLSTQNPEAPVMLDRMLRLLASYSVLNCT
LRTLPDSSVERLYSLAPVCKYLTKNADGVSVAPLLLMNQDKVLMESWYHLKDAVLDGGIPFNKAYGMTAFEYHGTDPRFNKVFNRGMSDHST
MSMKKILEDYKGFEGLNSIVDVGGGTGATVNMIVSKYPSIKGINFDLPHVIGDAPTYPGVEHVGGDMFASVPKADAIFMKWICHDWSDEHCLKF
LKNCYEALPANGKVIIAECILPEAPDTSLATKNTVHVDIVMLAHNPGGKERTEKEFEALAKGAGFTGFRKACCAYNTWVMEFNK
>Lamium album_Cluster-9669.0
MDEEGAFLSAMRLASGSVLPMVLKSAIELDLLQLIKKADSASATELASQLPTNNPEAATMIDRILRLLATYAVVNYSLTTLPDGGVERRYSLAPV
CRFLTRNEDGVSLAPVSLMLQDKVIMESWYHLKDAILEGGVAFERAYGMNAFEYPATDPRFNKVFNEAMHQQSTIFMKRILQIYKGFEGLNSL
VDVGGGIGASLGMITAKYPSIQAINFDLPHVIQDAPYIPGVKHVDGDMFVRVPKADAIFMKWICHDWSDAHCQKLLKNCYEALPENGKVIIAETN
LSDDPNCGPVIVGDMIMLTLNPGGKERTEKEFQTLAKQGGFKQLVKVCSAYNIWIMELHK
>Lamium galeobdolon_TRINITY_DN2990_c0_g1_i1
MSSTTKNLETSTVNYDEEESFLFGMQLASASVLPMVLKTAIELNLLELIKKAGPGAFVSPAEIAAQLPTTNPEARVMLDRILRLLTTYSVVNCSLK
TLPDGGVERLYGLAPVCRFLTKNDDGVSMAPLLLMNQDKVLMESWYHLKDAVLDGGIPFNKAYGMSAFEYHGTDPRFNKVFNQGMSNHSTM
TMKKILETYRGFDGLKTVVDVGGGTGATLNMIVSKFPSIKGINFDLPHVIEDAPSYPGVEHVGGDMFVSVPKGDAIFMKWICHDWSDEHCIKFL
KNCYDALPQNGKVLLAECVLPEEPDTGFATKNVVHIDVIMLAHNPGGKERTEKEFHNLAKAAGFKGFRKACCAYNTWIIELLK
>Lamium galeobdolon_TRINITY_DN5298_c0_g1_i1
MDSHSSIAPSDEEACAFAMQLVTASVLPAALKTAIDLDLLELINKTGPVSPADLAAQIHTTNSDAPAMLDRILRLLASNNILDCKLRTLPHGGVER
LYALSPLSKFFTKNKDGHLKDAVVEGGVPFERAYGMTAFEYPATDPRVNEVFNRGMSEQSAIFTERIVEMYKGFEGLKTLVDVGGGIGTSLKMI

TSKYPSIKAINFDLPHVIHHAPSLPGVEHVSGDMFLKVPSGDAIFMKWISHDWGDAQCLTLLKNCHEALPDNGKVIIADRILPEIPEKNLLASIDSA
VDLLMLAYYQGGKERTETEFQAIAKASGFKEFRKVCSCFNIWIMELYK
>Lamium maculatum_Cluster-9204.4402
MGSTTKNHETSTVNSDEEESFLFAMQLASASVLPMVLKTAIELNLLELIKKAGPGAFVSPAEIAAQLPTTNAEARVMLDRILRLLTTYSVLNCSLK
TLPDGGVERLYGLAPVCKFLTENDDGVSMAPLLLMNQDKILMESWYHLKDAVLDGGIPFNKAYGMTAFEYHGTDSRFNKVFNLGMSNHSTMT
MKKILETYEGFDGLKTVVDVGGGTGATLNMIVSKFPSIKGINFDLPHVIEDAPSYPGVEHVGGDMFVSVPKGDAIFMKWICHDWSDEHCIKFLK
NCYDALPQNGKVLLAECVLPEEPDTGLATKNVVHIDVIMLAHNPGGKERTEKEFHNLAKAAGFKGFRKACCAYNTWIIELLK
>Lamium maculatum_Cluster-1448.0
MNNDSSPSDKEAFAFAMKLTTASVLPAALKTAIELDLLELIKKAGPTSPAELAAQIHSTNPDAPAMVDRILRLLASNNILDCKLRTLPNGGGVERL
YALSPLSKFLTKNKDGVSLAPLLLVVQDKVFMEPWQHLKDAVVEGGVPFERAYGMSAFEYSATDPRLNEVFNRGMSEQSTIFMEKIVQVYKG
FEGLKTLVDVGGGIGTSLKMITSKYSTIKAINFDLPHVIQRAPSLPGVEHVSGDMFLKVPSGDAIFMKWILHDWGDAECLKLLKNCHEALPDNGK
VIIADRILPEIPENNLLASIDSAVDLVMLAYYQGGKERTETEFQALAKASGFNQFRKVCSCFNIWIIELYK
>Lamium orvala_Cluster-2218.10006
MDEEAAFLSAMRLASGSVLPMVLKSAIELDLLQLIKKADSASASATELASQLPTSNPEAATMIDRILRLLATYAVVNCSLTTLPDGGVERRYSLAP
VCRFLTTNEDGVSLAPISLMLQDKVIMESWYHLKDAILEGGVPFERAYGMNAFEYPGTDPRFNKVFNEAMHQQSTIFMKRILEIYKGFEGLESL
VDVGGGIGASLRMITAKYPSIQAINFDLPHVIQDAPSIPGVKHVDGDMFVRVPNADAIFMKWICHDWSDAHCQKLLKNCYEALPENGKLIIAEINL
SDDPNCGPIAVGDLIMLTLNPGGKERTEKEFQTLAKQAGFKQFIKVCSAYSIWIMELHK
>Lamium orvala_Cluster-2218.2791
MDKDSDEEAAFAFAMNLTTASVLPAALKTAIELDLIELIKKAGPTSPAELAAQIHTTNPDATAMVDRILRLLASNNILDCKLRTLPHGGVERLYALS
PLSKFLTKNKDGVSLAPLLLLVQDKVFMKTWQHLKDAVVEGGVPFERAYGMTAFEYPATDPRVNEVFNRGMSEQSTIFMERIVEMYKGFEGL
KTLVDVGGGIGTSLKMITSKYPSIKAINFDLPHVIQHAPCLPGVEHVSGDMFLKVPSGDAIFMKWILHDWGDAQCLTLLKNCHEALPDNGKVIIA
DRILSEIPENNLSASIDSAVDLIMLAYYQGGKERTETEFQALAKASGFNEFRKVSSCFNIWIIELYK
>Zea mays_XM_008679909.3
MGHQAQHATDDTEELLAAHRELWCHALGYVKSMALKCALDLRIPDIIQRCGGSATLGQLLAASEVPASNLGYLRRVMRTLTAMRIFAVVGHGH
GPDKADDPADDDATAVSYRLTPASRLLATSDDDDDASKNNLSIHPNISSHVRPKNVSLLFSMAEWMKDEQALSVSLYETVNRKCMWACVEDD
AATRACFYESMDADTRLVMQAVIRKCPAVFDDGLTSLVDVGGARGTAAAAVVAAFPHIQKCTVMDLPHIVAEAPAGTGLCFHGGDMFEHIPSA
DAVMLKWILHDWDDDKCVKIMERCKEAISGKEGRGGKVIIIETVLGSRPDDDATCKETYVLDLQILSFVNGAEREEHEWRRIFLAAGFRDYKITH
TRGIPSIIEVF
>Sorghum bicolor_JX673942.1
MVLISEDSRELLQAHVELWNQTYSFMKSVALAVALDLRIADAIHRCGGAATLSQILGEIGVRPCKLPALHRLMRVLTVSGTFTIVQPSAATMSLV
SDGNELVYKLTTASRLLVSSESSATASLSPMLNHVLSPFRDSPLSMGLTAWFRHDEDEQAPGPCPFTLMYGTTLWEVCSRDDAINALFNNAM
AADSNFLMQIVLREFGKVFHGIDSLVDVGGGVGGATMAIATAFPSLKCTVLDLPHVVAKAPSSSIGNVQFVGGDMFESIPPANVVFLKWILHDW
SNDECIKILKNCKQAIPSRDAGGKIIIIDVVVGSESSDTKLLETQVMYDLHLMKIGGVERDEQEWKKIFLEAGFKDYNIIPVLGLRSIIELYP
>Zea mays_BX10a
MALMQESSSQDLLQAHDELLHHSLCFAKSLALAVALDLRIPDAIHHHGAGGATLLQILAETALHPSKLRALRRLMRVLTVTGIFSVVEQPPAGGG
DDSTVHTSDDEAVVVYRLTAASRFLVSDDVSTATLAPFVSLALQPIAACPHALGISAWFRQEQHEPSPYGLAFRQTPTIWEHADDVNALLNKG
MAADSRFLMPIVLRECGETFRGIDSLVDVGGGHGGAAATIAAAFPHLKCSVLDLPHVVAGAPSDGNVQFVAGNMFESIPPATAVFLKKTLHDW
GDDECVKILKNCKQAISPRDAGGKVIILDVVVGYKQSNIKHQETQVMFDLYMMAVNGVERDEQEWKKIFTEAGFKDYKILPVIGDVSVIIEVYP
>Zea mays_BX10b
MALIMQESSSHDLLQAHDELLHHSLCFAKSLALAVALDLRIPDAIHHHGAGGATLLQILAETALHPSKLRALRRLMRVLTVTGIFSVEQPPAGGG
DNSTVHTSDDEAVVVYRLTAASRFLVSDEVSTATLAPFVSLALQPIAACPHALGISAWFRQEQHEPSPYGLAFRQTPTIWEHADDVNALLNKG
MAADSRFLMPIVLRECGETFRGIDSLVDVGGGHGGAAAAIAAAFPHLKCSVLDLPHVVAGAPSDGNVHFVAGNMFESIPPATAVFLKKTLHDW
GDDECVKILKNCKQAISPRDAGGKVIILDVVVGYKQSNIKHQETQVMFDLYMMAVNGVERDEQEWKKIFIEAGFKDYKILPVIGDVSVIIEVYP
>Setaria italica_XM_004962055.4
MAHPGAGDHQEPQARHDATDAEELLQAHLHLWSHALGYVKSMALKCALDLRIPDAIQRCGGAATLDDLLAATGLPPSSLPFLRRLMRALTASR
IFSLRQDPADDPAAVSSYHLTATSRLLLSGGDDASCFSLFPAIYPLVQEGLVSPMLRMHEWMGRHDAASTSLYEVAHGKGLWDTLQASAAYR
AAFQDAMDADTRLVMHAVLGGSPAVFRGLTSLVDVGGGRGRAAAAIAGAFPHIQCTVMDLPHVVAEAPAGNGVCFLAGDMFEHIPSADALLL
KWILHDWDDANCIKIMQRCLEAIGGKEGRGKVIIIDAVIGSVPNDDDAICREAQVLCDLQIMAAFNGAEREEHEWRRIFLEAGFCDYKITRIRGVK
SIIEVYP
>Setaria viridis_XM_034728629
MAHPGAGDHQEPQARHDATDAEELLQAHLHLWSHALGYVKSMALKCALDLRIPDAIQRCGGAATLDDLLAATGLPPSSLPFLRRLMRALTASR
IFSLRQDPADDPAAVSSYHLTATSRLLLSGGDDASCFSLFPAIYPLVQEGLVSPMLRMHEWMGRHDAAATSLYEVAHGKGLWDTLQASAAYR
AAFQDAMDADTRLVMHAVLGGSPAVFRGLTSLVDVGGGRGRAAAAIAGAFPHIQCTVMDLPHVVAEAPAGNGVCFLAGDMFEHIPSADALLL
KWILHDWDDANCIKIMQRCLEAIGGKEGRGKVIIIDAVIGSVPNDDDAICREAQVLCDLQIMAAFNGAEREEHEWRRIFLEAGFCDYKITRIRGVK
SIIEVYP
>Brachypodium distachyon_XM_014896896.2
MEEQQRTEELLQGQLELWHHALGYIKSMALKCAMDAGIPDAIARCGGGATLPELLAATGLPPCNLSYLRRLMRVLTVSGIFHHQRKQPASGEH
EEEAVYALTRASSLLVRGEGNGGAPSFGQLPFISILLGRTFVTPFFGMHPWIADERAAATSFFEREHGQALWEMARTSTGINDEFNQGMTADT
RLIMHAVLTLSPAVFEGLTSLVDVGGADGTAAAAVARAFPHINCTVLDLPHVIENAPAPDEAAGGNTVRFVAGDMFHHIPSAHAVLLKWILHDW
NDDECIKILRECKKAIPTRGNGGKVIIIDTVVGSNTCDNICNETQALFDMYIMFINGVEREEREWKRIFLEAGFINYKITSTLGYRSIIEVYP
>Triticum aestivum_XM_044595926
MSALMQQKELAMSTDELLQAQVELYHHSFAFVKSLALKAATDLCIPDAIQHLGGAATLSDLAAETGIHPTKLSNLRRLMRVLTTSGVFSVTGDH
AGAVYKLTRVSRLLVVDRAGGRTHLSPVVSAFVSPYPVTTLFSMREWFTDERAAAMSLFEVAHGCTLWEAAAMDGTAFNAAMAADSRFTME
NLLRECGSVFETVRGSLVDVGGGHGAAAAAIARAFPHVKCTVLDLPHVVATAPTDDKLTFVGGDMFDNVPPADAVLLKWVLHDWQHEDCVKI
LRRCKEAIPARNVGGKVIIVDTVIGSAGSQQGIASKETEVLFDVFMMAADGVEREEHEWSKIFIEAGFTDYKITPTMGLRSIIELYP
>Setaria italica_XM_004975346
MAITKDSKELLQAQLELWHHAFGYVKSMALAVALDLGIADAIHRHGGAATLSQILAEAKLSPCKLHGLRRLMRALTVAGTFTIATSSGHGGDDE
DIYELTPASRLLISDDVAGDDGEPSLSPVLSLVLNPFRVSPLGMGIGAWFRQGDQPGVAPFAVAHGKNMWEMAARKPAFNALVNDAMAADSR
FLMRIVLRECAEVFHGVSSLVDVAGGLGGAATSIAKAFPELRCSVLDLPHVVANAPSGGNVQFVAGDMFQSIPQADAVFLKWILHDWGDDECI
KILKNCKQAIPSRDKGGKVIIIDMVVGSESSDTRHLETQVLYDLLIMGINGVERDEQEWKKIFLEAGFNDYKIMSILGVRSIIELYP
>Aegilops tauschii_XM_020320754

MSALMQQKELAMSTDELLQAQVELYHHSFAFVKSLALKAATDLCIPDAIQHLGGAATLSDLAAETGIHPTKLSNLRRLMRVLTTSGVFSVTGDH
AGAVYKLTRVSRLLVVDRAGGRTHLSPVVSAFVSPYPVTTLFSMREWFTDERAAAMSLFEVAHGCTLWEAAAMDGTAFNAAMAADSRFTME
NLLLECGSVFETVRGSLVDVGGGHGAAAAAIARAFPHVKCTVLDLPHVVATAPTDDKLTFVGGDMFDNVPPADAVLLKWVLHDWQHEDCVKI
LRRCKEAIPARNVGGTVIIVDTVIGSAGSQQGIASKETEVLFDVFMMAADGVEREEHEWSKIFIEAGFTDYKITPTMGLRSIIELYP
>Panicum virgatum_XM_039916653
MAIAEESKELLQAQLELWHHAFGYVKSMALAVALDLGVADAIHRRGGAATLSQILADAALSPCKLHGLRRLMRALTVAGTFTVANTTSSEDDSG
GEAVYELTPASRLLVSDSVSGGDGDGDGDGDGSTAASLSPVLRLVLNPFRVSPLGMGIGAWFRRDDQPGVAPFAVAHGRDMWETAARKPA
FNALVNDAMAADSRFLMRIVLRECAEVFHGIGSLVDVAGGLGGAATSIARAFPELRCSVLDLPHVVASAPSGGGNVQFVEGDMFQSIPPADAV
FLKWILHDWGDDECIKILKNCKQAIPSRDEGGKVIIIDMVVGSGSSDAKQLETQVLYDLLIMGINGVERDEQEWKIFFEAGFKDYKIMPILGVRSVI
ELYP
>Zea mays_GRMZM2G177424_P01
MEPQCNVGMFTRAELLEASVELRHHALGCVKSTALRCAVKLGVANAIQRRGGRASVEDLLTELSLDASRLRCLHSVMRALAALGVFKEGSDG
EYGLTAISSLLVDDDSSVRGSLRPITLLYLEPAFVAPVLNLADWALAGVDGSDDDNNCSARGTAFKMTHGEDVWDVLARDAFLGDFFNGALAS
ETRFLMDIAIRGSPQVFEGIASLVDAGGGTGAAAQAVAAAFPDTRCTVLELPQVVDAAPIDGPVRFVGGDMTKFIPPADAVLLKNVLHDWSDKD
CVIILKRCKEAIAASGKVIVIDIVLGSSSLAICNETQLWLDLFMSTVTTGKERREEEWYRLFKEAGFSAYKISPVLGLLSIIEVFL
>Zea mays_GRMZM2G349791
MATAIVPTDAELLQAQADLWRHSLYYLTSMALKCAVELHIPTAIHNLGGSATLPDLVAALSLPAAKLPFLGRVMRLLVTSGVFASSDDVQYRLNP
LSWLLVEGVESEDHTYQKYFVLGTVSRHYVEAGMSLADWFKKEEDEDRQLPSPFEALHGVPLVHESTKLLDEELDRVVEEGVAAHDNLAIGT
VIRECGADVFSGLRSLTYCCGRQGNASAAAIVKAFPDIKCTVLNLPRVVEETTTKTITIPPAQAVMLKLVLHFWSDDDCVKILELCRKAIPSRQEG
GKVIIIEILLGPYMGPVMYEAQLLMDMLMMVNTKGRQRGEDDWRHIFTKAGFSDYKVVKKIGARGVIEVYP
>Zea mays_GRMZM2G136857
MEMKDDAVVLAARPRPDAAAAVVVVTLRRMNSSGDGRMSYADNSGFQRAIASVTKNARQELAAALYRARGRPSSMVIADLGCATGPNALLM
VLDAVEAVLAVAEESHPSPPPQLHVFLNDLPANDFNAVFRLLPSSPLAATGCCFVSAWPGSFYERVFPEASLDYVVSSSSLHFLSMAPTMRTE
HPNRGRVYVSESGPAAVLDAYRSQFHADFLAFLSCRADEMRPRGLLILTFVARRTARPTAHDCYLWDFLADALMDMAADGLVDEEQVHAFNV
PFYGPCLDDLAKVIAREGSFTVRTMQLFDISRRCLLLQSASDDDDDLPQWLAMETTSTVRAVVEPMLRAHFGLEAMDGLFCRYSLRLEEYYRS
NTSRNKDDLTNVFLVLEKKLH
>Zea mays_GRMZM2G331103
MSRLVVDDHEEIKRLDRELDMEVILQWRMLAHKFVEQSVETYQYAQQNKKSWWTGSSKDEEDSKSFTDEDWERLNRIIGYKETNEYVTDQQ
DMKLMQFDFEIRMKHNASKLTIDDSECLADLSCQDFCCNLKMYPEAKIFDLKLGSYRLLSPYGLLAESASVIDSFVGLFSYKPFDEQLDWSLTA
KASPCYITYLKDSVDQIVGFFKSSPTISQNLALETAAAVQMTLDEVKRTAQQQMTRVLKDQSSSLGWCPKKLVAYFLLQKLRRGENLPSLHRN
SAVVDGLVWALSTIPQIPSAKIMVMKAIHNTSDTNQLNREDLETYAKLFCHPLSYSQIQALAALFGWAVPDDLGGAATMVC
>Salvia hispanica_XM_048112964
MAFQNGEVSTADLLQAQAHVWNHMLAFVSSMSLKCAIQLGIPDAIHKHGEPITLSRLVESVNADVSKSQCIHRLMRVLVQSEFFTEVANQEEV
RYWLTPSSRLLLKEAPLTVAPLALLMLDPVLTDSWHHMSEWLTSENHLTQFEAVHGSMFWEHVAHGPGMGTLFDEAMRSDSRLVASVLVRD
YKHVFEGIKSLVDVGGGTGTMAKAIVEAVPSIKCIVLDLPHVVAGLEGTSKLSYVGGDMFEAIPPADAVLLKWIMHDWDSDNCLKILKRCKDIVK
SNGSTGAKVIIIDAVLDINREDDKVVNDQLYFDMTMMAYFNGIERSEKEWAKLFSDAGFTSYKITHAFGVRSLLEVYP
>Malus zumi_KJ156528
MSLSNEVGATSHELLGAQAQLWNHIFQFINSMSLKCAVQLGIADVIHNHGQPISLSELMAGLKVHPSKAHFVSRLMRILVHSNFFAQHHHVHHD
RADVEEEETVVLYSLTPASRLLLKDGSLNTTPFLLMILDPVVTTPFHLMGAWLKINGGDDPAATCTPFEMENGMPFWELGAREPRFGNLFNEA
MEADSKLIGRVVVEECGGVFEGLKSLMDVGGGSGTMAKAIANAFPNINCTVFDQPHVVAGLQGTTHNLGFMGGDMFEEIPPADAILLKWIMHD
WNDEESVTILKKCREAISLSKNEGGNKKIIIIDIVVGYVDNKKKMMDKKSIETQLMFDMLMMSILPGKERSKSEWEKIFFAAGFTHYNITHTLGFR
SLIEVYP
>Citrus depressa_LC126056
MNLIDGEHDTELLEAQAHVWNHIFNFINSMSLKCAVELSIPDIINKHGKPMTLNKLVSALTINPSKTRCVYRLMRILIHSGFFAQQTLNSSRNNND
EEQGYVLTNASKLLLKDSPLSVTPFLQAMLDPILLSPWLKLSTWFQSDDPAPFDTAHGKSFWEYAGDEPKINNFFNEAMASDARLATRVVIHKC
KDVFEGLNSLVDVGGGTGTVAKAIAIAFPNLECTDFDLPHVVNGLESDLANLKYVGGDMFEAIPPADAVVLKWILHDWNDEECVKILKKCKEAIT
SDGKKGKVIIIDMMRENKKGNDESTETQLFFDMLMMVLATGTERDEKEWAKLFADAGFSDYKITSILGLRSLIEIYP
>Traes_2BL_C467B516F.1
MPAAQHIERDQDLAMSSDELLQAQLELYHHGLAFVKSLALKAATDLRIPDAIHRRGGAATLSELASDTGIHPTKRSNLRRVMRVLTTTGVFSIVQ
GKGSNDHAGDGAAYYKLTRVSRLLVERSPHNLSPMVGTIVNTLCWTSLLKMPEWFTQGQEGESAQSHSLVQLANGCTFWDTTKVDGGLFND
GMAADSRIAMKVLLKEHGGAFGEVKSSLVDIGGNHGATASAVARAFPHLKCSVLDLPHVVAAAPASDILTFVAGNMFEYVPPADAVLLKWILHD
WKHEDCVKIMRRCKEAIPAKEAGGKVIIIDMVVGYPVTQPNHSKEAQVLLDIYMMGSDGMEREENEWSLIFSEAGFSDYKITPTNGIRSIIEVYP
>Traes_4AL_C467B516F.1
MPAAQHIERDQDLAMSSDELLQAQLELYHHGLAFVKSLALKAATDLRIPDAIHRRGGAATLSELASDTGIHPTKRSNLRRVMRVLTTTGVFSIVQ
GKGSNDHAGDGAAYYKLTRVSRLLVERSPHNLSPMVGTIVNTLCWTSLLKMPEWFTQGQEGESAQSHSLVQLANGCTFWDTTKVDGGLFND
GMAADSRIAMKVLLKEHGGAFGEVKSSLVDIGGNHGATASAVARAFPHLKCSVLDLPHVVAAAPASDILTFVAGNMFEYVPPADAVLLKWILHD
WKHEDCVKIMRRCKEAIPAKEAGGKVIIIDMVVGYPVTQPNHSKEAQVLLDIYMMGSDGMEREENEWSLIFSEAGFSDYKITPTNGIRSIIEVYP

Sequences for Polyphenol oxidases (PPOs) phylogenetic analysis

>Mimulus_1 guttatus_Migut.M00643
MTSLYLSCATTNTAISATPSSHPRPLLPKPSHSHFITHAKLTNRLHHVSCSSTPQNQNDETKKPTAKVVDRRNMLVGMGGLYGATTLASTPSA
AANPIQAPELNKCGTATNLNNGEKLDINCCPPISDRIIDYKLPPVFQMKIRPSAHRVSPEYMFKFNTAIDRMKRLPKDDPRNFMQQANIHCAYCN
GAYDQPGQGTLDLQIHNSWLFFPFHRWYLYFYERILGKLIGDPTFALPFWNWDNPKGMTMPPMFVDPKSSLYDKKRNQEHLPPAVVDLGLTN
STDTLQVVANNLTIMYSEMIRGNSDANDFMGQAYREGDESSAKAAGSSERGSHTSVHAWVGDFKDQPSGEDMGNFYSAGRDPLFYSHHAN
VDRMWTLWQYFLPSNKVPDKRITDTDFLNTAFLFYDENAQLVRVTVKDCLDNLRMGYDFERIDLPWLDYRPPRQSARAKINRASSTAAPKAAA
LFPLTLDGVVRFQVDKTKKGKADESLVLEDIKVDTTKLLKFDVFVNDEDDNPGELDKAAYLGTYAQVPHKSANNNSSTSSIKLKLTELYDDMDID
DDDTIVVTLVPRHQGPVLPLVVSRLSRIHRNRLPVVLDH
>Mimulus_1 guttatus_Migut.M00634

MASLPLSCYTTTQFSSSISTQPPPRQRFSAQTPPPTRRFQVRSTGGPNNEKGENSPPNGGKFDRRNLLMGMGGLYSAANLVPTDSPASATPI
SSPDLSKCSRGTDLSTQQPLDVNCCPPVVDGITNYKLPPVTKMRFRPAAHLAGKEYIAKYEKAVAAMKALDKTDPTDPRGYSQQANVHCAYC
NLTYEQSEDSDTKLQIHNSWHFYPWHRWYLYFYERILGSLIDDPTFGLPFWNWDNPPGMVMPDMFAVTTSPLYDARRNLEHLPPAAADLNFA
SKPTTDPSKIVTNNLTTMYGEMVRNVSKLEDFYGAKYVVGTAPDPGPGTVERGSHTALHVWVGENTPTGEDMGNFYSAGRESLFYSHHANID
RLWSTWQGLRGKKPKGFKDKDWLDATYLFYDENKNLVQVNVKDALDNEKMGYTYQKVDNPWVNYRPKPKVKKSKAAKNSKAPTATDTIFPV
KLDKVVKVLVDRPRKSRSKKDKEDEEELLVIEGIEVDNSKFVRFEVFINDEDDKPEDLRDKAEFAGAYSQVPHKNSTRTKSKIRLGLTELLEDLD
VDDDDQILVALVPKAGGEDITIGGIKIIYAS
>Mimulus_1 guttatus_Migut.M01175
MACSIKNTPHIATTGAGGAASSPLLVKPTHIPTIRKPSHRHIISCKSTNNHDQEPAGKFDRRDVLIGLGGLYGATTLSASPFAFAAPISAPDVTKC
GPADLPQGAAPTNCCPPPTATIVDYKFPPPSTTMRVRPAAHLADKAYVAKFNRAMELMRALPDDDPRSFKQQAAVHCAYCDGAYDQAGFPN
LELQVHNSWLFFPFHRYYLFFFERILGKLIDDPTFAMPYWNYDSPAGMTIPAMYTNPSSPLYDKLRNQGHQPPALIDLNFSGSGTITAEQQVKR
NLTVMYRQMVSNSKTPRLFFGSAYRRGEDPNPGSGSVENIPHGPVHVWTGDPTQPNGENMGNFYSAGRDPIFYAHHSNIDRLWSLWKGLG
GRRQDITDPDFLDASFLFYDENGQMVRVKTRDCLDNTKLGYTYQDVEIPWLKSRPTPKVSSVLKKLKKLGKAKAAAAETHRPPKEVLPAKLDK
VLKIMVKRPKKKRSKKEKDELEEILIIQGIELERDVYAKFDVYINDEDDETSTEENTEFAGSFVNVPHKHKHGKKIKTQLRLSITDILEDLDAEDDE
HVLVTLVPTNAGDSITIHGVKIELDD
>Mimulus_1 guttatus_Migut.M00639
MASSVQSSCTFLLATNHRHSRPPPSSPHSLTPAGRHRRSHLFQVSCKQQNPPPPSSEGKVDRRNMLLGLGGLYGAANLIPTNPEAATASPIE
PPEFKACGAARDVQSGDLLNINCCPPTSDKIVDFKLPPPPAKLRVRPPAHRVSKEYLAKFEEAIRRMKALDKSDPSDPRGFMQQANIHCAYCN
GAHDQVGYKDLDLQVHNSWLFFPFHRWYLYFYERILGSLIDDPTFVLPFWNWDNPKGMQMPKIFDNSGSPLYDANRNQKNRPPAIIDLGFSG
ATEDLQVVVNNLTVMYSEMIRSVNSTLDFMGQPYKAGDAASPGMGSSERGSHVAAHVWVGDPRNKYSEDMGNFYSAGRDVLFYCHHANVD
RMWSVWRTLKTDVPKDIKDPDYLNAAFLFYDENKQLVRVKVGDCLDHKKMGYEFEKVDTPWLNYKPTKKAAKAKILKIAAGAETVEALFPLNL
KKITRVLVNKSAKGKADEVLVLENIETDTTKFIKFDVFVNDEDDGSVELDKAEYAGTFAQVPHKTKDKKGKSTISLRLTELYEDVDVADDDTVVV
TLIPRSNGDDVTIGGIKIIASPPRSG
>Mimulus_1 guttatus_Migut.M00644
MASLHQVLPAAAPSSSSLRSSSSTATRPVVFAKPSHFLTHAKRNHHRRLISCSSSSSNEGSKVDRRNMLLGLGGMYGAANLLSTPDSASANPI
QAPQIDKCGNSTYDTSAGVATQINCCPPFTGTIIDYKLPNFPKTKVRPSAHRLSPEYLFKFNTAVDRMRRLPKDDPRSFMQQANIHCAYCNGG
YDQPGQGTLDLQVHNGWLFFPFHRWYLYFYERILGKLIGDPTFALPFWNWDNPKGMTIPPAFIDPKSALYDAKRNPDNMPPAVVDLGFTGST
DPLQVVSNNLTIMYTEMVRGNSDVYDFMGKAYREGTPVNPGPGSSERGSHTAIHAFVGDYKNQPAGEDMGNFYSAGRDPLFYSHHSNVDR
MWTLWQFFLPSNKIPDKKITDPDFLNTAFLFYDENAQLVRVTVKDCLDNLKMGFEYERVDLPWLDYRPPAQAASAKVTRAGKTTVKVEDTVFP
IVLDKIVRVLLPKTKKGKADELLVIENIVVDTSKFLKFDVFVNDEDDNVAEIDKASYAGTYAQIPHKTKEKTATTSIRLKLTDVYEDMDVTDDDTILV
TLVPRHEGPGVTIGGIKIIENPTPAPAAAAAAPAS
>Mimulus_1 guttatus_Migut.M00642
MTSLYLSCATTTTASRHPFLKPQHVITTHAKPSHPLHQISCSSTTSQNQNDDTKSQSGKVVDRRNVLLGMGGLCGAANLVSNTPAASANPILP
PELNKCGTATNWNNGESLDGINCCPPLTTDIIDYKLPPVFRMKIRPSAHRASAEYMYKYNLATDRMKRLPADDPRNFMQQANIHCAYCNGAYD
QPGQGTLDIQIHKCWLFFPFHRWYLYFYERILGKLIGDPTFALPFWNWDNPKGMTIPPMFVEDPRSALYDRKRNQKNLRAVVDLGLTDATDTL
QVVSNNLTIMHNEMIRGISDPLDFMGARYSEGSDPNPGEGSVERGSHTSIHAWVGDPEQPSGEDLGNFYSAGRDPLFYCHHANVDRMWYL
WQRLPNNKLPDKRITDPDYLNASFLFYDENAQLVKVYVRDTFDNRRMGFDYERIDLPWMDYRPPLQAARTKINKVDAPNAKTLFPLKLDRVVR
FQVDKTKKGKADEILVLENITVDASKLLKFDVFVNDEDDNPSELDKASYLGTYAQLPHKPRSGTTSNSSSIRLKLTDLYDDIDIGDDDAIVVTLVP
RHQGPGVTIGGIKIIENPTKPNPTAAAATDATTTTATSSG
>Mimulus_1 guttatus_Migut.M00640
MASLYLSCATSTTAASRHPFLKPSHVITTHAKPSHPLHQISCTSTTSQNQNDDTKPQGGKVVDRRNVLLGMGGLCGAANLVSGNTPSASANPI
VPPELNKCGTARNWNNGGKPLDDINCCPPLTTDIIDYKLPPVFHMKIRPSAHRVSAEYMYKYNLATDRMKRLPADDPRNFMQQANVHCAYCN
AAYDQPGQGALQIQIHKCWLFFPFHRWYLYFYERILGKLIGDPTFALPFWNWDHPRGMTMPPMFVEDPRSALYDRKRNQKHLPPAVVDLGKT
DSTDTLQLVSNNLTIMHNEMIRCISDPLDFMGARYCEGSEANPGEGSVESGSHTVIHSWVGDPEQPTSEDLGNFYSAARDPLFYCHHANVDR
MWYLWQRLPNNKLPNKRITDPDYLNASFLFYDENAQLVKVYVRDTFDNRRMGFDYERIDLPWVDYRPPPQAARTKINKVVDAPNAKTLFPLKL
DGVVRFQVDKTKKGKADEILVLENITVDASKMIKFDVFVNDEDDNPSELDKASYLGSYAQLPHKPRSGTTSTSSRIRLRLTDLYDDIDIGDDDAIV
VTLVPRQQGPGVTIGGIKIIENPTKPNPTAAAAADADTTTTT
TTMIRFII
>Mimulus_1 guttatus_Migut.H02075
MASLHIPCTTTATTPSSATRPSSRHHFAKPSHLITHAKRNNRLQISCSDSQKQKDSSQNADTPQGNVDRRNVLLGFGGLCGAANLISTHTASA
NPVQAPQIDKCGVATNLNTGKQLDINCCPPMGKNIIDFKPPPLVQMRTRPSAHRVSAEYMFKYNTAIDRMKRLPKDDPRSFMQQANIHCAYCN
GAYDQPGQGTLDLQVHNSWLFFPFHRWYLYFYERILGKLIGDPTFALPFWNWDNPKGMTIPPMFVDPKAAIYDAKRNPANMPPAVVDLGLTG
NKDPLQVVSNNLTVMYSEMVRANSDIYDFMGKPYREGTLVNPGPGASERGSHVAIHVFVGDPREPSGEDLGNFYSAGRDPLFYCHHANVDR
MWTLWQYFLPNNKLIDKKITDPDYLNASFLFYDENAQLVRVTVKDCLDNTKLGYDFERVDLPWLDYRPPPQTAQAKVMRTVSTTRKADAVFPL
KLDGIARVLIQKTKKGKADELLVIEDITVDTSKFLKFDVFVNDEDDNVGELDKAAYAGTYAQVPHKTNEKTATTTIRLKLTELYDDMDVTEDDTVL
VTLVPRHEGEGVTIGGIKIIENPTPAKTS
>Mimulus_1 guttatus_Migut.H02077
MASLALSWSSAAAAAAAASSQLPPPSNSSVKTQTRRCNNITCKATNNDKNTEPTQKLDRRNLLLGIGGVYGGAAHLLSADNPPAAFAAPITSP
DISKCTTGTNLNTQKPLDINCCPPQSAEIIDYQLPPVTQMRYRPAAHLAGKEYYAKFEKAVALMKALPADDPRSFMQQANVHCAYCNLTYPQT
GDPKLKLQIHNCWHFFPWHRWYLYFYERILGNLIGDPTFGLPFWNWDNPAGMVMPERFAVQDSPLFNPRRNQTHLPPTPTDLAYSAKSPTN
PKKIIPNNLTIMYGEMVRNVKKLEDFYGAKYVVGTKPDPGPGSVERGSHTAMHAWVGENTASGEDMGNFYSTGRDPLFYSHHTNVDRLWTI
WRNQRSGPKQPKDFPDSDWLNADYLFYDENARLVRVKNSDTLENEKMGYVYQEVDLPWLKNRPAARVTKSKVAATTAAPPPETVFPVKLDK
VVKVLVSRPKKSRSKKEKESEEELLVIEGIEVDTAKFVKFDVFVNDEDDEAADELDKAEYAGSYSQVPHRNSTTVKTRIRLGLTELLEDLDVEGD
DKILVTLVPKAGGEDITIGGINIIYADDD
>Mimulus_1 guttatus_Migut.H02076
MASLPLSCATTTTTSATPLRHSSPPPLFTKPSLFITRHANGSHHHRGLHVISAQKQNDNPESTHPQGKLDRRNVLLGMGGLYGAAASLVSTPP
EASANPVQAPQLDKCGTATDLNTGKKLDLNCCPPVTDNIIDYKIPPALRMKLRPSAHRVSAQYMFKYNLAVDRMKRLPDDDPRSFSQQAMIHC
AYCNGAYDQPGQGSLDLQIHNSWLFFPFHRWYLYFYERILGKLIGDPTFALPFWNWDNPKGMTIPPMFLDEKSAIYNSKRNQDNLKAIVDLGM
TGNKDPLQVVSNNLTVMYSEMIRGNADVYDFMGQPYREGTAVNPGPGSSERGSHTAIHVFVGDPREPSGEDLGNFYSAGRDSLFYSHHCNV
DRMWTLWQYFLPSNKVPDKKITDPDFLNASFLFYDENKQLVRVSVKDSLDNLKMGYEYERVDLPWLDYRPPPQAAAARITRTSASAAPKAETL
FPLKLDRVVRFEVQKSKKGKADELLVLENITVDTTKFLKFDVFVNDEDDGATELDKAAYAGTYAQVPHKTANKTATTSIRLKLTELYDDMDVGD
DDTIVVTLVPRHQGPGVTIGGIKIIENPTPPPS

>Lactuca_1 sativa_Lsat_1_v5_gn_7_100781
MASFQLVNPFASTTRKLPDSTSSRRLKTHPQKNHRFKVSCNVAQDGNEKLLLVPDSKNLILPKPSLDTLNVDRRNLLLGLGGLYSTVNFTSLPA
AIAAPITTPDISTCIPSEQGFNVQDSVRSNQCCPPMMTTTPKDFVFPKDKTIRVRPAAHRATPEYIAKYKAAIQAMKDLPDDHPHSFVQQAKIHC
AYCNGGYTQVASGYADKQLQIHNSWLFFPFHRWYLYFYERILGKLIDDPTFALPYWNWDNPAGMSFPAFFETDGKRNPVFDAFRNVNHVSPE
TVVDLDYNGSDSGAPCLQQISTNLAAMYKQMISNATDPLSFFGGEFRAGDDPFGNSDPSVGSIEAGCHTAMHRWTGNPRMPNNEDMGNFY
SAGYDPAFYVHHANVDRMWKVWKDLGIKGHTEPTDPDWLNASYVFYDENEELVRVYNKDCVQTENLKYDFELSPLPWLKNRPVAHTKPETT
TKPVEKVKVPDVKFPIKLDKIQKVLVKRPAKNRSQSEKEKATEQLLIKGIKFNVSKFVKFDVFVNDQDDVPTSSASESEFAGSFAQLPHHHGGH
KKLMTSAARFGLTELLEDIGAEDDEYILVTLVPKVGAEDLTVDEIKVELVPIV
>Lactuca_1 sativa_Lsat_1_v5_gn_7_101100
MSSFQSLATFTSITTRTLPNSPSNRRSNSYPKQTHRLKVSCNVAPEDNEKLLVVPETQKLILPKTSLDTLNVDRRNLLLGLGGLCTTVNFTSIPT
AFGRPITAPDISSCRASTDGLDLKNAIRTNACCPPNLSKKVKDFVFPNDKSLRIRRAAHKAPEDYITKYKAALKAMRALPDDHPHSFVSQAKIHC
AYCNGGYTQIATGDSDKIIQIHNSWLFFPFHRWYLYFYERILGKLINDPTFAIPYWNWDNPAGMTLPAFFEEGNNRKEKLENPAFDAFRNTSHF
APTIVELDYQGEDSGAPSAKQININLTQMNSQMIRNAHDTRSFFGGKYVAGSDPIPNGDRCVGSIEAGCHTAIHRWVGDSRTFNNEDMGNFYS
AGYDPLFYVHHANVDRMWVEWKGLDKRNKEPKDEDWLNASYVFYDENEELVRVYNKDCVRNDKLRYAYEFSPLPWLNNRPTPRTLKSKIAL
KSVGTVKQVEDTKFPLKLDKITKVLVKRPATNRSQEEKEKAVELLLIKDVKYNGGKFVKFDVFVNDQDDVRASSAEESEFAGSFAQLPHGPGD
DMLMTSGARFGLTELLEDIQAEDDELILVTLVPKAGCEEVTVGEIKVELVPLDD
>Lactuca_1 sativa_Lsat_1_v5_gn_9_102161
MMASLSLSTLPTSTPTKKPLFSKTSSHVKQSHRFKVSCNSAANNNEKTVKNSETPKLILPKTPLEMQNVDRRNLLLGLGGLYGAANLTSIPSAF
GTPIAAPDNISDCVTASSNLQNANDAVRGLACCPPVLSTDKPKDYVLPTNPVLRVRPAAQRATDEYIVKYKAAIQAMKNLPDEHPHSWKQQAKI
HCAYCNGGYNQEQSGFPDIQLQIHNTWLFFPFHRWYLYFYERILGKLINDPTFALPYWNWDNPTGMVLPAMFETDGKRNPIFDPYRNATHLPP
AIFEVGYNGTDSGATCIDQISANLSLMYKQMITNAPDTTTFFGGEFVAGDDPLNKEFNVAGSIEAGVHTAAHRWVGDPRMANSEDMGNFYSA
GYDPLFYVHHANVDRMWKIWKDLGIKGHTEPTSTDWLDASYVFYDENEELVRVYNRDSVNMTAMGYDYERSEIPWLHSRSVPHTKGANVAA
KLVGIVKKVEDVTFPLKLNETVKVLVKRPTKKRNKKNKQEANEMLFLNKIKFDGEEFVKFDVFVNDVDDGVETTAAESEFAGSFSQLPHGHKH
GTKMSMTSGAAFGLTELLEDIEAEDDDSILVTLVPKIGCDDVTVGEIKIKLVPIV
>Lactuca_1 sativa_Lsat_1_v5_gn_9_102320
MASLALSSLPTSTTTKKPLFSKTSSHVKPFHRFKVSCNAPADNNDKTVNNSDTPKLILPKTPLETQNVDRRNLLLGLGGLYGAANLTTIPSAFGI
PIAAPDNISDCVAATSNLRNSKDAIRGLACCPPVLSTNKPMDYVLPSNPVIRVRPAAQKATADYIAKYQQAIQAMKDLPEDHPHSWKQQGKIHC
AYCNGGYNQEQSGYPNLQLQIHNSWLFFPFHRWYLYFYEKILGKLINDPTFALPYWNWDNPTGMVIPAMFEQNSKTNSLFDPLRDAKHLPPSI
FDVEYAGADTGATCIDQIAINLSSMYRQMVTNSTDTKRFFGGEFVAGNDPLASEFNVAGTVEAGVHTAAHRWVGNSRMANSEDMGNFYSAG
YDPLFYVHHANVDRMWQIWKDIDKKTHKDPTSGDWLNASYVFYDENENLVRVYNRDCVDINRMGYDYERSAIPWIRSRPTAHAKGANVAAKS
AGIVQKVEDIVFPLKLNKIVKVLVKRPATNRTKEEKEKANELLFVNGITFDAERFLKIDVFVNDVDDGIQTTAADSEFAGSFAQLPHNHGDKMFM
RSGAAFGITELLEDIEAEGDDSVVVTLVPRTGCDEVTIGEIKIQLVPIV
>Lactuca_1 sativa_Lsat_1_v5_gn_4_54780
MASFSPSQATSYTSGRRFSSSSTYSSSFSFKSSQVPIARISKHRHAVSCKTLDDDHHHHANSGKLDRRNVLLGLGGLYGTAANFGSNSLAFAD
PIMGPDLSKCGPAELPQGAIPTNCCPPFTTKIIDFKLPPQSNPLRVRPAAHLVDKDYIDKFSKAIELMKALPDDDPRSFKQQANVHCAYCDAAYV
QLGYPDVELQVHNSWLFFPFHRCYLYFFEKICGKLIDDPTFAIPFWNWDAPVGMKIPDIYTDKNSSLYDTLRDAKHQPPTVVDLDYNGFDNNLS
PSEQTSTNLTIMYRQMVSNAKTASLFMGSPYRAGDDPSPGAGSLESVPHNPVHIWTGDRNQPNGEDMGNFYSAGKDPIFFAHHGNLDRLWS
VWKTLGGRRKDFTDNDWLDSSFLLYDENAELNRVKVRDCVDSKNMNYVYQDVELPWLESKPVPRLQKASRNIKKHAHEHIPFAKDVFPASLD
KVIKVRVPRLKKSRTKKQKEEEEEILVIEGIEVKRDEFVKFDVLVNDDDDGTQATAAKTEFAGSFASVPHMHKHGKNWKTKLRIGITDLLEDLKD
EEDHNVLVTLVPKTSGGDISIGGIKIEHEEC
>Lactuca_1 sativa_Lsat_1_v5_gn_4_54680
MASLAQSPTTTTTTGGRCFSSSSTYSSSFSFKSSQVPIARITNHRHAVSCKGALDDDDHHHENSGKFDRRNVLLGLGGLYGAAATFGSNSLAY
AAPIMAPDLTKCGPADLPQGAVPTNCCPPYTTKIHDFKLPPPSTTFRVRPAAHLANKDYIAKFNKAIELMKALPDDDPRSFKQQAAVHCAYCDG
AYDQVGFPDLELQVHGSWLFLPFHRYYLYFFEKICGKLIDDPNFAIPFWNWDAPDGMKIPDIYTNKKSPLYDALRDAKHQPPSLIDLDYNGDDE
NLSRSKQTSTNLTIMYRQMVSSSKTASLFMGSPYRAGDEASPGSGSLESIPHGPVHIWTGDRNQQNGEDMGNFYSAARDPIFYAHHANIDRM
WSVWKTLGGRRNDFTDKDWLDSSFLFYDENAEMVRVKVRDCLDSKKLGYVYQDVEIPWLKSKPEPRLKRALSKIKKLAVARADEHIPFAKDV
FPASLDKVIKVLVPRPKKSRSKKQKEDEEEILVIEGIELKRDEFAKFDVFVNDEDDGMRATADKTEFAGSFVNVPHKHKHGKNVKTRLRLGISEL
LEDLGAEDDDNVLVTLVPKNKGGEVSIKGIKIEHED
>Lactuca_1 sativa_Lsat_1_v5_gn_4_54301
MTSFASSPTKTLTGTASRSERRISSSSNYSSSFSFKSSQVPIARISKHRHAVSCKTLDDDHHHHANSGKLDRRNILLGLGGLYGTAATFGSNSP
AIAAPIMAPDLSKCGPADLPEGAVSTDCCPPYTTKILDFKLPPPSNTFRVRPAAHLANEDYIGKFNKAIELMKALPDDDPRSFKQQANVHCAYC
DGAYDQVGFPDLELQVHNSWLFFPFHRYYMYFFEKICGKLIDDPNFAIPFWNWDAPDGMKIPDIYTNKKSSLYDPLRDVDHQPPSLIDLDFNGV
DENLSPSEQTSKNLTVMYRQMVSSSKTSTLFMGSPYRAGDDASPGSGSIENTPHNPVHIWAGEWKHNNGKNMGKLYSAARDPLFYAHHGNI
DRMWSVWKTLGGRRKDFTDKDWLDSSFLFYDENAELNRVKVRDCLDTKNLGYVYQDVEIPWLKSKPVPRRTKPKQKPKNKNNKQAVARAD
EYIPFAKDVFPASLNEVIKVLVPRPKISRSKKQKEEEEEILVIEGIEVKIDEFVKFDVFVNDEDDGMRATADKTEFAGSFVNVPHTHKHGKNLKTR
LRLGISELLEDLNAEDDENVLVTLVPKTRGSGISIAEIKIEHEE
>Lactuca_1 sativa_Lsat_1_v5_gn_0_46281
MAPDLSKCGPADLPEGAVSTDCCPPYTTKILDFKLPPPSNTFRVRPAAHLANEDYIGKFNKAIELMKALPDDDPRSFKQQANVHCAYCDGAYD
QVGFPDLELQVHNSWLFFPFHRYYMYFFEKICGKLIDDPNFAIPFWNWDAPDGMKIPDIYTNKKSSLYDPLRDVDHQPPSLIDLDFNGVDENLS
PSEQTSKNLTVMYRQMVSSSKTSTLFMGSPYRAGDDASPGSGSIENTPHNPVHIWAGEWKHNNGKNMGKLYSAARDPLFYAHHGNIDRMW
SVWKTLGGRRKDFTDKDWLDSSFLFYDENAELNRVKVRDCLDTKNLGYVYQDVEIPWLKSKPVPRRTKPKQKPKNKNNKQAVARADEYIPFA
KDVFPASLNEVIKVLVPRPKISRSKKQKEEEEEILVIEGIEVKIDEFVKFDVFVNDEDDGMRATADKTEFAGSFVNVPHTHKHGKNLKTRLRLGIS
ELLEDLNAEDDENVLVTLVPKTRGSGISIAEIKIEHEE
>Lactuca_1 sativa_Lsat_1_v5_gn_2_43561
MAPDVTKCGPAELPIGVEPINCCPPITTNILDFKPPQSNTLRVRPAAHLVVNEDYIAKFNKAIELMKALPHDDPRNFMQQANIHCAYCDGSYKQV
GFPNLEHQVHTSWLFFPYHRYYVYFFEKICGKLIDDPNFAIPFWNWDAPDGMQIPDIFTNKNSQLYDPLRDTNHQPPSVVDLDFNGVDKNLSR
SELTSKNLSVMYRQMVSSAKTASLFMGRPYRAGDEPNPGSGSIEVSPHAPVHSWTGDPKQKNWEDMGTFYSAARDPIFYAHHANIDRMWSI
WKTLGGKDFTDKEWLDSSFVFYDENADLVRAKVRDCCDSKNLGYVYQDVDIPWINCKPYRPSERAPVSYDHIPYTKNVFPGNLDKVIKVLVPR
PKKSRSKKEKEEEEEILVIDGIEVKTDEFVKFDVLINCEDDGMSATADQLEFAGSFVNVPHNHDHGKNMKTRLRLGISELLEDLTADNDEKVLVT
LVPKTNGSGISIQEIKIEYEVR
>Lactuca_1 sativa_Lsat_1_v5_gn_5_119940

MASLPTPTVTAAGATTKTYSSSFTTTSPVISSWPLFSKKCALNKPLKHKISCNAGSSENSLNNLDRRNVLLGLGGLAGAVNLTSVPSVGAAPISA
PDISKCGTNPLSGFRPGESTPTGGDCCPPDSPQIMDFKFPKNEAFRVRPAAHLLSPKYIAKFNEAIKRMKELPETDPRNFLQQAHIHCAYCNGA
YTQSSSGFPDIEIQIHNSWLFFPFHRWYLYFYERILGSLIDDPTFALPFWNWDTPAGMTIPKYFNDPKNAVFDPKRNQGHLQGVVDLGYNGKD
SDTTDIEKVKNNLAIMYRQMVTNATDPTAFFGGEYRAGIEPISGGGSVEQSPHTPVHRWVGDPRELNGENLGNFYSAGRDTLFYCHHSNVDR
MWSLWKMQGGKHKDITDPDWLNTSFVFYDENKNLVRVYVKDCLYTNQLGYDYQRVDVPWLKSKPVPRAPRSGVARKSIGKVKQAKEVSFP
VKLDKTVKVLVARPKKSRSKKEKEDQEELLIVQGITYDSEKYVKFDVYVNDEDDDASAPDQTEFAGSFAQLPHKHKGKTMSKTNFRAGLTELL
EDLEADDDDNVLVTIVPRSGSEDITIDNIKIIYA
>Aphelandra_1 squarrosa_TRINITY_DN15617_c1_g1_i4
MASLHAPPSTTASAAPSSQFRRLPLFPKPSSLITHARRSHRLRLSASSPSQNDAAGKVDR
RNMLLGLGGLYGATNLISTPNQSALANPIQAPEMDKCGLSSYTTNDQQKVDINCCPPLSP
NIVDYKLPNFPKCKIRPSAHRVSADYIFKLNTAIDRMKRLPEDDPRNFMQQANIHCAYCN
GGYKQTGLSVDLQVHNSWLFFPFHRWYLYFYERILGKLIDDPNFALPFWNWDNPKGMTIP
PMFNDPKAAIYNTKRNQDRLTSVVDLGMTGNTDPLQLVTNNLTIMYTEMIRGNSTAYDFM
GKPYRQGTTEEEAGPGASERGSHTAIHVFVGDPREPSGEDLGKPYRQGTTEEEAGPGASE
RGSHTAIHVFVGDPREPSGEDLGNFYSAGRDTLFYCHHANVDRMWSVWQNLPSDKVPEKR
ILDSDFLNAQFLFYDENRNLVRVTVKDCLDNVKMGYDVERVDLPWLDYRPPPQTALARVS
RVGGTTARKAQHVFPLNLQKVERFQVSKTKKGQARELLVLENIVVDTSKFLKFDVFVNDE
DDNALELDKAAYAGTYAQVPHKSAKSTATATISLRLTDLYKDMDIEDDDTVVVTLVPRHH
GPGVTIGGIKIVEEPPQKSS
>Aphelandra_1 squarrosa_TRINITY_DN1230_c0_g1_i1
MASLGCPSGLLQVATNPQNRSPNFSAKPSTYFLTARRRRLNVSCNGGRSPDSGKVDRRNM
LLGMGGLYGAANLVSADPQAQAEPIEAPEFTKCGAAHDTTTGNPLDVNCCPPVSGAGFKD
YVLPPVVKLKMRPAAHRVTKEQLAKYELAMKRMRDLSVKDPNDPLGFTQQANIHCAYCNG
AHDQPGFDNLDIQVHNCWLFFPFHRWYLYFYERILGKLIDDPTFALPFWNWDHPKGMTFP
KIYDREGSPLFDVNRDQAHRGPALIDLGFTGATEDLQIISNNLGQMHTEMIKSVNSTLDF
MGKVYYVRGDQPNNNGGGGTSENGSHVAAHAWVGNPLNEPYHEDMGNFYSAGRDPVFYCHH
SNVDRMWAIWKTLDTDVPKQLTDPNFLNATFLFYNENRELVRVKVADCLDQRKMGYDFEK
VDTPWKNFRPPKRAERVDIKKIASGAKKADAVFPLNLKKISRVLVPKSAKGKADEVLVLE
NIVVDSTKFTKFDVYINDEDDSPKELDKAEYAGTFAQVPHKTKDKKGNATINLRLTELYE
DIDVGDDDTIVVTLIPRSGGEHVTIGGIKVIPSPPRSGAAS
>Aphelandra_1 squarrosa_TRINITY_DN15617_c0_g1_i1
MASLPNLPSLSHPSAGGHRSAAAVPHPTFAKPSHFITHAKRNARLQVTCSSGGGQSPDAA
ADGKVDRRNMLLGLGGLYGAAGLVSGPGASANPVQAPELDKCGVATNLNTGKQLDINCCP
PLSQNVIDYKLPPVFHMKTRLAAHRVSPEYMYKFNTAIDRMKRLPKDDPRNFMQQANIHC
AYCNGAYDQPGQGTLDLQVHNSWLFFPFHRWYLYFYERILGKLINDPTFTLPFWNWDNPK
GMTIPPMFVDPKAAIYDSKRNQANLPPAVVDLGMTGNKDPLQVVANNLTIMYTEMIRGNS
DAYDFMGQPYREGTPVNPGPGASERGSHTAVHVWVGDPREPSGEDLGNFYSAGRDPLFYC
HHANVDRMWSLWQYFLPSSKVPDKKITDPDFLNAAFVFYDENAQLVRVRAKDCLDNIRMG
YDFERIDLPWLDYRPPPQTAAARVKRTGTGAAKAETVFPLVLDRIVRVLVPKTKKGKADE
ILVLEDIVVDTTKFLKFDVFVNDEDDNAAELDKAAYAGTYAQVPHKTAKGTATTSIRLKL
TELYDDMDVADDDTVLVTLVPRHHGPGVTIGGIKIIENPPKAAATSS
>Aphelandra_1 squarrosa_TRINITY_DN89858_c0_g1_i2
MASTLLPCSITSPRFSSCPILVGKPPHFSPTPTRRFALSCTAGGSGGHRKDSPQFDRREM
LLGLGAAGGLYSSVAASSAEPIPQQVLDKCTTATDLATGSPVDFNCCPPGSSSGPLPYVL
PNPPGKPLFRPALHLASPEYIKKLEQAIQAAKDLSTKDPNDPRGFSQQANVHCAFCNLAY
PQSPGSDLKLQIHFSWYFFPWHRWYLYFYERILGKLIGDPTFALPFWNWDNPKGMTYPDS
VKKSDSPLYNPRRNTSHYPLTPDLQYNGTTSTKSADQIKSSNLAEMYTEMISGAKTLENF
YGGKYVVGTDNVNRQGTVEGGSHTALHRWVGEDNREGFDMGNFATAGKEVLFFAHHANID
RLWSVWRNYRDNLSKPSYFNEPDWLDAKFVFYDENANLVTVRNGDSLDYEKMGFTWQDVP
NPWLDAKPRALKKKSTFAKSAKAPKSTDSTIFPVKLDKVVRVLVDRPKKRRSAKEKDKEE
EELLIEGIDVEGSEFVRFEVFVNDEDVKPEELIGKAEYAGCYSQVPLTKPGRIKGVLKLG
LSELLEELDIEDDDKILVSIVPRGGGKFITIGGIKIVYDDV
>Aphelandra_1 squarrosa_ TRINITY_DN4762_c0_g1_i3
MASSIPSSTTIPSGAAASVAAFPSSQLLARSNRFPTPRKTARRHSISCKSADNTDEKSPG
RFDRRDVLIGLGGLYGATALSAASPFAFAAPISAPDVTKCGPADLPPGATPTNCCPPPTA
SIVDFKFPPPPATLRLRPAAHLADQAYVAKFNRAVELMRALPDDDPRSFKQQANVHCAYC
DGAYDQVGFPDLELQVHNSWLFFPFHRYYLYFFERILGKLIDDPNFAMPFWNWDSPGGMR
IPAMYTSPSSALFDQLRDRGHQPPTVIDLNFGGRDTGGVTPQQQMQRNLTIMYRQMVSNS
RTSRLFFGSPYRRGENPNPGPGSIENTPHGPVHVWTGDSTQPNLENMGNFYSAGRDPIFF
AHHSNIDRLWNVWKTLGGRRQDISDSDYLDASLIFYDENAQMVRVKVRDCLDQAKLGYAY
QEVENPWLQSRPTPRISSAVRRLKNLVKARATDTHHDPRHVFPAKLDKVLKVMVKRPKKK
RSRKEKEELEEILVIQGIEVDRDAYAKFDVFINDEDDQVSTPENTEFAGSFVNVPHKHKH
VRKIKTQLRLSITDILEDLDAEDDDHVLVTLVPSNAGESITVHGIKIELDD
>Aphelandra_1 squarrosa_TRINITY_DN15617_c1_g1_i1
MASLHAPPSTTASAAPSSQFRRLPLFPKPSSLITHARRSHRLRLSASSPSQNDAAGKVDR
RNMLLGLGGLYGATNLISTPNQSALANPIQAPEMDKCGLSSYTTNDQQKVDINCCPPLSP
NIVDYKLPNFPKCKIRPSAHRVSAEYIFKFNTAIDRMKRLSENDPRNFMQQANIHCAYCN
GGYKQTGLSVDLQVHNSWLFFPFHRWYLYFYERILGKLIDDPNFALPFWNWDNPKGMTIP
PMFNDPKAAIYNTKRNQARLTSVVDLGMTGNTDPLQLVTNNLTIMHTEMIRGNSTAYDFM
GKPYRQGTTEEEAGPGASERGSHTAIHVFVGDPREPSGEDLGNFYSAGRDTLFYCHHANV
DRMWSVWQNLPSDKVPEKRILDSDFLNAQFLFYDENSNLVRVTVKDCLDNVKMGYDVERV
DLPWLDYRPPPQTALAKIKRVGGTTARKAEHVFPLNLQKVERFQVAKTKKGQARELLVLE

NIVVDTSKFLKFDVFVNDEDDNALELDKAAYAGTYAQVPHKSAKSTATATISLRLTDLYK
DMDIEDDDTVVVTLVPRHQGPGVTIGGIKIVEEPPQKSS
>Aphelandra_1 squarrosa_TRINITY_DN15617_c1_g1_i3
MASHHHVPSAPPSSQFLRLPLFPKPSSLITLHARRCHGLRLHASTPSQNEAGGGRRNMLL
GLGGLYGATNLISNPNQAALANPIQAPEVDKCGVATNLNTGEKLDINCCPPLSQNIVDYR
LPHFRRLKIRPSAHRVSPEYVFKFNTAIDRMKRLPEDDPRNFMQQANIHCAYCNGAYKQA
GLSVDLQVHNSWLFFPFHRWYLYFYERILGKLIDDPNFALPFWNWDNPKGMTIPPMFNDP
RAAIYNTKRNQDRLRSVVDLGMTGNTDPLQLVTNNLTIMHTEMIRGNSSAYDFMGKPYRQ
GSTTEETGPGASERGSHTAIHVFVGDPREPSGEDMGNFYSAGRDAIFYGHHANVDRMWTV
WQNMPIDKVPEKDISDPDFLDAQFLFYDEDSNFVRVKVADSLSNVKMGYGYERVDLPWLD
YRPPPQTALARVSRVGGTTARKAQHVFPLNLQKVERFQVSKTKKGQARELLVLENIVVDT
SKFLKFDVFVNDEDDNALELDKAAYAGTYAQVPHKSAKSTATATISLRLTDLYKDMDIED
DDTVVVTLVPRHHGPGVTIGGIKIVEEPPPPSR
>Lamium_1 galeobdolon_Contig_3120
MVTLALSWSTPTTTSRPTFSTKPLHISAQIHSRKISCHGGETPENIQQKYDRRNLLLGMG
CLYGAVISESPAVATPITSPDLSKCTKGTNLNTQSPLDINCCPPPSPKIIDYKLPPMTKM
RVRPAAHLAGKEYYAKYQKAIALMKALPAEDPRSFMQQANVHCAYCNLTYEQTGDPKLKL
QIHNCWHFYPWHRWYLYFYERILGKLIDDPTFGLPYWNWDNPDGLILPEVFMDKGSPLYN
ARRNPDHLPPAQADLGYSNSSPTDPKKIVKNNLTIMYNEMVRNGKKLPDFYGAKYVVGTA
TDPGPGSVERGSHMSLHSWVGENTPTGEDMGNFYSTGRDVIFYSHHANVDRLWTVWRNLR
GSKPKHFTDSDWLNADFAFYDENANLVKVRVSDTLDNEKMGYVYQEMDLPWLNKRPKARV
QKSKVAAGSPETAFPVKLDKVVKVLVKRPKKSRSKKEKEKEEELLVIEGIQVDTGKFVKF
DVFVNDEDDKPDELDKAEYAGCYSQVPHKNSTKVTTKIRLSLDEVMEDLDVEDDDEILVS
LVPKAGGQDITIGGIKIIYDSD
>Lamium_1 galeobdolon_Contig_1707
MASPFISQPLTTLSAAATSTTATTFLVNPRQFPKTKKSSHTYKISCKSDQESSQPNKFDR
RDILLGLGGLYGATTLATSPFAFAAPIGAPDVTKCGPADLPSGAAPTNCCPPPTSKIVDF
KFPSPSSKLRLRPAAQLVDEAYLAKFNKAVELMRALPDDDPRSFKQQANVHCAYCDGAYD
QVGFPNLEYQVHSSWLFFPFHRYYLYFFERILGKLIDDPTFAMPYWNWDGPGGMSLPAIY
ANAKSPLYDPLRDRLHQPPTVLDLNYSGSDPSISPDQQTKRNLTVMYRQMVANSKTPRLF
FGSPYRRGEDPDPGSGSIENIPHGPIHVWTGDRTQPNTENMGNFYSAARDPIFFAHHSNI
DRMWSLWKSLGGRRQDITDRDFLDAAFIFYDENGEMVRVKVRDCLDNTKLGYDYQKVETP
WLQSRPEPRVSSILRKLKKLGRAKAADAGSPREVLPAKLDRVLKVMVKRPKKRRSKKEKD
ELEEILVIKGIELERDVYAKFDVYINDEDDVVTTEDNTEFAGSFVNVPHKHKHGKKIKTQ
LRLSITEIMEDLDAEDDDHVLVTLVPTNAGDSIKIHDVKIVLDD
>Lamium_1 galeobdolon_Contig_1193
MASLPLCSTYSTQLSSRPSLFGKPPPLKTRTHHQPGVSCNSQINEPKVDRRNLLMGMGGM
YGAANLESSALAIPISGPDLSKCSNSQVQSPEGVTLDVNCCPPLSTEIHEYKLPPVRTMR
YRPAAHLVTKEYVDKYEQAVALMKALPKDDPRSFYQQAMVHCAYCNLGYEQTGDPNKKLQ
IHFCWHFFPWHRWYLYFYERILGKLIGDPTFGLPFWNWDNSTPASPPYIPEMYVKETSPL
YDWRRNSCHLPLALADLKYSKSVSTPPHKILSSNLAEMYNEMVRTKTLVGFYGEPYCVGS
NLDPGAGSVEXGSHTAMHLWVGENTAGGEDMGNFYSAGRDSIFFAHHANIDRLWTVWRHL
NSPFRPTKFSSTDWLDANYLFYDENKQLVRVYVRDTLDQGKMGYGYQVVDNPWESNRPMS
YLKKSRVSAMTKAVGKTNFPVKLDKVVKLLVDRPNRKRSNEEKEKAEELLVIEGIEADMS
KAVRFEVYVNDVDDKWKELIDKAEYVGCYGQVPHRNSSTKMKSMIRLGLTEVLEDLEIED
DDKILVSLVPKAMGEEITIGGVKIVRVDYAAEN
>Lamium_1 galeobdolon_contig_11686
MASLPLLCGSTAPPHRRSSLPNHSHFLTHANRNLRIHVSSSATQNDSSSSSQSVDSSPAK
VDRRNMLLGLGGLYGAGNLMSTPAALADPVQAPDLSKCGVATDVVKNDTIDVSCCLTTED
RIVDYKLPPVFQVKMRPSAHRVSPEYIYKYNMAIDRMKRLPADDPRSFMQQANIHCAYCN
TVYKQGGTDVDLQVHNSWLFFPFHRWYLYFYERILGKLINDPTFALPFWNWDNPKGMTVP
PMFMDPRSAIYDSKRNPRVQNTPPAIVNLDMGNNITDPLQLVTNNLTIMHTEMVRGNADV
YGFMGNDISQGKGFAGAGSVERGSHTAVHVWVGDPRQPRGEDLGNFYSAGRDPLFYCHHG
NVDRMWTIWQYFLPSISFDKRITQTDFLDSSFLFYDENAQLVRVYVKDALDNLRMGFEYE
RIDLPWLDYRPPQQSIRAKINRKSTVQAPAAATLFPLKLEQVVRFQVDKTKKGKADESLL
LENITVDTSKFLKFDVFVNDEDDNASELDKASYVGTYAQVPHNSKDHFATTSIKLNLKEV
YKDMDVEDDDTIVVTLVPRHQGSGVTIGGIKIVENPPNKKA
>Lamium_1 galeobdolon_ Contig_14578
MASLQSSCLVLSNKPFSAKPSPFVTHARRSRISCNGNGSLEGRVDRRNMLLGLGGLYGTT
RLIPEPKGASADPIEPPDFSKCGKPSTGGETLDVNCCPPVSEVPPVDYKIPAFEKLQVRP
AAHRMSKEHVAKFEEAIRLMKKLSVDDPTDPRGFTQQANIHCAYCNGAHDQRGTTTDLQV
HNSWLFFPFHRWYLYFFERILGKLIQDPTFTLPFWNWDNPPGMHMPAMFDNTRSSLYDCN
RDQGHRPPAVAELSPTGATDEEVIIKNNLFEMHNEFYNAHSALDFMGQAYRAGDVAPDDP
KDGAGSLERGCHNALHWWVGDPRNKHHEDMGNFYSSGRDTLFYCHHVNVDRMWTIWRSFD
ETNVVKDIPDRDYLDSAFLFYDENKQLVSVKSRDCLNEARMGYEYEKMPLPWKKVTPRKT
AGKADINKMGAQTPDKLFPLTLDKITRVLVPKSAKGKTDEILVLDNIVTDPTKFVKFDVF
INDEEDNPQELDKAEYLGTFSQLPHRTSRSKHSNGFINLKLTRVYDHLDLADDDDTVLVT
LIPRSSGQHVTIGGIKIIPAPP
>Lamium_1 galeobdolon_Contig_22550
MASLQSSCALLSTTPHHRNPASSKPFSAKPSTHARRSNAFHVSCNGGADQKPSTEGRVDR
RNMLLGLGGLYGAANLPKTASANPIQAPEFNTCGAAYDVISGDLLDINCCPPVSDTIVDF
KLPPVHKMRVRPPAHKVTKEYLAKFERAISLMKKLDVDDPSDPRGFMQQANIHCAYCNGA

HDQVGYPKLDIQVHNSWLFFPFHRWYLYFYERILGKLIDDPTFTLPFWNWDNPKGMQIPK
IFNNEMSPLYNSNRNQANRPPAIIDLGLTGETDDLQKVVNNLSLMYSENIRSVNSTLDFM
GHAYRAGDQPGGGDGMGSSERGSHVAAHVWVGDPRNKYSEDMGNFYSAGRDILFYSHHAN
VDRMWTIWRTLPTDVPKEIKDPDYLNASFLFYDENKQLVRVKVGDCLDHKRMAYEYERVD
IPWLNYKPSKKAGKARIQKIAKGAQTVDKLFPLTLKNITRVLVPKPTKGKADEVLVLENI
VTDSTKFIKFDVYINDEDDNPKEMDKAEYAGTFAQVPHKSKSKQSNGSINLKLTDIYDDI
DVDDDDTVVVTLIPRSNGDDVTIGGIKIIPAPPKSA
>Populus_1 trichocarpa_Pt_PPO13
MEAKKLGLVLGIITIVAAFTRILHILEAPEVQHALLGEFNFIIPGMLKLASSPGKDQQEK
PLVILPNLTSCHESMGRSDLPVYCCPPMNQSNVAIIDFQFPDPSLPLRVRRPTHLLDDNY
ISKYKKAITIMKSLPDTDPRSYTRQANLHCLFCTGAYNQQGSNSPLNIHRSWLFFPWHRM
LIYFHERILGSLIGDDTFALPFWPWDIPEGMVIPEMYMKAPFFHEARDFSHFPPSVVDLD
YSCTTPSSEDYRCFESGLGPEDQVHTNLVMMYNQMVAGAKKMELFMGCPYKAGEGGSCNG
PGTIELAPHNTVHKWVGSNLNPGSREDMGVFYSAARDPIFYPHHANIDRLWDVWRTLHGN
KVITDPDWLDSSFFFYDEKLQLNRIKIRDVLSITKLQYAYEKVDFTWLNNRPKPSVPLEV
ARNILRMRQNAKQQQLQRNGILSSNFSPHGRFLDATLRTRVNRPKVRRTKKEKEEEEEIL
VVHGIDIPEERYVKFDVYVNVVNETIMNPRFREFAGTFVHIDPGVTRAARESNIEVFRKK
TDLKLGISELLEDLEAEGDENIWVTLLPRSEGCINTTVDGLRIEYIR
>Lt_PPO_1
MASLSSQSKLLATPYSFPYHTKPSRVSLRRVSCKASNDNKDKPNDQEKTFSIDRRNMLIG
LGGLYGASNVFPSNQSTLAAPIQPPVLPDSCHPPEDLAEGVNVLCCPPDVKDPIDFQMPS
NPSRLRIRPAAHLADPTYIEKYKKALAAMKALPQNDPRSFYQQANIHCAYCNGAYDQVGF
PDVNIQVHHSWLFLPFHRWYLYFYERILGSLIDDPTFAIPFWNWDAPKGMHMPHMFIDPN
SPLYDAKRNPAHFPDTIVDLDFSSGEAPSHNPRQIGNNLSIMYKQVVRAKKARLFHGRPL
QAGSFPDESGDGSLEGTPHGNIHLWSGDPRQSNFENMGNFYSAGRDPLFYAHHANVDRMW
YIWKNSLGRKDYKKKDWLNAGFLLFDENAQPVRVYVRDALDERKLGYAYQEVDIPWINSK
PKPRKANPWPNRLRSKATTTTKLINKFPLTLDSTVSFEVKRPKKSRSKSEKEDEEEVLVI
EKIKHEPQFPLKFDVYINDEDEDPSAADQTEFAGSFVNVPHFHRHGDKKDKRQTTNLSIG
ISEVLDELDVDGDDSIVVTLVPRVGSGQITIGGAKIEFVRDEED
>Am_AS1_1
MFKNPNIRYHKLSSKSNDNDQESSHRCKHILLFIITLFLLIVGLYIANSLAYARFASTST
GPIAAPDVTKCGQPDLPPGTAPINCCPPIPAKIIDFELPPPSTTMRVRRAAHLVDDAYIA
KFKKAVELMRALPEDDPRSFKQQANVHCAYCAGAYNQAGFTNLKLQIHRSWLFFPFHRYY
IYFFERILGKLINDTTFALPFWNYDSPGGMTIPSMFIDTNSSLYDSLRDSNHQPPTIVDL
NYAFSDSDNTTTPEEQMIINLKIVYRQMVSSAKTPQLFFGRPYRRGDQEFPGVGSIELVP
HGMIHLWTGSENTPYGENMGAFYSTARDPIFFAHHSNVDRMWSIWKTLGGPRRTDLTDPD
FLDASFVFYDENAEMVRVKVRDCLDEKKLGYVYQDVEIPWLNTRPTPKVSPSLLKKFHRT
NTANPRQVFPAILDRVLKVIVTRPKKTRSRKEKDELEEILVIEGIELERDHGHVKFDVYI
NADEDDLAVISPENAEFAGSFVSLWHKPIKGKRTKTQLLTLSICDILEDLDADEDDYVLV
TLVPRNAGDAIKIHNVKIELDG

Sequences used for BX8 phylogenetic analysis

>Lamium montanum_Cluster-1060.0
MGSISTQHKPHAVCIPYPAQGHVNPMLKLAKLLHHNGFHITFVNTDYNHRRLLNSGGSSALHGLPDFRFATIPDGLPFSDADATQDIPSLCVST
TTTCLEPFSDLISSLNSAVPDMPPVSCIVSDGVMSFTLKAAERFGLPEVLFWTTSACGLLGYTHYHNLVEKGYIPLQDMSQATNGYLETKLNWV
DGMKDIRLRDIPSFIRTTDPKDTMLNFLLQETAALPRAKALILNTFDALEHDVISALSASLPRVYTVGPLQLMMDHIHDKRLDSFTSTLWKEDPGC
LEWLDSKPPGSVVYVNFGSITVMTAQQLTEFAWGLANTNKQFLWVIRPDIVAGESAMVPPEFVEETKDRCMMVSWSPQEKVLKHPAIGGFLT
HSGWNSTLESIVGGVPLICWPFFAEQQTNCRFSCVEWEMGMEIDNNVKRDEVESLVRELMDGEKGKKMKEKAMEWKEKAEAATVPGGSSY
LNLEKLIKEVLLH
>Lamium montanum_Cluster-13208.8564
MKPHAVFIPYPAQGHIAPGLKLAKLLHYRGFFITFVNTEFNHNRLLRARGPDSVKGLPDFQFRTIPDGLPPPLNADATQDIPLLSQSISQNCLAPF
VDLINNLNNSHDCPNVTCIVSDGVMSFTLDAAEKLDIPAALFYTTSACGFMAYLHYSELVSRGLVPFKDESCFTNGYLDTEIDWIQGMKGIRLRD
IPTFIRTTDPDDIMVKYNIVQHENASRAKAIIINTFDDLETEVLEAIRARFEKVYTIGPLQLLEKEIYQKKLTSIGSNLWKEDMECLKWLDEKERNSV
LYINFGSITPLSPSQMVEFAWGLAKSNLNFLWIIRPDLMNGKGSVLPEGFLDETEGRGLMVGWCPQEKVLAHAAVGGFLTHCGWNSTIETVSE
GVPMLCWPFFAEQQTNCRYACTKWGIGVEIEGDVSRGKVEKMVKLMMEGEEMREKALEWREKASLAAMPGGSSYMNLDLLINNLI
>Lamium montanum_Cluster-11699.0
MAHVLIFPLPLQGPVNCMLKLAEILCLENIKVTFLNTQHIQQRLFNTQIYFKKYPNFCFETVPDGLPDENPRTGDQIGKLLESMEDTAVPVFRDM
VSGDVYGPTSENPVTCLIADGIFTFAVDIAKEIGVPLLYFDTISPCGLWTYLCLPKLIQAGEFPFKDDNLDEIVTNVPGMDGIIR
RRDLPGFCRTNDINDRIIQLILKEEKHIPLAQGLIFNTFQELEEPILSQMLNICPNIYAIGPLHAHLKIRLGLEKGSEFKSTISNSIWREDESCIYWLD
QQPSKSVIYVSIGSLAVITRDQLFEIWHGLVNSGSRFLWVQRPGSIAGLDSNPDQVNLELLQGTKERGCVVSWVPQEEVLA
HPAIGGFLTHSGWNSTLESIVEGKPMICWPYFVDQQVNSRYVGEVWKLGLDMKDTCDRVIVEKMIREVMESRKGEFLKRAEEMAKLATSSVK
GEGSSFVDLDRLIENIKMMKISRK
>Lamium montanum_Cluster-1251.0
MEKSKRCSRIVLFPLPFQGHINPMIQLARILHSKGFTISIVHTRFNYPNTSQYPLFTFHLIPDGLSDSGTSSSTISDPILIIKTLITSCMEPFHRCLAQ
LCSDDSNDRVVCLVTDGMWYSTQASADKLKLPRIVLRTSSICSFLAFAAFPLLREKGYISNRDSEKEAAILELPPLKVKDIPTIETHNPEDLYEVIG
NMVEETKKCSALIFNTFKELEEPELSKLIDDQFLMPIFSIGPFHKYFSAASSLLTQDRSSISWLDKRAPNSVLYVSFGSVALMDEKSLVEVAWG
LANSEQPFLWVVRPGLVHGSEWLEMLPNGFLSSVSERSYIVKWAPQQEVLSHPSVGGFWTHSGWNSTVESICEGVPMICSPFFGDQMVNSR
YVSDVWKIGMKLEKGLERGEIEDTIRRLMSNGEGQGIRQSVVCLKDKVHVCLNEGGSSYEALDALINFISSF

>Lamium galeobdolon_Contig _5376
MKPHAVFIPYPAQGHIAPGLKLAKLLHYRGFFITFVNTEFNHNRLLRARGPDSVKGLPDFQFRTIPDGLPPPLNADATQDIPLLSQSISQNCLAPF
VDLINNLNNSHDCPNVTCIVSDGVMSFTLDAAEKLDIPAALFYTTSACGFMAYLHYSELVSRGLVPFKDESCFTNGYLDTEIDWIPGMKGIRLRD
IPTFIRTTDPDDIMVKYNIVQHENASRAKAIIINTYDDLETEVLEAIRARFKKVYTIGPLQLLEKEIYQKKLTSIGSNLWKEDMECLKWLDEKERNSV
LYINFGSITPLSPSQMVEFAWGLAKSNLNFLWIIRPDLMNGKGSVLPEGFLDETEGRGLMVGWCPQEKVLAHAAVGGFLTHCGWNSTIETVSE
GVPMLCWPFFAEQQTNCRYACTKWGIGVEIEGDVSRGKVEKMVKLMMEGEEMREKALEWREKASLAAMPGGSSYKNLDLLINNLI
>Lamium galeobdolon_Bx8
MSSFSKAESSEQKPHAVFIPAPGQGHINPMLMLAKLLHYNGFHVTFVNTEFIHRRLLKSCGPSALRGLPGFRFATIPDGLPESSTRDVAALCES
FTTTCLEPFCDLLLEIKSSGGGVPPVSCIVADGEMSFTLKAAERFGLPEVLFWTTSACGLLGYTQYRRLVEKGYVPLQDMSQGYLDTTVDWVP
GIKNIQLRDFPAFIRTTDPKDFMLNFKTQEVEAIPRAKALILNTFDALEQDVLNALSDMFPTIYTVGPLQLMMNHVHDQTLDSITSSLWKEDFQCI
EWLDSKEPGSVVYVNFGSITVVTAQQLTEFAWGLANSNKQFLWVIRPDIVAGESALVPPEFVEKTKNRSLLISWCPQEQVLKHPAIGGFLTHSG
WNSTLESVINGVPMICWPFFAEQQTNCRFSCVEWEMGMEIDNDVKRDEVEFLVRELMDGEKGKKMKEKAMEWKNKAEAATVPGGSSYLNF
EKLIKELLP
>Lamium galeobdolon_Contig_32831
MVNSVETRKPHAVLVPYPTQGHVTPMMRLGKLLHARGFHVTFVNTEFNHSRLIRSKGPESVSGLPNFRFETIPDGMPPSDDQDATQEVPLLC
HYTRKTCLEPFKKLLGRLNTTPYVPKVSCVVADGVMSFGMKAAQQMGIPDIRFWTASVCSMIGYLHYRELLRRGITPFKNEDFLRDGTLNTCV
DWVSGMGPDVRLKDLPSFIRTTDPNDIMFEFLGEEAQSCLNASSIIFNTFQEFEKEAIHTLISKFNYSNIYTIGPLHLLAEHMPKSEANSFNSSLW
KPDSKVFEWLDEKAPGSVVYVNYGSITTMTREHLQEFAWGLAKSSQPFLWIVRPDVVKVGGESGPLNEEFFEEVKGRGLLVSWCAQDRVLE
HESVGVFLTHCGWNSLIESVSCGVPVICWPFFADQLTNCYYSCGEWGIGMEIDSDVKREEVAELVKEMMEGERGEKMRLKAKEWQRIAEEAT
DVGGGSYAHFNKLIKEGLHFDG
>Lamium galeobdolon_Contig_2924
MESPVKTQKPHAICTPFPAQGHINPMFKLAIILHHKGFQITFVLTDYNHKRLLQSLGTAALNPPPDFHFATIPDGLPPPESATSTQDIRSLCVSTA
NNCLAPLNQLIDNLNRHSPPVSCVITDTIMSFALDAAERVGVPGVLLQTSTACSFMCNKHIKQLIEKGFVPLKDERCLTNGYLE
TPIDWIPGMIPLLLRHFSGIIRTTDPNDSFLSFVPTQLARSSKAAAIIMNTFDSLEHRVLSALYSICPPICTVGPLHLIVNQLPGNKWMSSSLWKED
SSCLKWLDSKPQNSVLYVNFGSIAVLNPRQLGEFGWGLAKSKKNFVWIVRPDLFEGENRAISPDFLEEIRDRGVVAGWCPQEEVLSHGAVGG
FLTHCGWNSILESLCNGVPMLCWPFFADQYLNCKYACDEWGVGVGMEIKSDVERDEVGVLMRELMDGERGNMLRKRAVEWKEKAAAAVAA
GSSCLNLDRLVKEVLCV
>Lamium_1 galeobdolon_Contig_20839
MNSNLEMTTHQEITAPPPLPPHILLAPLPLQGHVNPMLNLAEVLCLSDLHVTILLSDHTHSRLLHHAGIRSRFSHYPGFRIATISDGLPDDHPRSG
CHAIDIVMSLNQVGGSQFRHLMESTDALRDGGARRRVCCIIMDGFLSFTLPVIEEMGIPYIYFRTSSACSFWAYFCIQELIDAGEVPLKGKHEDK
LAYWGKKEMDLMVTSVPGMETFLRRRDVPGLCRVEDIHDPGFQTVKTQTKRTAKALGLIINTFEDLEGPILDQIRNHIPNVYSVGPLHAHLKLSR
EIRGIESSHFSGSFWEEDDSCIAWLDSQPNNSVIYVSFGSITILTRDELLEFWYGLVNSGKKFLWVIRPDSIVDENKNESLVELEEGTKSRGLMV
GWVPQHEVLAHRAIGAFLTHSGWNSTLESIVIGLPMLCWPYMADQMINSRFVSEVWRIGLDMKDTCDRILIEKMVRDVMEVRRDEFLERAHSL
AVLGNNAISEGGSSYLNLNRLIQFLKSTAS
>Lamium maculatum_Cluster-9204.4039
MGSFSNSKVAESSNHKPHAVCIPATAQGHINPMLKIAKLLHYNGFHVTFVNTEFNHRRLLKSCGPSALGTLLDFRFATIPDGLPYSDADSTQEA
AAICESTSTNCLAPFCDLLLEINSSGGDVPPVSCIVSDGVMSFTLKAAERFGLPEVLFWTTSACGLLGYTQYHKLVDKGYIPLQDMSQVTNGYL
DTIVDWIPVIKNIRLRDLPTFIRTTDPKDIMLNFTIQEVEAVPRAKALILNTFDALEQDVLNALSDMFPTIYTVGPLQLMMNHVHDQTLDSITSSLWK
EDFQCIEWLDSKEPGSVVYVNFGSITVVTAQQLTEFAWGLANSNKQFLWVIRPDIVAGESAMVPPEFVEETKDRSLLINWCPQEQVLGHPAIG
GFLTHSGWNSTLESITNGVPMICWPFFAEQQTNCCFSCVEWEIGMEIDNNVKRDEVELLVRELMDGEKGKKMKEKAMEWKEKAKAAAVPGG
SSYLNLEKLIKELLQ
>Lamium maculatum_Cluster-9962.0
MKQPHAVFIPYPAQGHIAPILKLAKLLHVRGFFITFVNTEFNHNRLVRARGPESVAGLDGFQFRAIPDGLPYSDKDATQDIPQLCDSLTKNGLPP
FLGLIKSLNESPDCPDVSCIVSDGAMSFTLDAAEQLDIPEVVFFTTSACGFMGYCQYGELVNRGLFPLQDESQISNGHFDTELDWVPGMKHIRL
RDFPSFIRTTDPDDIMVNYNILQTKNALRAKAVIINTFDELEKDVLDAIKQKFEHVATIGPLQLLEHEVKDVEVKSIGSSLWKQDDACLAWLDQRA
PNSVLYVNFGSITVLSHQQLLEFAWGLANSKQYVLWIIRPDLVSGDSAVLPEEYLKEVEDRLCMVGWCSQEKVLSHPSVGGFLTHCGWNSTIE
GVSEGKPMICWPFFAEQQTNCRYACTDWEIGLEIEGEVRRETVVELVKILMEGEKGKEMRKKALEWKEKAHFAAKSGGSSYHNLEVLINQILL
KN
>Lamium maculatum_Cluster-9204.9749
MESPGKPHAICTPFPAQGHINPMFKLAIILHHKGFHITFVLTDYNHKRLLQSLGAAALNPPPDFHFATIPDGLPPPQSANSTQDIRSLCLSTSNYC
LAPLNQLIHNLNRHSPPVSCVITDTIMSFALDAAERIGVPGVLLHTSAAYSFMCNKHIKHLIEKGFVPLKDESCLTNGYLETPINWIPGVIPLRLRH
FSSVIRTTDPNDSLLSFLPTQTARSSKAAAIIMNTFDSLEHRVLSALSSICPPICTVGPLPRNKWMSSSLWKEDSSCLKWLDSKPQNSVLYVNFG
SIAVLNPRQLSEFAWGLAKSMNFFVWIVRPDLFEGENRAFSPDFLEEIRDRGVVTGWCPQEEVLSHGAVGGFLTHCGWNSILESLCNGVPML
CWPFFADQYLNCKYACDEWGVGMEIKSDVERGEVGVLVRELMDGERGNMLRRRAMEWKEKAAAAVAGGGGGGGGGSSCL
>Lamium orvala_Cluster-2218.7465
MGSETQYKPHAVCVPFPAQGHINPMLKLAKLLHHNGFHVTFVNTHFNHRRLLKSCGPSAVQSLPDFRFATIPDGLPESDADATQDIPALCEST
TTTCLEPFSDLLLEINSSGGDVPPVSCIVSDGVMSFTLKAAERFGLPEVLFWTTSACGFLGYTQYLKLVEEGYTPLQDMSQVTNGYLETIVDWI
PEIKNIRLRDLPTFIRTTDPKDIMLNFAIQEVEAVPRAKALILNTFDALEQDVLNALSDMFPTIHTVGPLQLVMNNHVHDQTLDSITSSLWKEDFQC
FEWLDSKEPGSVVYVNFGSITVVTAQQLTEFAWGLANSNKQFLWVIRPDIVAGESAMVPPEFVEETRDRSMMINWCPQELVLKHPAIGGFLTH
SGWNSTLESVVNGVPLICWPFFAEQQTNCRFSCVEWEVGMEIDNNVKRDEVELLVRELMDGEKAKKMKEKAMEWKGKAKAATVPGGSSYL
NFQKLIKELLP
>Lamium orvala_Cluster-7286.0
MKQQPHAVLIPYPAQGHIAPILKLAKLLHARGFFITFVNTEFNHNRLVRARGPESVAGLDGFQFKAIPDGLPYSDKDATQDIPQLCDSLTKNGLP
PFLGLIKSLNESPDCPDVSCIVSDGVMSFTLDAAEQLGIPEVVFFTTSACGFMGYCQYGELVNRGLFPLQDENQISNGHLDTELDWVPGMKHI
RLRDFPSFIRTTDPDDIMVNYNILQTKNALRAKAVIINTFDELEKDVLDAIRQKFEHVATIGPLQLLEHEVKDAEVKSIGSSLWKQDDACLAWLDQ
RAPNSVLYVNFGSITVLSHQQLLEFAWGLANSNQYVLWIIRPDLVSGDSAVLPEEYLKEVKDRAFMVGWCSQEKVLSHPSVGGFLTHCGWNS
TIEGVSEGKPMICWPFFAEQQTNCRYACTDWEIGLEIEGEVRREKVAELVKILMEGEKGKEMRKKALEWKEKAHFAAKSGGSSYHNLEVLINQI
LLKD
>Lamium orvala_Cluster-2218.7728
MVVNSVETTKPHAVLVPYPAQGHVNPMMRLGKLLHGRGFHVTFVNTEFNHRRLIRSKGPESVSGLPDFRFETIPDGMPPSDDQDATQDVPLL
CHYTRKTCLEPFKKLLGRLNTTPDVPKVSCVVADGVMSFGMIAAQQMGIPDLRLWTASVSSMIGYLHYRQLLTRGITPFKNEDYLTDGTLNTR
CDWVSGMGPDVRLKDLPSFIRTTDPNDIMFEFLGEEAQSCLNASSIIFNTFQEFEKEAIDALISTFNYSNIYTIGPLHLLAEHMPKSEANSFNSSL

WKPDSKVFEWLDEKAPGSVVYVNYGSITTMTREHLQEFAWGLAKSSQPFLWVVRPDVVKDDDESGSLDEEFFEEVKDRGLLVSWCAQDRVL
AHESVGVFLTHCGWNSMMESVSCGVPVICWPFFADQQTNSYYSCGEWGIGMEIDRDVKRDEVAELVKEMMGGERGKKLRMKAKGWQMIA
EEATDVGGGSYDDFNKLIKEGLHFDG
>Lamium album_Cluster-2496.5279
MKPHAVLVPLPAQGHVNPMMRLAKLLHGRGFHVTFVNTEFNHRRLIRSKGPESVSGLPDFRFETIPDGMPAAASDDVQDATQDVPLLCHYTR
KTCLEPFKKLLGRLNATPDIPKVSCVVADGVTSFGMKAAQQMGIPDVQLWTASVSSMIGYLHYRELLRRGITPFKNEDFLTDGTLNTSCDWVS
GMGPDVRLKDLPSFIRTTDPDDIMFEFLGEEAQSCLNASSLILNTFQEFEKEAIDTLISTFNYSNIYTIGPLPLLAKHMPKSEAYSLNSSLWKPNSK
VFEWLDEKTPGSVVYVNYGSITTMTCEHLQEFAWGLARTNRPFLWIVRPDVVKDGGELGPLNEEFFEEVKDKGLLVSWCAQDRVLAHESVGV
FLTHCGWNSMTESVSCGVPVICWPFFADQQTNCYYSCGKWGIGMEIDSDVKRDEVAKLVNEMMGGGRGKKMRMKAKEWQRIAEEATNVG
GGSYVDFNKLIKEGLHFDG
>Salvia hispanica_XM_048121407.1
MGSIWKIESKPHVVCIPYPAQGHVNPMLKLAKLLHHSGFFVTFVNTEYNHRRLINSRGPSAVAGLPDFRFETIPDGLPPSDADATQDIPSLCVST
TTTCLEPFCDLIAKLNASGAGVPPVSCIVSDGVMSFTLKAAERLGLPEVLFWTTSACGLLAYVHYKDLVEKGYTPLQDMSQVTDGYLETRIDWI
PGMKNMRLRDMPSFIRTTDSEDTMLQFLIQEAAAIPRAKALILNTFDSLEHDVLCALSARFPSVYTVGPLQLMMNHIHDDTLKPFTSSLWKEEAE
CIEWLHTMAPQSVVYVNFGSITVMTADQMTEFAWGLANSKKPFLWIIRPDIVAGESAMLPAEFVAETKDRSMLVSWCPQEQVLSHPAIGGFLT
HAGWNSTLESVVGGVPVICWPFFAEQQTNCRYSCVEWGTGMEIDNNVKRDEVELLVREMMDGDKGKEMKRNALEWKRKAEEAAAPHGSS
FLNLHKFIKEELLH
>Sesamum indicum_XM_011094127.2
MGSLPRPESKPHAVCIPYPAQGHVNPMLKLAKLLHHHGGFHITFVNTEYNHRRLIKSGGPSAVEGLSDFRFHTIPDGLPPSDADTTQDVPSLCV
STTTTCLEPFCDLLSKLNSEAPPVSCIVSDGVMSFTLKAAERFGLPEVLFWTTSACGFLGYRQYRRLVEEGHTPLRDMSQVTNGHFETVIDWV
PGMKDIRLRDFPSFIRTTDPKDIMLNFLIQEAEAAPRAKALIINTFDALERDVLDALSASFPRIYTVGPLQLMMDHVQDERLGAMASSLWKEDLS
CMEWLDTKEPESVVYVNFGSITVMTADQMSEFAWGLANSKKPFLWVIRPDLVAGDKAMLPPEFVTETKDRSRLMSWCPQEQVLKHPAVGGF
LTHSGWNSTIESIVGGVPVICWPFFAEQQTNCRFSCVEWGMGMEIDNNVKRGEVEALVKELMDGEKGKKMKEKALEWKRKAEEAAAPGGSS
YLNLENLINELLLQ
>Scoparia dulcis_MG011426.1
MGSIAGNEQKPHLVFVPYPAQGHITPMIKLAKLLHQKGFFITFVNTEFNHRRLLKSLGPSALDGLPGFHFTTIPDGLPPSDADATQDVPSLCVST
TTTCLEPFCELLSRLSSPTSDGSPPVSCIVSDGVMSFTLKAAERFGLPEVLFWTPSACGFLAYTHYKNLVEKGYTPLKDASQVTNGYLDTTIDW
IEGMKDIRLRDIPSFIRTTDRNDIMLNFFIQETEAAKRGKALIINTFDALEQDSLDALSSVLPPIYTLGPLQLMTKRVQDQNLNAIGSSLWKEDTECI
KWLDTKEPDSVLYVNFGSITTVTAQQLTEFAWGLANSKKQFLWIIRPDLVAGDSATLPEEFVTETKDRSLLAKWGAQEQILNHPAVGGFLTHSG
WNSTIETIGAGKPVICWPFFAEQQTNCRYSCVEWGNGLEIDNNVKRDEVEALVRELIDGEKGKSIKNKALDWKKKAEEATAPGGSSYLNLDKLI
NEVLLH
>Coffea arabica_XM_027257043.1
MGAVAANDLKPHAVCVPYPAQGHINPMLKLAKLLHHKGFHITFVNTEYNHNRLLKSRGPNSLDGLPDFRFEAIPDGLPPTDADSTQDIPSLCDS
TSKTCLAPFSSLLSRLNNAAPDVPPVTCIVSDGCMSFTVEAARQFGLPEALFWTPSACGLLGYTQYSHLVERGYTPLKDMSYLTNGYLETKLD
WIPGMRNDIRLRDLPSFIRTTAADDIMFNFVVHEVNNIPKGTAVVLNSFYELEHDAFDALSAMYPRVFSIGPLQLMLNQIQDEGLKSIGSNLWKE
DPVCIEWLDDKEPNSVVYVNFGSITVMTAHQLTEFAWGLANSKKSFLWIIRPDIVAGDTAMLPPEFVTETKERGKLASWCPQEEVLKHPAIGGF
LTHSGWNSTLESVCGGVPVICWPFFAEQQTNCRYSCVEWEMGMEIDNDVKRDEVELLVRELMDGEKGEKMRNKALEWKSKAEAAAGPDGP
CFQNLDQLINDLLLPKIKLSQ
>Zea mays Bx8_AAL57037.1
MAASCGGRVVVFPFPFQGHFNPVMRLARALHARGVGITVFHTAGARAPDPADYPADYRFVPVPVEVAPELMASEDIAAIVTALNAACEAPFRD
RLSALLSAADGEAGEAGGRVRCVLTDVSWDAVLSAARGLGVPALGVMTASAATFRVYMAYRTLVDKGYLPVREERKDDAVAELPPYRVKDLL
RHETCDLEEFADLLGRVIAAARLSSGLIFHTFPFIEAGTLGEIRDDMSVPVYAVAPLNKLVPAATASLHGEVQADRGCLRWLDAQRARSVLYVS
FGSMAAMDPHEFVELAWGLADAGRPFVWVVRPNLIRGFESGALPDGVEDRVRGRGVVVSWAPQEEVLAHPAVGGFFTHCGWNSTVEAVSE
GVPMICHPRHGDQYGNARYVCHVWKVGTEVAGDQLERGEIKAAIDRLMGGSEEGEGIRKRMNELKIAADKGIDESAGSDLTNLVHLINSY
>Triticum aestivum_XM_044585537.1
MAGAGRRRVVFFPFPFLGHFNPVLRLAGALHARGLAVTVFHTEQRVPDPADYPAGYRFVPLPVEVPPELAASEDIARMGMAMNDAAEAPFRD
RLAALLAEEAGEDGGVLCVITDVVWYSAQAVARELGVPALGIMTASAAIFRVYMAYQTLIDKAYLPVQDARKDDPVEELPPYLVKDLLRHDTSK
LEDFAELLRHSVAGARQSSGLIINTLGAIEAANLERIREDLSVPVFAVAPLHKLAPSAKSTSLGETQADRGCLGWLDTQKPGSVLYVSFGSLAAM
DPHEFVELAWGLALSKRPFVWVVRPKLIRGFESGELPDGLGEELRGRGMIVSWAPQEEVLAHPAVGAFFTHSGWNSTVEAIAEGVPMICHPL
HGDQYGNARYVADVWRVGVEVDGSHRLERGRIKAAIERMMESGEGREIRERMKGLKMAAEDGINERGSSHTHLSDLVALINSF
>Secale cereale _AB548283.1
MAGAPRRVVFFPFPFLGHFNPVLRLAGALHARGLAVTVFHTEQRVPDPADYPADYRFVSLPVEVPPELVTSEDIARMGMAMNDASEAPFRDR
LAALLAKEAEDGGVLCVISDVVWYSAQAVARELGVPALGIMTASAAIFRVYMAYQTLIDKAYLPVQDARKDDPVEELPPYLVKDLLRHDTSRLE
DFAELLRHTVAGARQSSGLIINTLGAIEADNLQQIREDLSVPVFAVAPLHKLAPSAKAGSLGDTQADRGCLDWLDTQNPGTVLYVSFGSLAAMD
PHEFVELAWGLAQSKRPFVWVVRPKLIRGFESGELPDGLGEELSRRGKIVSWAPQEEVLAHPAVGAFFTHSGWNSTVEAISEGVPMICHPLH
GDQYGNARYVSDVWKVGVEVDGTHRLERGSIKAAIERMMDSSEGQEIRERMKGLKMAADDGINERGSSHTHLSDLVALIKSF
>Triticum dicoccoides_XM_037603932.1
MAGAGRRRVVFFPFPFLGHFNPVLRLAGALHARGLAVTVFHTEQRVPDPADYPADYRFVSLPVEVPPELAASEDIARMGMAMNDAAEAPFRD
RLAALLAEEEAEDGGVLCVITDVVWYSAQAVARELGVPALGIMTASAAIFRVYMAYQTLIDKAYLPVQDARKDDPVEELPPYLVKDLLRHDTSKL
EDFAELLRHTVAGARQSSGLIINTLGAIEAANLERIREDLSVPVFAVAPLHKLAPSAKSSSLGETQADRGCLGWLDTQEPGSVLYVSFGSLAAM
DPHEFVELAWGLALSKRPFVWVVRPKLIRGFESGELPDGLGEELRGRGVIVSWAPQEEVLAHPAVGAFFTHSGWNSTVEAIAEGVPMICHPL
HGDQYGNARYVADVWRVGVEVDGSHRLERGSIKAAIGRMMESGEGREIGERMKALKMAAEDGIGERGSSHTHLSDLVALIKSF
>Setaria italica_XM_004972856.2
MASPTIRAGRRVVFFPLPYQGHFNPMLRLAGALHARGVAVTVFHTDLRAPDPTYYPSDYRFVPVPVHVPTELVGSEDIARFVMELNVSCAAPF
KERLAALLAGEEEEEEEAGGVQCVITDVIWYSAQAAARELGVPALGLMTSSAASFRNIMVYPTLIEKCYLPVQEEHKDDPVDVLPPFRVRDLQRI
ETSSLADFASLLEHTVDGGRQSAGLIINTVEAIEAVDLDKIREDMPIPVFPIGPLNMVSPPVESSLYQLQQDRRCLDWLDTKAPGSVIYVSFGSLA
AMDPHEFAELAWGLADSKRPFIWVVRPSLIRGSESGDLPEGFREEIGDRGRIVDWAPQDEVLAHPAVCAFLTHNGWNSTMEAISQGVPMISR
PFFGDQYGNAMFVCHVWRVGVEVQVENQLERGKVRDAIEKLMGSKEGKEIGERMMNLKEIAEKGIKESGSSHTAFLNLADLIFSL
>UGT76B1_1
METRETKPVIFLFPFPLQGHLNPMFQLANIFFNRGFSITVIHTEFNSPNSSNFPHFTFVSIPDSLSEPESYPDVIEILHDLNSKCVAPFGDCLKKLIS
EEPTAACVIVDALWYFTHDLTEKFNFPRIVLRTVNLSAFVAFSKFHVLREKGYLSLQETKADSPVPELPYLRMKDLPWFQTE

DPRSGDKLQIGVMKSLKSSSGIIFNAIEDLETDQLDEARIEFPVPLFCIGPFHRYVSASSSSLLAHDMTCLSWLDKQATNSVIYASLGSIASIDESE
FLEIAWGLRNSNQPFLWVVRPGLIHGKEWIEILPKGFIENLEGRGKIVKWAPQPEVLAHRATGGFLTHCGWNSTLEGICEAIP
MICRPSFGDQRVNARYINDVWKIGLHLENKVERLVIENAVRTLMTSSEGEEIRKRIMPMKETVEQCLKLGGSSFRNLENLIAYILSF
>UGT85A1_1
MGSQIIHNSQKPHVVCVPYPAQGHINPMMRVAKLLHARGFYVTFVNTVYNHNRFLRSRGSNALDGLPSFRFESIADGLPETDMDATQDITALC
ESTMKNCLAPFRELLQRINAGDNVPPVSCIVSDGCMSFTLDVAEELGVPEVLFWTTSGCAFLAYLHFYLFIEKGLCPLKDESYLTKEYLEDTVID
FIPTMKNVKLKDIPSFIRTTNPDDVMISFALRETERAKRASAIILNTFDDLEHDVVHAMQSILPPVYSVGPLHLLANREIEEGSEIGMMSSNLWKEE
MECLDWLDTKTQNSVIYINFGSITVLSVKQLVEFAWGLAGSGKEFLWVIRPDLVAGEEAMVPPDFLMETKDRSMLASWCPQEKVLSHPAIGGF
LTHCGWNSILESLSCGVPMVCWPFFADQQMNCKFCCDEWDVGIEIGGDVKREEVEAVVRELMDGEKGKKMREKAVEWQRLAEKATEHKLG
SSVMNFETVVSKFLLGQKSQD
>Avena strigosa_UGT710E5
MSLLCDRSNGTGRRVVIIPLPYLGHMTPMFRLAAALHARGHAITVLHTELHAPDPASYPSDYRFVGVGVPAAELPAASEDIAAFLVALNDSCAA
AFKDRLAAMLAAEGSVCCVVTDVVWFSAQAAARELGVPALALMTSSAASFRTFMAYPALLANGHLPYDESRRDHLVEELPPFRVRDLQRIDTS
SLDTFAGLLERFVDGARRSSGLILNTFHSIEDQEVRNIRDGLAVPVFPVGPLNKISSSPPPLPQDQDQDQDCLILDWLDTKPTGSVLFVSLGSVA
TVDAQELAELARGLADTGHPFLWVVRPGMIRGGPPDLDLELPADRGMVVPWAPQEEVLRHAAVGAFLTHSGWNSTVEALSEGVPMACLPCF
GDQLGTARYACHVWRVGVEVQGIKRDTVRSAIHRLMGPGAIEEGKEIRERAHDLKSKVGQSIAQGGSSHMALLGLLEKIACF

Supplemental data set 2

Supplementary NMR data

NMR data for isolated BXDs (HBOA-Glc, DIBOA-Glc, DIMBOA-Glc and TRIBOA-Glc

HBOA-Glc ((2*R*)-2-*O*-β-D-glucopyranosyl-2*H*-1,4-benzoxazin-3(4*H*)-one)

^{1}H-NMR (700 MHz, MeOH-d_3) *δ* ppm: 7.07 (*m*, 1H, H-6), 7.003 (*m*, 1H, H-5), 6.998 (*m*, 1H, H-8), 6.92 (*m*, 1H, H-7), 5.74 (*s*, 1H, H-2), 4.68 (*d*, *J*= 7.8 Hz, 1H, H-1'), 3.85 (*dd*, *J*= 12.0, 1.5 Hz, 1H, H-6'), 3.69 (*dd*, *J*= 12.0, 4.6 Hz, 1H, H-6'), 3.36 (*dd*, *J*= 8.5, 8.4 Hz, 1H, H-3'), 3.314 (*m*, 1H, H-5'), 3.31 (*m*, 1H, H-4'), 3.19 (*dd*, *J*= 8.4, 7.8 Hz, 1H, H-2'). ^{13}C-NMR (175 MHz, MeOH-d_3) *δ* ppm: 163.2 (C-3), 142.0 (C-9), 127.1 (C-10), 125.0 (C-5), 124.1 (C-8), 118.9 (C-6), 116.7 (C-7), 103.9 (C-1'), 96.4 (C-2), 78.3 (C-5'), 77.9 (C-3'), 71.9 (C-2'), 71.1 (C-4'), 62.6 (C-6'). The chemical shifts were in agreement with published data (Yin et al., 2008). HRMS (ESI-TOF, negative) *m/z*: calc'd for $C_{14}H_{16}NO_8$ $[M-H]^-$ 326.0875, found 326.0866 .

DIBOA-Glc ((2*R*)-2-*O*-β-D-glucopyranosyl-4-hydroxy-2*H*-1,4-benzoxazin-3(4*H*)-one)

^{1}H-NMR (700 MHz, MeOH-d_3) *δ* ppm: 7.36 (*m*, 1H, H-5), 7.07-7.13 (*m*, 3H, H-6, 7, 8), 5.93 (*s*, 1H, H-2), 4.67 (*d*, *J*= 8.0 Hz, 1H, H-1'), 3.85 (*bd*, *J*= 11.8 Hz, 1H, H-6'), 3.68 (*dd*, *J*= 11.8, 4.5 Hz, 1H, H-6'), 3.28-3.36 (*m*, 3H, H-3', H-4'. H-5'), 3.18 (*dd*, *J*= 8.4, 8.0 Hz, 1H, H-2'). ^{13}C-NMR (175 MHz, MeOH-d_3) *δ* ppm: 158.1 (C-3), 142.2 (C-9), 129.1 (C-10), 125.7 (C-6), 124.1 (C-7), 118.6 (C-8), 114.2 (C-5), 103.6 (C-1'), 97.7 (C-2), 78.4 (C-3'), 77.9 (C-5'), 74.8 (C-2'), 71.0 (C-4'), 62.5 (C-6'). The chemical shifts were in agreement with published data (Yin et al., 2008). HRMS (ESI-TOF, negative) *m/z*: calc'd for $C_{14}H_{16}NO_9$ $[M-H]^-$ 342.0825, found 342.0825. $[\alpha]_D^{20}$ +66.1 (c 0.47, H_2O).

TRIBOA-Glc ((2*R*)-2-*O*-β-D-glucopyranosyl-4-hydroxy-7-hydroxy-2*H*-1,4-benzoxazin-3(4*H*)-one)

^{1}H-NMR (700 MHz, MeOH-d_3) *δ* ppm: 7.17 (*d*, *J*= 8.4 Hz, 1H, H-5), 6.55 (*d*, *J*= 2.6 Hz, 1H, H-8), 6.54 (*dd*, *J*= 8.4, 2.6 Hz, 1H, H-8), 5.88 (*s*, 1H, H-2), 4.66 (*d*, *J*= 7.9 Hz, 1H, H-1'), 3.86 (*dd*, *J*= 12.1, 1.4 Hz, 1H, H-6'), 3.69 (*dd*, *J*= 12.1, 4.7 Hz, 1H, H-6'), 3.34 (*dd*, *J*= 8.6, 8.4 Hz, 1H, H-3'), 3.294 (*m*, 1H, H-4'), 3.289 (*m*, 1H, H-5'), 3.18 (*dd*, *J*= 8.4, 7.9 Hz, 1H, H-2'). ^{13}C-NMR (175 MHz, MeOH-d_3) *δ* ppm: 157.2 (C-3), 156.3 (C-7), 143.3 (C-9), 121.7 (C-10), 115.0 (C-5), 110.7 (C-6), 105.8 (C-8), 103.6 (C-1'), 98.0 (C-2), 78.4 (C-5'), 77.9 (C-3'), 74.8 (C-2'), 71.0 (C-4'), 62.5 (C-6'). HRMS (ESI-TOF, positive) *m/z*: calc'd for $C_{14}H_{18}NO_{10}$ $[M+H]^+$ 360.0925, found 360.0919.

DIMBOA-Glc ((2*R*)-2-*O*-β-D-glucopyranosyl-4-hydroxy-7-methoxy-2*H*-1,4-benzoxazin-3(4*H*)-one)

^{1}H-NMR (700 MHz, Acetone-d_6) *δ* ppm: 7.27 (*d*, *J*= 8.9 Hz, 1H, H-5), 6.76 (*d*, *J*= 2.6 Hz, 1H, H-8), 6.70 (*dd*, *J*= 8.9, 2.6 Hz, 1H, H-6), 5.85 (*s*, 1H, H-2), 4.71 (*d*, *J*= 8.0 Hz, 1H, H-1'), 3.88 (*dd*, *J*= 11.7, 2.6 Hz, 1H, H-6'), 3.78 (*s*, 3H, O$\underline{CH_3}$), 3.65 (*dd*, *J*= 11.7, 5.8 Hz, 1H, H-6'), 3.40 (*dd*, *J*= 9.0, 9.0 Hz, 1H, H-3'), 3.38 (*m*, 1H, H-5'), 3.30 (*dd*, *J*= 9.2, 9.0 Hz, 1H, H-4'), 3.17 (*dd*, *J*= 9.0, 8.0 Hz, 1H, H-2'). ^{13}C-NMR (175 MHz, Acetone-d_6) *δ* ppm: 156.9 (C-7), 154.8 (C-3), 141.9 (C-9), 122.0 (C-10), 113.6 (C-5), 108.5 (C-6), 103.6 (C-8), 97.2 (C-2), 103.0 (C-1'), 77.3 (C-5'), 77.0 (C-3'), 73.8 (C-2'), 70.4 (C-4'), 62.0 (C-6'). The chemical shifts were in agreement with published data (Wouters et al., 2014). HRMS (ESI-TOF, positive) *m/z*: calc'd for $C_{15}H_{20}NO_{10}$ $[M+H]^+$ 372.0930, found 372.0924.

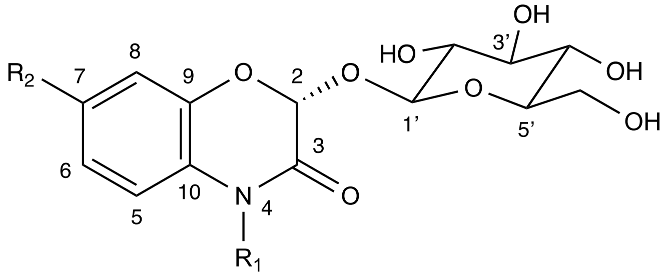

R_1 = OH, R_2 = H DIBOA-Glc
R_1 = H, R_2 = H HBOA-Glc
R_1 = OH, R_2 = OCH_3 DIMBOA-Glc
R_1 = OH, R_2 = OH TRIBOA-Glc

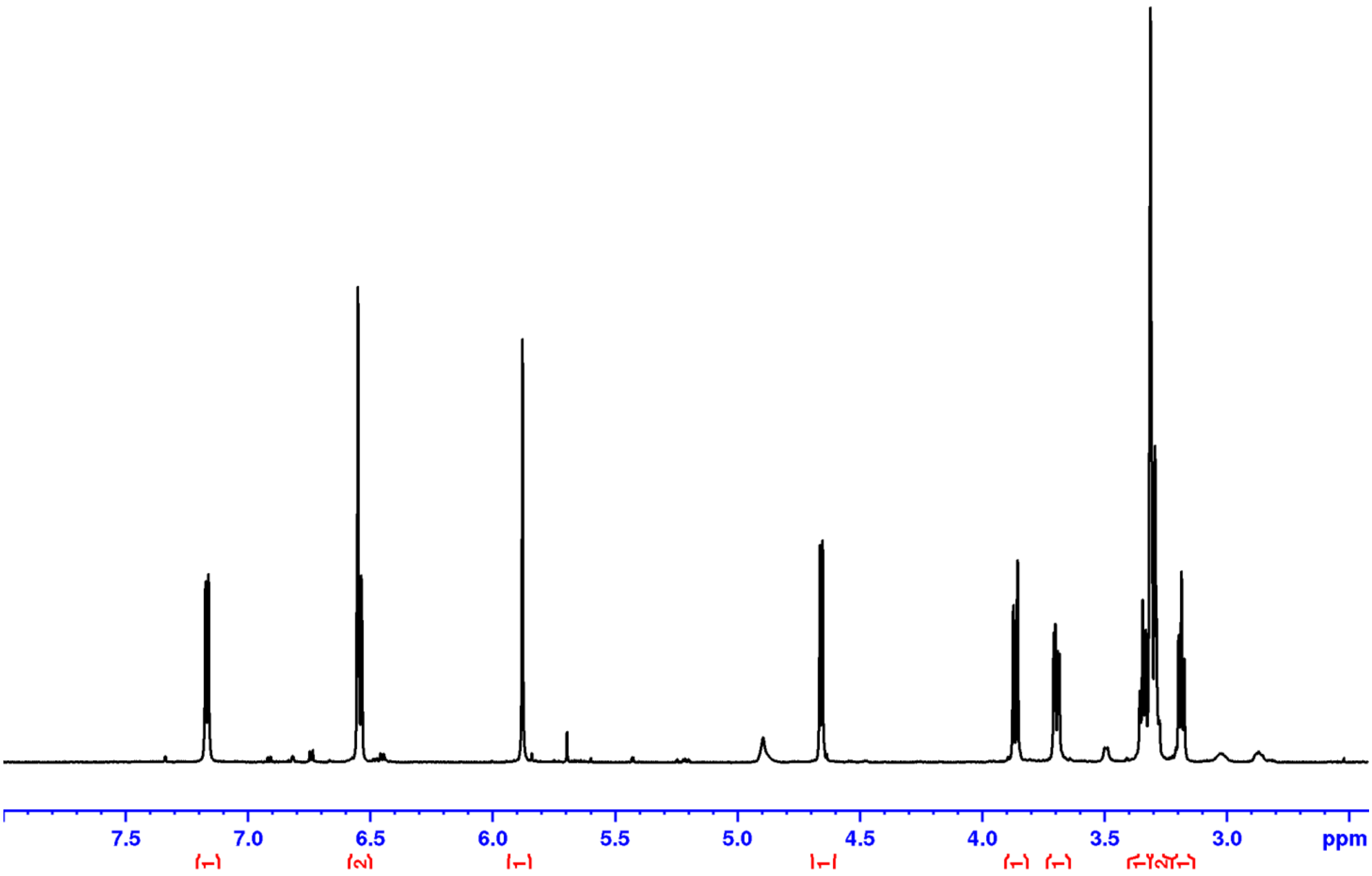

TRIBOA-Glc. ^{1}H NMR with water suppression, full range in MeOH-d_3

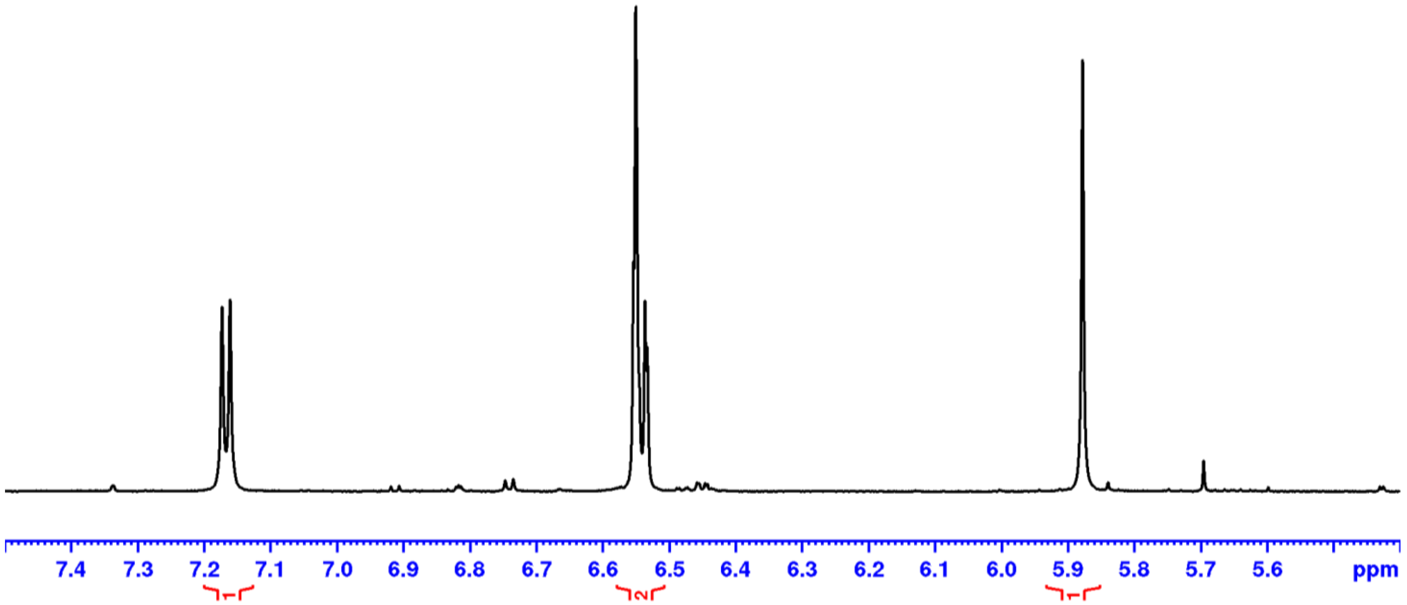

TRIBOA-Glc. ^{1}H NMR with water suppression, aromatic range in MeOH-d_3

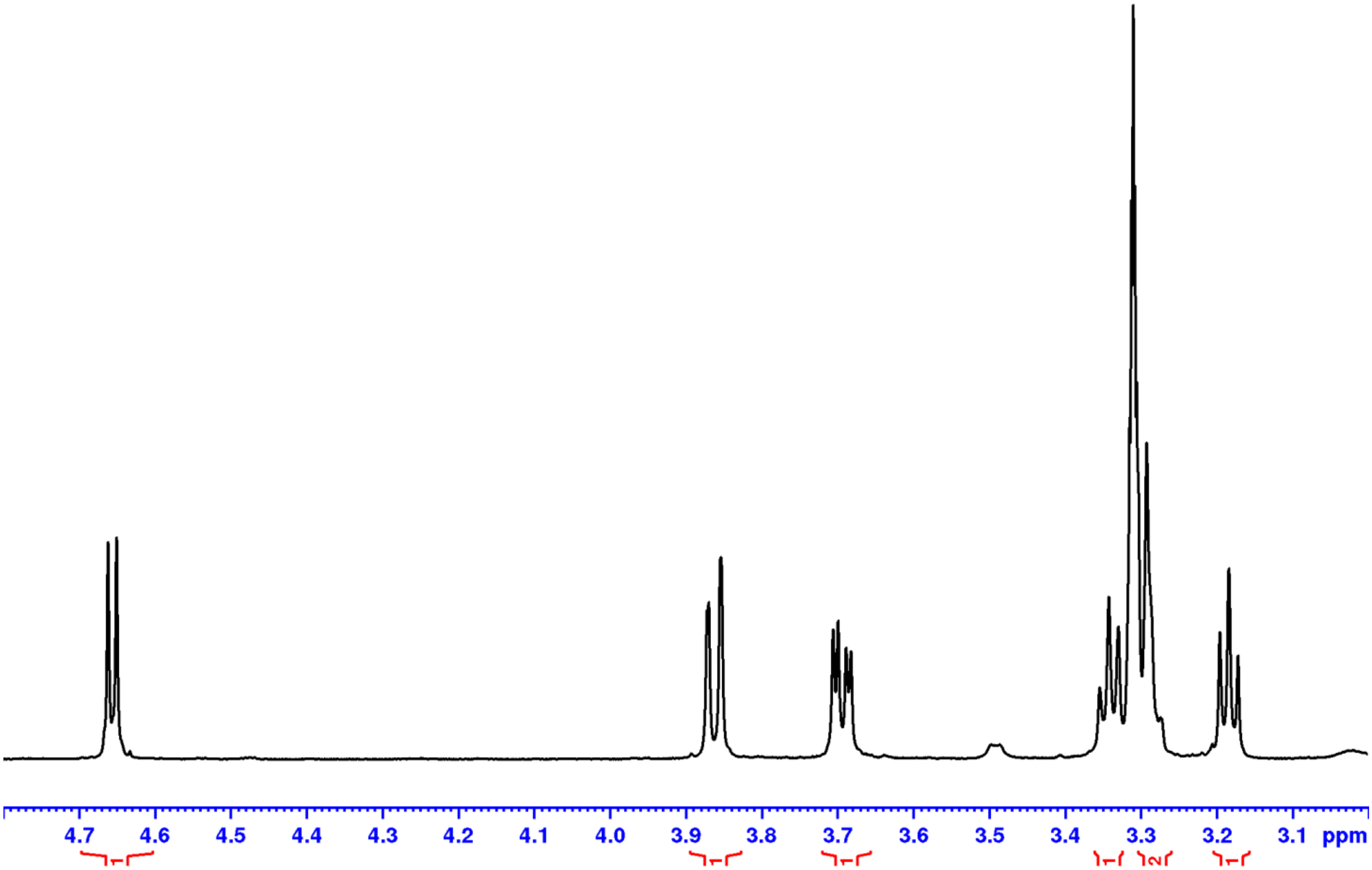

TRIBOA-Glc. ^{1}H NMR with water suppression, aliphatic range in MeOH-d_3

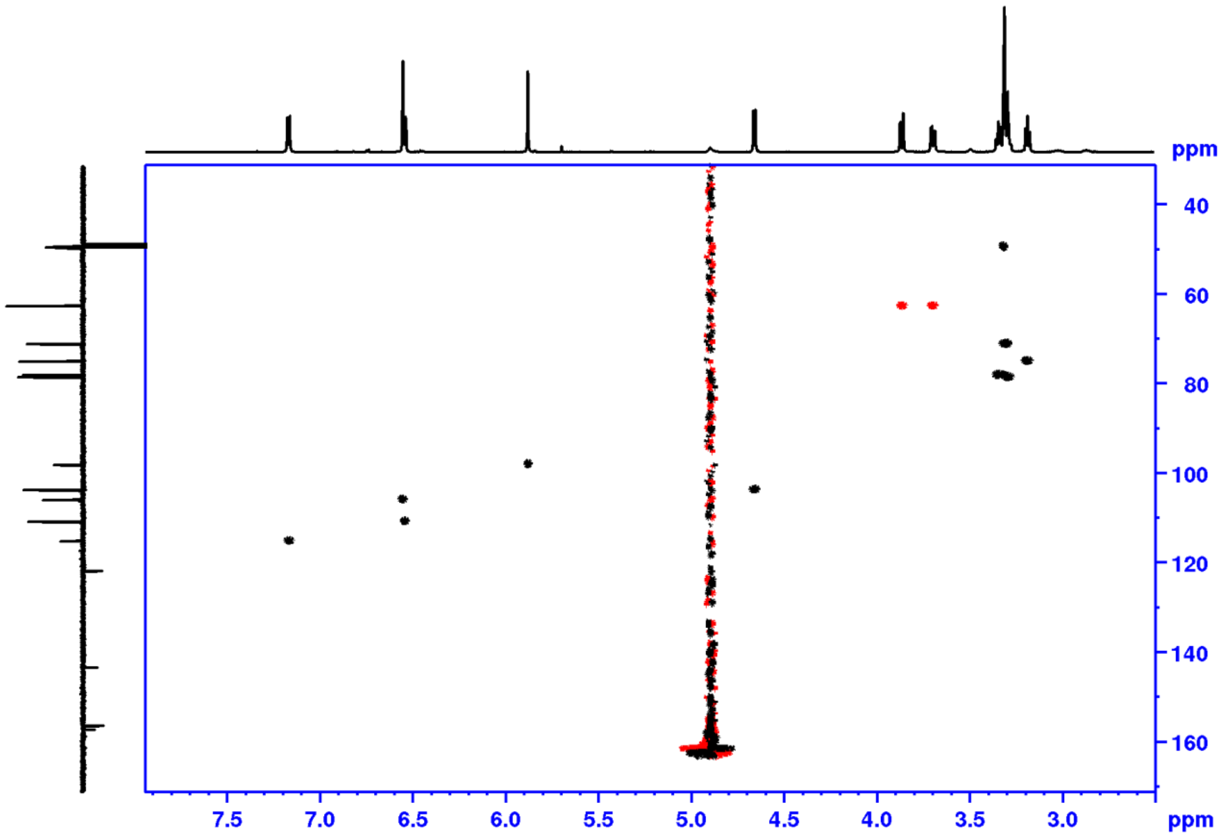

TRIBOA-Glc. phase sensitive HSQC, full range in MeOH-d_3

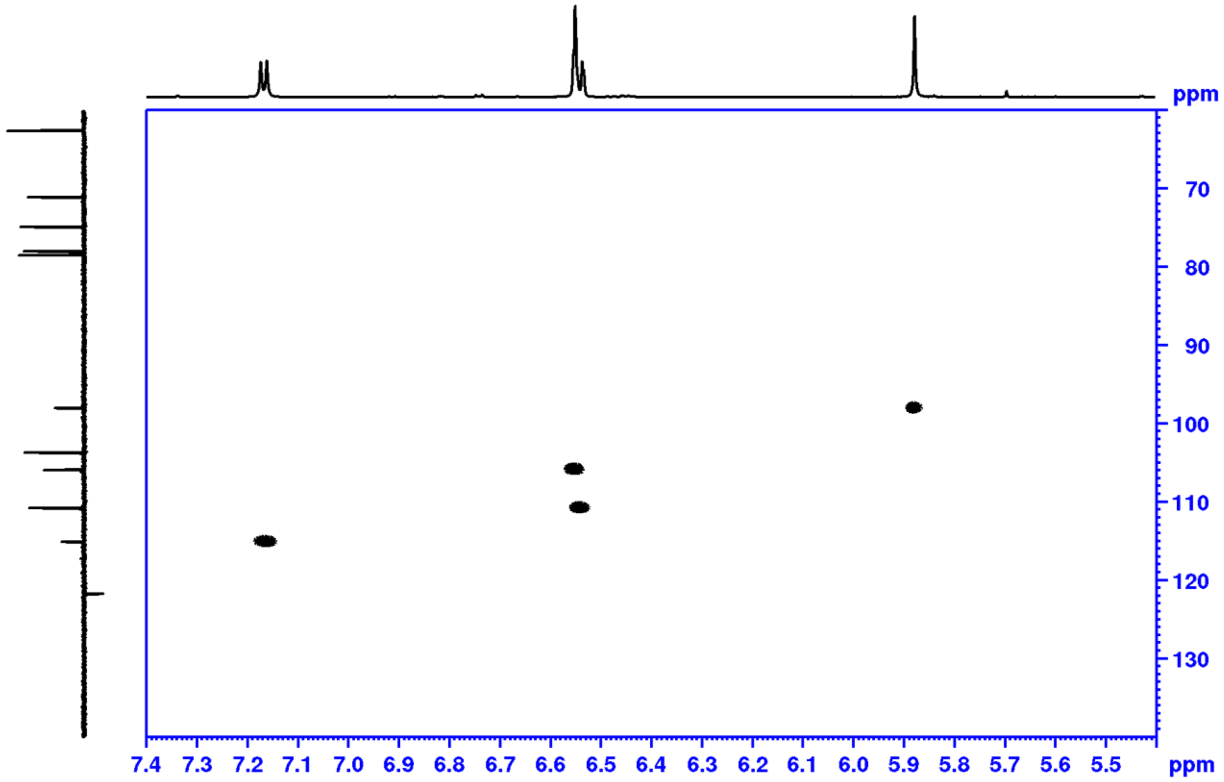

TRIBOA-Glc. phase sensitive HSQC, aromatic range in MeOH-d_3

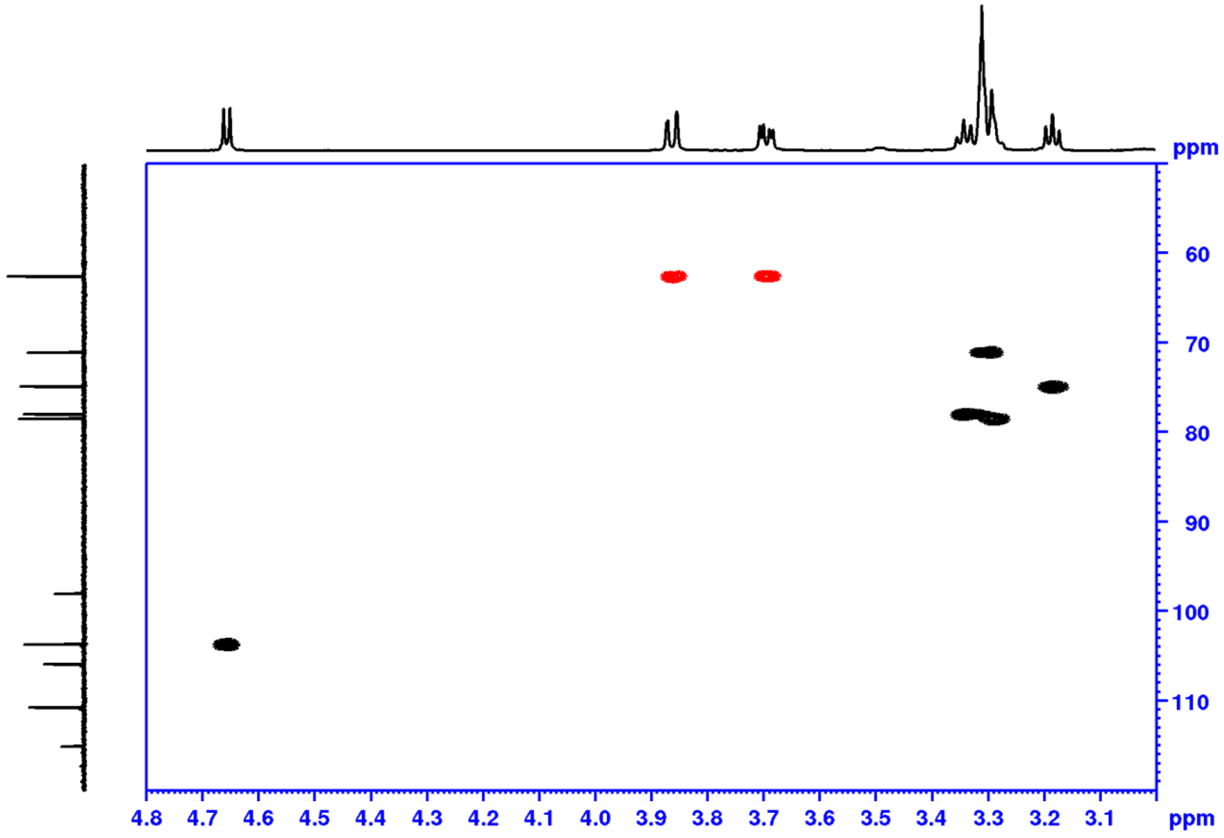

TRIBOA-Glc. phase sensitive HSQC, aliphatic range in MeOH-d_3

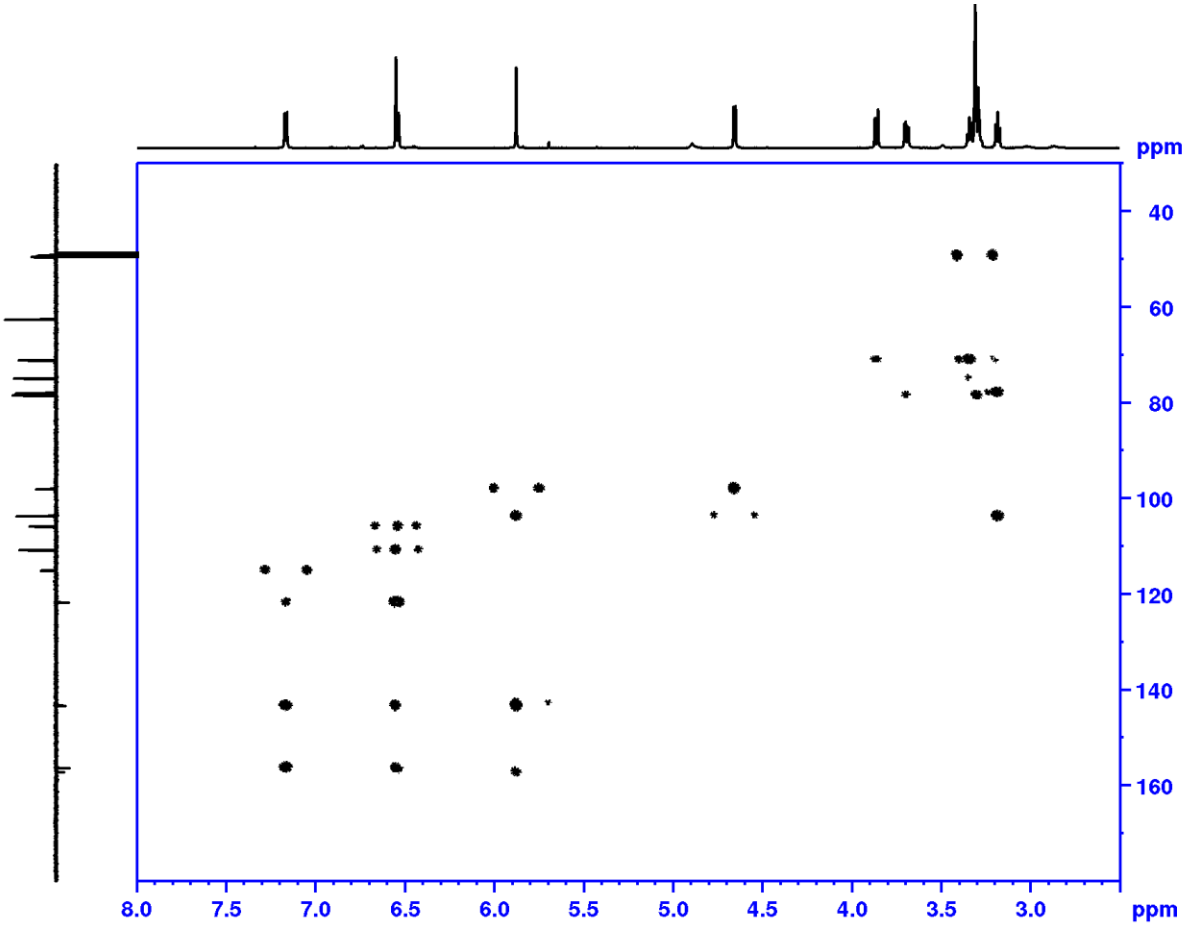

TRIBOA-Glc. HMBC, full range in MeOH-d_3

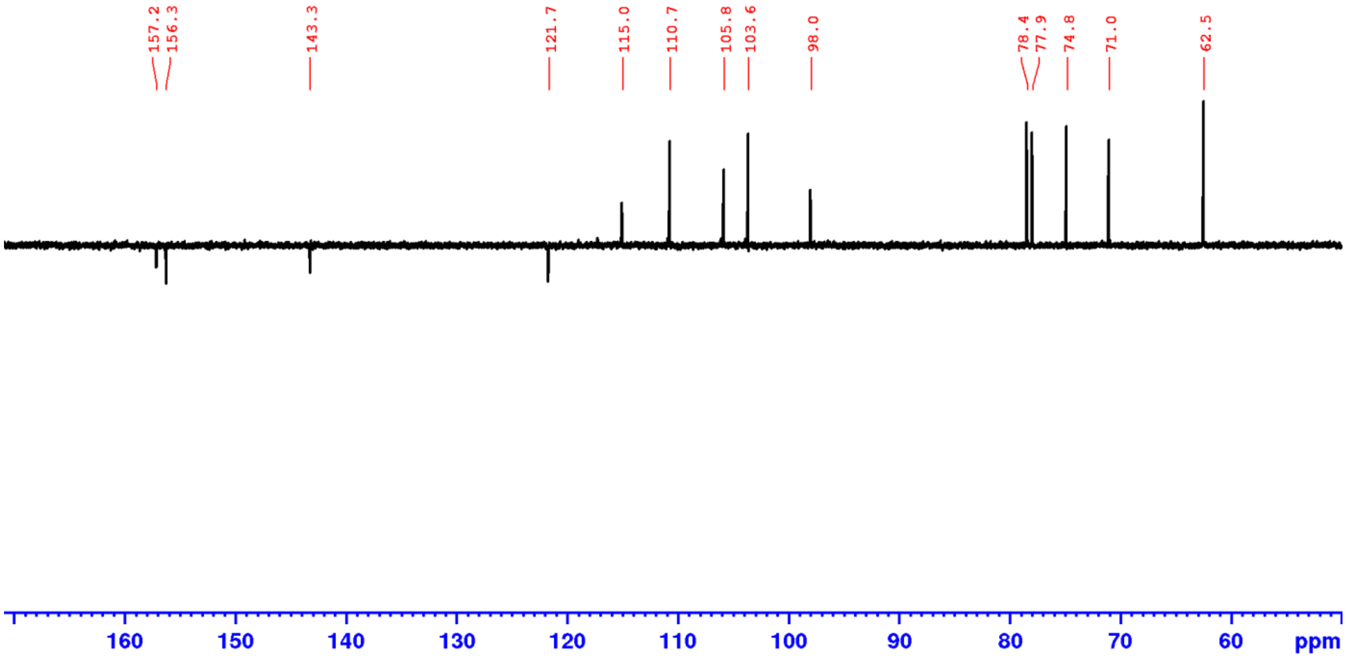

TRIBOA-Glc. ^{13}C (DEPTQ), full range in MeOH-d_3

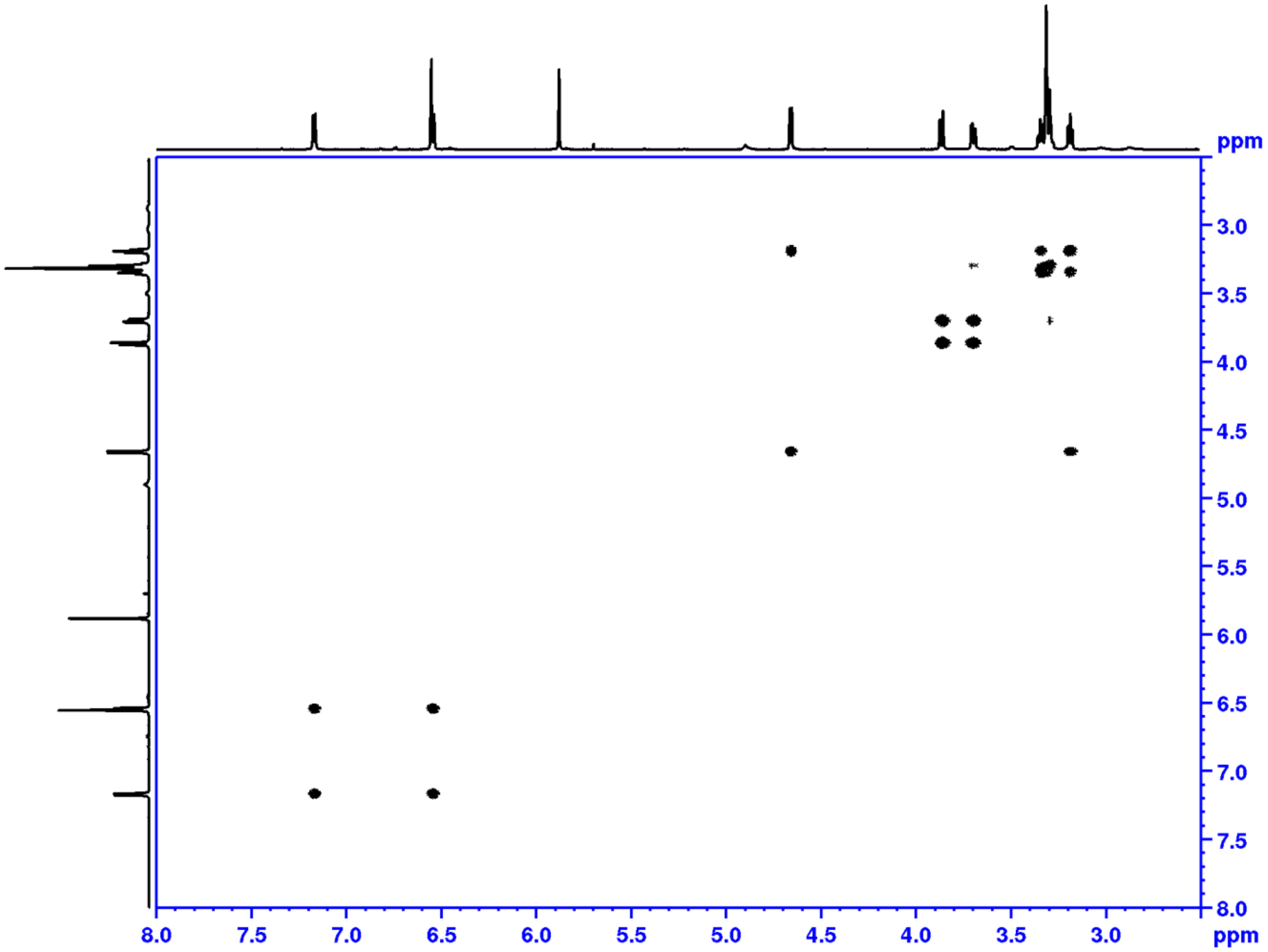

TRIBOA-Glc. ^1H-^1H DQF COSY with water suppression, full range in MeOH-d_3

Synthesis and Characterization of compounds

NMR spectra were measured on a 400 MHz Bruker Avance III HD spectrometer (Bruker Biospin GmbH, Rheinstetten, Germany). CD3OD and $CDCl_3$ were used as solvents. NMR spectra were referenced to the residual solvent signals at δ_H 3.31 and δ_C 49.0 for CD3OD and δ_H 7.26 and δ_C 77.0 for $CDCl_3$. For spectrometer control and data processing Bruker TopSpin ver. 3.6.1 was used.

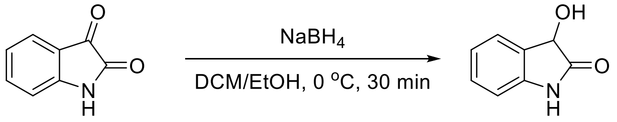

isatin

3-hydroxyindolin-2-one
(**3HI2O**)

^{1}H-NMR (400 MHz, $CDCl_3$) δ 7.44 (d, *J* = 7.4 Hz, 1H), 7.29 (d, *J* = 7.7 Hz, 1H), 7.10 (t, *J* = 7.6 Hz, 1H), 6.86 (d, *J* = 7.8 Hz, 1H), 5.08 (s, 1H);
^{13}C-NMR (101 MHz, $CDCl_3$) δ 178.0, 140.8, 129.9, 127.1, 125.6, 123.2, 110.1, 70.0.

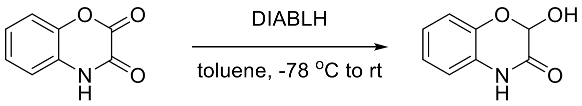

2,3-dioxo-1,4-benzoxazine

HBOA

^{1}H-NMR (400 MHz, CD_3OD) δ 6.93 (m, 4H), 5.52 (s, 1H);
^{13}C-NMR (101 MHz, CD_3OD) δ 165.3, 142.4, 127.5, 124.9, 123.7, 118.8, 116.8, 92.1.

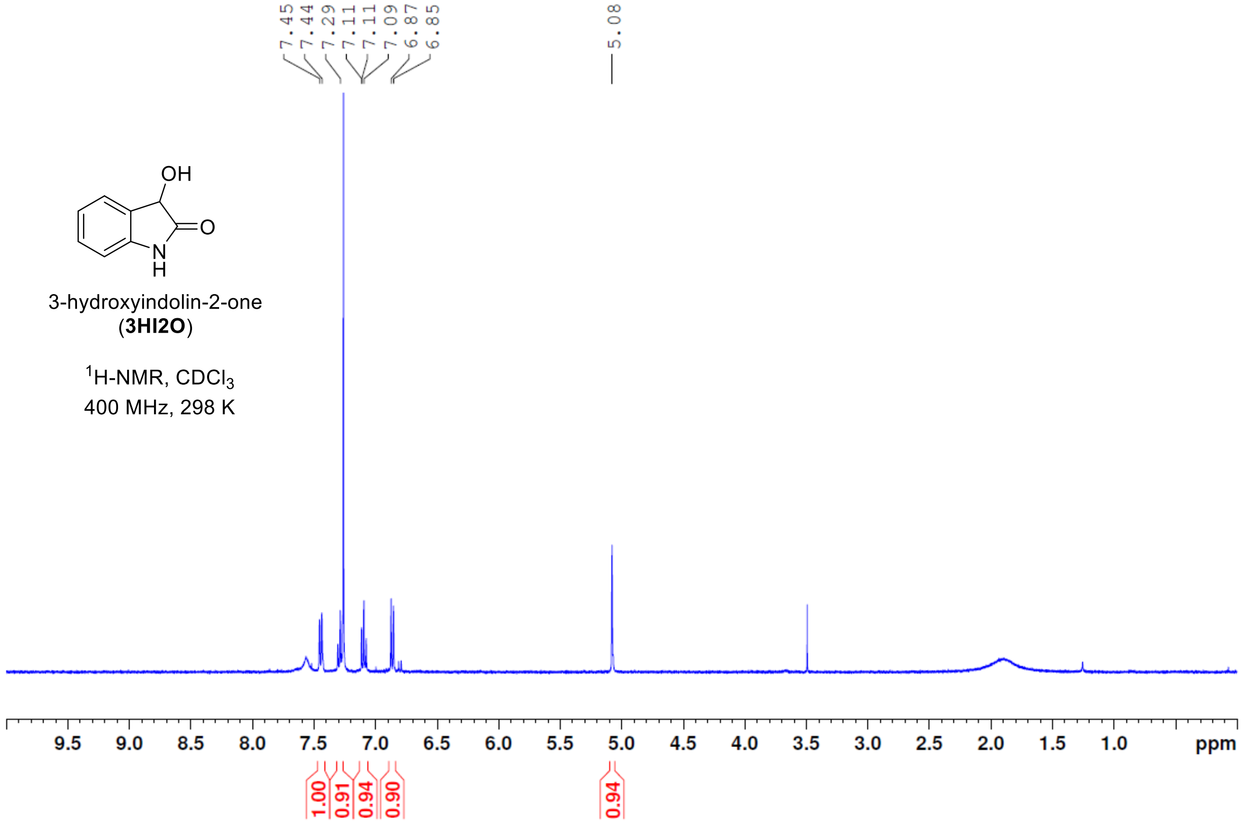

3HI2O, ^{1}H-NMR, CDCl3 400MHz, 298K.

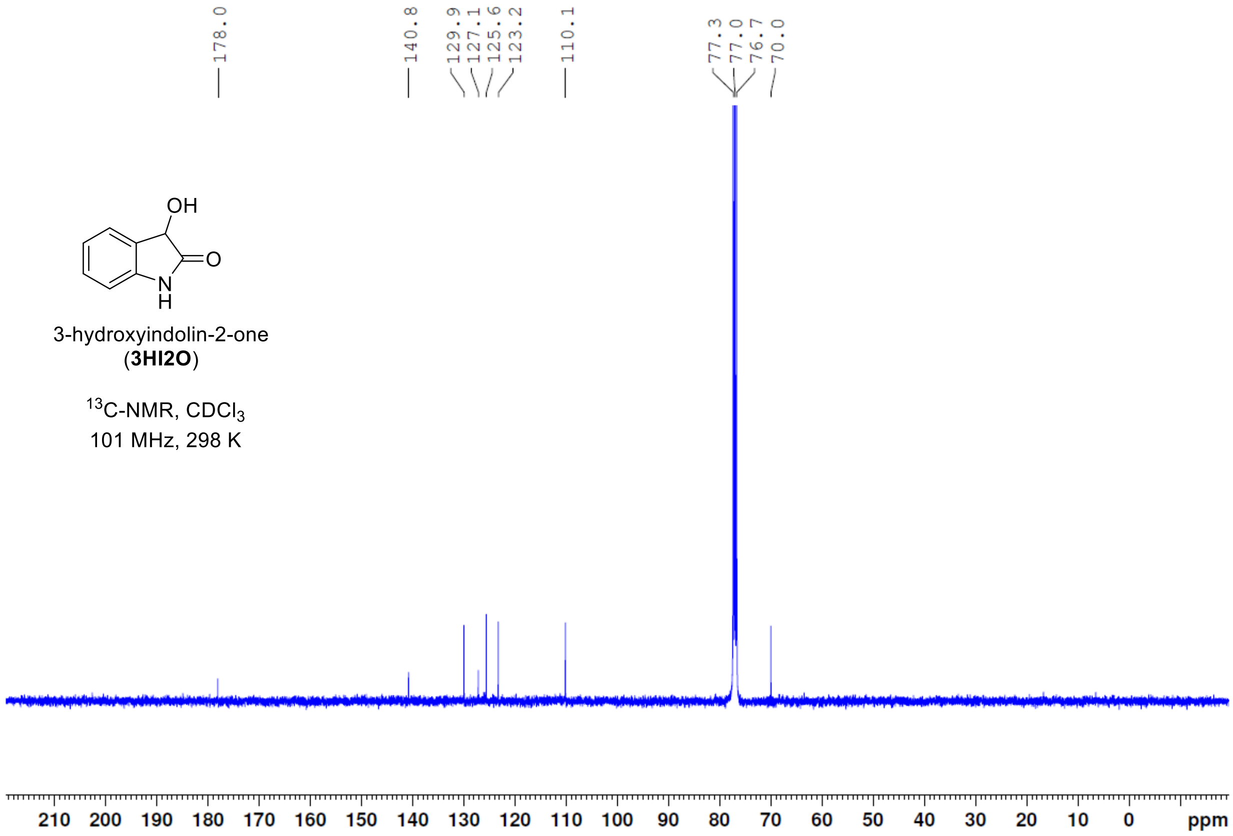

SI Figure 22: **3HI2O**, ^{13}C-NMR, CDCl3 101MHz, 298K.

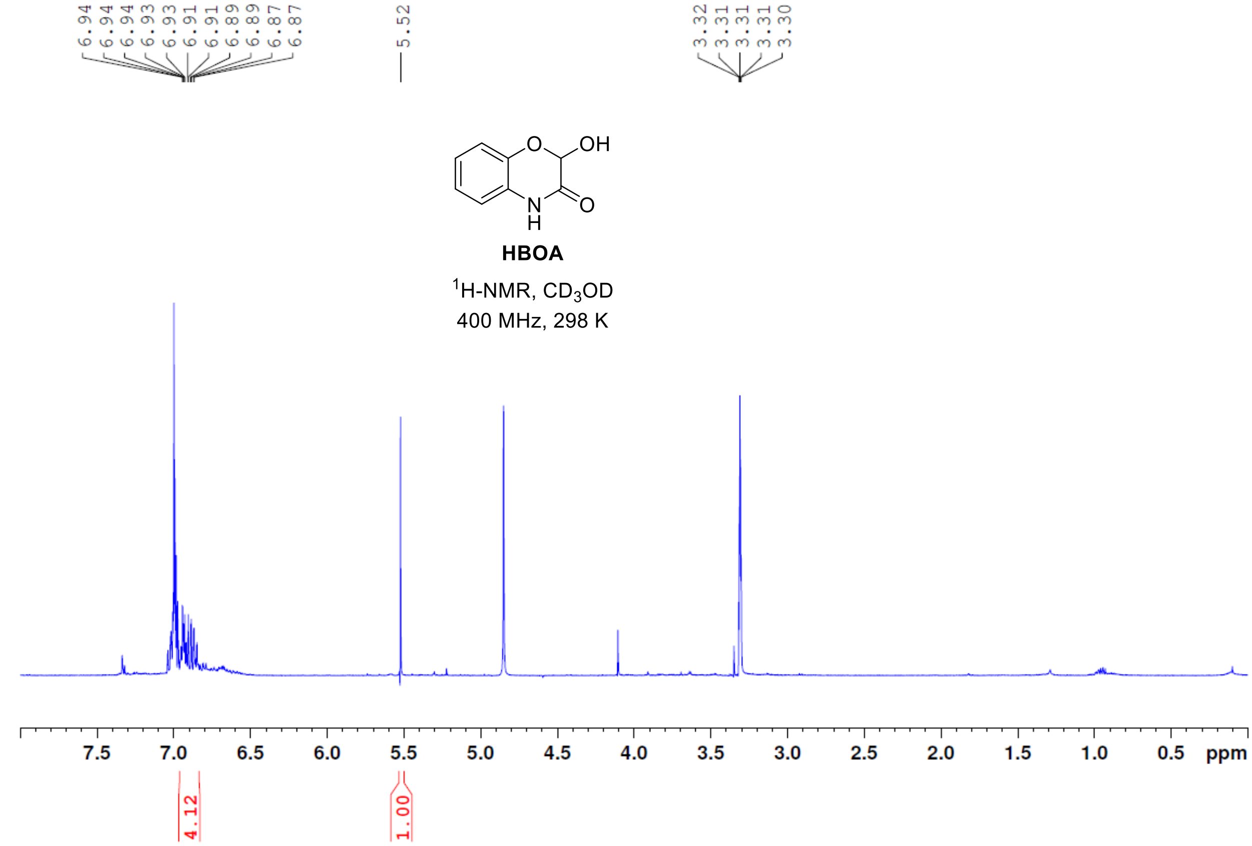

HBOA, ^{1}H-NMR, CDC3OD 400MHz, 298K.

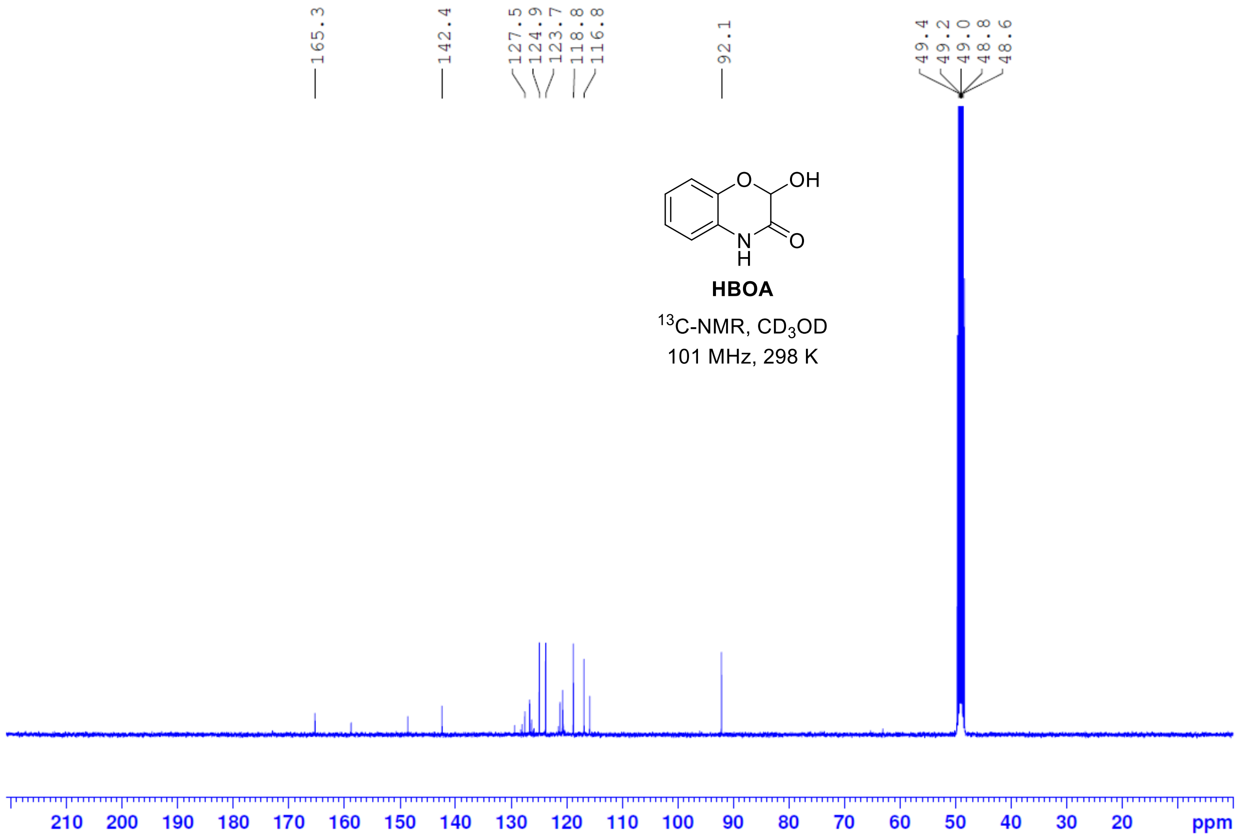

HBOA, ^{13}C-NMR, CD3OD 101MHz, 298K.

Literature:

Yin H, Zhang S, Luo X, Liu Y (2008) Preparative isolation and purification of two benzoxazinoid glucosides from *Acanthus ilicifolius* L. by high-speed counter-current chromatography. *J Chromatogr A* 1205: 177-181

Wouters FC, Reichelt M, Glauser G, Bauer E, Erb M, Gershenzon J, Vassao GV (2014) Reglucosylation of the Benzoxazinoid DIMBOA with Inversion of Stereochemical Configuration is a Detoxification Strategy in Lepidopteran Herbivores. *Angew Chem Int Ed* 53: 11320-11324
